# Challenging nickel-catalysed amine arylations enabled by tailored ancillary ligand design

**DOI:** 10.1038/ncomms11073

**Published:** 2016-03-23

**Authors:** Christopher M. Lavoie, Preston M. MacQueen, Nicolas L. Rotta-Loria, Ryan S. Sawatzky, Andrey Borzenko, Alicia J. Chisholm, Breanna K. V. Hargreaves, Robert McDonald, Michael J. Ferguson, Mark Stradiotto

**Affiliations:** 1 Department of Chemistry, Dalhousie University, 6274 Coburg Road, PO Box 15000, Halifax, Nova Scotia, Canada B3H 4R2; 2 X-Ray Crystallography Laboratory, Department of Chemistry, University of Alberta, Edmonton, Alberta Canada T6G 2G2

## Abstract

Palladium-catalysed C(*sp*^2^)–N cross-coupling (that is, Buchwald–Hartwig amination) is employed widely in synthetic chemistry, including in the pharmaceutical industry, for the synthesis of (hetero)aniline derivatives. However, the cost and relative scarcity of palladium provides motivation for the development of alternative, more Earth-abundant catalysts for such transformations. Here we disclose an operationally simple and air-stable ligand/nickel(II) pre-catalyst that accommodates the broadest combination of C(*sp*^2^)–N coupling partners reported to date for any single nickel catalyst, without the need for a precious-metal co-catalyst. Key to the unprecedented performance of this pre-catalyst is the application of the new, sterically demanding yet electron-poor bisphosphine PAd-DalPhos. Featured are the first reports of nickel-catalysed room temperature reactions involving challenging primary alkylamine and ammonia reaction partners employing an unprecedented scope of electrophiles, including transformations involving sought-after (hetero)aryl mesylates for which no capable catalyst system is known.

## Abstract (web summary)

The development of highly effective Earth-abundant catalysts for C(*sp* ^2^)-N cross-coupling represents an on-going challenge in synthetic chemistry. Here, the authors report a nickel complex containing a bisphosphine ancillary ligand allowing room-temperature couplings of amines and ammonia with a range of electrophiles.

## Graphical abstract

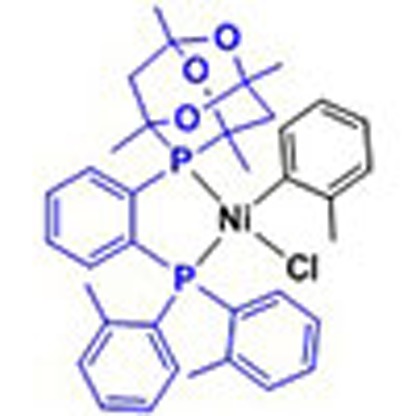

Homogeneous transition metal catalysis is employed broadly in synthetic organic chemistry, including in the pharmaceutical industry for the preparation of active pharmaceutical ingredients on bench-top and production scales^1^. The development of useful catalysts of this type that offer broad substrate scope is often made possible through the application of appropriately designed ancillary ligands; when coordinated to the reactive metal of interest, such ligands can dramatically enhance catalytic performance (for example, activity, selectivity and lifetime). The palladium-catalysed cross-coupling of NH substrates and (hetero)aryl (pseudo)halides (that is, Buchwald–Hartwig amination^2^,^3^,^4^,^5^,^6^, BHA; Fig. 1a) is a well-established C(*sp*^2^)-N bond-forming methodology whose rapid evolution can be attributed in large part to advances in ancillary ligand design. Indeed, BHA chemistry is now employed widely the synthesis of biologically active molecules and functional materials, including in the pharmaceutical industry with applications ranging from the rapid diversification of small-molecule libraries to larger-scale production^1^,^7^,^8^,^9^.

In the years following the initial development of BHA chemistry, the preponderance of empirical data revealed that optimally configured ancillary ligands must be chosen when using challenging substrates such as (hetero)aryl chlorides, so as to promote the formation of electron-rich Pd(0) complexes that are activated towards otherwise challenging C(*sp*^2^)–Cl oxidative additions^10^,^11^, whereas also enabling C–N bond reductive elimination to afford the (hetero)aryl amine product. The steric demands of these two key mechanistic steps suggest the application of bulky ancillary ligands, to favour low-coordination^12^ and to encourage reductive elimination. However, the ancillary ligand electronic requirements for oxidative addition and reductive elimination are orthogonal, with strongly electron-donating ligands favouring oxidative addition, and less electron-donating ligands favouring reductive elimination^13^. Nonetheless, electron-rich and sterically demanding alkylphosphine and *N*-heterocyclic carbene ancillary ligands have in general proven to be most effective in challenging BHA chemistry, collectively giving rise to palladium catalysts that are capable of promoting the cross-coupling of a useful spectrum of aryl electrophiles and NH substrates^6^.

However, in recent years, there has been a shift in focus away from the use of catalysts based on rare and expensive precious metals such as palladium^14^, including in the pharmaceutical industry where the cost of both palladium and the required electron-rich ancillary ligands, as well as the potential for bulk palladium supply limitations, can be problematic. Notwithstanding some selected recent reports of copper-catalysed C(*sp*^2^)–N cross-couplings (that is, Ullmann C–N coupling) of ammonia^15^ or primary alkylamines^16^ at elevated temperatures (>100 °C), which are of relevance to our work presented herein, copper catalysis is typically not effective with low-cost and widely available (hetero)aryl chlorides or sulfonates^17^. Conversely, nickel catalysis offers the potential to supplant BHA protocols, being significantly less rare and expensive than palladium and having a proven track-record in alternative cross-couplings involving such challenging electrophiles^18^,^19^,^20^,^21^,^22^,^23^. The first report of nickel-catalysed C(*sp*^2^)–N cross-couplings involving aryl chlorides followed shortly after the establishment of BHA protocols^24^; nonetheless, nickel has received scant attention relative to palladium in such applications, due in part to the perception that the accessible oxidation state range for nickel^21^,^25^ would lead to complex reactivity profiles. However, recent studies support a Ni(0)/Ni(II) catalytic cycle in C(*sp*^2^)–N cross-couplings involving phosphine-ligated nickel species with aryl (pseudo)halides, much like that traversed by palladium in BHA chemistry^26^,^27^,^28^. This augers well for the application of rational ancillary ligand design as a means of developing highly effective nickel catalysts that are competitive with, and ideally superior to, palladium in such synthetically important amination reactions.

Despite such opportunities, the design of ancillary ligands specifically for use in enabling nickel-catalysed C(*sp*^2^)–N cross-couplings has not been reported. Rather, the most common strategy employed has involved the screening of ancillary ligands that have proven useful in related palladium catalysis. Such an approach has proven to be reasonably effective for the cross-coupling of some secondary amine substrates, with nickel catalyst systems based on triphenylphosphine^29^, 1,1′-bis(diphenylphosphino)ferrocene^24^,^30^, *N*-heterocyclic carbenes^31^,^32^, including 1,3-bis-(2,6-diisopropylphenyl)imidazol-2-ylidene (IPr)^33^, and also phenanthrolines^24^,^34^ exhibiting desirable performance. Conversely, nickel catalysts capable of promoting the cross-coupling of alternative high-value NH substrates (for example, primary alkylamines, ammonia, azoles and others) are rare, limited in scope, and commonly require forcing reaction conditions.

Given the smaller atomic radius and distinct electronic properties of nickel, it is unlikely that the ‘re-purposing' of ancillary ligands that function well with palladium will be a universally effective strategy in the pursuit of superlative nickel catalysts for challenging C(*sp*^2^)–N cross-couplings; the design and application of new ancillary ligands targeted specifically for use in nickel catalysis represents a promising and complementary approach. More specifically, unlike the bulky *electron-rich* ancillary ligands that have proven optimal for use with palladium, we envisioned that sterically demanding yet relatively *electron-poor* bisphosphines might be well-suited for use with nickel, given the greater propensity for C(*sp*^2^)-Cl oxidative additions to Ni(0) versus Pd(0)^10^,^35^,^36^, and the associated potential for rate-limiting C(*sp*^2^)–N reductive elimination (Fig. 1b).

We report here on the development of the inexpensive, air-stable *ortho*-phenylene bisphosphine ligand **L1** (PAd-DalPhos) that embodies these design criteria (Fig. 1b). The derived air-stable pre-catalyst (**L1**)Ni(*o*-tolyl)Cl (**C1**) is remarkably useful in otherwise challenging C(*sp*^2^)–N cross-coupling chemistry at relatively low catalyst loadings, accommodating the broadest combination of (hetero)aryl (pseudo)halide and NH coupling partners reported to date for any single nickel catalyst, without the need for precious metal co-catalysts or other additives. Included herein are the first reports of room-temperature nickel-catalysed cross-couplings involving primary alkylamines and ammonia, as well as examples of sought-after ammonia monoarylations employing (hetero)aryl mesylate electrophiles, for which no capable catalyst system of any type is known. Furthermore, we demonstrate the unusual versatility of **C1** in the cross-coupling of carbazole, indole and 7-azaindole.

## Results

### Ancillary ligand design and catalyst screening

**Figure 2:**
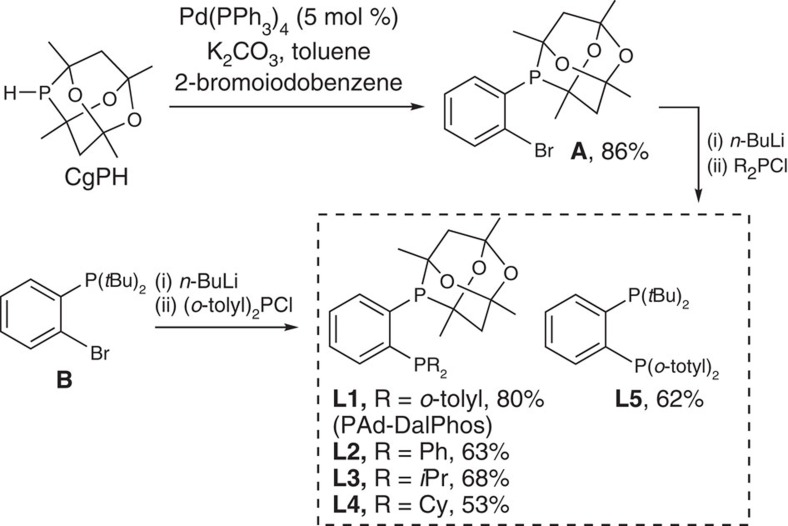
Synthesis of the bisphosphine ligands L1–L5. **L1–L4** are prepared from **A**, while **L5** is prepared from **B**.

In the pursuit of bulky electron-poor bisphosphine ancillary ligands that would function well in nickel-catalysed amination chemistry, possibly by promoting C(*sp*^2^)–N reductive elimination, we sought tunable *ortho*-phenylene bisphosphines featuring the 1,3,5,7-tetramethyl-2,4,8-trioxa-6-phosphaadamantane (CgP) group with an adjacent phosphorus donor fragment that could be modified easily. Prior structural analyses have established that CgP is as sterically demanding as a P(*t*Bu)_2_ fragment, while also being a relatively electron-poor phosphorus donor comparable to a P(OR)_2_ group^37^, thus making such a fragment well-suited to our ancillary ligand design interests. The use of CgPPh and related monophosphine ancillary ligand derivatives in palladium-catalysed cross-couplings dates to 2003 (ref. 38), with subsequent reports involving (carbonylative) BHA chemistry featuring only a limited scope of secondary amines and anilines^39^,^40^. Conversely, journal publications documenting the application of CgP-based ligands in nickel catalysis are limited to a single report focused on CgPOR-type phosphinites for the hydrocyanation of 3-pentenenitrile^41^. The new air-stable phosphaadamantane ligands **L1**–**L4** (Fig. 2) were prepared straightforwardly in a two-step synthesis from relatively inexpensive, commercially available components via the common synthetic intermediate **A**. The new air-stable bisphosphine **L5**, a complementary variant of **L1** featuring a P(*t*Bu)_2_ donor in place of the CgP group, was prepared analogously from synthon **B**.

Ammonia ranks among the most widely produced commodity chemicals^42^, and selective ammonia monoarylation is sought-after as an atom-economical and scalable route to (hetero)anilines, including in the pharmaceutical industry. However, the development of such transformations has proven to be extremely challenging^43^,^44^ owing to uncontrolled polyarylation arising due to the primary (hetero)aniline products acting as contending substrates versus ammonia. Our group^45^, as well as Hartwig and co-workers^28^, independently disclosed the first and only examples of nickel-catalysed ammonia monoarylation, in which relatively electron-rich JosiPhos-type ligands were employed. Notwithstanding such progress, the failure of other top-performing bulky and electron-rich ancillary ligands^45^ from the domain of palladium-catalysed ammonia monoarylation (for example, CyPF-*t*Bu JosiPhos^46^,^47^, Mor-DalPhos^48^, BippyPhos^49^) underscores the inconsistent nature of simply re-purposing ancillary ligands from palladium chemistry in the pursuit of nickel catalysts for such challenging C(*sp*^2^)–N cross-couplings. Moreover, there are a number of drawbacks associated with the reported Ni/JosiPhos catalyst systems^28^,^45^, including: the costly nature of the unnecessarily enantiopure JosiPhos ligand class; negligible demonstrated scope involving synthetically useful phenol-derived electrophiles (for example, sulfonates); and the need for elevated reaction temperatures (⩾100 °C).

**Figure 3:**
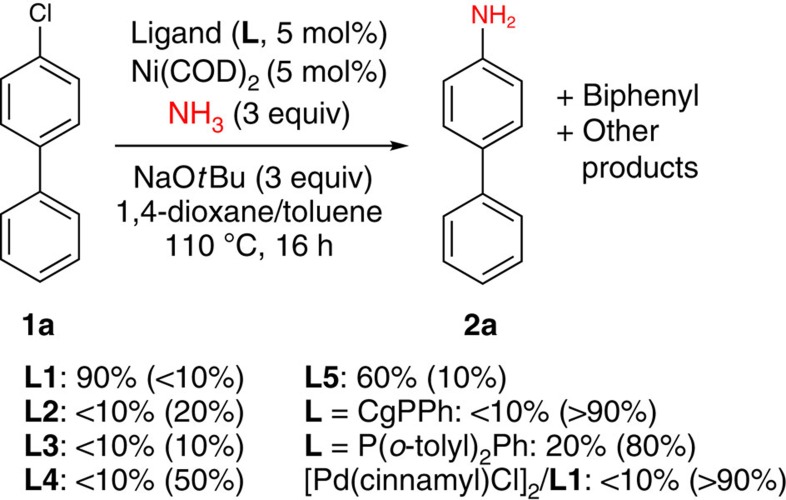
Preliminary ligand screen in the nickel-catalysed monoarylation of ammonia. Reactions employed 4-chlorobiphenyl (0.12 mmol), 0.5 M stock solutions of ammonia in 1,4-dioxane (0.72 ml) and toluene (1.00 ml). Conversions estimated on the basis of gas chromatographic data, reported as % 4-aminobiphenyl (% 4-chlorobiphenyl unreacted); mass balance attributable to biphenyl and/or other unidentified products; COD, 1,5-cyclooctadiene.

Against this background, we selected the monoarylation of ammonia with 4-chlorobiphenyl (**1a**) as a testing ground on which to evaluate the catalytic abilities of **L1**–**L5** and related ligands (Fig. 3). Although Ni(COD)_2_/**L2**-**L4** mixtures (COD=1,5-cyclooctadiene) performed poorly, use of Ni(COD)_2_/**L1** afforded high conversion to the desired 4-aminobiphenyl (**2a**). These findings are in keeping with our view that sterically demanding yet relatively *electron-poor* bisphosphine ancillary ligands should support particularly effective nickel catalysts for such challenging cross-couplings (*vide supra*). Nonetheless, the competent performance of the P(*t*Bu)_2_/P(*o*-tolyl)_2_ ancillary ligand **L5**, although inferior to the CgP/P(*o*-tolyl)_2_ variant **L1** in the test reaction employed (Fig. 3), suggests that ancillary ligand sterics is particularly important in engendering useful catalytic behaviour within the nickel-catalysed C(*sp*^2^)–N cross-couplings under scrutiny herein. The poor performance of both CgPPh^38^ and P(*o*-tolyl)_2_Ph^50^, which did not vary when using Ni:L=1:1 or 1:2, confirms the benefit of bisphosphine ligation by **L1**. Finally, the observation that [Pd(cinnamyl)Cl]_2_/**L1** mixtures afforded no conversion of **1a** under analogous conditions underscores that the design of **L1** (PAd-DalPhos) is particularly well-matched to the properties of nickel, rather than those of palladium, in this reaction setting.

**Figure 4:**
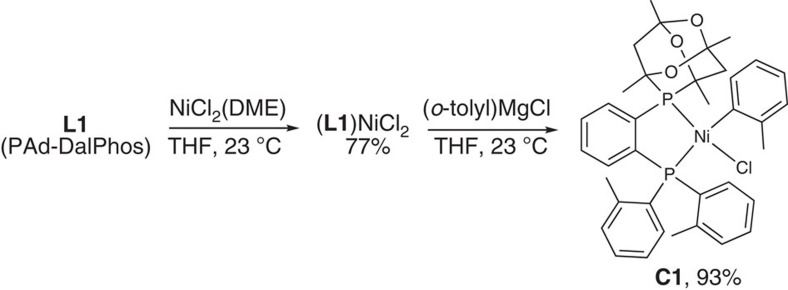
Synthesis of the L1-derived nickel complexes (L1)NiCl_2_ and C1. Isolated yields are provided; DME, 1,2-dimethoxyethane.

**Figure 5:**
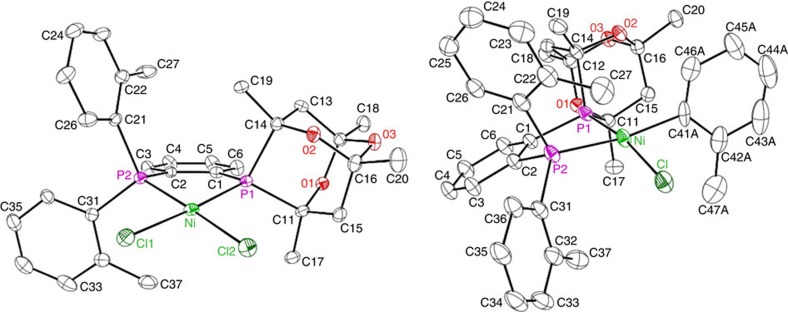
Single-crystal X-ray structures of (L1)NiCl_2_ and C1 depicted with hydrogen atoms omitted for clarity. Selected interatomic distances (Å) and angles (°): for (**L1**)NiCl_2_ Ni-P1 2.1903(12), Ni-P2 2.1691(13), Ni-Cl1 2.2195(13), Ni-Cl2 2.1865(13), P1-Ni-P2 86.74(5), P1-Ni-Cl2 94.03(5), P2-Ni-Cl1 88.88(5), Cl1-Ni-Cl2 90.37(5); for **C1**: Ni-P1 2.1766(8), Ni-P2 2.2263(8), Ni-Cl 2.1916(8), Ni-C(aryl) 1.971(3), P1-Ni-P2 86.52(3), P1-Ni-C(aryl) 95.79(10), P2-Ni-C1 92.72(3), Cl-Ni-C(aryl) 87.53(10).

We subsequently sought to circumvent the use of costly and air/temperature-sensitive Ni(COD)_2_. Notwithstanding the reactivity benefits associated with the use of nitriles as co-ligands/additives^27^,^28^,^30^, and the utility of precious metal/nickel photoredox dual catalysis^51^, in an effort to facilitate end-user uptake we intentionally targeted operationally simple, air-stable nickel pre-catalysts, without recourse to the aforementioned experimental modifications. We thus turned our attention to the synthesis of (**L1**)Ni(*o*-tolyl)Cl (**C1**), which could be reduced to a requisite (**L1**)Ni(0) species under the amination conditions employed without the formation of inhibiting by-products^30^,^52^. Combination of **L1** with NiCl_2_(DME) afforded (**L1**)NiCl_2_ in 77% isolated yield, and subsequent treatment with (*o*-tolyl)MgCl afforded (**L1**)Ni(*o*-tolyl)Cl (**C1**) in 93% isolated yield (Fig. 4). Each complex was obtained as an analytically pure solid, and was characterized by use of spectroscopic and crystallographic techniques (Fig. 5; solution and refinement information are provided in Supplementary Tables 1 and 2). The κ^2^-*P*,*P*-bidentate nature of **L1** is evident in the solid-state structures of both complexes, which are best described as exhibiting a distorted square planar geometry at nickel (Σ_angles at Ni_∼360°). The catalytic performance of **C1** was found to be identical to that of Ni(COD)_2_/**L1** mixtures in the test reaction featured in Fig. 3, with no loss of performance observed following storage of **L1** or **C1** in air for an extended period (months). Moreover, the performance of **C1** was found to be vastly superior to that of Ni(COD)_2_/**L1** under more challenging reaction conditions (that is, room temperature, lower catalyst loadings), in keeping with catalyst inhibition by COD^53^.

### Ammonia monoarylation substrate scope using C1

**Figure 6:**
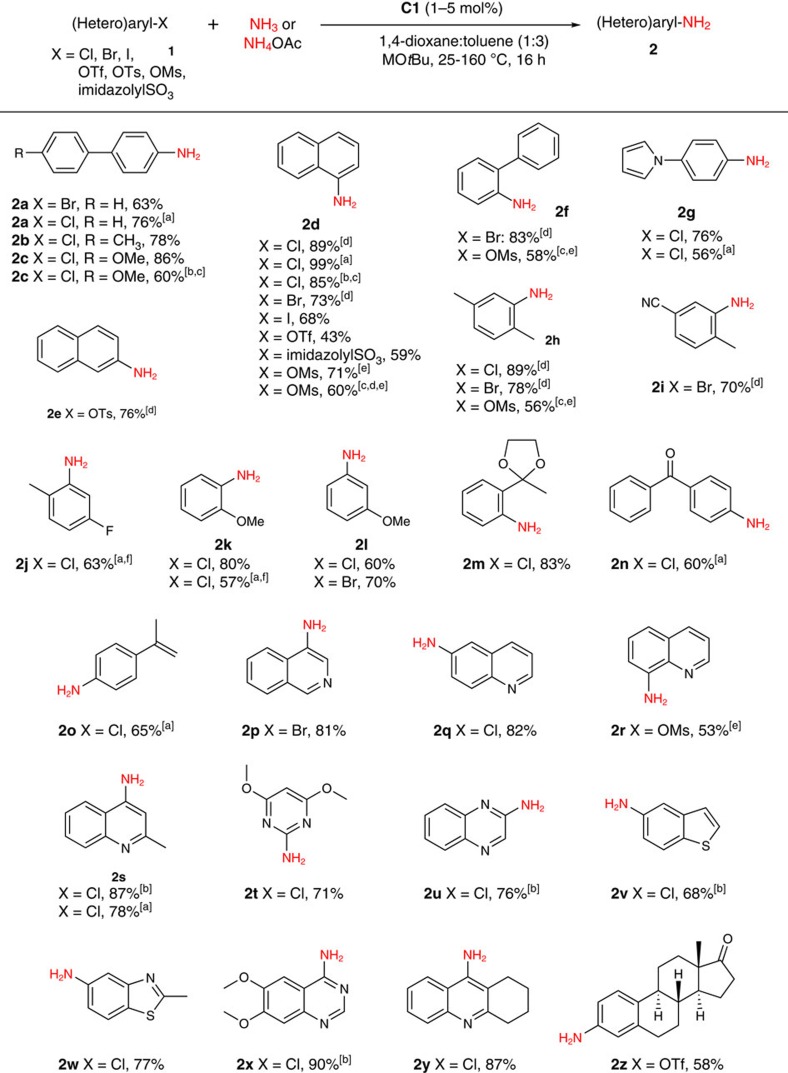
Scope of ammonia monoarylation using C1. Unless stated otherwise, reactions were conducted employing **C1** (1–5 mol%), MO*t*Bu (M=Li or Na; 1.5–2.0 equiv), NH_3_ (from 0.5 M solutions in 1,4-dioxane; 3–8.3 equiv), in toluene for 16 h (unoptimized), with yields of isolated products reported; throughout, see the Supplementary Information for complete experimental details. [a] 110–160 °C for 5–30 min under microwave conditions using NH_4_OAc (5 equiv) and NaO*t*Bu (6.5 equiv) in CPME. [b] Conducted using gaseous ammonia (114 psi initial pressure). [c] Yield on the basis of ^1^H NMR data relative to ferrocene as an internal standard. [d] 25 °C. [e] K_3_PO_4_ (6 equiv) used as base at 110 °C without toluene co-solvent. [f] Isolated as the *N*-tosylated derivative.

The scope of reactivity exhibited by **C1** in the monoarylation of ammonia (Fig. 6), both in terms of the breadth of electrophilic partner (for example, chlorides, bromides, iodides, mesylates, tosylates, triflates and imidazolylsulfonates^54^) and the varied reaction conditions (for example, room temperature; microwave conditions; use of gaseous ammonia), exceeds that demonstrated previously for any catalyst (that is, palladium, copper, nickel or other). In keeping with the preliminary ligand screening experiments (Fig. 3), 4-aminobiphenyl monoarylation products (**2a-c**) were isolated in synthetically useful yields, as were 1- or 2-naphthylamines (**2d,e**) derived from an unprecedentedly wide array of 1- or 2-(pseudo)halonaphthalenes. Electrophiles featuring or lacking *ortho*-substitution were also accommodated, including variants incorporating pyrrole, cyano, fluoro, methoxy, dioxolane, ketone and alkene functionalities (**2f-o**). Given the importance of biologically active (hetero)anilines in pharmaceutical chemistry, we turned our attention to **C1**-catalysed ammonia monoarylations employing (hetero)aryl (pseudo)halide electrophiles. We were pleased to find that quinoline, isoquinoline, quinaldine, pyrimidine, quinoxaline, quinazoline, benzothiophene and benzothiazole core structures each proved compatible in this chemistry (**2p-y**). Notably, the quinazoline **2x** represents the core structure found within a series of commercialized drugs, including doxazosin which is employed for the treatment of symptoms associated with benign prostatic hyperplasia. Moreover, the quinoline **2y** (tacrine) has been used as a cholinesterase inhibitor for the treatment of Alzheimer's disease, whereas 3-aminoestrone (**2z**) has been identified as a key synthon for the construction of non-natural C-18 steroids for use in the treatment of prostate and breast cancers^55^.

Other distinguishing facets of the above-mentioned reactivity warrant further commentary. Whereas room temperature C(*sp*^2^)–N cross-couplings are attractive in terms of operational simplicity and reduced energy footprint, ammonia monoarylation has proven difficult; only a small number of such transformations have been achieved using palladium catalysis^44^, and none involving nickel catalysis. The remarkable ability of **C1** to catalyse room temperature ammonia monoarylations is demonstrated in entries **2d-f,h,i**, covering chloride, bromide, tosylate and mesylate electrophiles. Furthermore, the ability to conduct such room temperature ammonia monoarylation reactions on gram-scale was confirmed in the reaction of 1-chloronaphthalene with ammonia leading to **2d** (2 mol% **C1**, 2.285 g, 76% isolated yield). Although we did not make an effort to optimize the reaction times, the monoarylation of ammonia using 1-chloronaphthalene was found to be complete (>90% conversion to **2d** on the basis of GC data) after only 15 min when using 5 mol% **C1**, thereby underscoring the highly active nature of **C1** under room temperature conditions. Although no loss in catalytic activity was observed in the monoarylation of ammonia using 1-chloronaphthalene when the solid reaction components including **C1** were handled in air, followed by delivery of the ammonia stock solution on the benchtop within a nitrogen-purged glove-bag, analogous reactions conducted under an atmosphere of air were unsuccessful. The first examples of ammonia monoarylation employing (hetero)aryl mesylates (**2d,f,h,r**) involving any catalyst system are also reported. There is significant interest in the development of cross-couplings involving such electrophiles, given their low cost and greater atom economy relative to (hetero)aryl tosylates and triflates, and in the context that the methanesulfonic acid generated upon workup is naturally occurring and can undergo biodegradation under conventional waste-water processing^56^,^57^. Finally, scalability issues in ammonia monoarylation would suggest the use of gaseous ammonia, whereas the application of ammonium salts^28^ offers operational simplicity in bench-scale syntheses. The ability of **C1** to function effectively both when using high pressures of gaseous ammonia (**2c,d,s,u,v,x**), and alternatively ammonium acetate under microwave reaction conditions at elevated reaction temperatures (**2a,d,g,j,k,n,o,s**), is unique among all previously reported catalyst systems for ammonia monoarylation.

### Primary alkylamine monoarylation substrate scope using C1

**Figure 7:**
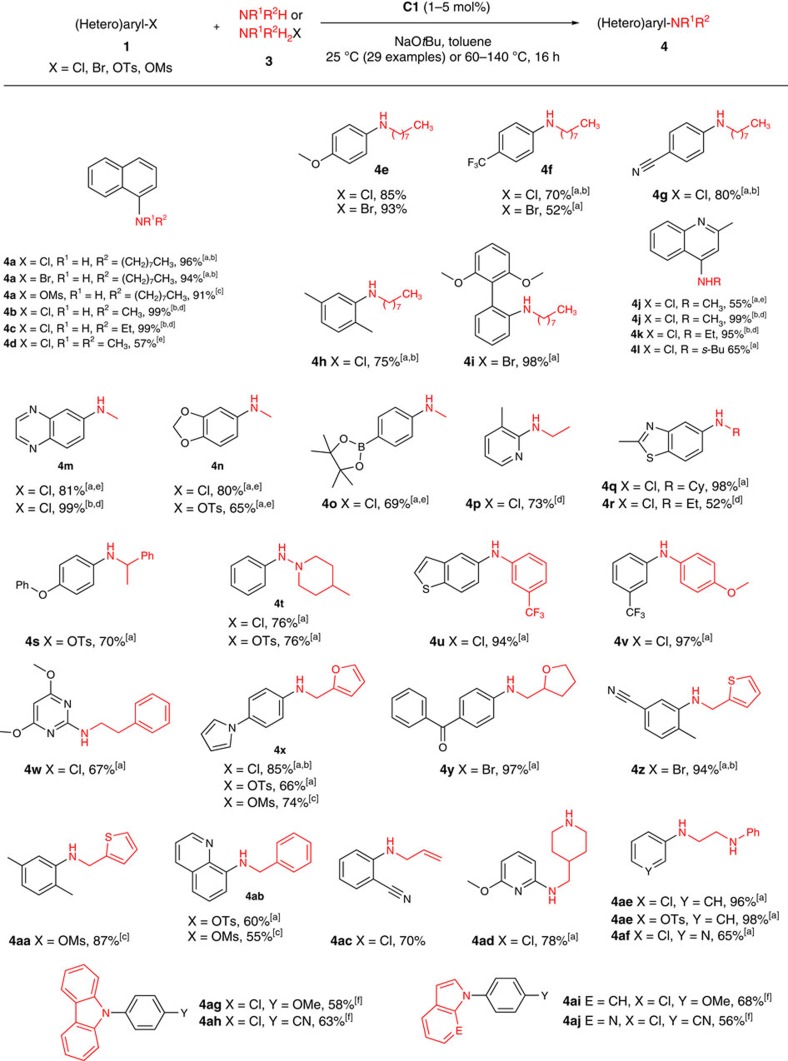
Scope of amine monoarylation using C1. Unless stated otherwise, reactions were conducted employing **C1** (1–5 mol%), NaO*t*Bu (1.5 equiv), amine (1.1 equiv), in toluene for 16 h (unoptimized), with yields of isolated products reported; throughout, see the Supplementary Information for complete experimental details. [a] 25 °C. [b] 1 mol% **C1**. [c] K_3_PO_4_ (6 equiv) used as base, in CPME at 110 °C. [d] 110–140 °C for 5–30 min under microwave conditions using NaO*t*Bu (6.5 equiv) and MeNH_3_Cl or EtNH_3_Cl (5 equiv) in CPME. [e] 3–7 equiv amine. [f] 10 mol% **C1**, NaO*t*Bu (3 equiv), amine (1 equiv) in 1,4-dioxane at 110 °C.

Primary alkylamines represent an important yet relatively challenging class of substrates in C(*sp*^2^)–N cross-coupling chemistry^6^. As in the case of ammonia monoarylation, palladium complexes featuring bulky electron-rich ancillary ligands are the catalysts of choice for such transformations. The state-of-the-art nickel pre-catalyst for the monoarylation of primary alkylamines is (*rac*-BINAP)Ni(η^2^-NC-Ph) (*rac*-BINAP=racemic 2,2′-bis(diphenylphosphino)-1,1′-binaphthyl) reported by Hartwig and co-workers^27^. However, a number of notable limitations exist, including: the air-sensitive nature of this nickel(0) pre-catalyst; the lack of electrophile scope beyond (hetero)aryl chlorides; the use of 50% excess of the alkylamine versus the electrophile; and the absence of demonstrated room temperature catalysis. Use of the air-stable pre-catalyst **C1** addressed these shortcomings, with the observed reaction scope (Fig. 7) rivaling that achieved by use of the best palladium catalysts. A diversity of electron-rich and electron-poor (hetero)aryl (pseudo)halides were successfully cross-coupled with linear and branched primary alkylamines featuring in some cases heterocyclic addenda, as well as a hydrazine derivative and primary anilines. Remarkably, 29 of the reported entries proceeded efficiently at room temperature, covering chloride, bromide and tosylate electrophiles. The gram-scale cross-coupling of 1-chloronaphthalene and octylamine at room temperature leading to **4a** was also achieved (3 mol% **C1**, 2.703 g, 90% isolated yield). Included in the substrate scope are the first examples of nickel-catalysed primary alkylamine monoarylation employing (hetero)aryl mesylates (**4a,x,aa,ab**); related palladium-catalysed transformations are limited to a single report^58^. In an effort to evaluate whether C(*sp*^2^)–N cross-couplings employing **C1** and chiral amines could be conducted without racemization^59^, the room-temperature cross-coupling of racemic and separately enantiopure α-methylbenzylamine leading to **4s** was conducted; ^1^H NMR analysis, employing a europium chiral shift reagent, of the **4s** product thus formed indicated the absence of racemization when using enantiopure α-methylbenzylamine. Reactions involving small nucleophilic reagents such as methylamine and ethylamine employing commercial stock solutions of these amines, or alternatively alkylammonium salts under microwave conditions, proceeded successfully. The formation of the pinacolborane derivative **4o** demonstrated the feasibility of conducting **C1**-catalysed C(*sp*^2^)–N cross-couplings in the presence of a potentially reactive pinacolborane moiety, which may be exploited subsequently in an orthogonal cross-coupling step. The preferred arylation of a primary alkylamine fragment in the presence of contending secondary amine groups by **C1** was demonstrated in the chemoselective formation of **4ad-af**. These transformations are in keeping with our observation that secondary dialkylamines (for example, morpholine) are not suitable substrates for **C1** under the conditions outlined in Fig. 7, despite our successful arylation of dimethylamine leading to **4d**. Finally, we sought to evaluate the ability of **C1** to catalyse the *N*-arylation of carbazole, indole and 7-azaindole—a class of nickel-catalysed transformations that was reported for the first time in 2015 by Belderrain, Nicasio and co-workers employing IPr^60^. These nucleophiles were successfully *N*-arylated employing electron-rich and electron-poor electrophiles, affording **4ag-aj**. The successful C(*sp*^2^)–N cross-coupling of ammonia, primary alkylamines and indoles by use of **C1** is particularly remarkable, given that these compounds span more than 20 orders of magnitude in terms of NH acidity.

## Discussion

The pre-catalyst **C1**, featuring the sterically demanding and electron-poor bisphosphine ancillary ligand **L1** (PAd-DalPhos), enables unprecedented nickel-catalysed C(*sp*^2^)–N cross-coupling chemistry under mild conditions and without the need for a precious metal co-catalyst. The utility of **C1** is showcased in the first reports of room-temperature nickel-catalysed reactions involving primary alkylamines and ammonia, including reactions involving (hetero)aryl mesylates, for which no capable catalyst system of any type had been described previously. The unusual versatility of this pre-catalyst is further demonstrated in the cross-coupling of (hetero)indoles.

The ‘re-purposing' of ancillary ligands that function well with palladium has in some cases proven useful in the development of nickel catalysts for use in cross-coupling chemistry. A complementary approach involves the design of new ancillary ligands whose structural features take into account the specific reactivity properties of nickel, as compared with those of palladium. Using this latter approach, our results presented herein confirm that sterically hindered and relatively electron-poor bisphosphines, as exemplified by **L1**, represent promising ancillary ligand candidates in the quest to address outstanding challenges in nickel-catalysed C(*sp*^2^)–N cross-coupling chemistry and beyond. Unlike palladium chemistry, the applicability of diverse ancillary ligand strategies for use in enabling varied nickel-catalysed cross-couplings is crucial not only in terms of promoting elementary catalytic steps, but also as a means of favouring desired oxidation states of nickel, given the established viability of both Ni(0)/Ni(II) and Ni(I)/Ni(III) catalytic cycles. In this regard, we envision that the ancillary ligand design strategies employed herein will contribute towards the development of high-performing nickel catalysts for use in a range of synthetically important cross-coupling applications, so as to enable more sustainable chemical practices that circumvent the use of precious metals such as palladium.

## Methods

### General considerations

Unless otherwise stated, all reactions were setup inside a nitrogen-filled inert atmosphere glovebox, and were worked up in air using benchtop procedures. Toluene was deoxygenated by sparging with nitrogen followed by passage through a double column solvent purification system packed with alumina and copper-Q5 reactant, and storage over activated 4 Å molecular sieves. Cyclopentyl methyl ether (CPME) was degassed by use of three repeated freeze–pump–thaw cycles and was stored over activated 4 Å molecular sieves. 1,4-Dioxane used in General Procedure I (GPI) was dried over Na/benzophenone followed by distillation under a nitrogen atmosphere. Otherwise, all reagents, solvents and materials were used as received from commercial sources. Column chromatography was carried out using Silicycle SiliaFlash 60 silica (particle size 40–63 μm; 230–400 mesh) or using neutral alumina (150 mesh; Brockmann-III; activated), as indicated. Preparatory thin-layer chromatography (TLC) was carried out on Silicycle plates (TLG-R1001B-341, silica glass-backed TLC Extra Hard Layer, 60 Å, thickness 1 mm, indicator F-254). Unless stated, NMR spectra were recorded at 300 K in CDCl_3_ with chemical shifts expressed in parts per million (p.p.m.) using the residual CHCl_3_ solvent signal (^1^H, 7.26 p.p.m.; ^13^C, 71.4 p.p.m.) as an internal reference or H_3_PO_4_ as an external reference (^31^P, 0.00 p.p.m.). Splitting patterns are indicated as follows: br, broad; s, singlet; d, doublet; m, multiplet, with all coupling constants (*J*) reported in Hertz (Hz). In some cases, fewer than expected independent ^13^C NMR resonances were observed despite prolonged acquisition times. For NMR analysis of the compounds in this article, see the Supplementary Methods and Supplementary Figs 1–142. Mass spectra were obtained using ion trap electrospray ionization (ESI) instruments operating in positive mode, and gas chromatography (GC) data were obtained on an instrument equipped with a SGE BP-5 column (30 m, 0.25 mm i.d.).

### Synthesis of A

To a glass screw-capped vial containing a magnetic stir bar was added 2-bromoiodobenzene (0.73 ml, 5.7 mmol, 1.05 equiv), toluene (9.0 ml), Pd(PPh_3_)_4_ (0.330 g, 0.285 mmol), K_2_CO_3_ (1.571 g, 11.4 mmol, 2.0 equiv) and 1,3,5,7-tetramethyl-2,4,8-trioxaphosphaadamantane (1.14 g, 5.3 mmol). The vial was sealed with a poly(tetrafluoroethylene) (PTFE)-lined cap and was removed from the glovebox. The vial was placed in an oil bath set to 110 °C and magnetic stirring was initiated. After 48 h (unoptimized), the reaction mixture was cooled, diluted with CH_2_Cl_2_ (50 ml) and washed with distilled water (3 × 50 ml). The organic layer was dried over anhydrous Na_2_SO_4_, filtered and the collected eluent solution was concentrated under reduced pressure by use of a rotary evaporator. The resulting yellow oil was filtered through an alumina plug (ca 50 g) eluting with 90% hexanes/CH_2_Cl_2_; the solvent was then removed from the collected eluent under reduced pressure by use of a rotary evaporator. The resulting yellow solid was purified by flash chromatography over silica, eluting with 10% ethyl acetate/hexanes to afford **A** as a white solid (1.69 g, 86% yield). ^1^H NMR: (CDCl_3_, 500 MHz) 8.29 (d, *J*=7.7, 1H), 7.66–7.64 (m, 1H), 7.37 (apparent t, *J*=7.5 Hz, 1H), 7.25 (apparent t, *J*=7.6 Hz, 1H), 2.14 (m, 1H), 2.02-1.89 (m, 2H), 1.55–1.44 (m, 13H). ^13^C{^1^H} NMR: (CDCl_3_, 125.8 MHz) 135.3 (d, *J*=22.6 Hz), 135.2, 133.8 (d, *J*=2.5 Hz), 133.2 (d, *J*=37.7 Hz), 131.0, 127.5, 97.0, 96.2, 74.5 (d, *J*=10.1 Hz), 73.9 (d, *J*=25.2 Hz), 45.8 (d, *J*=20.1 Hz), 36.5, 28.7 (d, *J*=18.9 Hz), 28.2, 27.9, 26.7 (d, *J*=11.3 Hz). ^31^P{^1^H} NMR: (CDCl_3_, 202.5 MHz) −29.6. High resolution mass spectrometry-electrospray ionization (HRMS-ESI) (*m*/*z*): calculated for C_16_H_20_^79^BrNaO_3_P [M+ Na]: 393.0226; found: 393.0214.

### General procedure for the synthesis of L1–L4

Compound **A** and diethyl ether (∼0.3 M in **A**) were added to a glass screw-capped vial containing a magnetic stir bar. The vial was sealed with a cap featuring a PTFE septum. The solution was then cooled to −33 °C and magnetic stirring was initiated, followed by dropwise addition of *n*-butyllithium (1.5 equiv, 2.5 M in hexanes) via syringe. The resulting mixture was left to stir for 30 min while warming to ambient temperature. At this point, the appropriate chlorophosphine (R_2_ClP, R=*o*-tol, Ph, *i*Pr, Cy; 1.2 equiv) was added dropwise via syringe with continued stirring. The resulting mixture was left to stir for 48 h (unoptimized) at ambient temperature, after which the crude reaction mixture was opened to air on the benchtop and was filtered through a short Celite plug; the collected eluent was concentrated by use of a rotary evaporator. The residue was adsorbed onto silica (ca 1 g) and was then concentrated to dryness by use of a rotary evaporator. The so-formed silica dry pack was added to a silica plug (ca 50 g), and 10% EtOAc/hexanes (ca 300 ml) was passed through the plug. The collected eluent was then concentrated to dryness by use of a rotary evaporator, was washed with cold pentane (3 × 1.5 ml) and was then dried *in vacuo* to afford the desired bisphosphine as a white to off-white solid (R=*o*-tolyl, **L1**: 80%; R=Ph, **L2**: 63%; R=*i*Pr, **L3**: 68%; R=Cy, **L4**: 53%). Please note that the cyclohexyl variant **L4** was purified by flash chromatography over silica (ca 50 g) eluting with 10% EtOAc/hexanes. Note that the NMR spectral assignments for **L1**–**L4** and **C1** in some cases was rendered complex by: the *C*_1_-symmetric nature of these species owing to the chiral (racemic) phosphaadamantane group; second-order coupling; dynamic behaviour (as evidenced in the temperature-dependent ^31^P{^1^H} NMR spectra of **L1**) and possibly in the case of **C1** dynamic equilibria involving rotamers and/or between tetrahedral and square planar species.

### Data for L1 PAd-DalPhos

^1^H NMR (CDCl_3_, 300 MHz): 8.32 (m, 1H), 7.39 (m, 1H), 7.29–7.21 (m, 5H), 7.11–7.04 (m, 2H), 6.89–6.87 (m, 1H), 6.79 (dd, *J*=7.2, 3.1 Hz, 1H), 6.64 (m, 1H), 2.42 (s, 3H), 2.36 (s, 3H), 2.14–1.79 (m, 3H), 1.57–1.21 (m, 13H). ^13^C{^1^H} NMR (CDCl_3_, 125.8 MHz): 142.7–142.2 (m), 134.2, 133.5, 130.3-130.0 (m), 128.8-128.6 (m), 126.4, 125.8, 97.2, 96.3, 74.5–74.2 (m), 46.1 (d, *J*=18.9 Hz), 36.6, 28.4–28.0 (m), 26.3 (d, *J*=11.3 Hz), 21.7, 21.5. ^31^P{^1^H} NMR: (CDCl_3_, 202.5 MHz, 298 K) −24.1 (broad m), −37.7 (d, *J*=166 Hz). ^31^P{^1^H} NMR: (CDCl_3_, 121.5 MHz, 223 K) −23.8 (d, *J*=160 Hz, major species), −30.2 to −33.0 (broad m, minor species), −38.8 (d, *J*=177 Hz, minor species), −39.4 (d, *J*=160 Hz, major species). HRMS-ESI (*m*/*z*): calculated for C_30_H_34_NaO_3_P_2_ [M+Na]: 527.1881; found: 527.1875. Analysis calculated for C_30_H_34_O_3_P_2_: C, 71.42; H, 6.79. Found: C, 71.12; H, 6.84.

### Data for L2

^1^H NMR: (CDCl_3_, 500 MHz): 8.36–8.33 (m, 1H), 7.40–7.30 (m, 10H), 7.23–7.19 (m, 2H), 7.02–6.99 (m, 1H), 2.12–2.07 (m, 2H), 1.94 (m, 1H), 1.56–1.53 (m, 1H), 1.49 (s, 3H), 1.43–1.40 (m, 6H), 1.33 (d, *J*=12.4 Hz, 3H). ^13^C{^1^H} NMR (CDCl_3_, 125.8 MHz): 147.5–147.1 (m), 140.5–140.0 (m), 137.9–137.5 (m), 134.6 (m), 134.2 (m), 133.5 (m), 129.9, 128.9–128.6 (m), 128.4 (two signals), 97.1, 96.2, 74.6, 74.5 (m), 46.1 (d, *J*=18.9 Hz), 36.6, 28.4-28.0 (m), 26.5 (d, *J*=11.3 Hz). ^31^P{^1^H} NMR (CDCl_3_, 202.5 MHz): −12.5 (d, *J*=168 Hz, 1P), −37.6 (d, *J*=168 Hz, 1P). HRMS-ESI (*m*/*z*): calculated for C_30_H_34_NaO_3_P_2_ [M+Na]: 499.1562; found: 499.1562.

### Data for L3

^1^H NMR (CDCl_3_, 300 MHz): 8.36–8.31 (m, 1H), 7.59 (m, 1H), 7.40–7.36 (m, 2H), 2.42–2.36 (m, 1H), 2.20–1.87 (m, 4H), 1.46–1.36 (m, 13H), 1.24 (dd, *J*=15.4, 6.8 Hz, 3H), 1.14 (m, 3H), 1.01–0.90 (m, 6H). ^13^C{^1^H} NMR (CDCl_3_, 125.8 MHz): 134.1, 133.5 (m), 132.4 (m), 129.1 (two signals), 128.7 (m), 97.1, 96.2, 74.8. 74.7, 74.3–74.0 (m), 46.3 (d, *J*=20.1 Hz), 36.4, 28.4–28.0 (m), 26.7 (d, *J*=11.3 Hz), 22.9 (m), 20.7–19.8 (m), 18.0. ^31^P{^1^H} NMR (CDCl_3_, 202.5 MHz): −38.5 to −39.2 (m). HRMS-ESI (*m*/*z*): calculated for C_30_H_34_ NaO_3_P_2_ [M+Na]: 431.1875; found: 431.1867.

### Data for L4

^1^H NMR (CDCl_3_, 500 MHz): 8.35–8.33 (m, 1H), 7.68 (broad s, 1H), 7.42–7.40 (m, 2H), 2.19–1.09 (m, 38H). ^13^C{^1^H} NMR (CDCl_3_, 125.8 MHz): 134.0, 133.1, 128.8, 128.5, 97.1, 96.2, 74.7 (two signals), 74.2 (m), 46.4 (d, *J*=18.9 Hz), 37.0–36.4 (m), 33.0–32.8 (m), 31.1–30.0 (m), 28.4–26.6 (m). ^31^P{^1^H} NMR (CDCl_3_, 202.5 MHz): −14.0 (broad m), −39.6 (broad m). HRMS-ESI (*m*/*z*): calculated for C_28_H_43_O_3_P_2_ [M+H]: 489.2687; found: 489.2682.

### Synthesis of L5

To a glass screw-capped vial containing a magnetic stir bar was added (2-bromophenyl)-di-*tert*-butylphosphine^61^ (**B**, 145.1 mg, 0.482 mmol, 1.0 equiv) and diethyl ether (1.5 ml). The vial was sealed with a cap featuring a PTFE septum. The solution was then cooled to −33 °C and magnetic stirring was initiated, followed by dropwise addition of *n*-butyllithium (0.29 ml, 2.5 M in hexanes, 1.5 equiv) via syringe. The resulting mixture was left to stir for 30 min while warming to ambient temperature. At this point, chlorodi(*o*-tolyl)phosphine (126 mg, 0.506 mmol, 1.05 equiv) was dissolved in diethyl ether (1.0 ml) and was added dropwise via syringe to the reaction vial with continued stirring. The resulting mixture was left to stir for 48 h (unoptimized) at ambient temperature, after which the crude reaction was opened to air on the benchtop and was filtered through a short Celite plug; the collected eluent was concentrated by use of a rotary evaporator. The residue was adsorbed onto silica (ca 1 g) and was then concentrated to dryness by use of a rotary evaporator. The so-formed silica dry pack was added to a silica plug (ca 50 g), and 1% EtOAc/hexanes (ca 200 ml) was passed through the plug. The collected eluent was then concentrated to dryness by use of a rotary evaporator, was washed with cold pentane (3 × 1.5 ml) and was then dried *in vacuo* to afford **L5** as an off-yellow solid (0.130 g, 62% yield). ^1^H NMR (300.1 MHz, CDCl_3_): *δ*=7.78 (br s, 1H), 7.34–7.19 (m, 6H), 7.07–6.98 (m, 3H), 6.86–6.82 (m, 2H), 2.47 (br s, 6H), 1.11–1.07 (br d, *J*=10.5 Hz, 18H); ^13^C{ ^1^H} NMR (125.8 MHz, CDCl_3_): *δ*=143.1 (m), 135.4, 135.1, 133.2, 130.1, 129.2, 128.4, 127.4, 126.0, 33.3 (m), 30.7 (d, *J*=11.3 Hz), 21.8, 21.7; ^31^P{^1^H} NMR (121.5 MHz, CDCl_3_): *δ*=20.7 (d, *J*=167 Hz, 1P), −27.5 (d, *J*=166 Hz, 1P). HRMS-ESI (*m*/*z*): calculated for C_28_H_37_P_2_ [M+H]: 435.2292; found: 435.2365.

### Synthesis of (L1)NiCl_2_

In a dinitrogen filled glovebox, a 100-ml oven-dried round bottom flask containing a magnetic stir bar was charged with NiCl_2_(DME) (1.78 g, 8.10 mmol) and **L1** (PAd-DalPhos; 4.54 g, 9.00 mmol, 1.1 equiv). The solid mixture was dissolved in ca 90 ml of tetrahydrofuran (THF) and the resulting solution was stirred magnetically at room temperature for 1 h. The crude reaction mixture was poured directly onto a glass frit and was washed with pentane (5 × 30 ml). The remaining solid on the frit was dissolved by passing CH_2_Cl_2_ through the frit (ca 50 ml), followed by collection of the eluent. The solvent was removed *in vacuo* affording the desired product as a dark purple paramagnetic solid (3.93 g, 77%). Anal. calculated for C_30_H_34_Cl_2_NiO_3_P_2_ C, 56.82; H, 5.40. Found: C, 56.72; H, 5.65. A single crystal suitable for X-ray diffraction analysis was prepared by slow evaporation of pentane into a solution of CH_2_Cl_2_ at room temperature.

### Synthesis of C1

(**L1**)NiCl_2_ (3.90 g, 6.15 mmol) and THF (62 ml) were added to an oven-dried 100 ml round-bottom flask containing a magnetic stir bar. Magnetic stirring was initiated and *ortho*-tolylmagnesium chloride was then added dropwise (7.40 ml, 7.40 mmol, 1.2 equiv, 1.0 M in THF) to the heterogeneous mixture, resulting in an immediate colour change from red to orange. The reaction mixture was allowed to stir at room temperature for 2 h. The reaction mixture was subsequently treated with MeOH (5 ml) in air, and then was reduced to dryness *in vacuo*. The residue was treated with cold MeOH (0 °C, 15 ml), and the crude reaction mixture was then filtered through a glass frit, affording a retained orange solid that was washed with additional cold MeOH (0 °C, 3 × 10 ml), followed by pentane (3 × 50 ml). The orange solid on the frit was then dissolved via addition of CH_2_Cl_2_ (50 ml). Collection of the eluent followed by removal solvent afforded (**L1**)Ni(o-tolyl)Cl, **C1**, as an orange solid (3.95 g, 93% yield). The existence of a major and minor disastereomers (ca 2:1) in solution is suggested on the basis of ^31^P{^1^H} NMR data. ^1^H NMR (CDCl_3_, 500 MHz): 8.74 (m, 1H), 7.59–7.09 (m, 10H), 6.86–6.67 (m, 5H), 3.33–2.59 (m, 9H), 1.98–1.93 (m, 1H), 1.59–1.53 (m, 6H), 1.42 (s, 3H), 1.10–0.92 (s, 6H). ^13^C{^1^H} NMR (CDCl_3_, 125.8 MHz): 145.9 (m), 145.8 (m), 143.4–143.2 (m), 136.7–133.1 (m), 132.0–130.9 (m), 129.6–128.6 (m), 126.3–125.8 (m), 124.7 (m), 123.8 (m), 122.7, 97.8–96.2 (m), 40.2–39.6 (m), 28.8–24.2 (m). ^31^P{^1^H} NMR (CDCl_3_, 202.5 MHz): 32.6 (d, *J*=4.3 Hz, minor species), 31.5 (d, *J*=4.6 Hz, major species), 27.6 (d, *J*=4.6 Hz, major species), 26.5 (d, *J*=4.3 Hz, minor species). On the basis of the observed positional disorder associated with the Ni-bound *ortho*-tolyl fragment within the X-ray structure of **C1**, arising from Ni-C(tolyl) bond rotation (80:20 occupancy ratio), we interpret the major and minor species as being rotamers of this type. Anal. calculated for C_37_H_41_ClNiO_3_P_2_ C, 64.42; H, 5.99. Found: C, 64.11; H, 5.84. A single crystal suitable for X-ray diffraction analysis was prepared by slow evaporation of pentane into a solution of CH_2_Cl_2_ at room temperature.

### General catalytic procedure A (GPA)

Unless specified otherwise in the text, **C1** (8.3–20.7 mg, 0.0012–0.030 mmol, 2–5 mol %), aryl (pseudo)halide (0.60 mmol, 1 equiv) and NaO*t*Bu (115.3 mg, 1.20 mmol, 2.0 equiv) were added to a screw-capped vial containing a magnetic stir bar, to which was added toluene (9 ml) and NH_3_ as a 0.5 M solution in 1,4-dioxane (1.8–4.2 mmol, 3–7 equiv, 3.0–8.4 ml). The vial was sealed with a cap containing a PTFE septum, was removed from the glovebox and placed in a temperature-controlled aluminum heating block set at 110 °C, and was allowed to react under the influence of magnetic stirring for 16 h (unoptimized). The vial was then removed from the heating block and was left to cool to ambient temperature, after which the reaction mixture was worked up as indicated.

### General catalytic procedure for heteroaryl halides and ammonia (GPB)

Unless specified otherwise in the text, **C1** (10.4 mg, 0.015 mmol, 3 mol %), aryl (pseudo)halide (0.5 mmol, 1 equiv) and LiO*t*Bu (60.0 mg, 0.75 mmol, 1.5 equiv) were added to a screw-capped vial containing a magnetic stir bar, followed by the addition of toluene (4.2 ml) and NH_3_ as a 0.5 M solution in 1,4-dioxane (3.5 mmol, 7 equiv). The vial was sealed with a cap containing a PTFE septum, was removed from the glovebox and placed in a temperature-controlled aluminum heating block set at 110 °C, and was allowed to react under the influence of magnetic stirring for 16 h (unoptimized). The vial was then removed from the heating block and was left to cool to ambient temperature. The crude reaction mixture was dissolved in ethyl acetate (10 ml) and poured onto brine (10 ml). The layers were separated and the aqueous layer was extracted with ethyl acetate (2 × 10 ml). The organic fractions were combined, dried over Na_2_SO_4_ and concentrated under reduced pressure. The crude residue was purified by use of column chromatography over silica.

### General catalytic procedure for aryl mesylates and ammonia (GPC)

Unless specified otherwise in the text, **C1** (4.14 mg, 0.006 mmol, 5 mol%), K_3_PO_4_ (152.8 mg, 0.72 mmol, 6.0 equiv) and aryl mesylate (0.12 mmol, 1.0 equiv), followed by NH_3_ as a 0.5 M solution in 1,4-dioxane (0.75 mmol, 6.25 equiv) were added to a screw-capped vial containing a magnetic stir bar. The vial was sealed with a cap containing a PTFE septum, was removed from the glovebox and placed in a temperature-controlled aluminum heating block set at 110 °C, and was allowed to react under the influence of magnetic stirring for 16 h (unoptimized). The vial was then removed from the heating block and was left to cool to ambient temperature. The crude reaction mixture was dissolved in ethyl acetate (10 ml) and poured onto brine (10 ml). The layers were separated and the aqueous layer was extracted with ethyl acetate (2 × 10 ml). The organic fractions were combined, dried over Na_2_SO_4_ and concentrated under reduced pressure. The crude residue was purified by use of column chromatography over alumina or silica as specified.

### General catalytic procedure for microwave reactions with ammonium salts (GPD)

**C1** (6.9–34.5 mg, 0.01–0.05 mmol, 1–5 mol%), ammonium salt (5 equiv, 5 mmol), NaO*t*Bu (6.5 equiv, 6.5 mmol) and aryl (pseudo)halide (1 mmol) were weighed into an oven-dried 20 ml microwave vial equipped with a magnetic stir bar. CPME (10 ml) was then added to the vial, the vial was sealed with an aluminum crimp cap featuring a PTFE/silicone septum, and was removed from the glovebox. The vial was then heated to the specified temperature in a Biotage Initiator^+^ microwave reactor for the specified time, using fixed-hold time. The vial was then removed from the microwave reactor and was left to cool to ambient temperature, at which point the reaction mixture was taken up in CH_2_Cl_2_ (ca 50 ml) and was washed with distilled water (3 × 50 ml). The organic layer was dried over Na_2_SO_4_ and concentrated under reduced pressure. The crude residue was purified by use of column chromatography using a Biotage Isolera One automated column using a EtOAc:hexanes gradient on a 25-g Biotage Snap cartridge. Notably, although NH_4_OAc proved effective as an ammonia source in these reactions; the use of NH_4_Cl, (NH_4_)_2_SO_4_, LiNH_2_ or LiN(TMS)_2_ afforded negligible conversion to product on the basis of gas chromatographic analysis.

### General catalytic procedure for reactions using ammonia gas (GPE)

Unless specified otherwise in the text, a vial (1 dram, 3.696 ml) containing a magnetic stir bar was charged with **C1** (0.018 mmol, 5 mol%), LiO*t*Bu (43.2 mg, 0.54 mmol, 1.5 equiv), toluene (1.0 ml) and aryl (pseudo)halide (0.36 mmol, 1.0 equiv). The resulting solution was stirred briefly and then was sealed with a cap containing a PTFE septum; the septum was then punctured with a 26G1/2 PrecisionGlide needle and the needle was not removed until the final workup. The reaction vial was placed in a high-pressure reaction chamber purchased from the Parr Instrument Company (type 316 stainless steel, equipped with a thermocouple immersed in oil to allow for accurate external temperature monitoring within the reaction chamber proximal to the placement of the reaction vial), and the reaction chamber was sealed under nitrogen within the glovebox. The reaction chamber was removed from the glovebox, and was placed in an oil bath at room temperature that was mounted on top of a hot-plate/magnetic stirrer. The reaction chamber was fitted with a braided and PTFE-lined stainless steel hose designed for use with corrosive gases that was connected to a tank of anhydrous ammonia gas. Magnetic stirring was initiated and the reaction chamber was purged with ammonia for ∼5 min, after which time the reaction chamber was pressurized with ammonia (114 psi maintained for 30 min at room temperature). The reaction chamber was then sealed, disconnected from the ammonia tank, and was heated at 110 °C for 16 h; pressure was built up to 150 p.s.i. over the course of the reaction. The reaction chamber was allowed to cool to room temperature, after which the contents of the reaction chamber were vented slowly within a fumehood. The products were removed from the pressure reaction chamber, the crude reaction mixture was dissolved in ethyl acetate (10 ml) and poured onto brine (10 ml). The layers were separated and the aqueous layer was extracted with ethyl acetate (2 × 10 ml). The organic fractions were combined, dried over Na_2_SO_4_ and concentrated under reduced pressure. The crude residue was purified by use of column chromatography or over silica.

### General catalytic procedure for aryl halides and primary amines (GPF)

Unless specified otherwise in the text, **C1** (10.3 mg, 0.015 mmol, 3 mol%), NaO*t*Bu (72.3 mg, 0.75 mmol, 1.5 equiv), aryl (pseudo)halide (0.5 mmol, 1.0 equiv), amine (0.55 mmol, 1.1 equiv) and toluene (4.7 ml) were added to a screw-capped vial containing a magnetic stir bar. The vial was sealed with a cap containing a PTFE septum, was removed from the glovebox and placed in a temperature-controlled aluminum heating block set at the specified temperature, and was allowed to react under the influence of magnetic stirring for 16 h (unoptimized). The crude reaction mixture subsequently was dissolved in ethyl acetate (10 ml) and poured onto brine (10 ml). The layers were separated and the aqueous layer was extracted with ethyl acetate (2 × 10 ml). The organic fractions were combined, dried over Na_2_SO_4_ and concentrated under reduced pressure. The crude residue was purified by use of column chromatography over alumina or silica.

### General catalytic procedure using methyl or dimethylamine (GPG)

Unless specified otherwise in the text, **C1** (17.2 mg, 0.025 mmol, 5 mol%), NaO*t*Bu (72.3 mg, 0.75 mmol, 1.5 equiv) and aryl (pseudo)halide (0.5 mmol, 1.0 equiv), followed by the addition of amine (2 M solution in THF, 1.75 ml, 3.5 mmol, 7.0 equiv) were added to a screw-capped vial containing a magnetic stir bar. The vial was sealed with a cap containing a PTFE septum, was removed from the glovebox and placed in a temperature-controlled aluminum heating block set at the specified temperature, and was allowed to react under the influence of magnetic stirring for 16 h (unoptimized). The crude reaction mixture subsequently was dissolved in ethyl acetate (10 ml) and poured onto brine (10 ml). The layers were separated and the aqueous layer was extracted with ethyl acetate (2 × 10 ml). The organic fractions were combined, dried over Na_2_SO_4_ and concentrated under reduced pressure. The crude residue was purified by use of column chromatography over alumina or silica.

### General catalytic procedure for aryl mesylates and primary amines (GPH)

Unless specified otherwise in the text, **C1** (17.2 mg, 0.025 mmol 5 mol%), K_3_PO_4_ (636.8 mg, 3 mmol, 6.0 equiv), aryl mesylate (0.5 mmol, 1.0 equiv), amine (0.55 mmol, 1.1 equiv) and CPME (2.0 ml) were added to a screw-capped vial containing a magnetic stir bar. The vial was sealed with a cap containing a PTFE septum, was removed from the glovebox and placed in a temperature-controlled aluminum heating block set at 110 °C, and was allowed to react under the influence of magnetic stirring for 16 h (unoptimized). The crude reaction mixture subsequently was dissolved in ethyl acetate (10 ml) and poured onto brine (10 ml). The layers were separated and the aqueous layer was extracted with ethyl acetate (2 × 10 ml). The organic fractions were combined, dried over Na_2_SO_4_ and concentrated under reduced pressure. The crude residue was purified by use of column chromatography over alumina or silica.

### General catalytic procedure using carbazole or indole (GPI)

Unless specified otherwise in the text, **C1** (13.8 mg, 0.02 mmol, 10 mol%), NaO*t*Bu (57.6 mg, 0.60 mmol, 3.0 equiv), aryl halide (0.2 mmol, 1.0 equiv), amine (0.2 mmol, 1.0 equiv) and 1,4-dioxane (2.0 ml) were added to a screw-capped vial containing a magnetic stir bar. The vial was sealed with a cap containing a PTFE septum, was removed from the glovebox and placed in a temperature-controlled aluminum heating block set at 110 °C, and was allowed to react under the influence of magnetic stirring for 16 h (unoptimized). The crude reaction mixture was dissolved in ethyl acetate (10 ml) and poured onto brine (10 ml). The layers were separated and the aqueous layer was extracted with ethyl acetate (2 × 10 ml). The organic fractions were combined, dried over Na_2_SO_4_ and concentrated under reduced pressure. The crude residue was purified by use of preparatory silica TLC.

### Room-temperature gram-scale synthesis of 2d

Following **GPA**, **C1** (2 mol%, 0.42 mmol, 290 mg), 1-chloronapthalene (1 equiv, 21 mmol, 2.85 ml), NaOtBu (2 equiv, 42 mmol, 4.03 g), ammonia (0.5M in dioxane, 3 equiv, 63 mmol, 126 ml) and toluene (224 ml) were added to an oven dried 500 ml round-bottom flask equipped with a magnetic stir bar. The reaction flask was sealed with a septum and stirring was initiated at room temperature. After 16 h (unoptimized), the solvent was removed with the aid of a rotary evaporator. The crude residue was dissolved in EtOAc (150 ml), washed with distilled water (2 × 150 ml) and once with brine (150 ml). The organic layer was dried over anhydrous Na_2_SO_4_, filtered and concentrated with the aid of a rotary evaporator. The crude material was purified via automated column chromatography using a EtOAc:hexanes gradient 0–40% EtOAc, giving **2d** (2.285 g, 76% yield).

### Room-temperature gram-scale synthesis of 4a

Following **GPF**, **C1** (3 mol%, 0.35 mmol, 241 mg), 1-chloronaphthalene (1 equiv, 11.7 mmol, 1.60 ml), octylamine (1.1 equiv, 12.9 mmol, 2.13 ml), NaOtBu (1.5 equiv, 17.6 mmol, 1.69 g) and toluene (120 ml) were added to an oven dried 250 round-bottom flask equipped with a magnetic stir bar. The reaction flask was sealed with a septum and stirring was initiated at room temperature. After 16 h (unoptimized), the solvent was removed with the aid of a rotary evaporator. The crude residue was dissolved in EtOAc (150 ml), washed with distilled water (2 × 150 ml) and once with brine (150 ml). The organic layer was dried over anhydrous Na_2_SO_4_, filtered and concentrated with the aid of a rotary evaporator. The crude material was purified via automated column chromatography using a EtOAc:hexanes gradient 0–20% EtOAc, giving **4a** (2.703 g, 90% yield).

### Crystallographic solution and refinement details

Crystallographic data for **C1**·0.5C_5_H_12_·0.5C_4_H_8_O and [(**L1**)NiCl_2_]·0.5CH_2_Cl_2_ were obtained at −100(±2) °C on a Bruker PLATFORM or D8/APEX II CCD diffractometer using graphite-monochromated Mo K*α* (*λ*=0.71073 Å) radiation, employing samples that were mounted in inert oil and transferred to a cold gas stream on the diffractometer. Programmes for diffractometer operation, data collection and data reduction (including SAINT) were supplied by Bruker. Gaussian integration (face-indexed) was employed as the absorption correction method for **C1**, whereas multi-scan (*TWINABS*) was employed for (**L1**)NiCl_2_. The structure of **C1** was solved by use of intrinsic phasing methods, whereas (**L1**)NiCl_2_ was solved by use of a Patterson/structure expansion; both were refined by use of full-matrix least-squares procedures (on *F*^2^) with *R*_1_ based on *F*_o_^2^⩾2σ(*F*_o_^2^) and *wR*_2_ based on *F*_o_^2^⩾–3σ(*F*_o_^2^). In the case of **C1**, attempts to refine peaks of residual electron density as disordered or partial-occupancy solvent pentane and/or tetrahydrofuran oxygen or carbon atoms were unsuccessful. The data were corrected for disordered electron density through use of the SQUEEZE procedure as implemented in *PLATON*. A total solvent-accessible void volume of 1,666 Å^3^ with a total electron count of 323 (consistent with 4 molecules of solvent pentane and 4 molecules of tetrahydrofuran, or 0.5 molecules each of pentane and tetrahydrofuran per formula unit of the Ni complex) was found in the unit cell. Positional disorder that was observed in the Ni-bound *ortho*-tolyl fragment during the solution and refinement of **C1** was modelled in a satisfactory manner (80:20 occupancy ratio); only the larger of these is discussed in the text. In the case of (**L1**)NiCl_2_, the crystal used for data collection was found to display non-merohedral twinning. Both components of the twin were indexed with the programme *CELL_NOW* (Bruker AXS Inc., 2004). The second twin component can be related to the first component by 180° rotation about the [−0.2 −0.25 1] axis in real space and about the [0 0 1] axis in reciprocal space. Integrated intensities for the reflections from the two components were written into a *SHELXL-2014* HKLF 5 reflection file with the data integration programme *SAINT* (version 8.34A), using all reflection data (exactly overlapped, partially overlapped and non-overlapped). The refined value of the twin fraction (*SHELXL-2014* BASF parameter) was 0.4855(13). Furthermore, in the case of (**L1**)NiCl_2_ attempts to refine peaks of residual electron density as disordered or partial-occupancy solvent dichloromethane chlorine or carbon atoms were unsuccessful. The data were corrected for disordered electron density through use of the SQUEEZE procedure as implemented in *PLATON*. A total solvent-accessible void volume of 156.7 Å^3^ with a total electron count of 39 (consistent with one molecule of solvent dichloromethane, or one-half molecule of CH_2_Cl_2_ per formula unit of the Ni complex molecule) was found in the unit cell. Anisotropic displacement parameters were employed for all the non-hydrogen atoms. In all cases, non-hydrogen atoms are represented by Gaussian ellipsoids at the 30% probability level.

## Additional information

**Accession codes:** The X-ray crystallographic coordinates for structures reported in this study have been deposited at the Cambridge Crystallographic Data Centre (CCDC), under deposition numbers 1403891-1403892. These data can be obtained free of charge from the Cambridge Crystallographic Data Centre via www.ccdc.cam.ac.uk/data_request/cif.

**How to cite this article:** Lavoie, C. M. *et al*. Challenging nickel-catalysed amine arylations enabled by tailored ancillary ligand design. *Nat. Commun.* 7:11073 doi: 10.1038/ncomms11073 (2016).

## Supplementary Material

Supplementary Information Supplementary Figures 1-142, Supplementary Tables 1-2, Supplementary Methods and Supplementary References

## Figures and Tables

**Figure 1:**
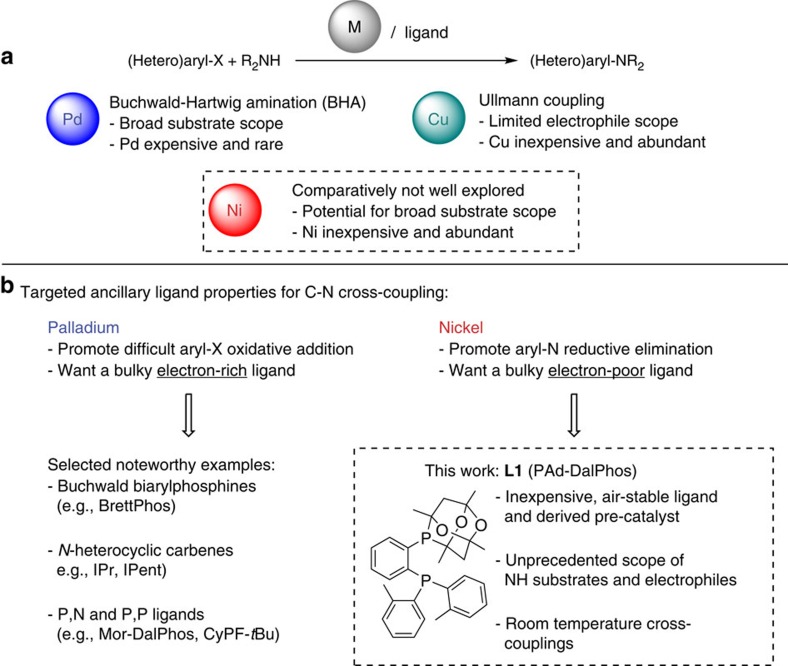
Approaches to metal-catalysed C–N cross-coupling. (**a**) Generic equation for Buchwald–Hartwig amination and Ullmann C–N coupling to form a (hetero)aniline. (**b**) Ancillary ligand design strategies for palladium and nickel, along with the new ancillary ligand **L1** (PAd-DalPhos).
