# Supplementary material for: Challenging nickel-catalysed amine arylations enabled by tailored ancillary ligand design
Source: Nat Commun. 2016 Mar 23;7:11073. doi: 10.1038/ncomms11073 (PMC4814586; doi:10.1038/ncomms11073)
Supplement: Supplementary Information — Supplementary Figures 1-142, Supplementary Tables 1-2, Supplementary Methods and Supplementary References [file ncomms11073-s1.pdf]

Supplementary Figure 1. ^{1}H NMR of **A**, ($CDCl_3$, 500.1 MHz)

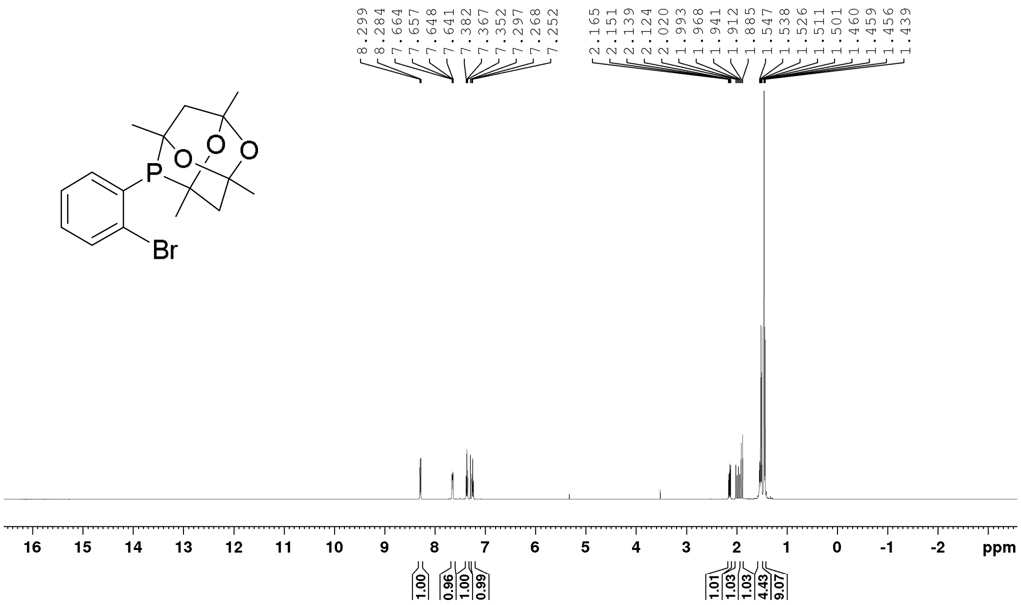

Supplementary Figure 2. ^{13}C{^{1}H} NMR of **A**, ($CDCl_3$, 125.8 MHz)

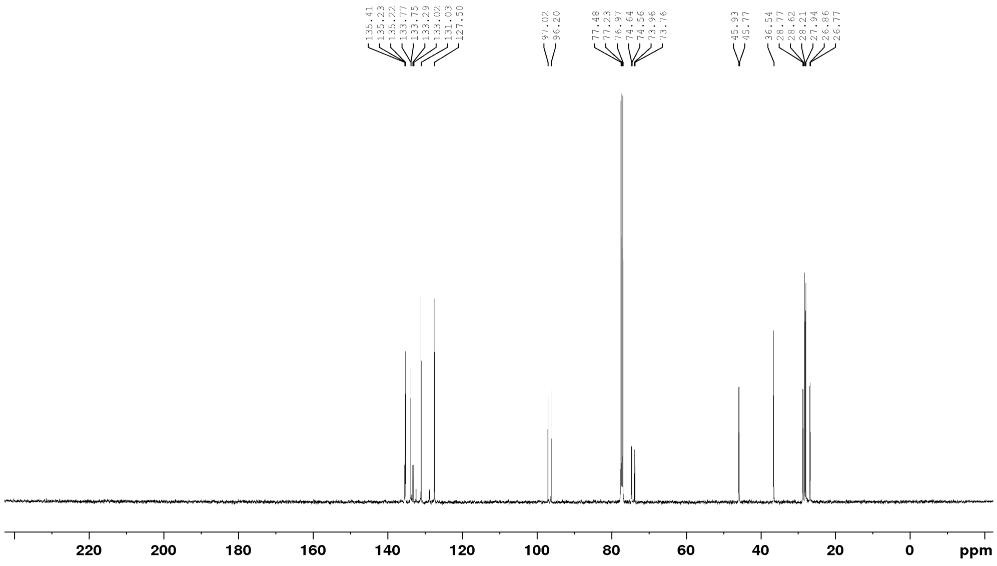

Supplementary Figure 3. $^{31}P\{^{1}H\}$ NMR of **A**, ($CDCl_3$, 202.5 MHz)

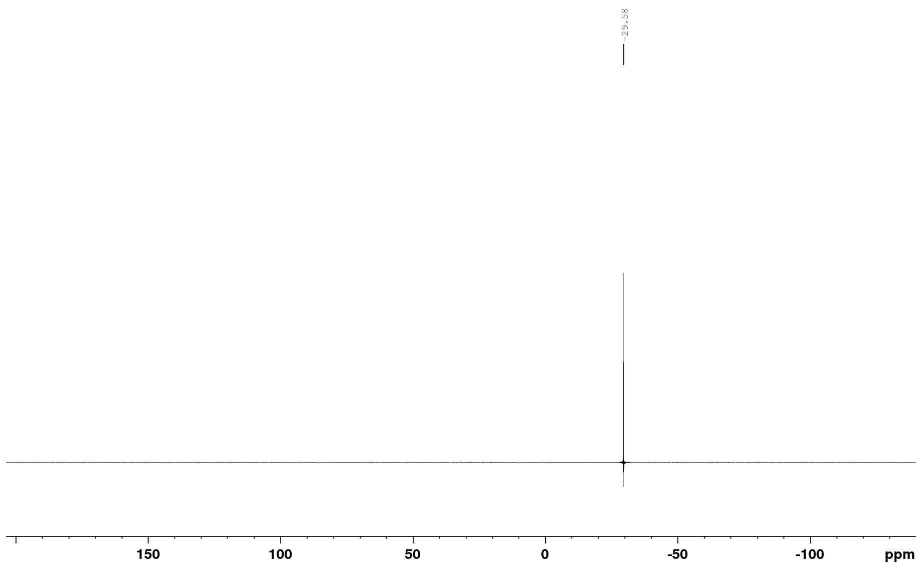

Supplementary Figure 4. ^{1}H NMR of **L1**, ($CDCl_3$, 300.1 MHz)

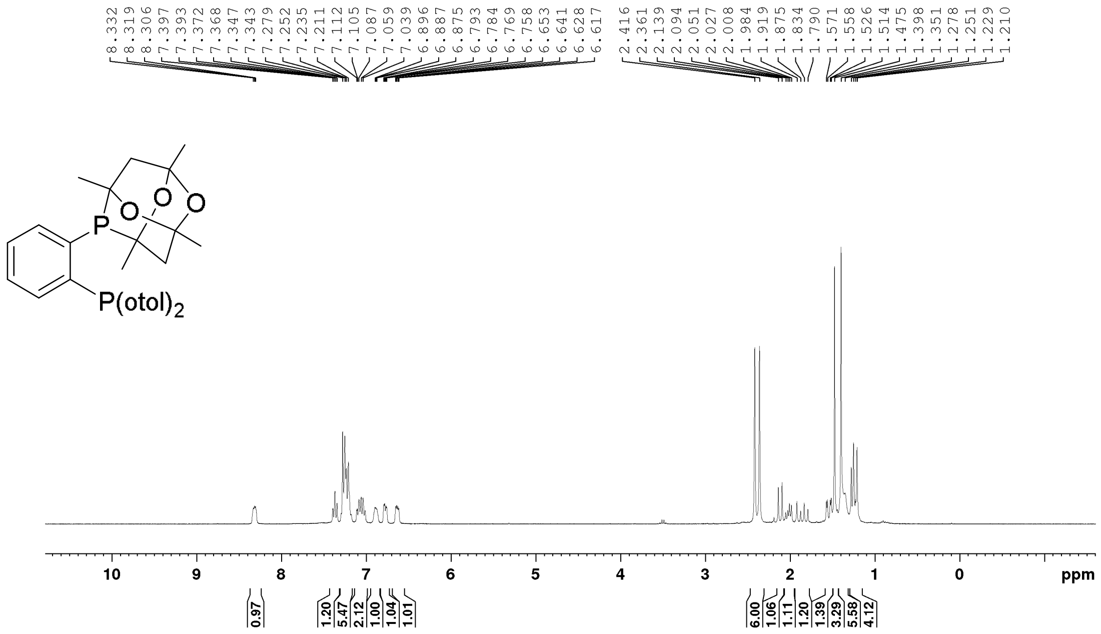

Supplementary Figure 5. $^{13}C\{^1H\}$ NMR of **L1**, ($CDCl_3$, 125.8 MHz)

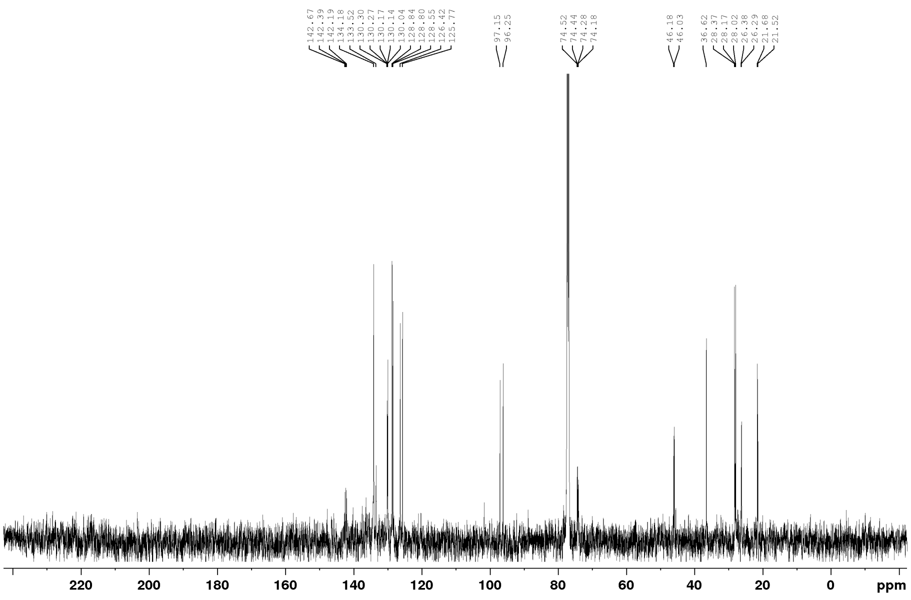

Supplementary Figure 6. $^{31}P\{^1H\}$ NMR of **L1**, ($CDCl_3$, 25 °C, 202.5 MHz)

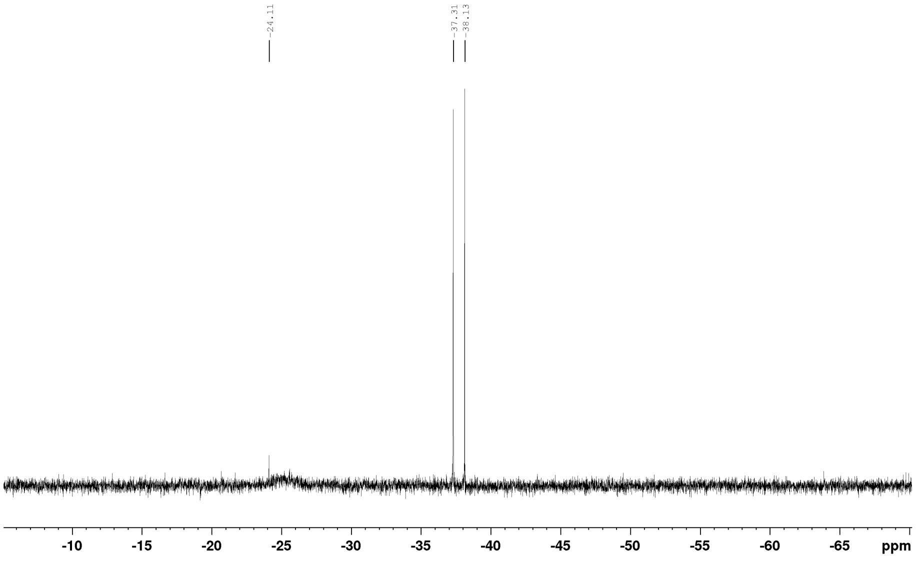

Supplementary Figure 7. $^{31}P\{^{1}H\}$ NMR of **L1**, ($CDCl_3$, -50 °C, 121.5 MHz)

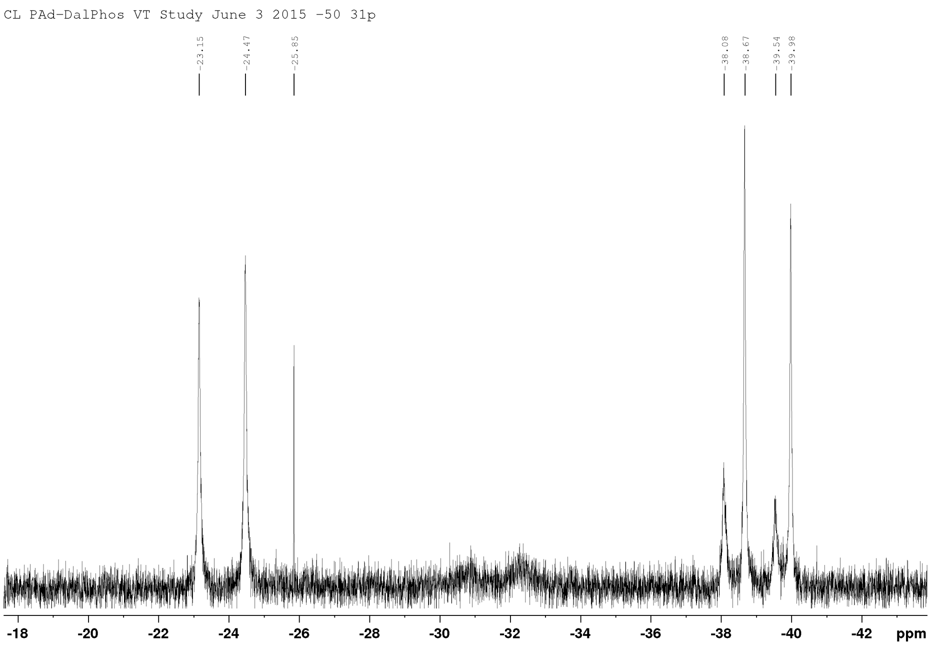

Supplementary Figure 8. ^{1}H NMR of **L2**, ($CDCl_3$, 500.1 MHz)

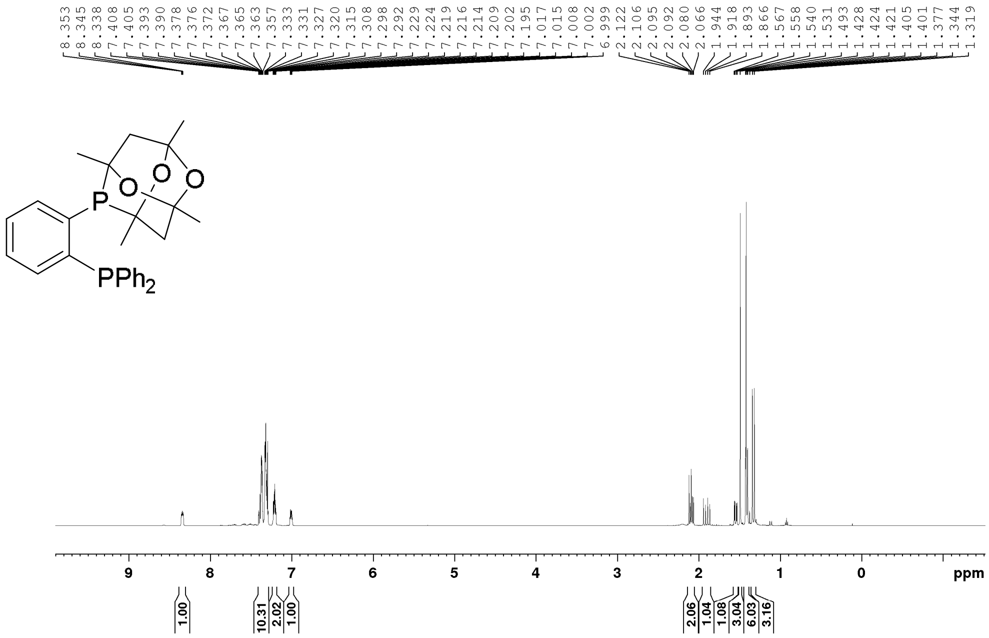

Supplementary Figure 9. $^{13}C\{^{1}H\}$ NMR of **L2,** ($CDCl_3$, 125.8 MHz)

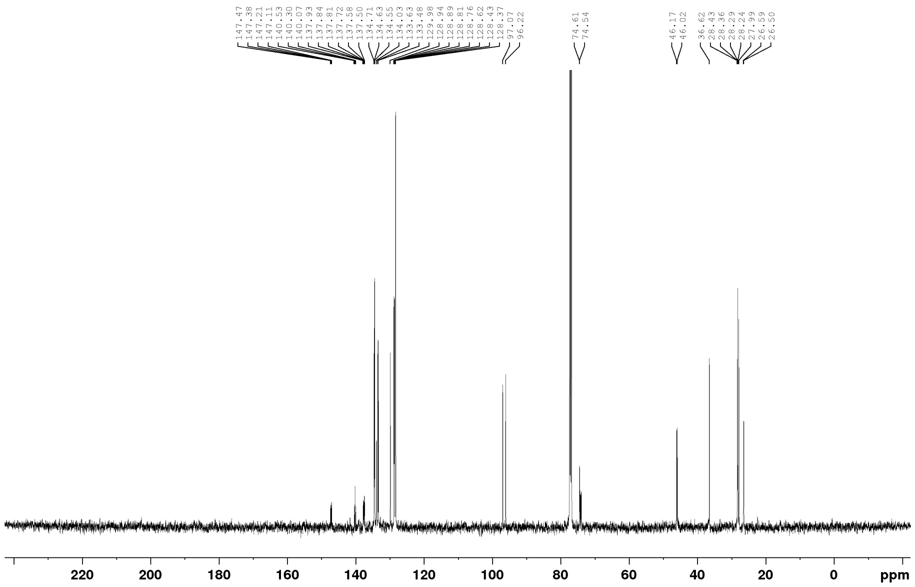

Supplementary Figure 10. $^{31}P\{^{1}H\}$ NMR of **L2**, ($CDCl_3$, 202.5 MHz)

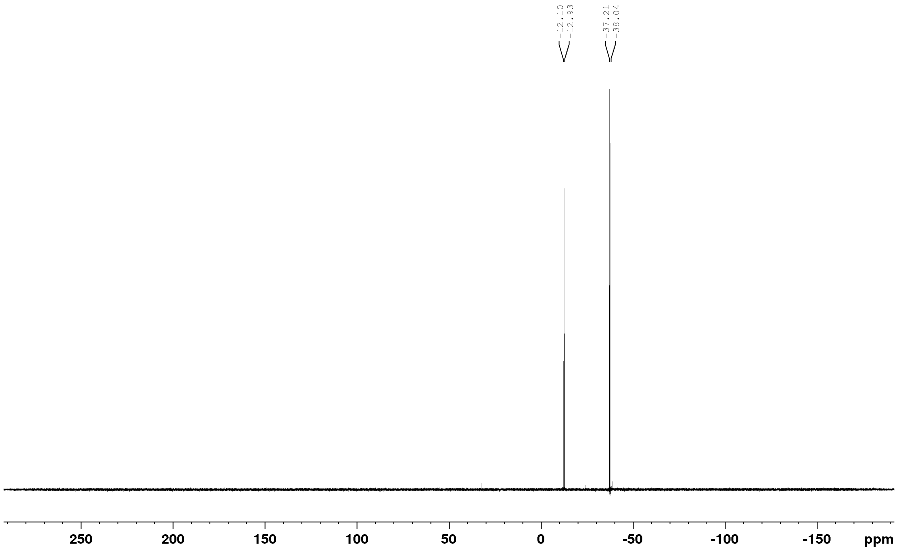

Supplementary Figure 11. ^{1}H NMR of **L3**, ($CDCl_3$, 300.1 MHz)

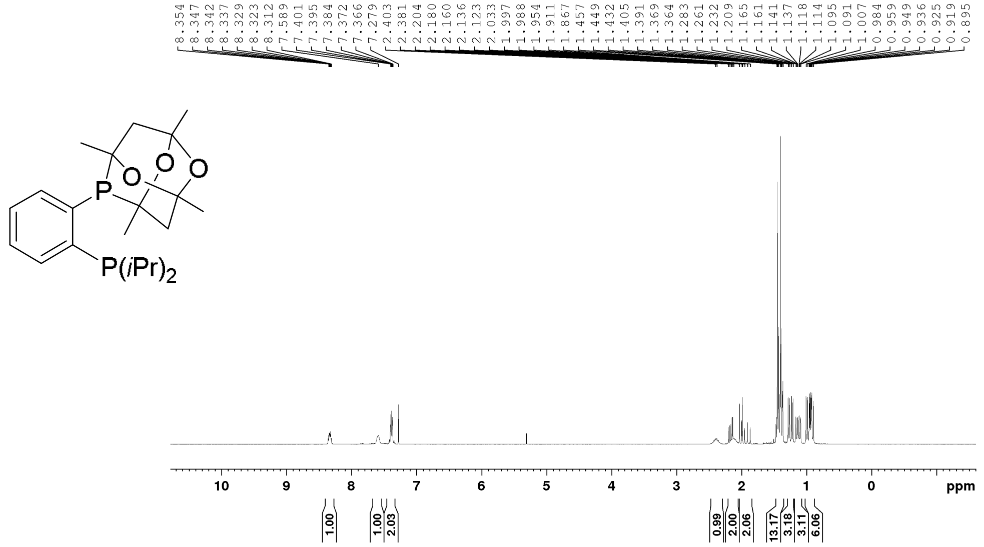

Supplementary Figure 12. ^{13}C{^{1}H} NMR of **L3**, ($CDCl_3$, 125.8 MHz)

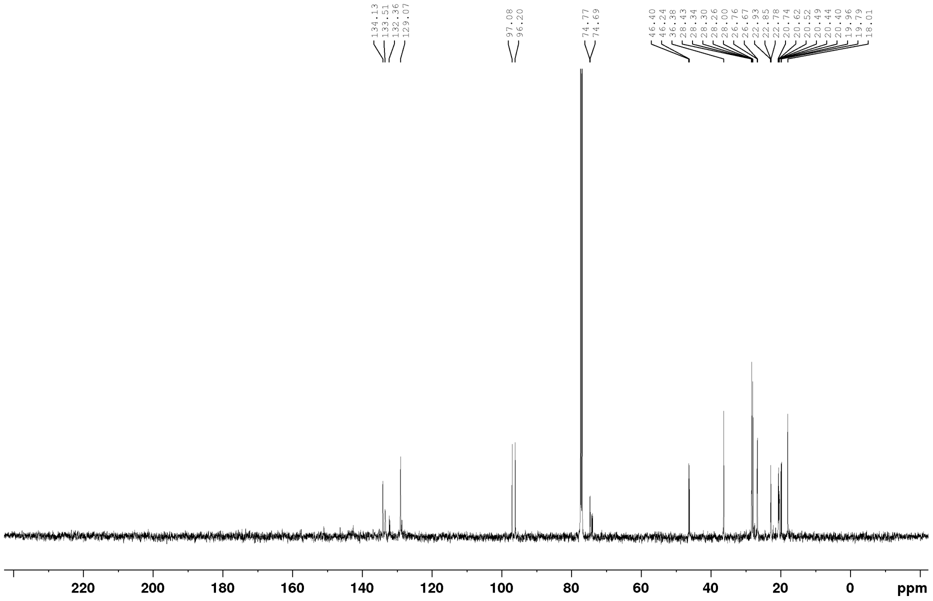

Supplementary Figure 13. $^{31}P\{^{1}H\}$ NMR of **L3**, ($CDCl_3$, 202.5 MHz)

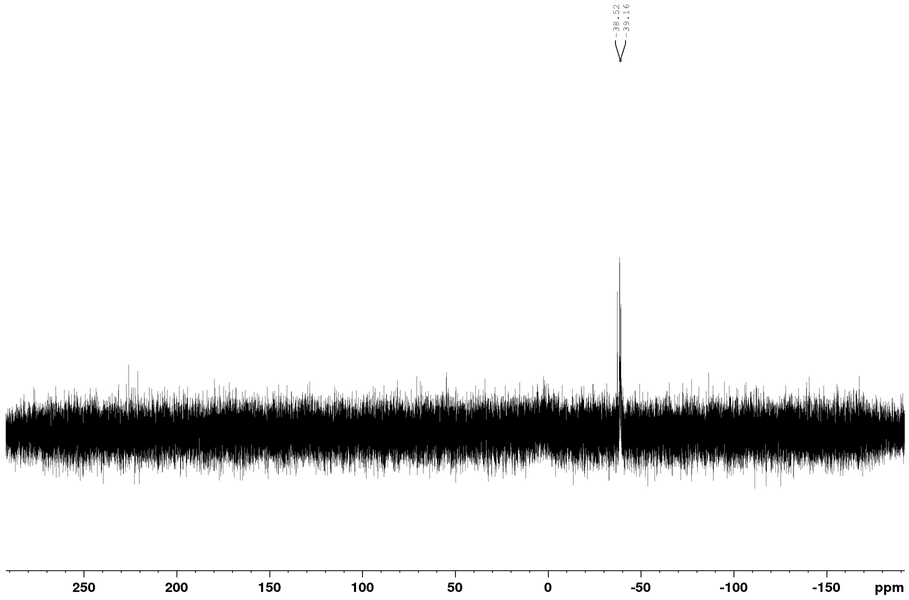

Supplementary Figure 14. ^{1}H NMR of **L4**, ($CDCl_3$, 500.1 MHz)

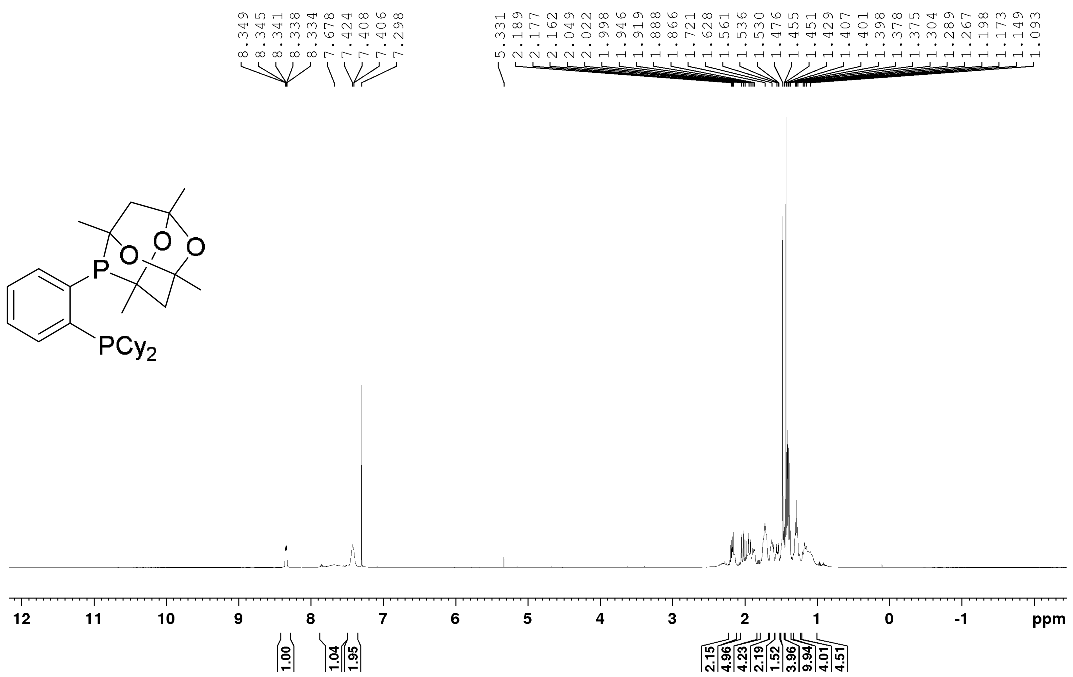

Supplementary Figure 15. $^{13}C\{^{1}H\}$ NMR of **L4**, ($CDCl_3$, 125.8 MHz)

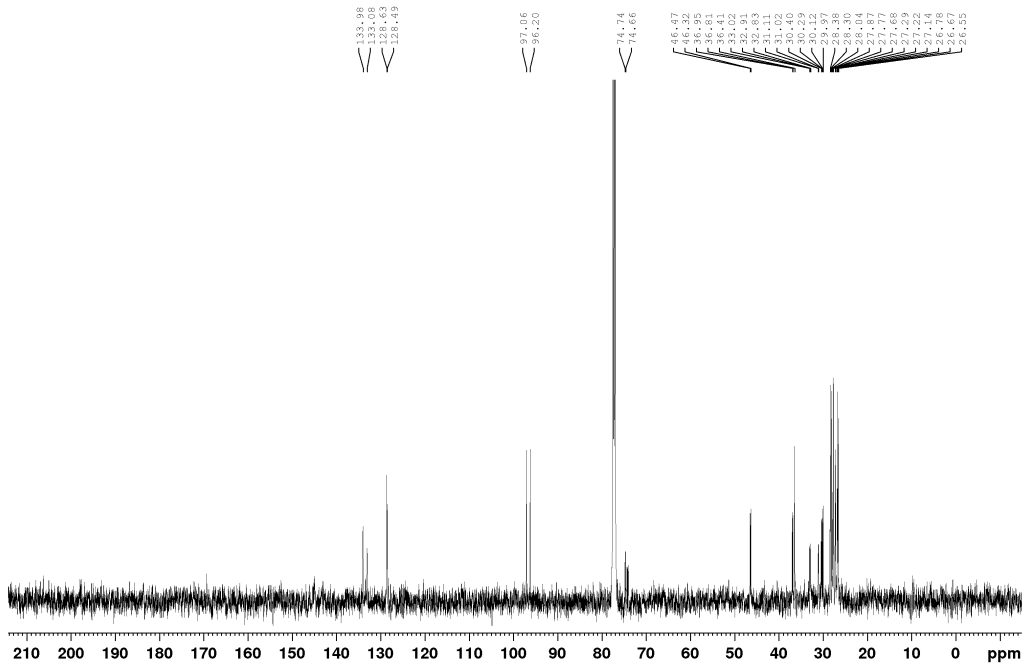

Supplementary Figure 16. $^{31}P\{^{1}H\}$ NMR of **L4,** ($CDCl_3$, 202.5 MHz)

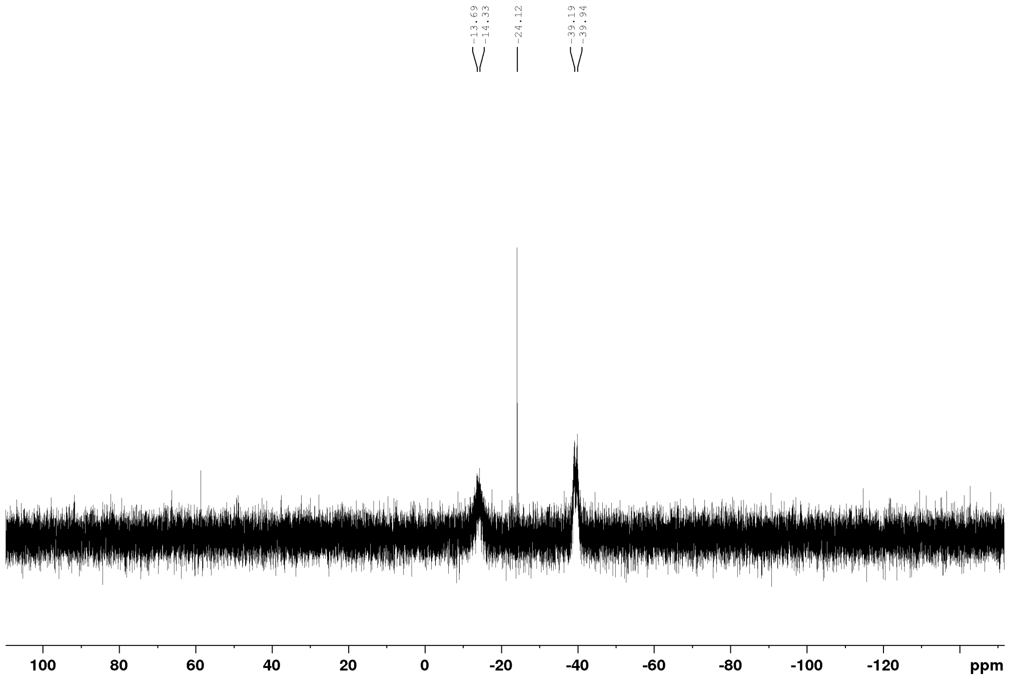

Supplementary Figure 17. ^{1}H NMR of **L5** ($CDCl_3$, 300.1 MHz)

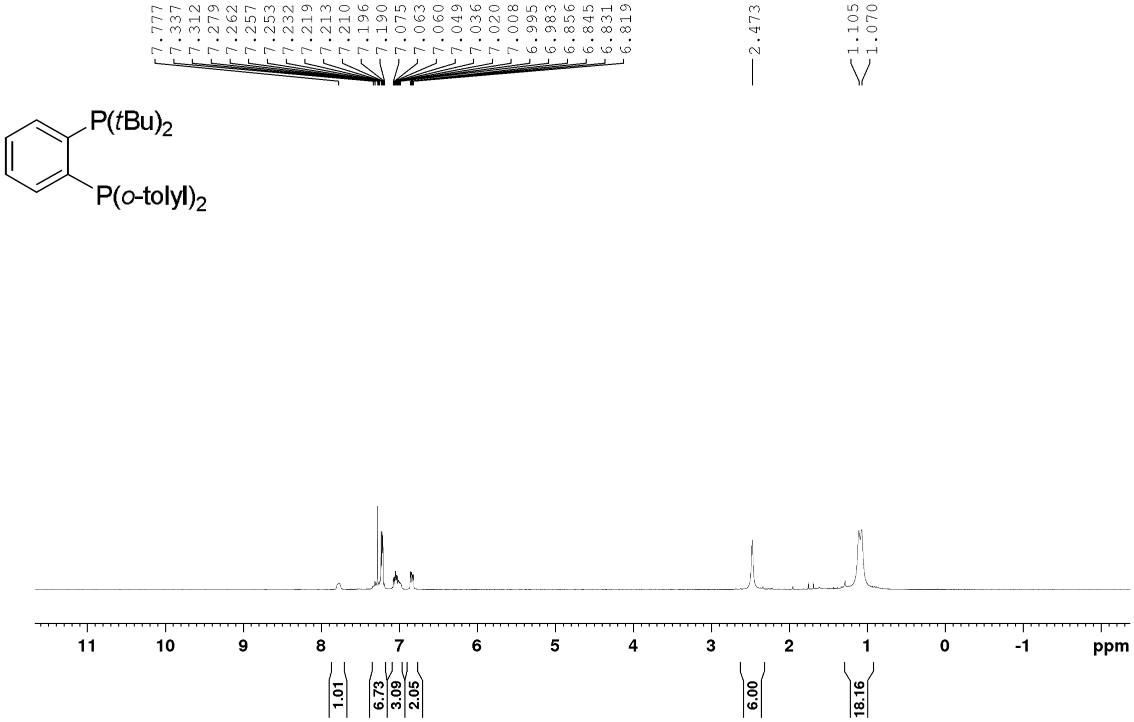

Supplementary Figure 18. ^{13}C NMR of **L5** ($CDCl_3$, 125.8 MHz)

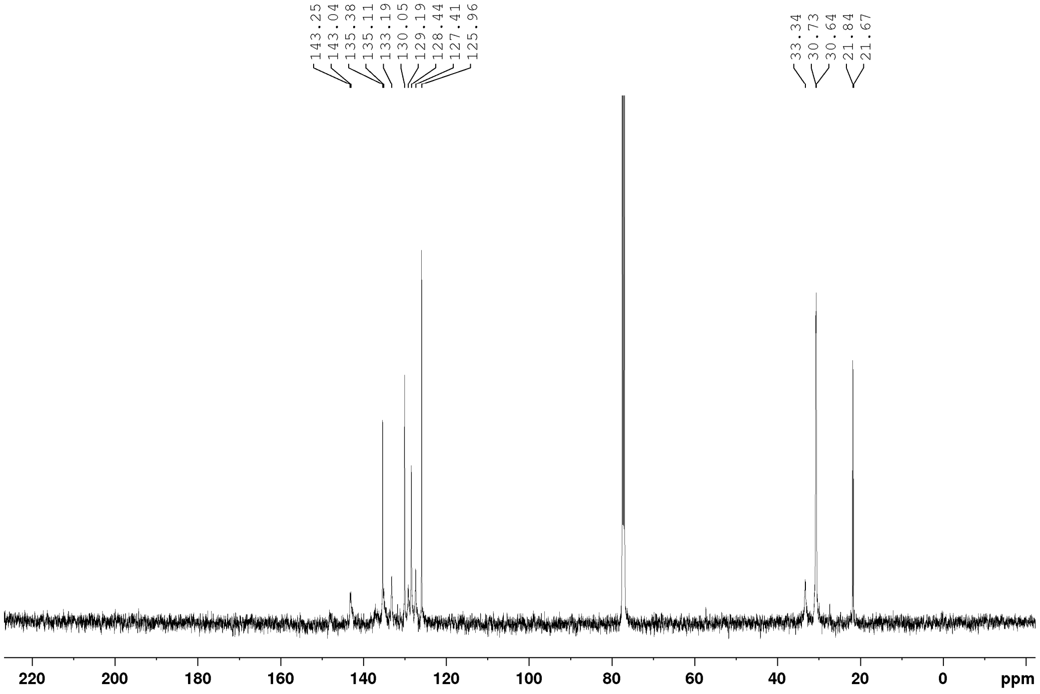

Supplementary Figure 19. ^{31}P NMR of **L5** ($CDCl_3$, 121.4 MHz)

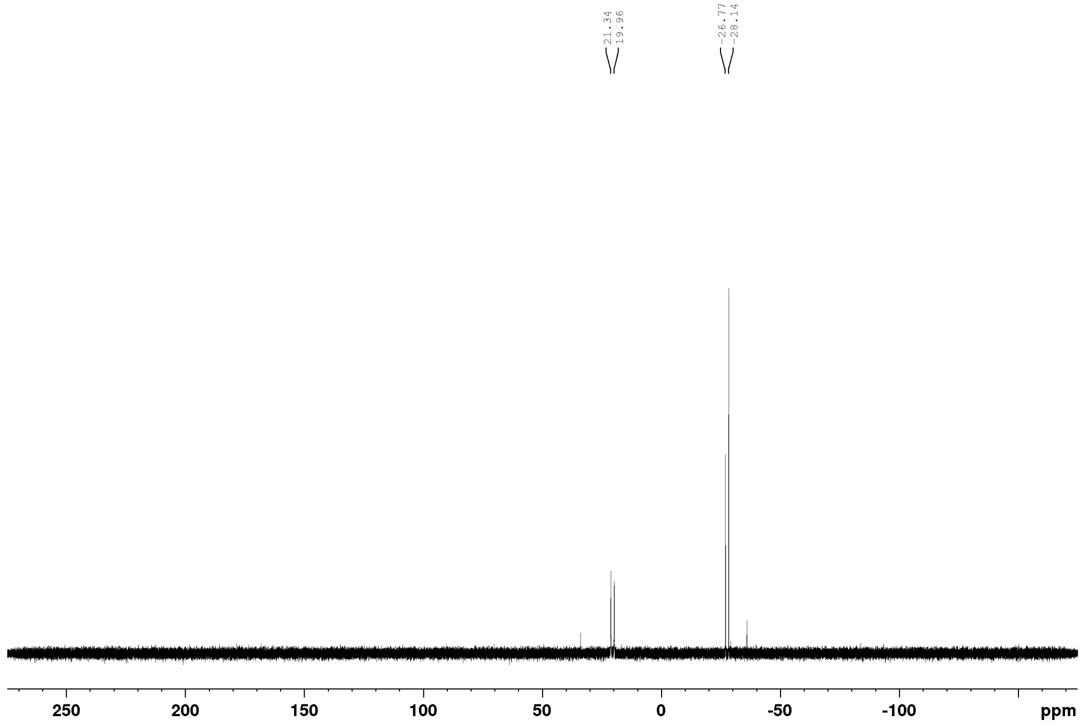

Supplementary Figure 20. ^{31}P{^{1}H} NMR of **C1**, ($CDCl_3$, 202.5 MHz)

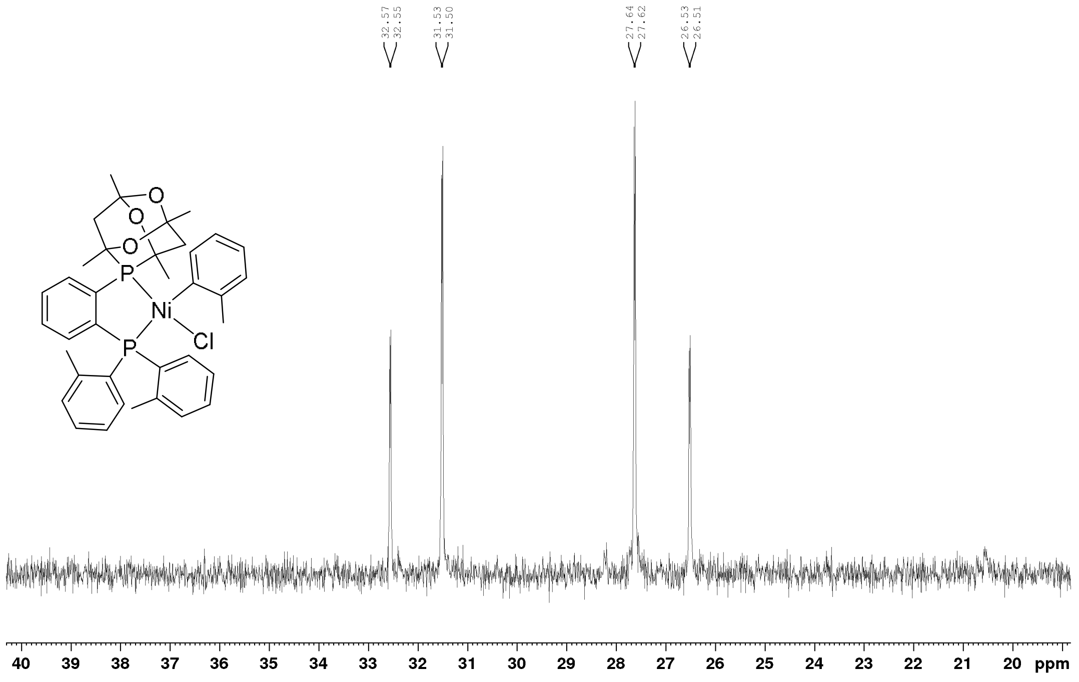

Supplementary Figure 21. ^{1}H NMR of 4-phenylaniline, **2a** ($CDCl_3$, 300.1 MHz)

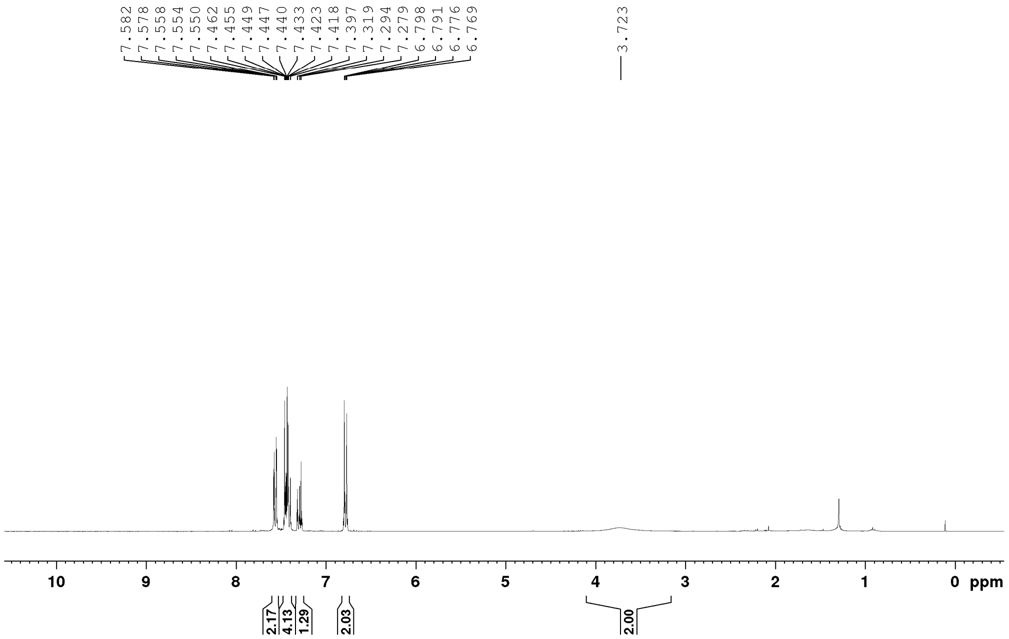

Supplementary Figure 22. ^{13}C NMR of 4-phenylaniline, **2a** ($CDCl_3$, 75.5 MHz)

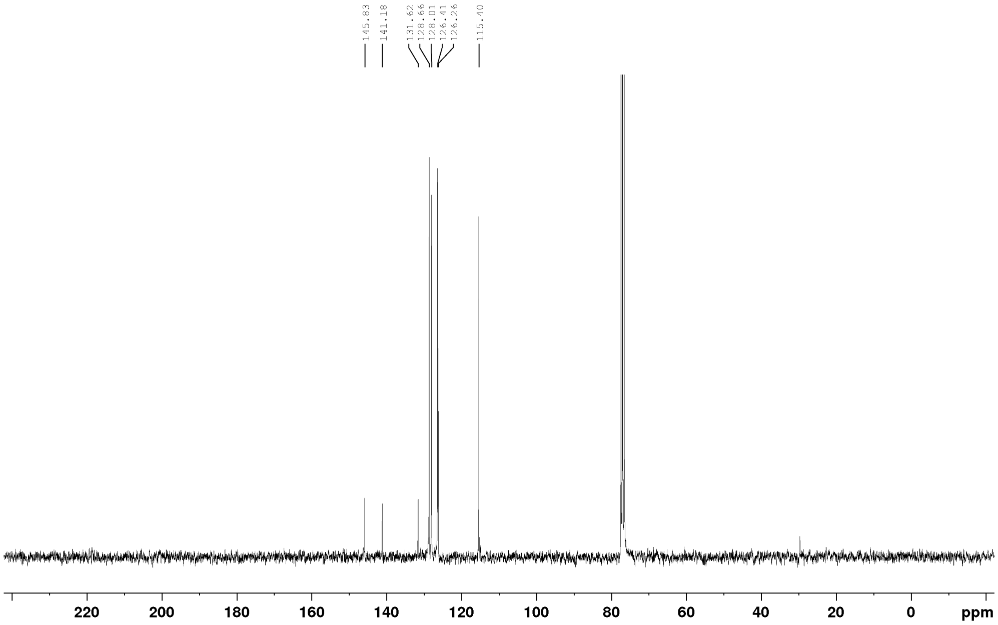

Supplementary Figure 23. ^{1}H NMR of 4'-methylbiphenyl-4-amine, **2b** ($CDCl_3$, 500.1 MHz)

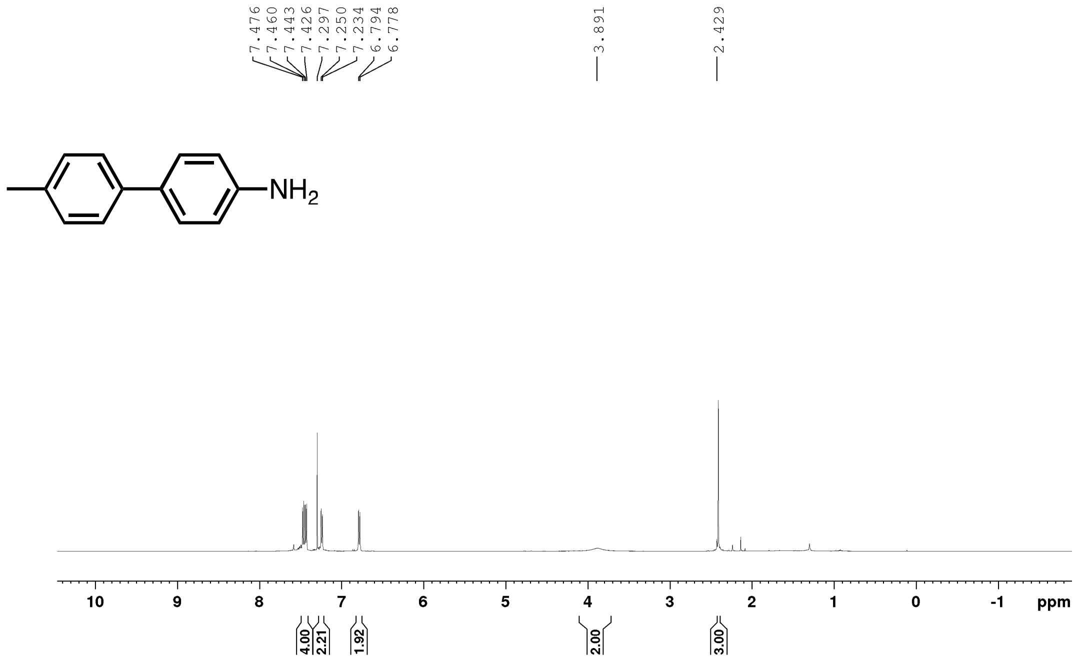

Supplementary Figure 24. $^{13}C\{^1H\}$ NMR of 4'-methylbiphenyl-4-amine, **2b** ($CDCl_3$, 125.8 MHz)

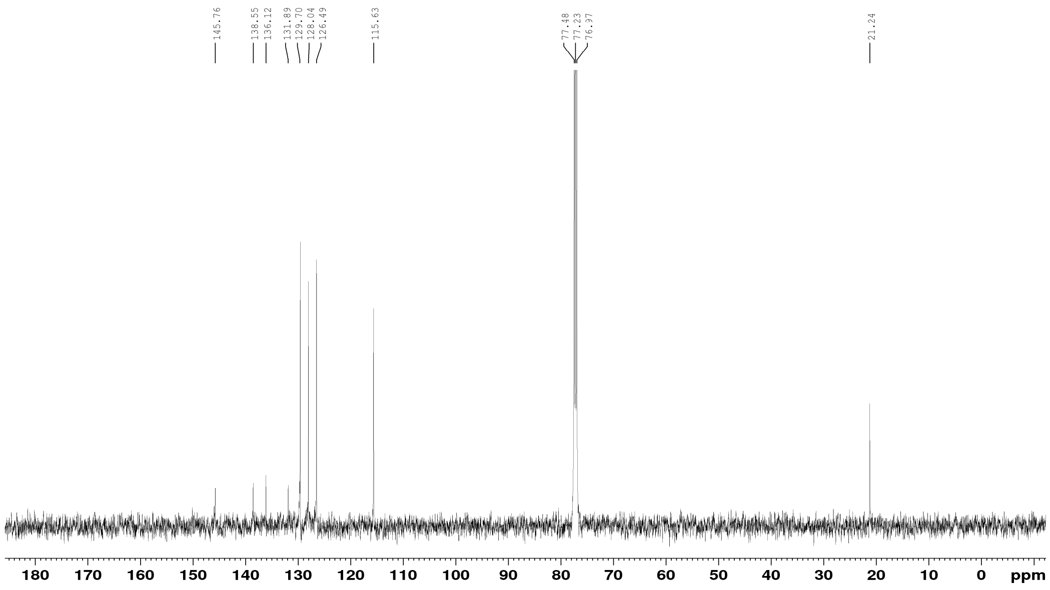

Supplementary Figure 25. ^{1}H NMR of 4'-methoxybiphenyl-4-amine, **2c** ($CDCl_3$, 500.1 MHz)

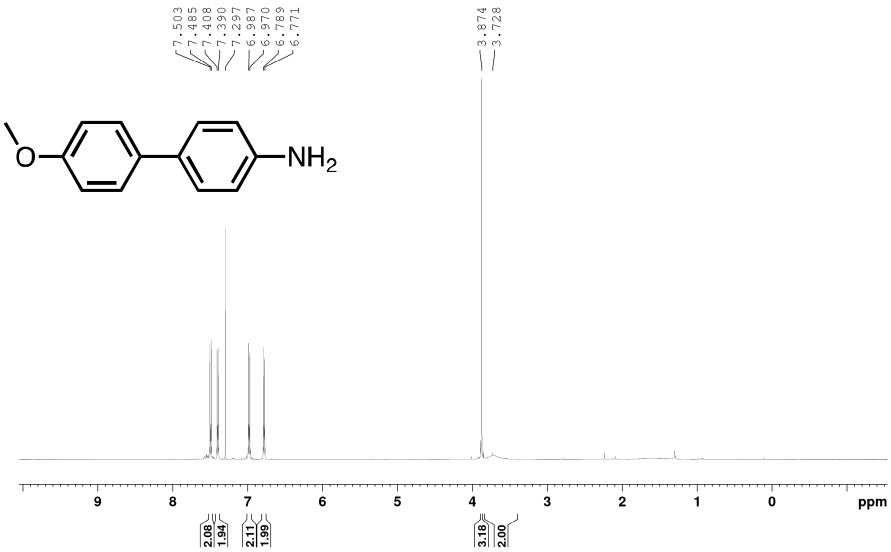

Supplementary Figure 26. $^{13}C\{^{1}H\}$ NMR of 4'-methoxybiphenyl-4-amine, **2c** ($CDCl_3$, 125.8 MHz)

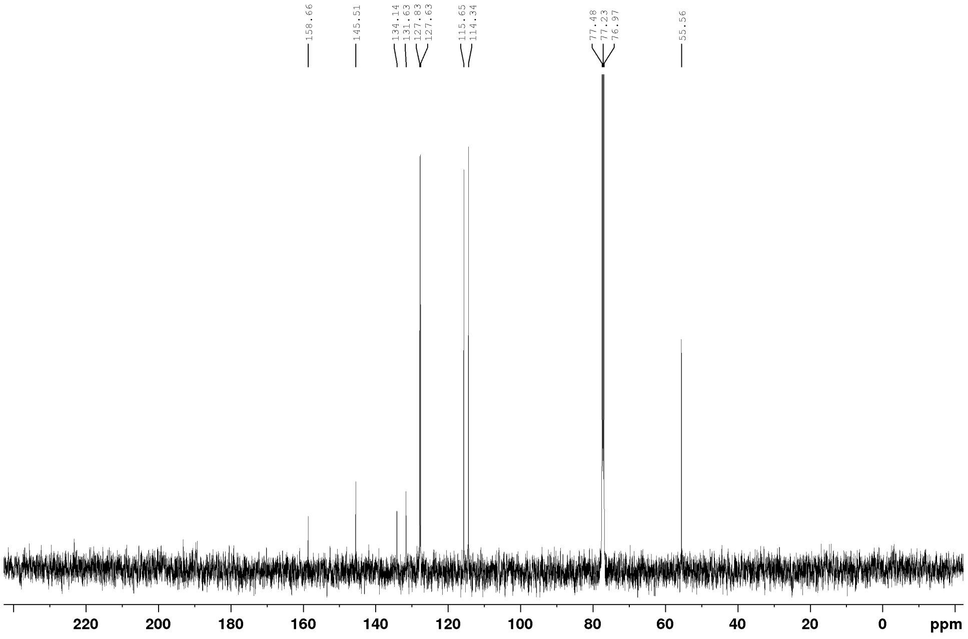

Supplementary Figure 27. ^{1}H NMR of Naphthalen-1-amine, **2d** ($CDCl_3$, 500.1 MHz)

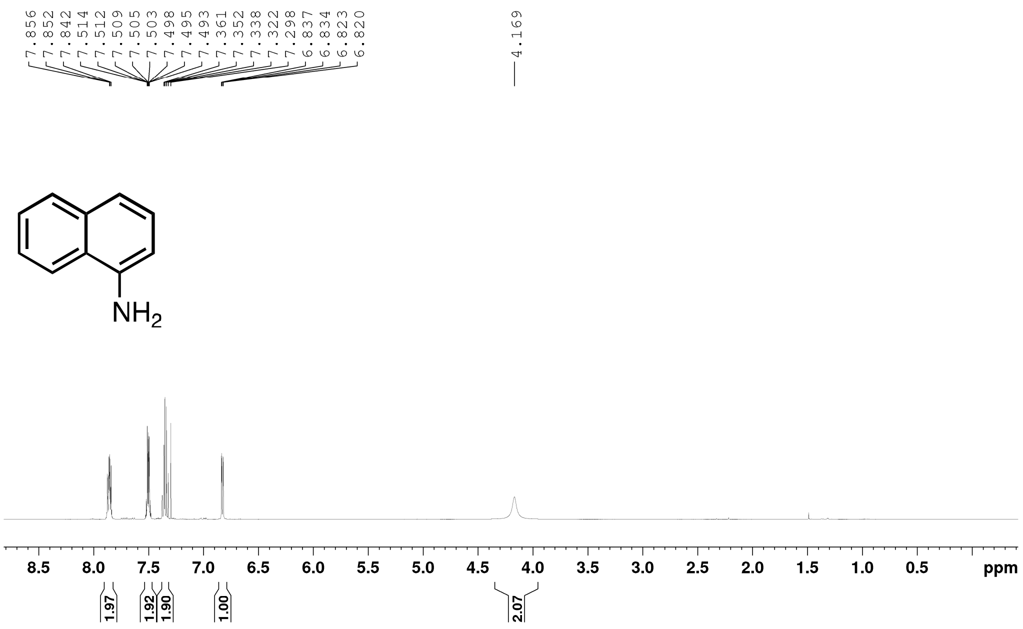

Supplementary Figure 28. ^{13}C{^{1}H} NMR of Naphthalen-1-amine, **2d** ($CDCl_3$, 128.1 MHz)

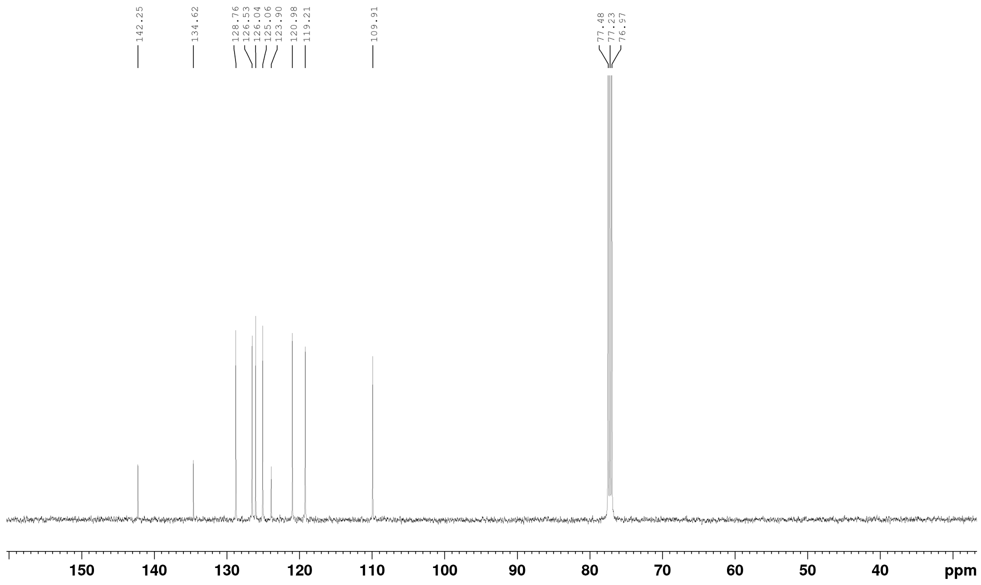

Supplementary Figure 29. ^{1}H NMR of Naphthalen-2-amine, **2e** ($CDCl_3$, 500.1 MHz)

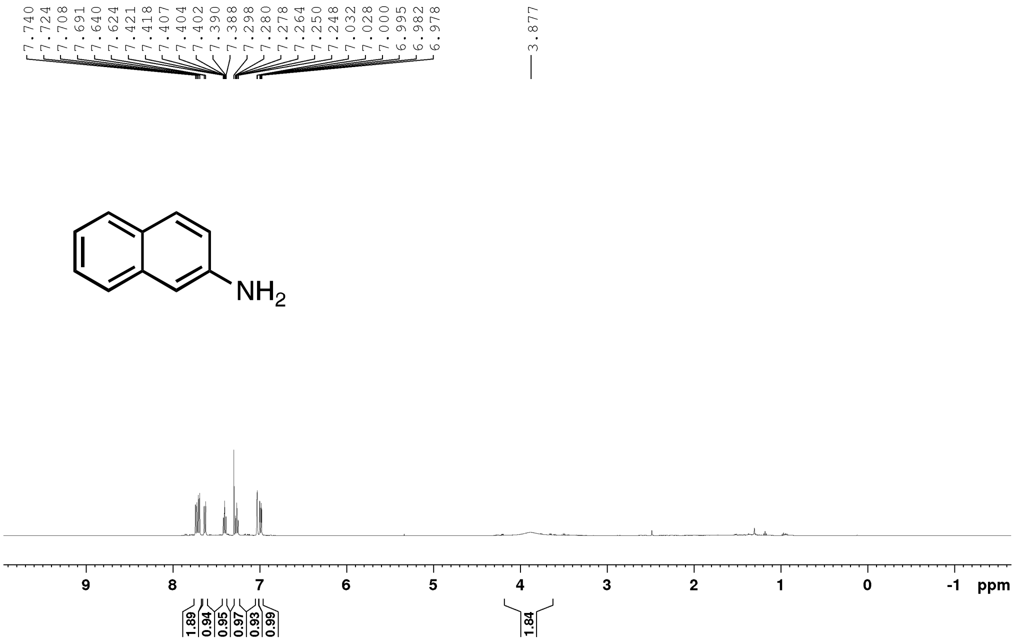

Supplementary Figure 30. ^{13}C{^{1}H} NMR of Naphthalen-2-amine, **2e** ($CDCl_3$, 125.8 MHz)

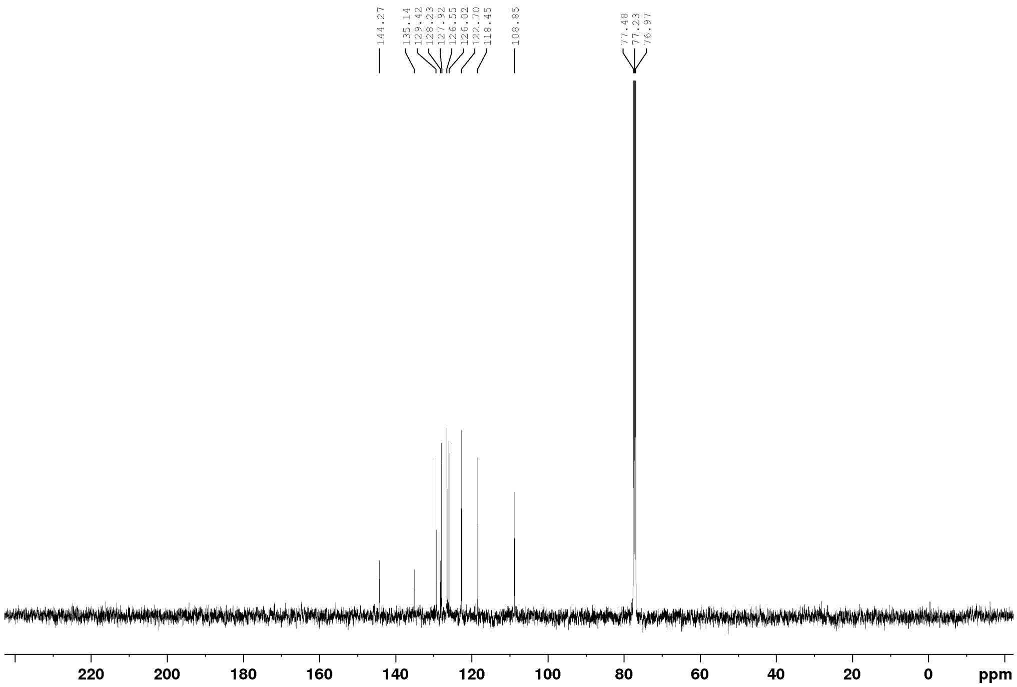

Supplementary Figure 31. ^{1}H NMR of [1,1'-Biphenyl]-2-amine, **2f** ($CDCl_3$, 300.1 MHz)

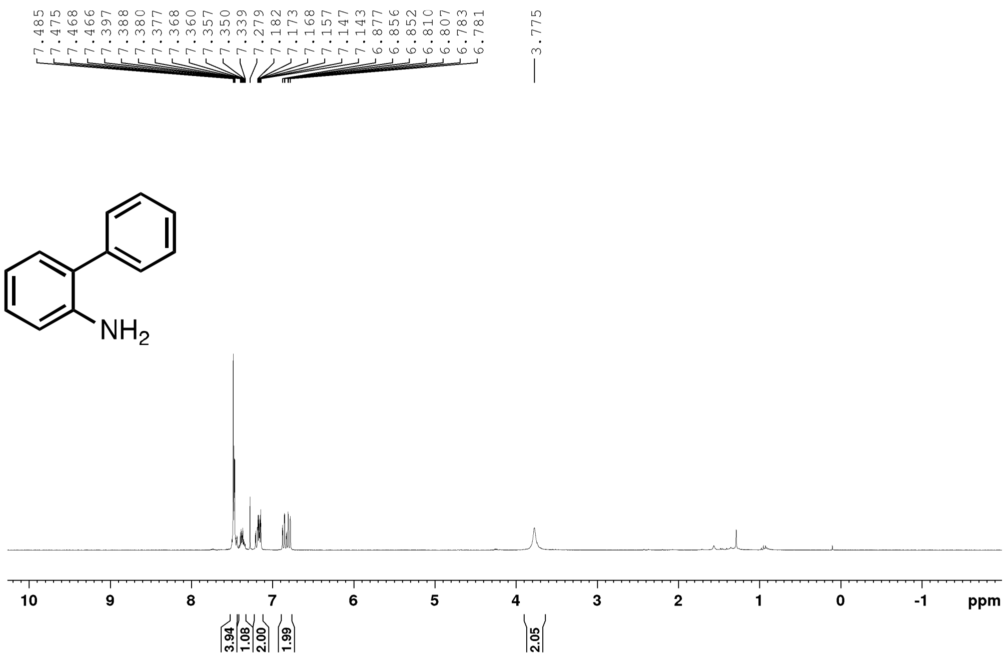

Supplementary Figure 32. ^{13}C{^{1}H} NMR of [1,1'-Biphenyl]-2-amine, **2f** ($CDCl_3$, 125.8 MHz)

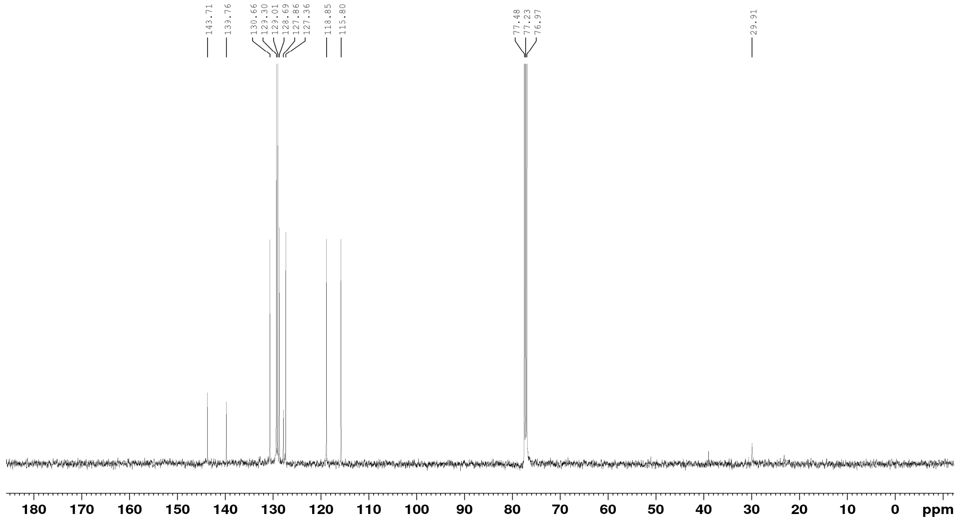

Supplementary Figure 33. ^{1}H NMR of 4-(1H-pyrrol-1yl)aniline, **2g** ($CDCl_3$, 500.1 MHz)

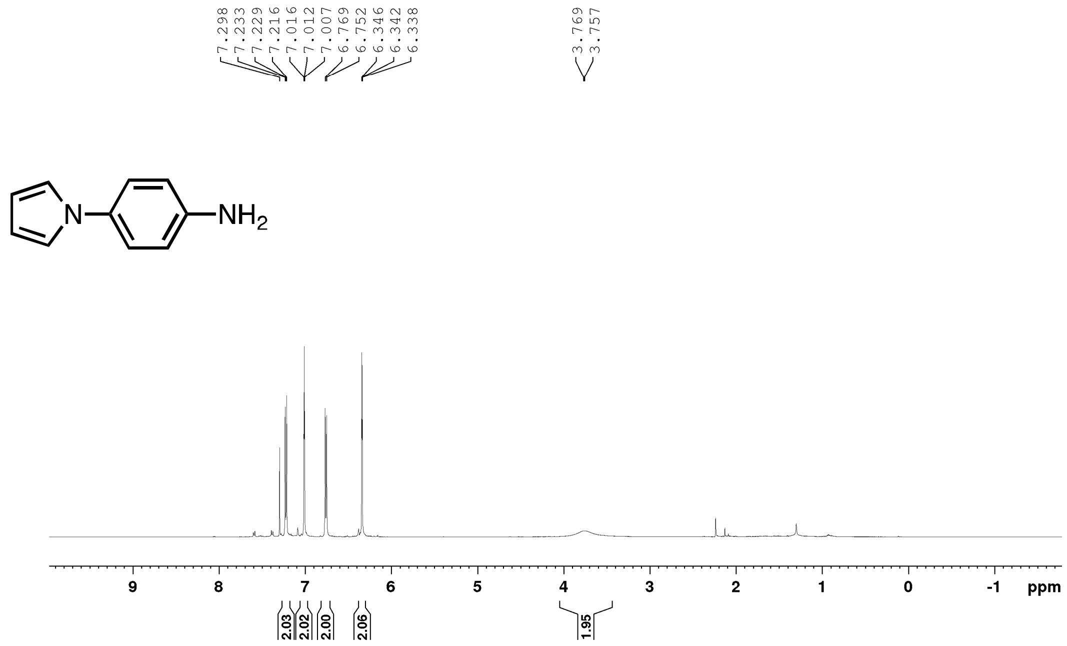

Supplementary Figure 34. ^{13}C NMR of 4-(1H-pyrrol-1yl)aniline, **2g** ($CDCl_3$, 125.8 MHz)

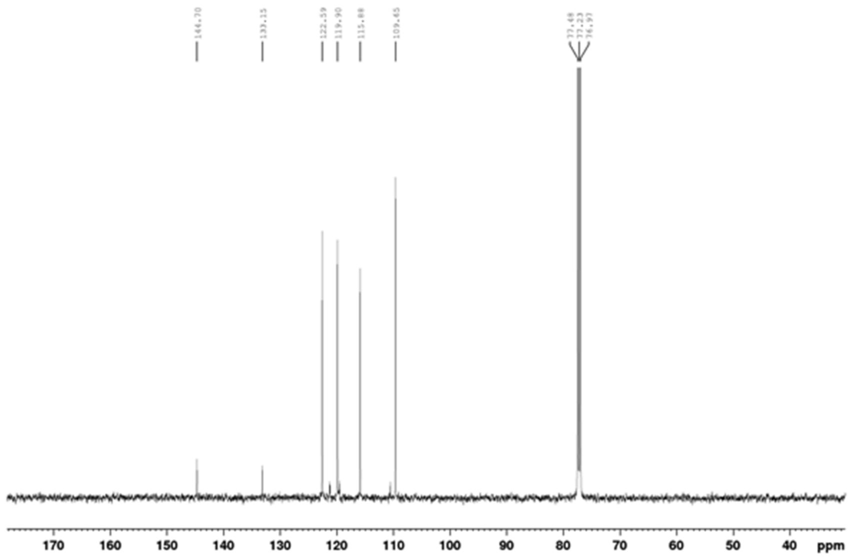

Supplementary Figure 35. ^{1}H NMR of 2,5-dimethylaniline, **2h** ($CDCl_3$, 500.1 MHz)

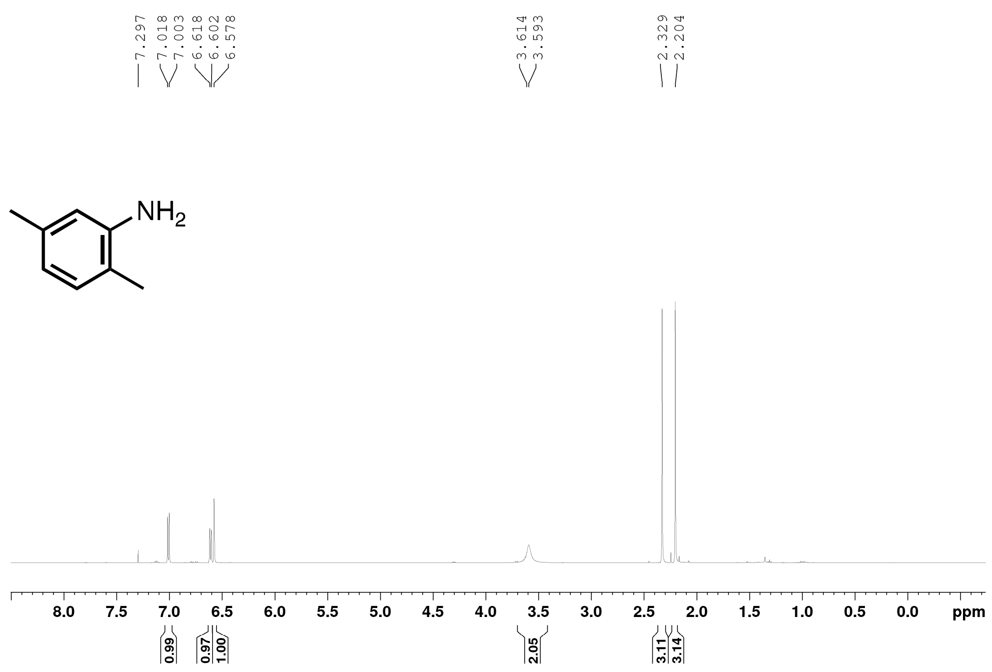

Supplementary Figure 36. $^{13}C\{^{1}H\}$ NMR of 2,5-dimethylaniline, **2h** ($CDCl_3$, 125.8 MHz)

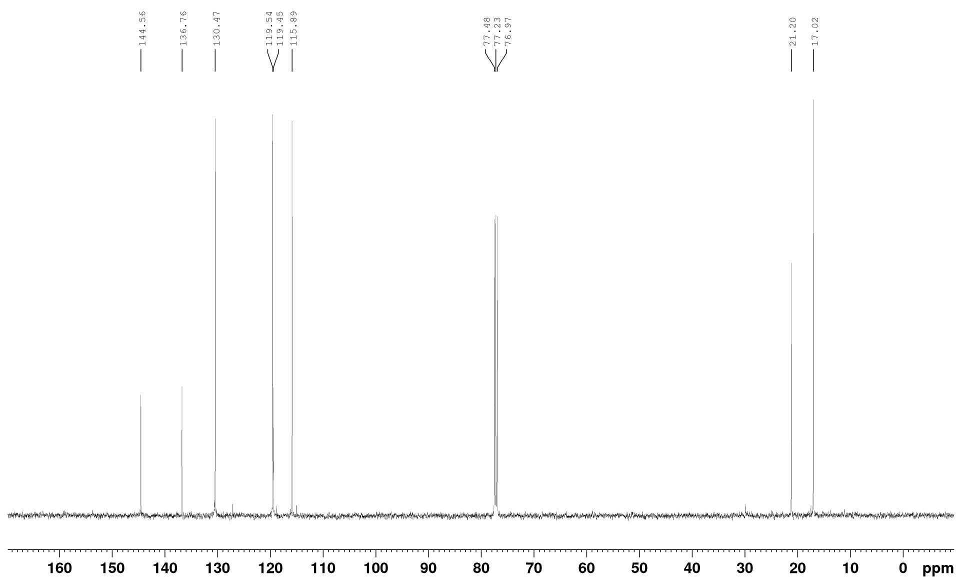

Supplementary Figure 37. ^{1}H NMR of 3-amino-4-methylbenzonitrile, **2i** ($CDCl_3$, 300.1 MHz)

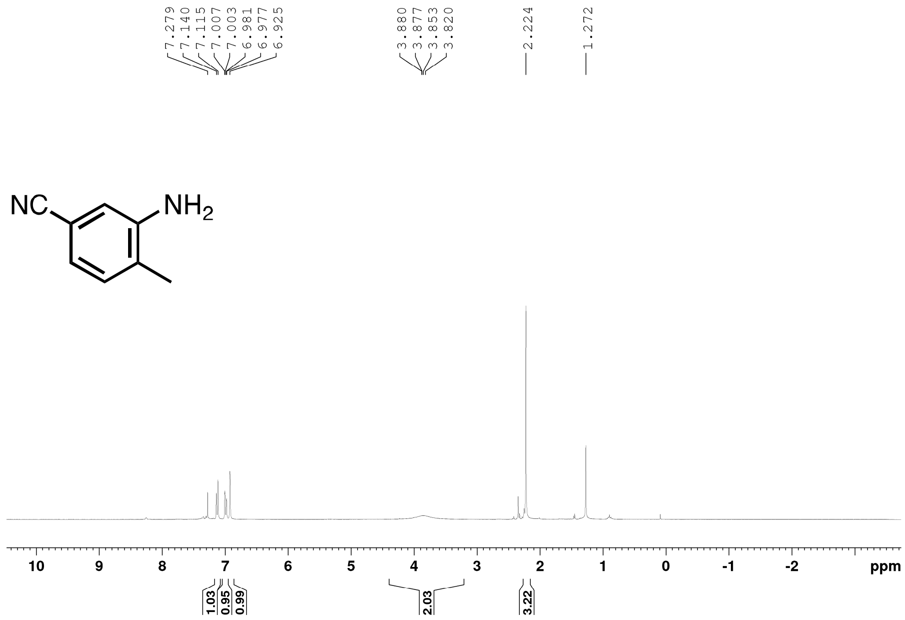

Supplementary Figure 38. $^{13}C\{^{1}H\}$ NMR of 3-amino-4-methylbenzonitrile, **2i** ($CDCl_3$, 125.8 MHz)

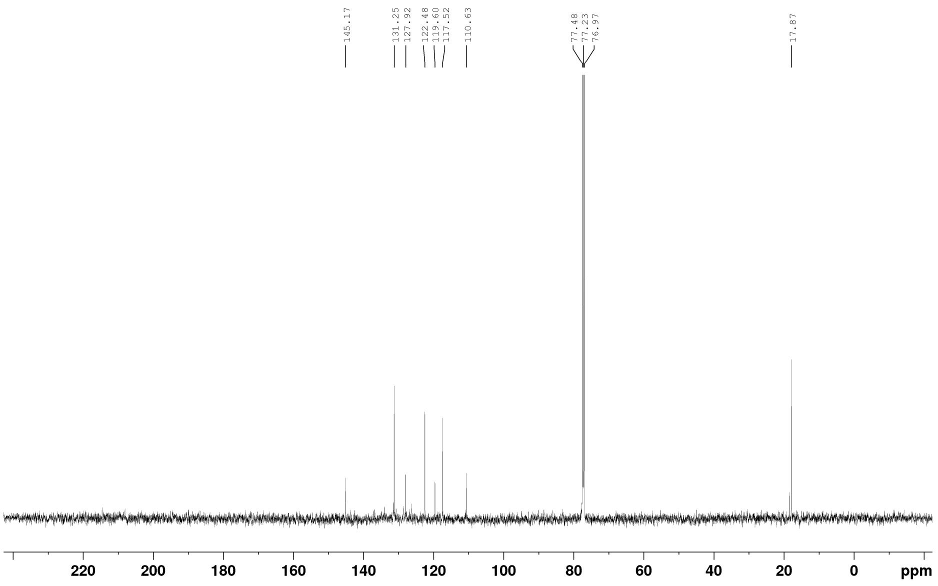

Supplementary Figure 39. ^{1}H NMR of N-(5-fluoro-2-methylphenyl)-4-methylbenzenesulfonamide, **2j** ($CDCl_3$, 300.1 MHz)

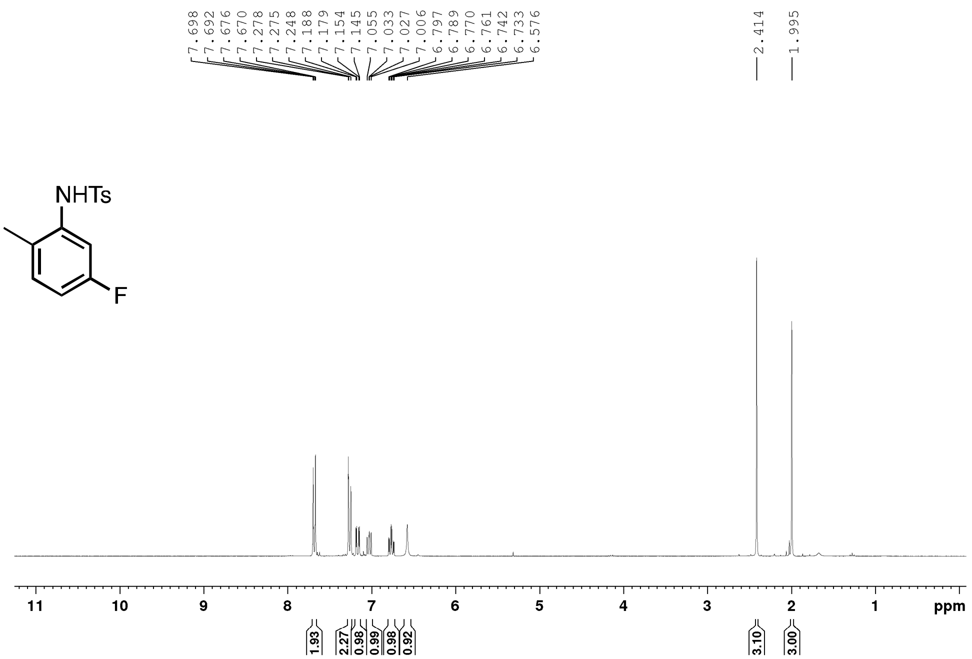

Supplementary Figure 40. $^{13}C\{^{1}H\}$ NMR of N-(5-fluoro-2-methylphenyl)-4-methylbenzenesulfonamide, **2j** ($CDCl_3$, 75.5 MHz)

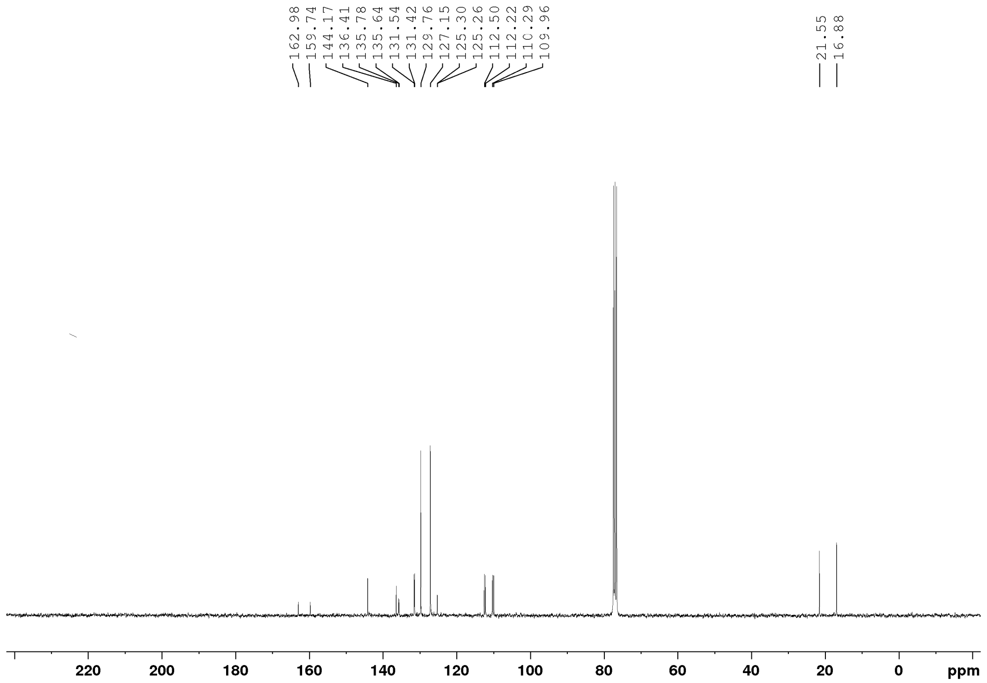

Supplementary Figure 41. ^{1}H NMR of N-(2-methoxyphenyl)-4-methylbenzenesulfonamide, **2k** ($CDCl_3$, 300.1 MHz)

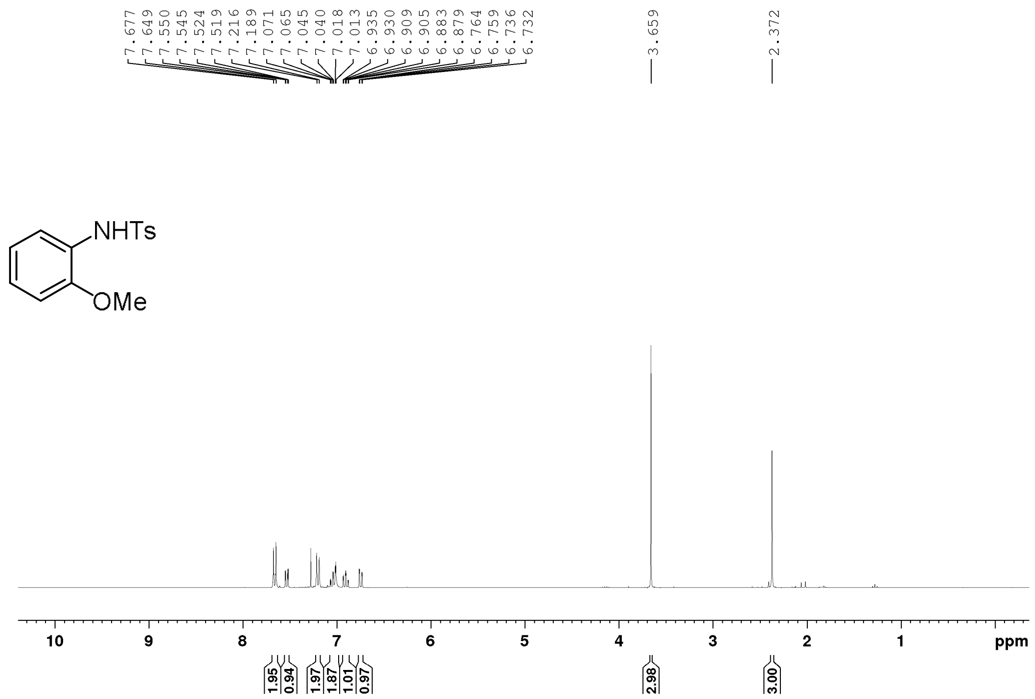

Supplementary Figure 42. $^{13}C\{^{1}H\}$ NMR of N-(2-methoxyphenyl)-4-methylbenzenesulfonamide, **2k** ($CDCl_3$, 75.5 MHz)

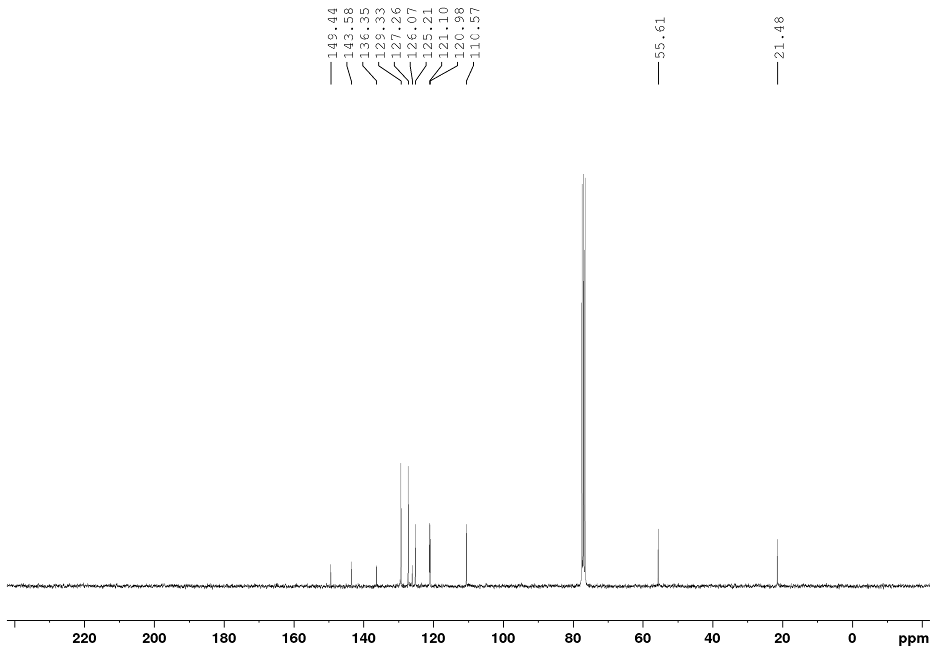

Supplementary Figure 43. ^{1}H NMR of 2-(2-Methyl-1,3-dioxolan-2-yl)aniline, **2m** ($CDCl_3$, 500.1 MHz)

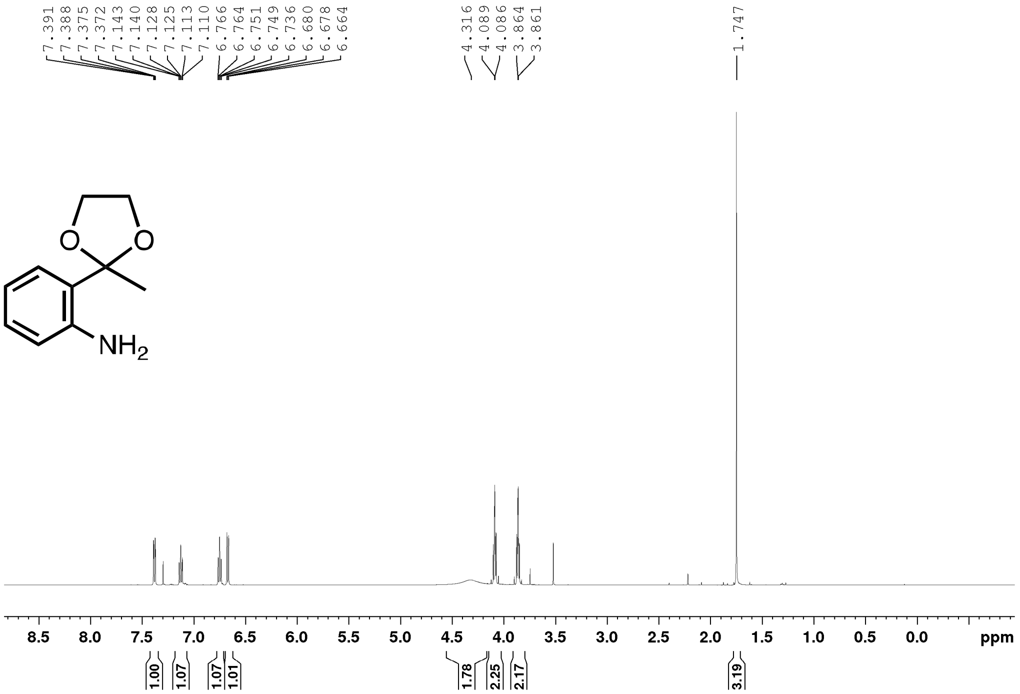

Supplementary Figure 44. $^{13}C\{^{1}H\}$ NMR of 2-(2-Methyl-1,3-dioxolan-2-yl)aniline, **2m** ($CDCl_3$, 125.8 MHz)

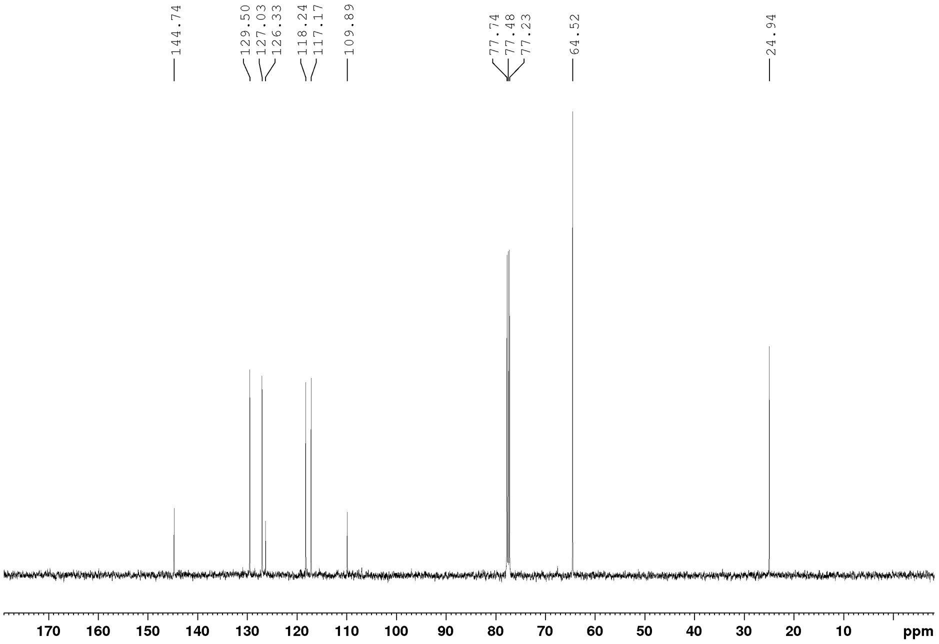

Supplementary Figure 45. ^{1}H NMR of 4-aminobenzophenone, **2n** ($CDCl_3$, 300.1 MHz)

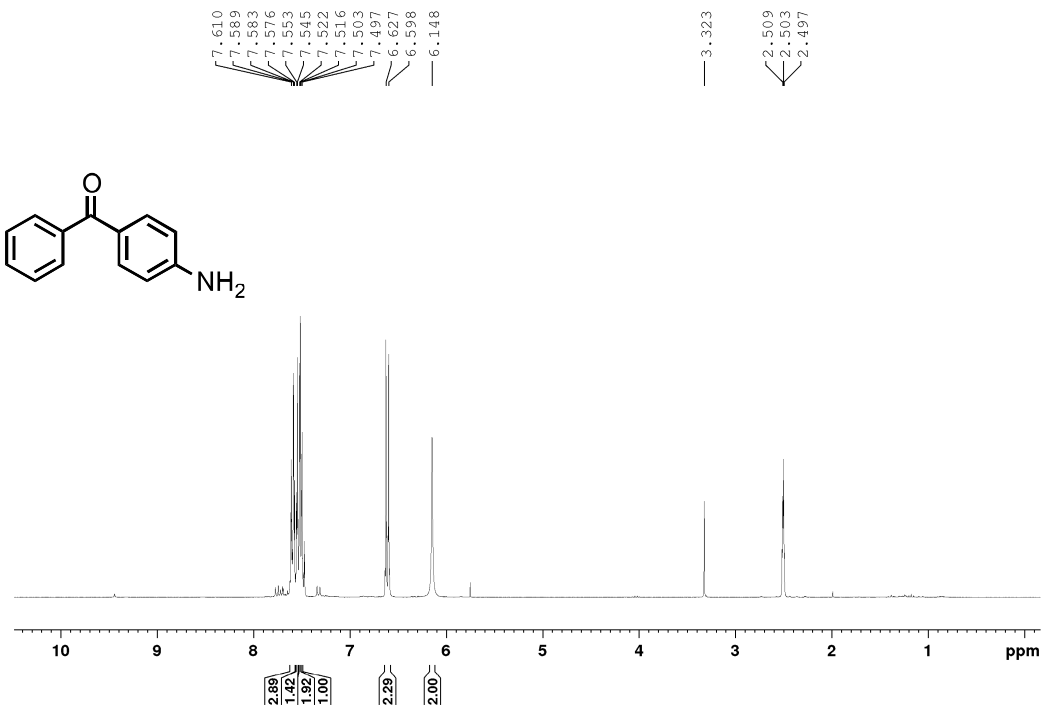

Supplementary Figure 46. ^{13}C{^{1}H} NMR of 4-aminobenzophenone, **2n** ($CDCl_3$, 75.4 MHz)

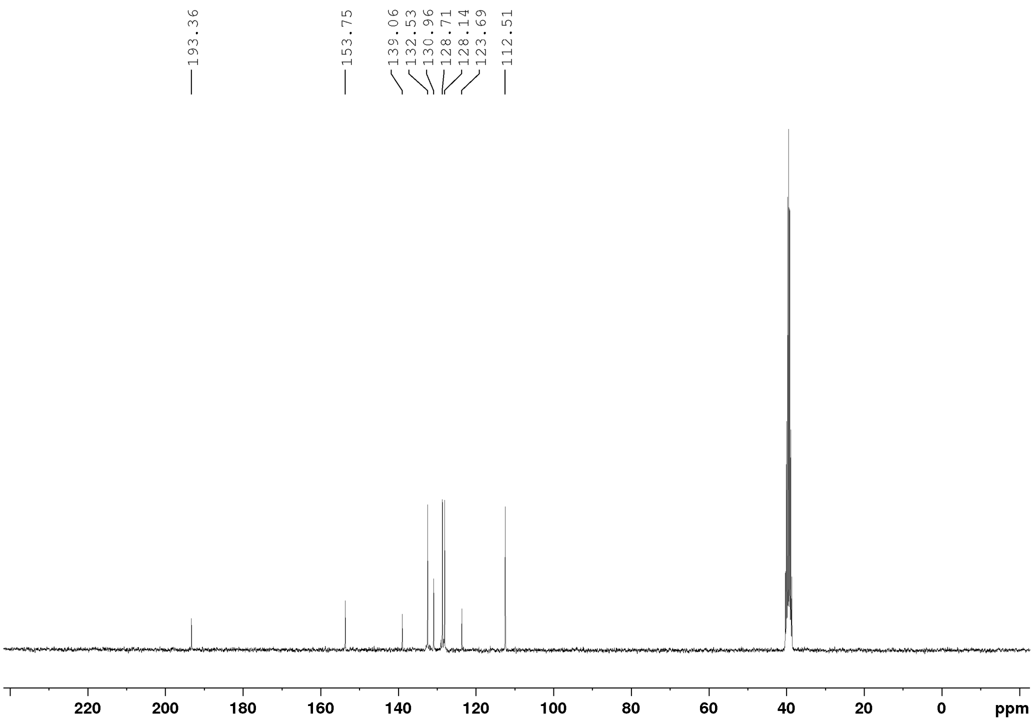

Supplementary Figure 47. ^{1}H NMR of 4-amino-α-methylstyrene, **2o** ($CDCl_3$, 300.1 MHz)

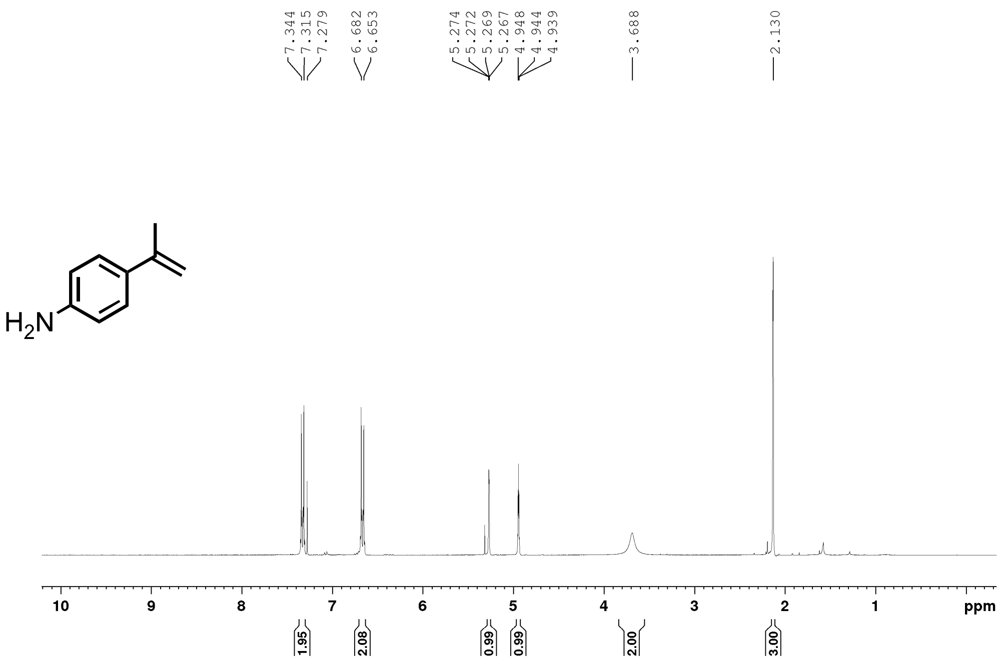

Supplementary Figure 48. $^{13}C\{^{1}H\}$ NMR of 4-amino-α-methylstyrene, **2o** ($CDCl_3$, 75.4 MHz)

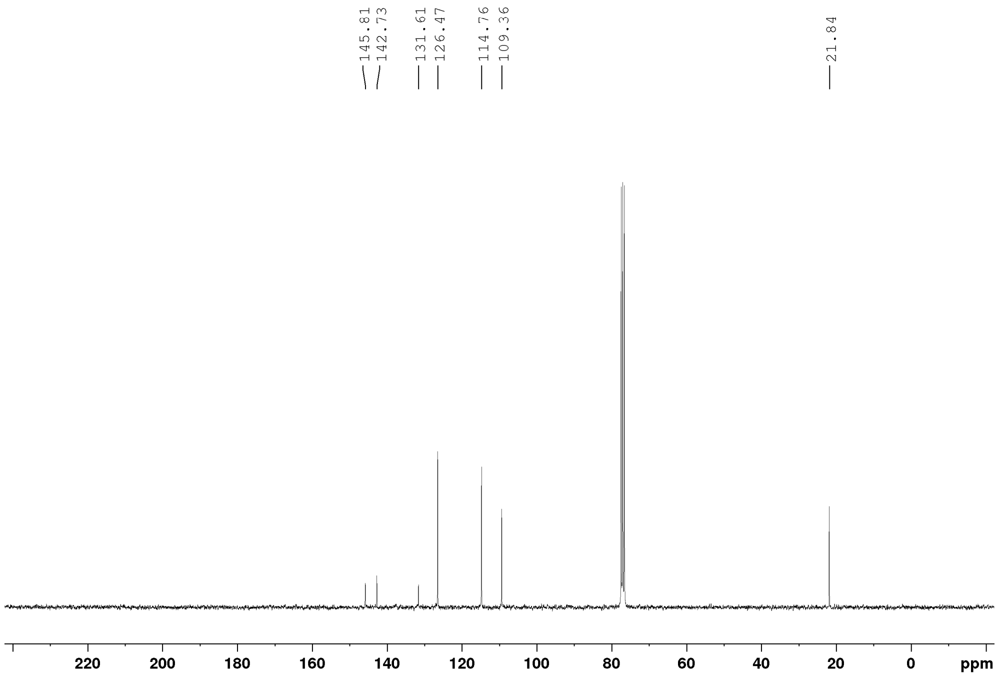

Supplementary Figure 49. ^{1}H NMR of 4-aminoisoquinoline, **2p** ($CDCl_3$, 500.1 MHz)

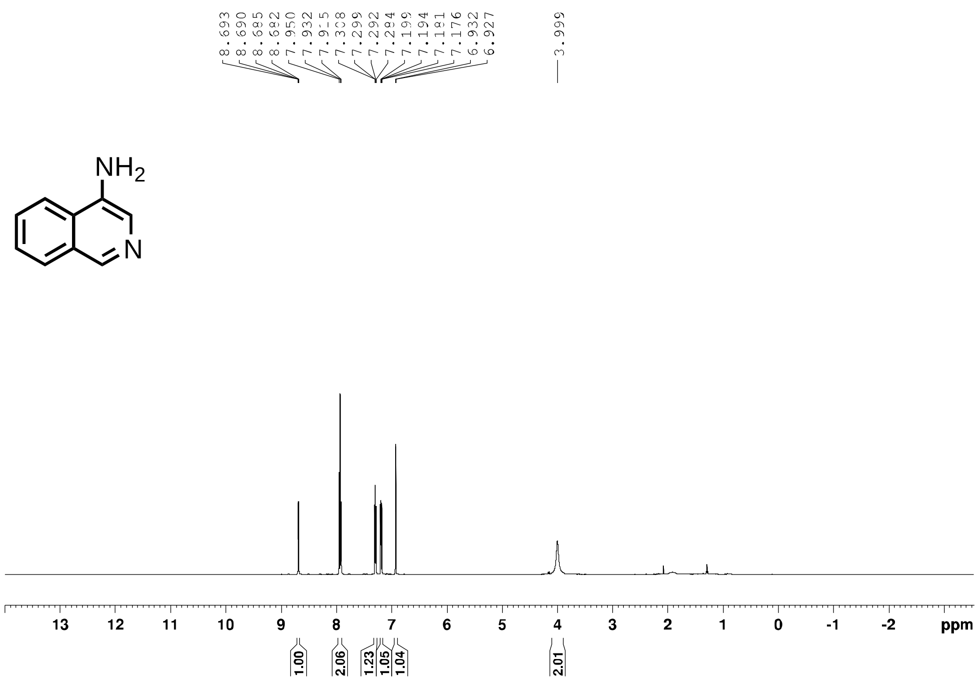

Supplementary Figure 50. $^{13}C\{^{1}H\}$ NMR of 4-aminoisoquinoline, **2p** ($CDCl_3$, 125.8 MHz)

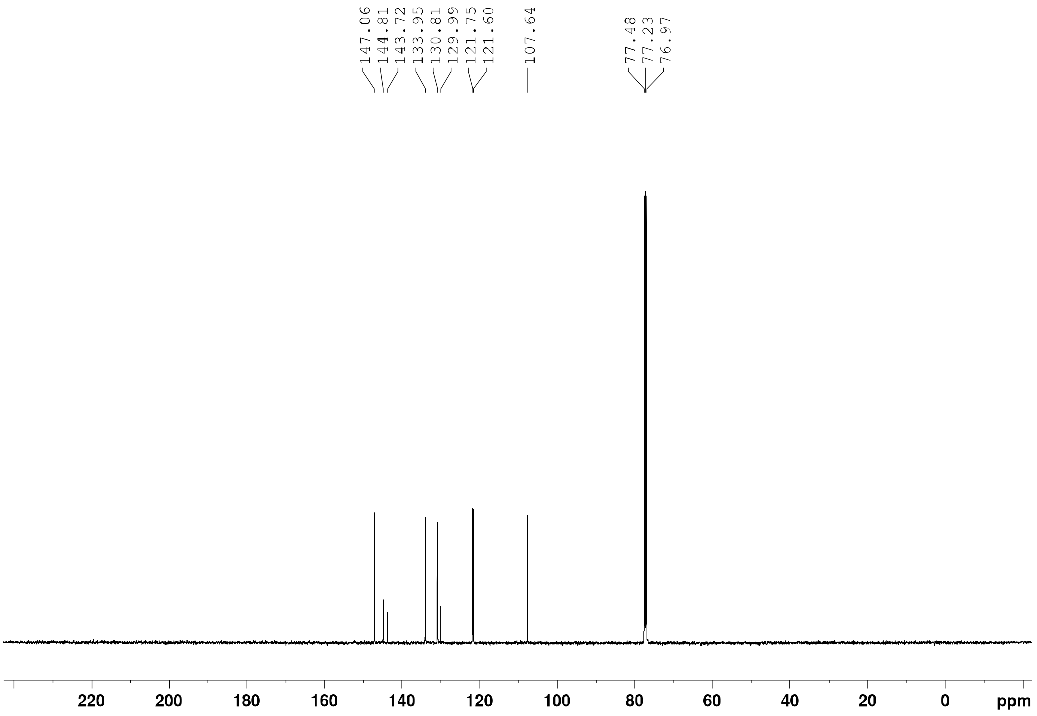

Supplementary Figure 51. ^{1}H NMR of 6-aminoquinoline, **2q** ($CDCl_3$, 500.1 MHz)

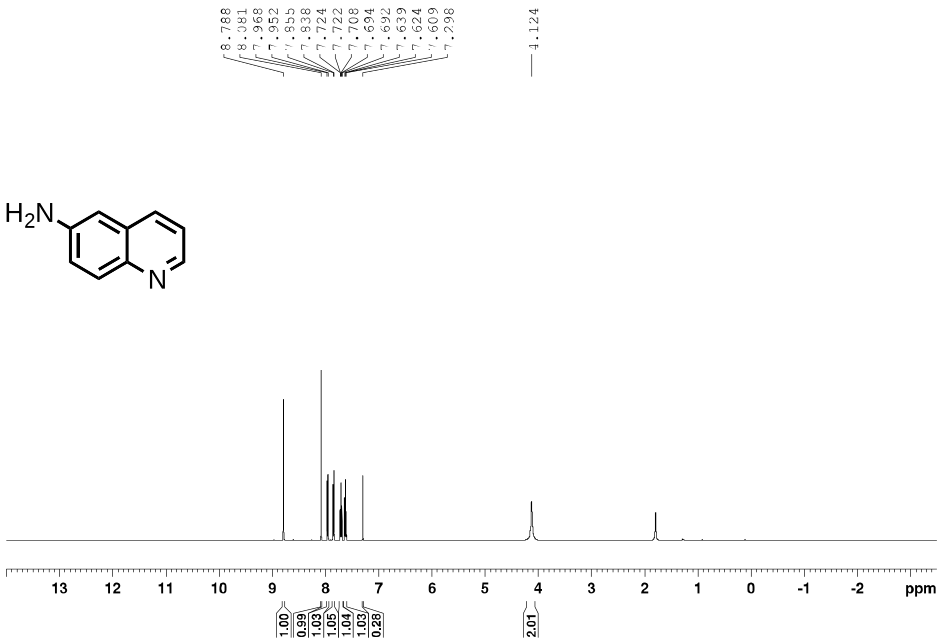

Supplementary Figure 52. $^{13}C\{^{1}H\}$ NMR of 6-aminoquinaldine, **2q** ($CDCl_3$, 125.8 MHz)

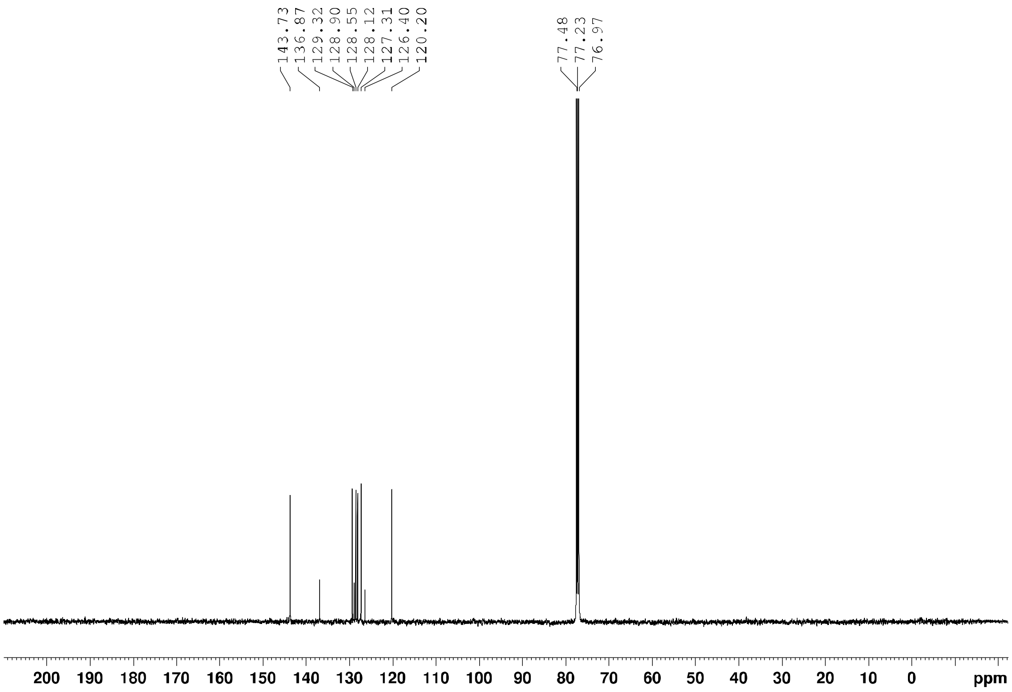

Supplementary Figure 53. ^{1}H NMR of Quinolin-8-ylamine, **2r** ($CDCl_3$, 500.1 MHz)

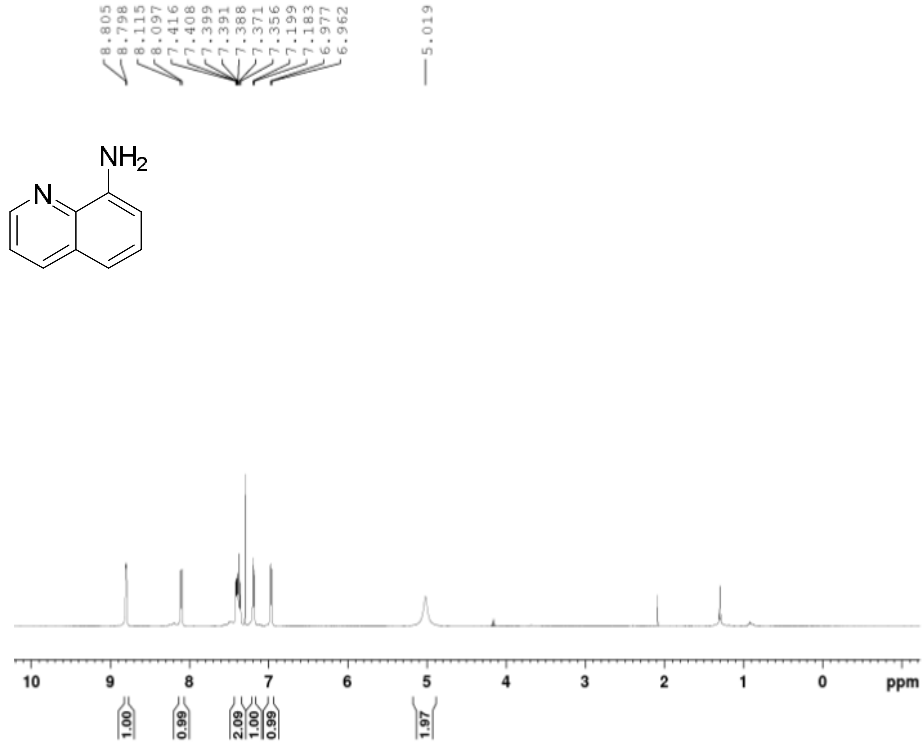

Supplementary Figure 54. $^{13}C\{^{1}H\}$ NMR of Quinolin-8-ylamine, **2r** ($CDCl_3$, 125.8 MHz)

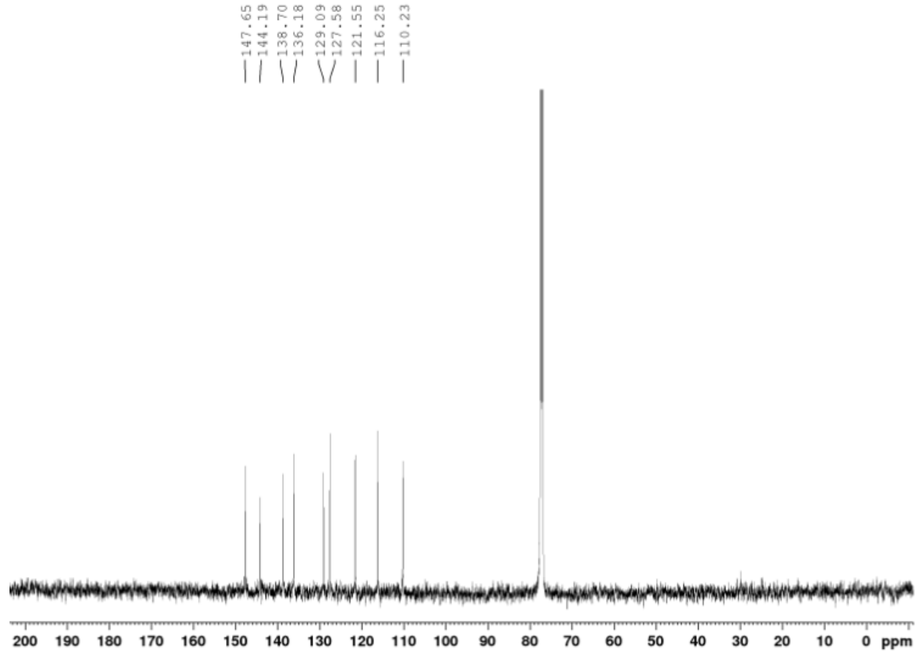

Supplementary Figure 55. ^{1}H NMR of 4-aminoquinaldine, **2s** ($CDCl_3$, 500.1 MHz)

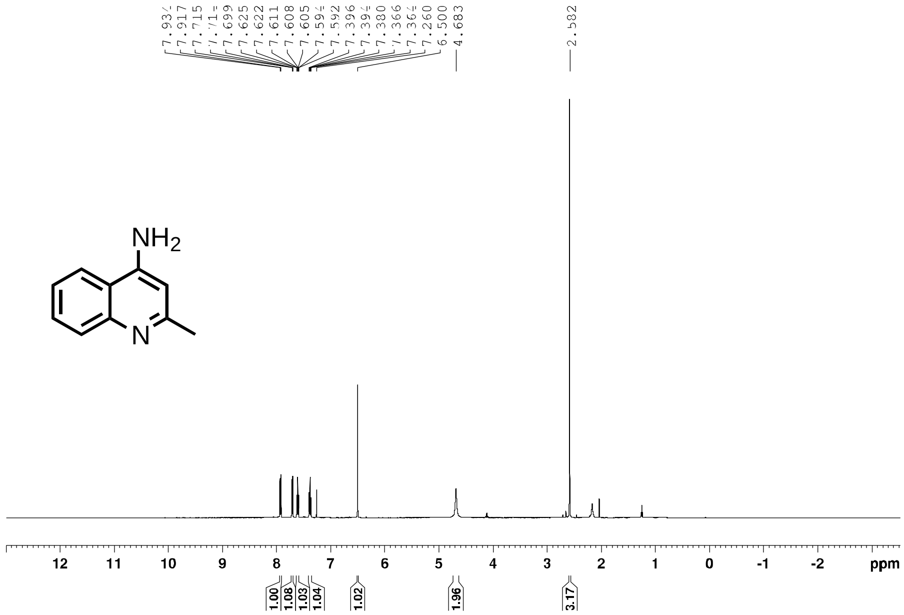

Supplementary Figure 56. $^{13}C\{^{1}H\}$ NMR of 4-aminoquinaldine, **2s** ($CDCl_3$, 125.8 MHz)

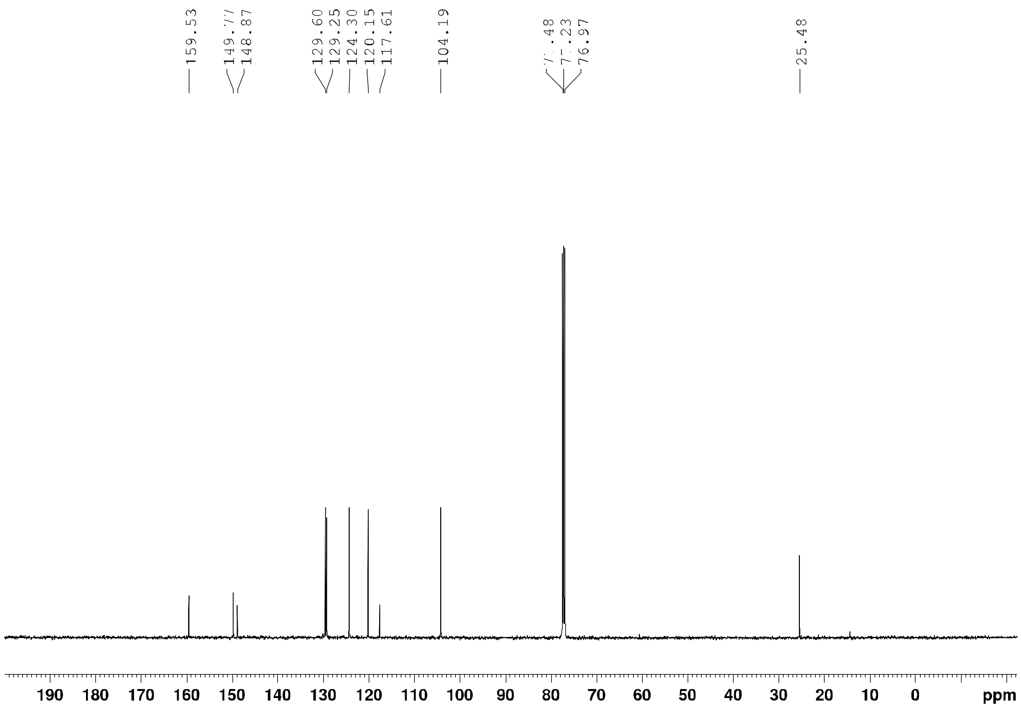

Supplementary Figure 57. ^{1}H NMR of 2-amino-4,6-dimethoxypyrimidine, **2t** ($CDCl_3$, 500.1 MHz)

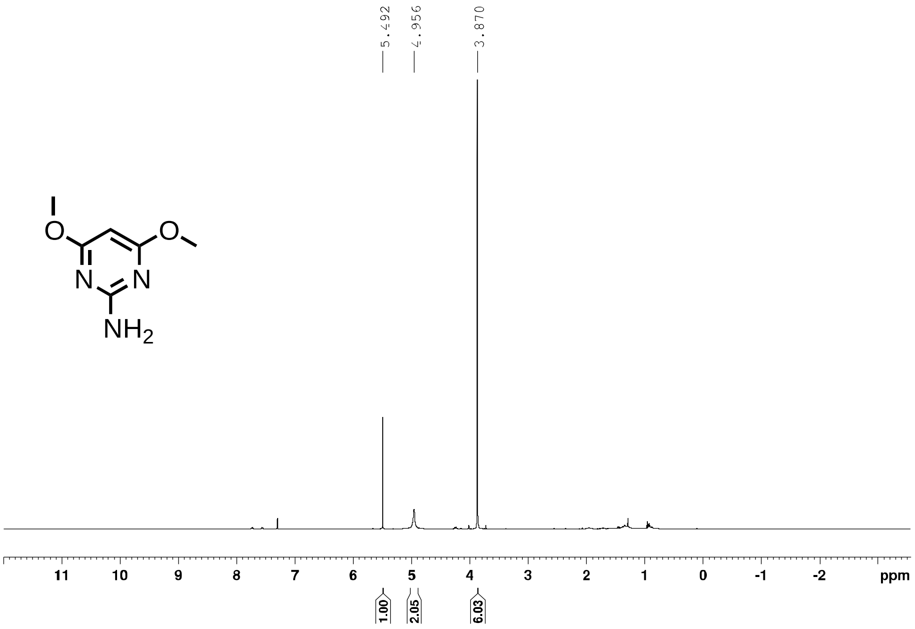

Supplementary Figure 58. ^{13}C{^{1}H} NMR of 2-amino-4,6-dimethoxypyrimidine, **2t** ($CDCl_3$, 125.8 MHz)

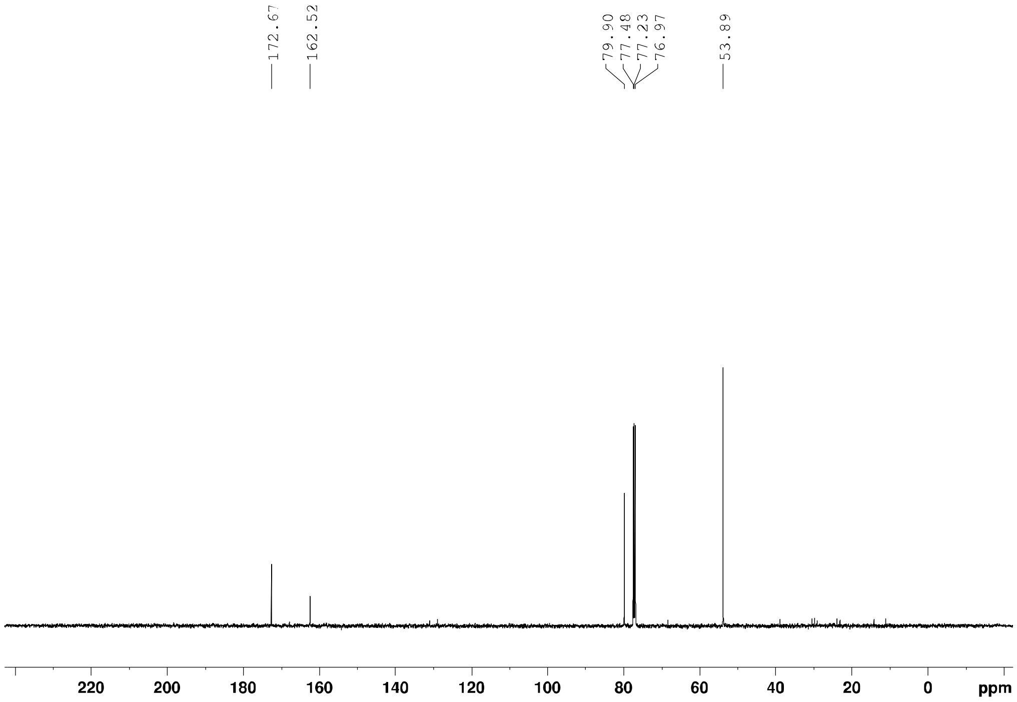

Supplementary Figure 59. ^{1}H NMR of 2-aminoquinazoline, **2u** ($CDCl_3$, 500.1 MHz)

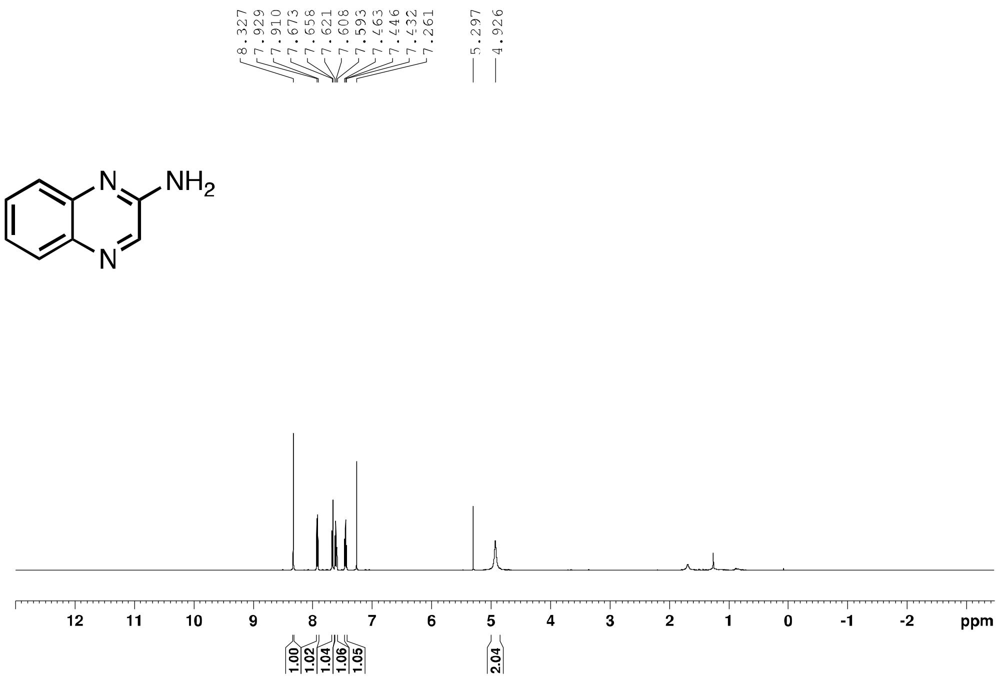

Supplementary Figure 60. $^{13}C\{^{1}H\}$ NMR of 2-aminoquinazoline, **2u** ($CDCl_3$, 125.8 MHz)

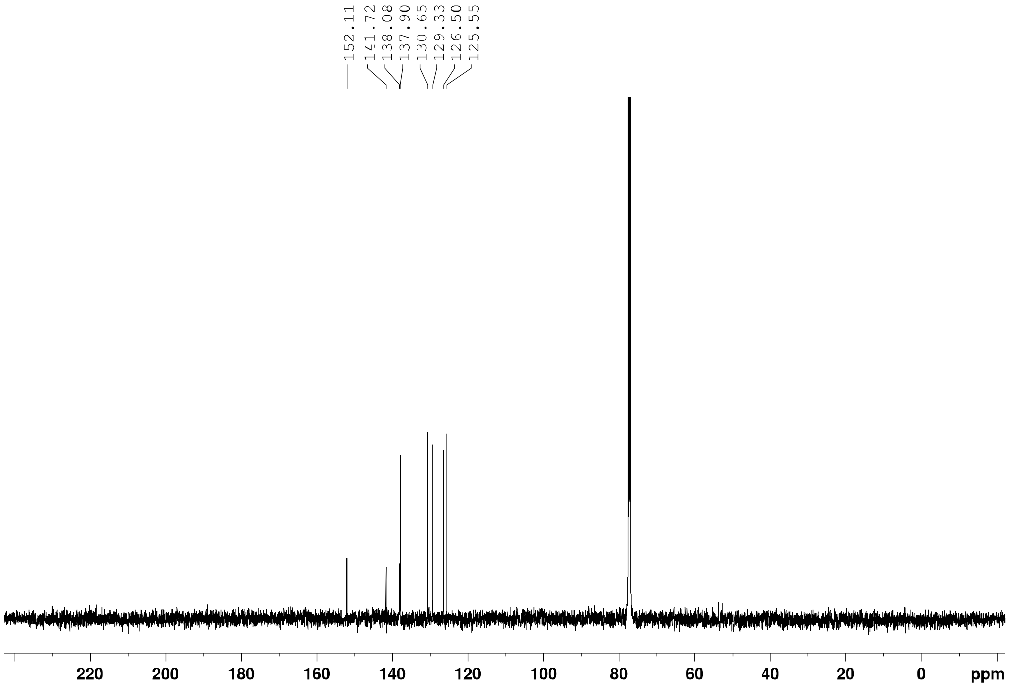

Supplementary Figure 61. ^{1}H NMR of 5-aminobenzothiophene, **2v** ($CDCl_3$, 500.1 MHz)

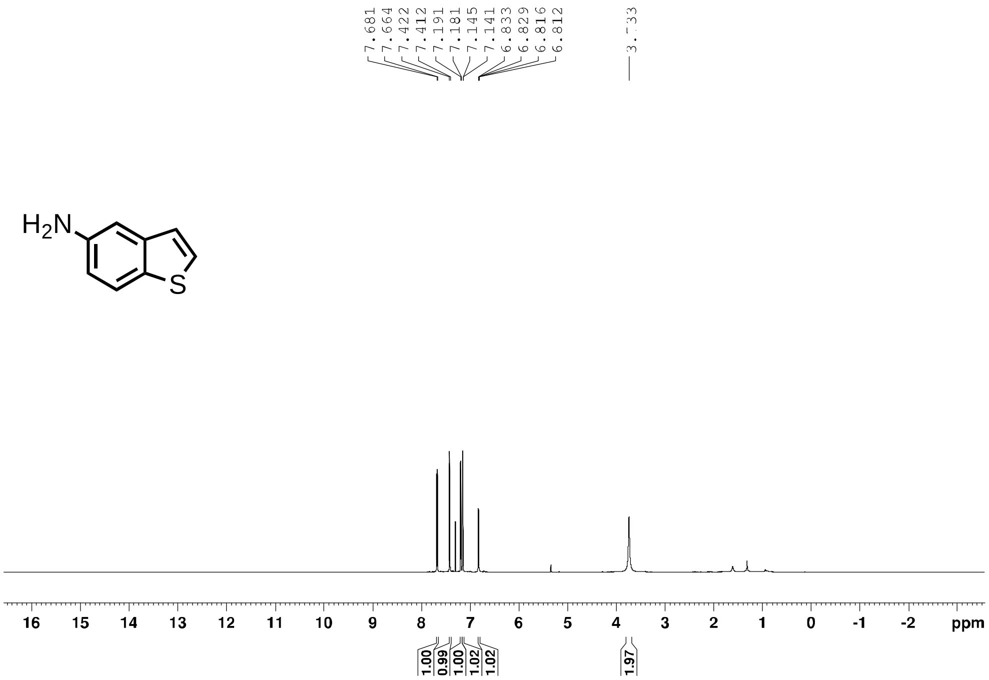

Supplementary Figure 62. ^{13}C{^{1}H} NMR of 5-aminobenzothiophene, **2v** ($CDCl_3$, 125.8 MHz)

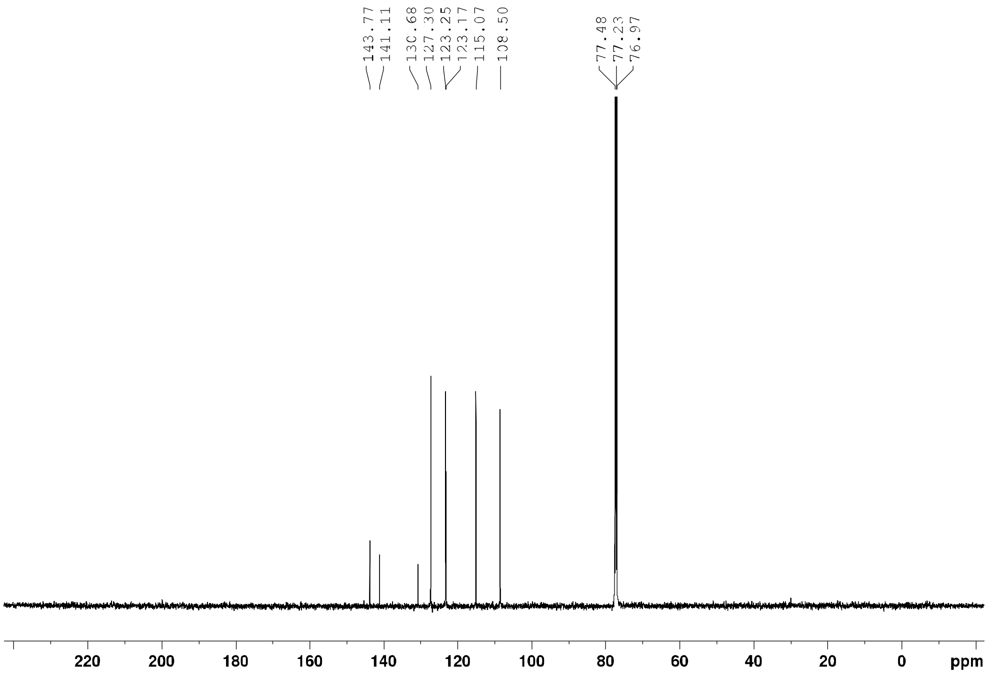

Supplementary Figure 63. ^{1}H NMR of 5-amino-2-methylbenzothiazole, **2w** ($CDCl_3$, 500.1 MHz)

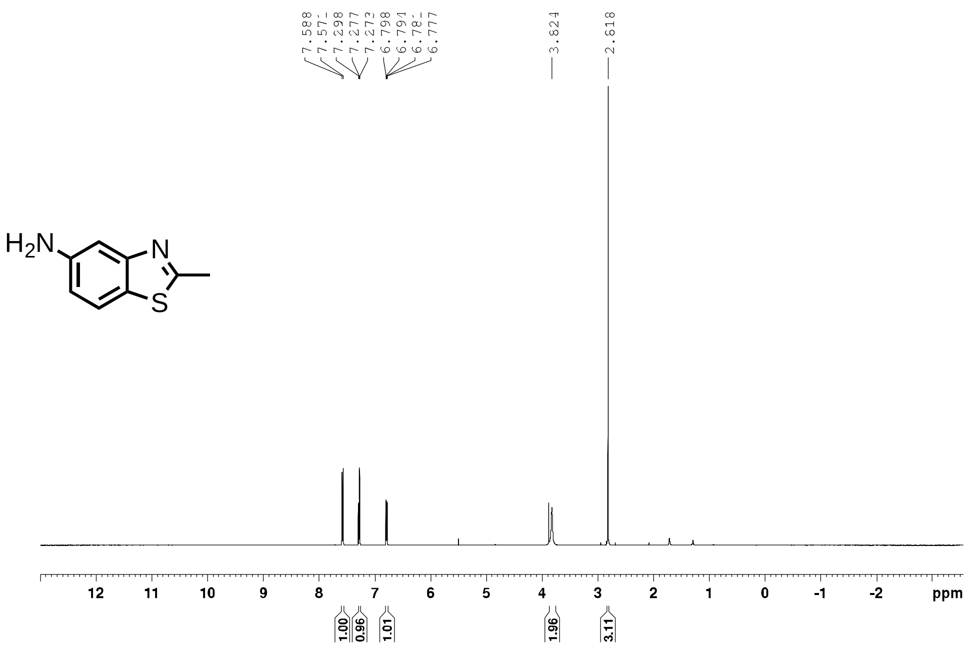

Supplementary Figure 64. ^{13}C{^{1}H} NMR of 5-amino-2-methylbenzothiazole, **2w** ($CDCl_3$, 125.8 MHz)

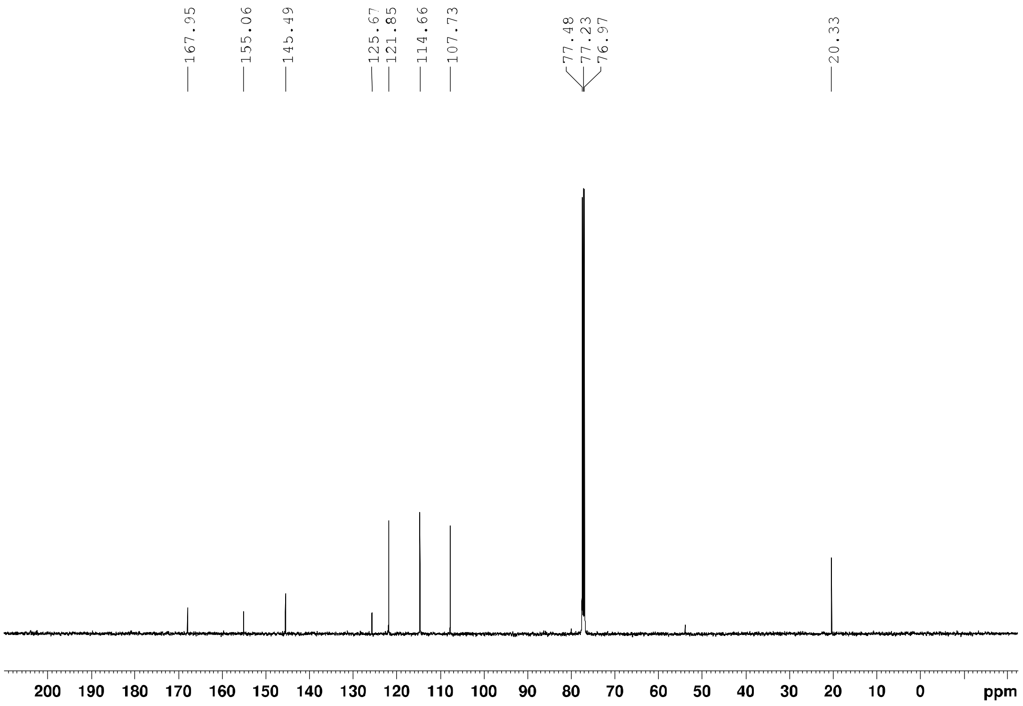

Supplementary Figure 65. ^{1}H NMR of 2-amino-6,7-dimethoxyquinazoline, **2x** ($CDCl_3$, 500.1 MHz)

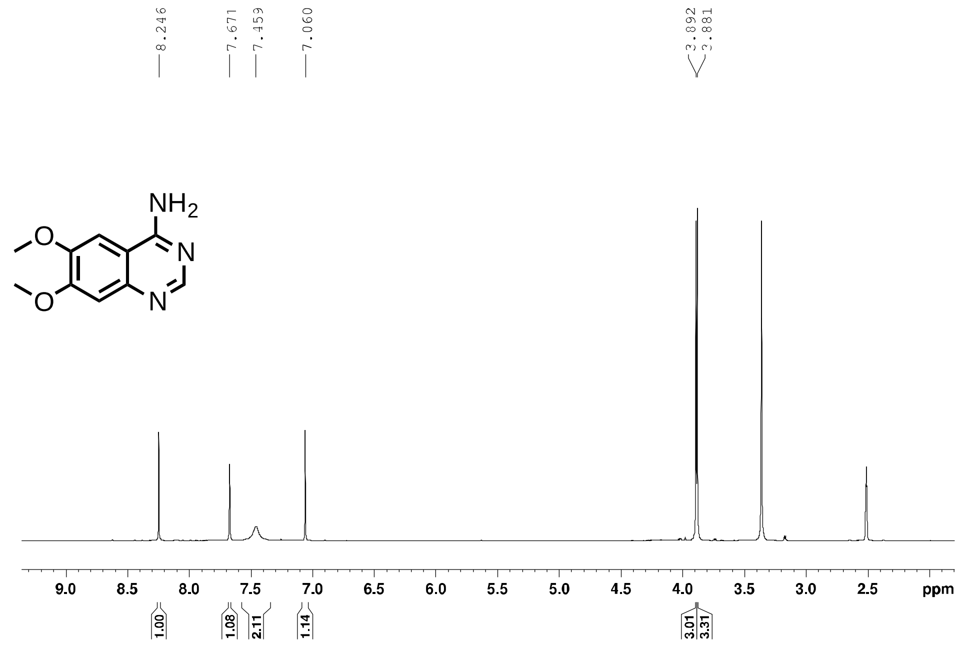

Supplementary Figure 66. $^{13}C\{^{1}H\}$ NMR of 2-amino-6,7-dimethoxyquinazoline, **2x** ($CDCl_3$, 125.8 MHz)

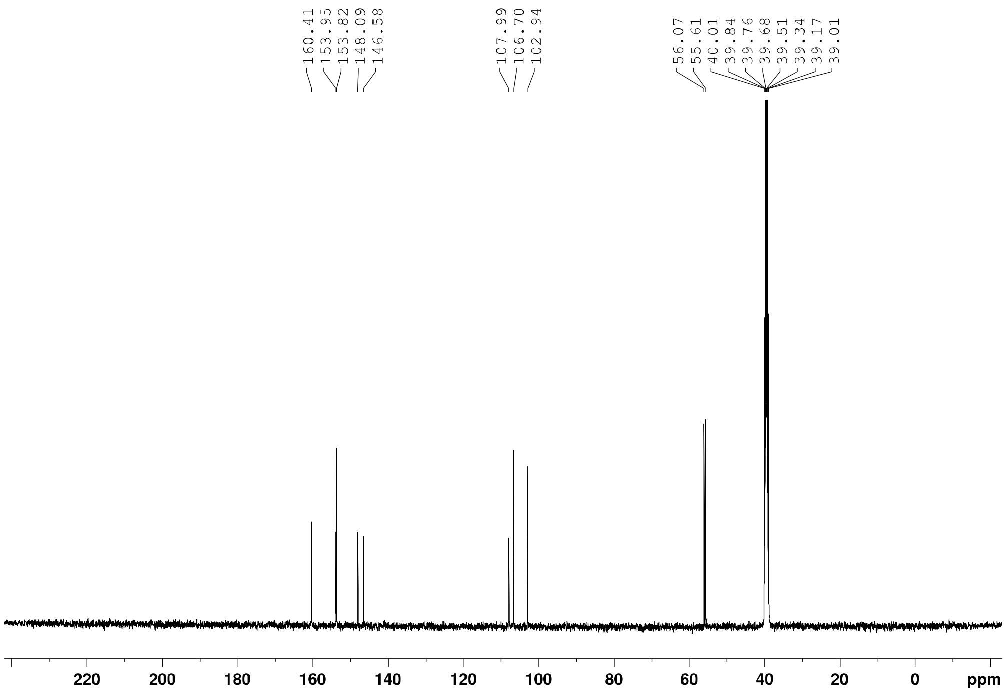

Supplementary Figure 67. ^{1}H NMR of 9-amino-1,2,3,4-tetrahydroacridine, **2y** ($CDCl_3$, 500.1 MHz)

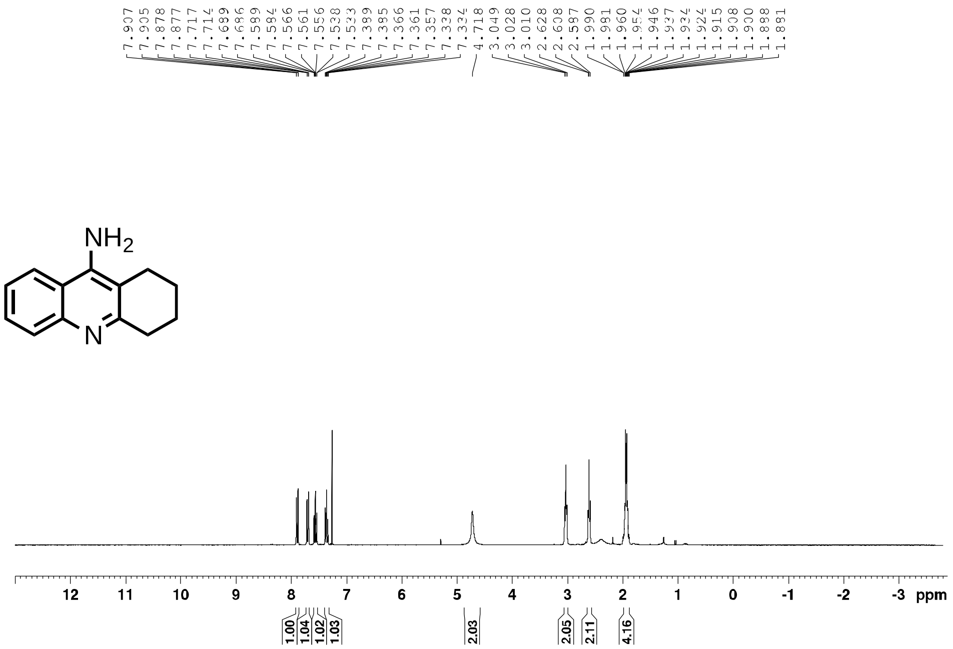

Supplementary Figure 68. $^{13}C\{^{1}H\}$ NMR of 9-amino-1,2,3,4-tetrahydroacridine, **2y** ($CDCl_3$, 125.8 MHz)

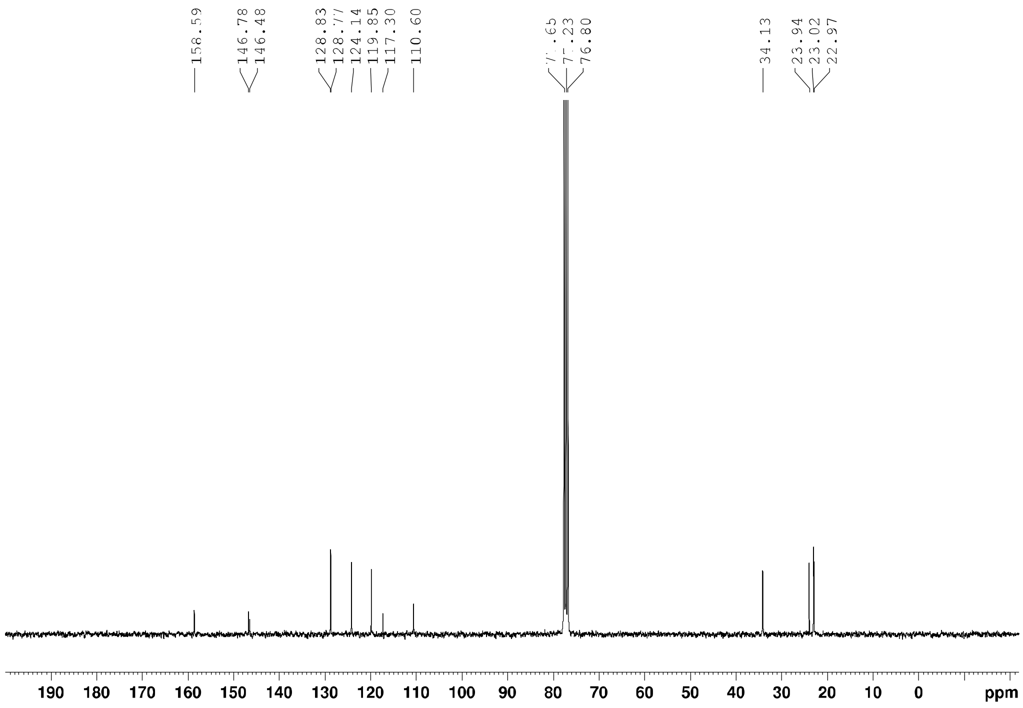

Supplementary Figure 69. ^{1}H NMR of 3-aminoestrone, **2z** ($CDCl_3$, 500.1 MHz)

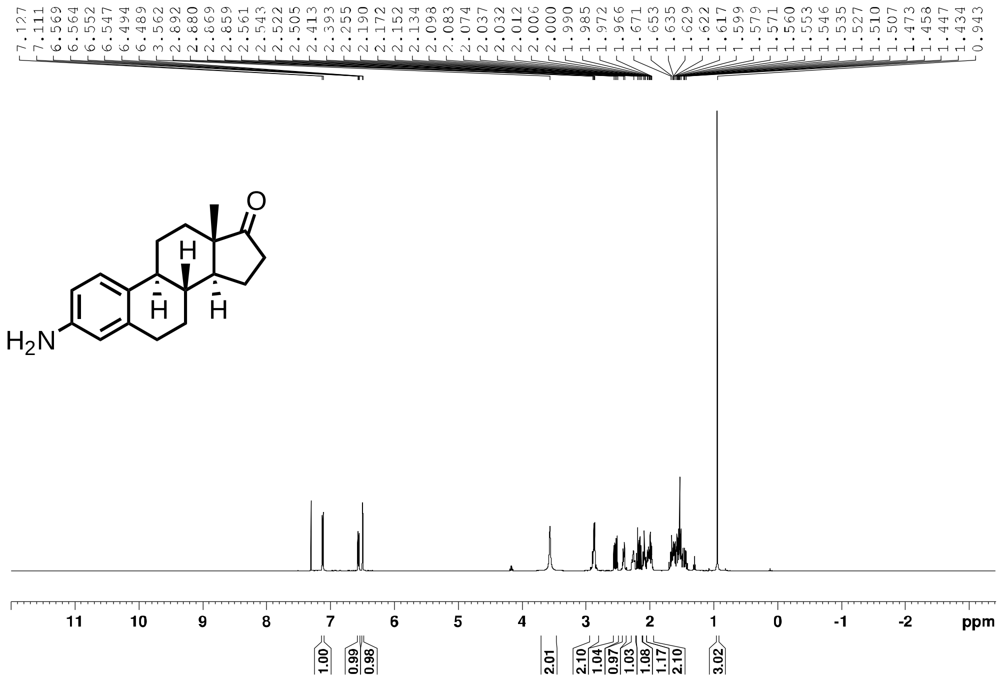

Supplementary Figure 70. $^{13}C\{^1H\}$ NMR of 3-aminoestrone, **2z** ($CDCl_3$, 125.8 MHz)

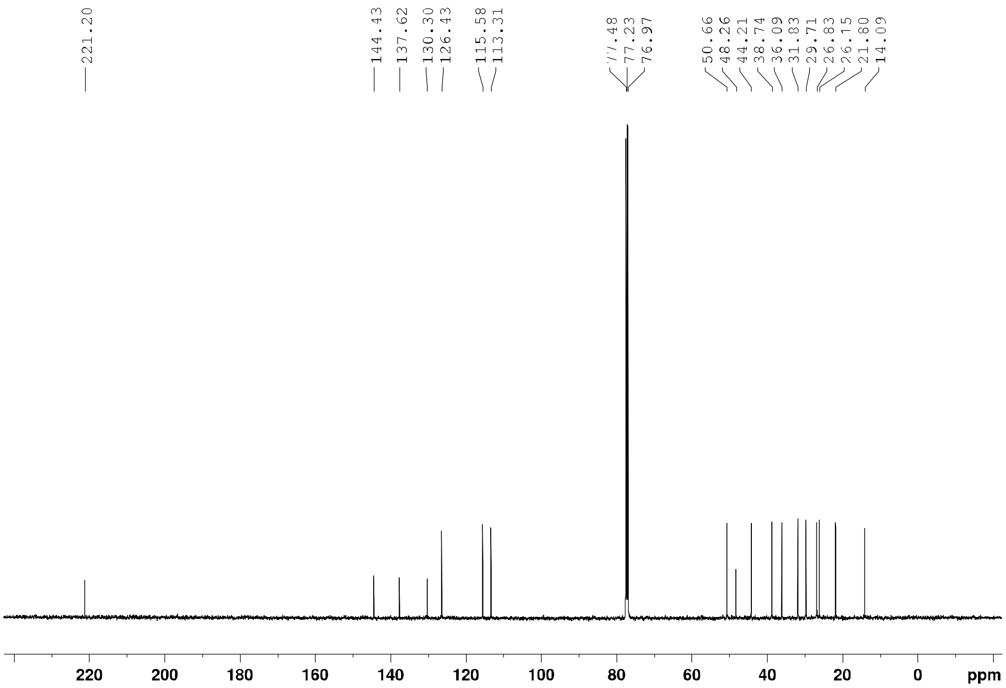

Supplementary Figure 71. ^{1}H NMR of *N*-(1-naphthyl)-Octylamine, **4a** ($CDCl_3$, 500.1 MHz)

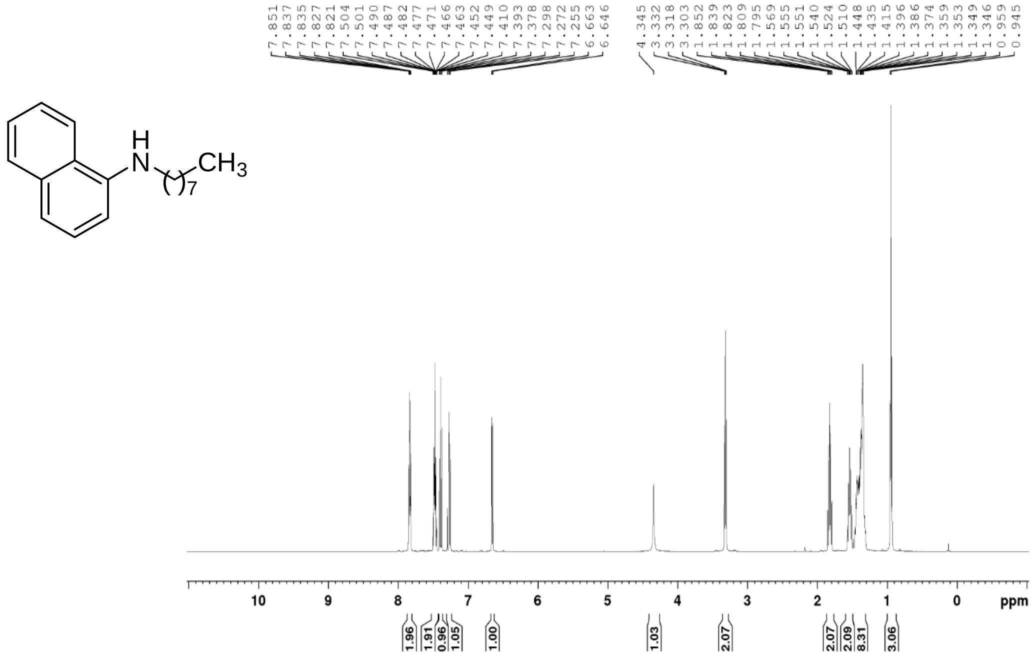

Supplementary Figure 72. $^{13}C\{^{1}H\}$ NMR of *N*-(1-naphthyl)-Octylamine, **4a** ($CDCl_3$, 125.8 MHz)

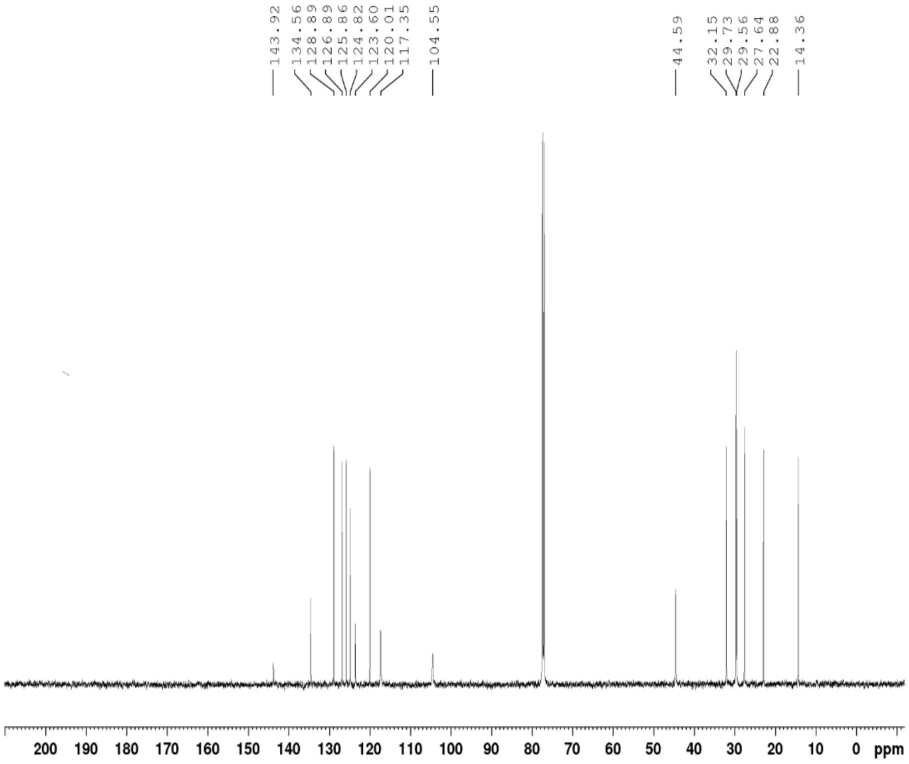

Supplementary Figure 73. ^{1}H NMR of *N*-methylnaphthalen-1-amine, **4b** ($CDCl_3$, 300 MHz)

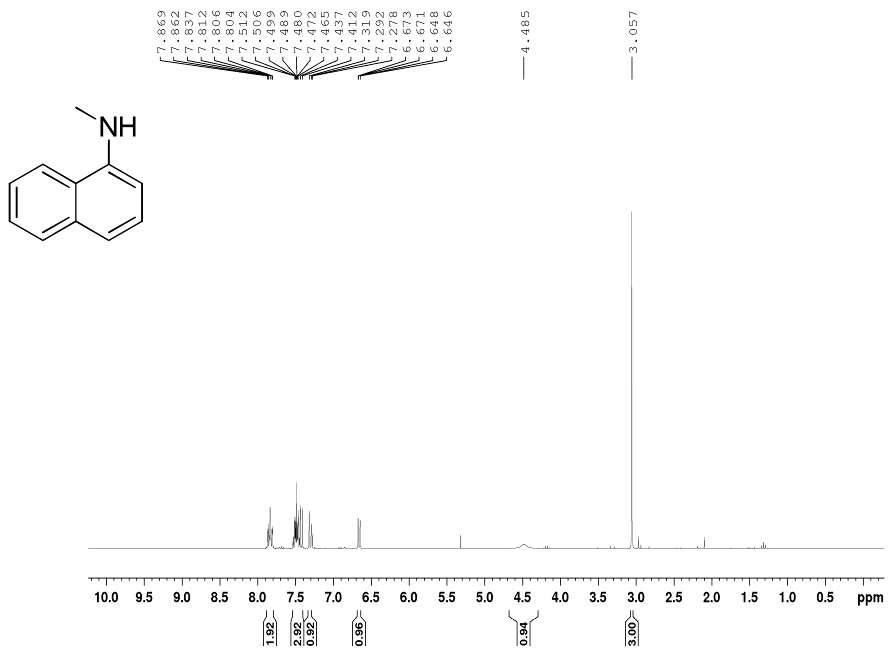

Supplementary Figure 74. $^{13}C\{^{1}H\}$ NMR of *N*-methylnaphthalen-1-amine, **4b** ($CDCl_3$, 75.5 MHz)

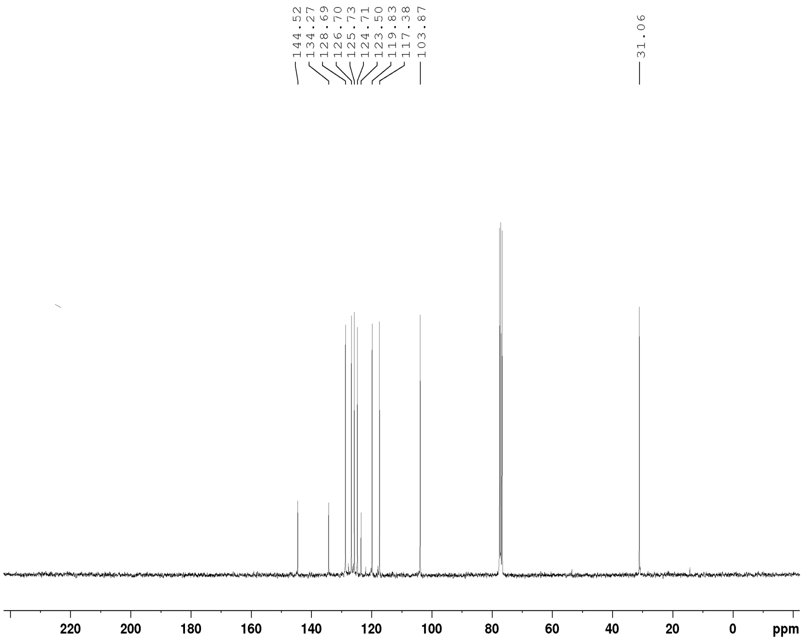

Supplementary Figure 75. ^{1}H NMR of *N*-ethylnaphthalen-1-amine, **4c** ($CDCl_3$, 300 MHz)

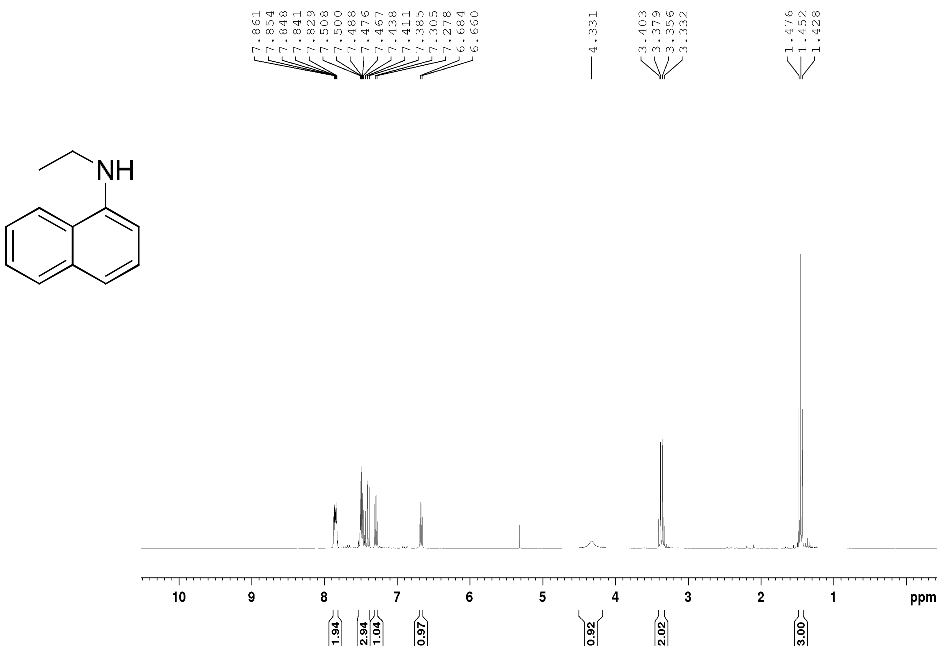

Supplementary Figure 76. $^{13}C\{^{1}H\}$ NMR of *N*-ethylnaphthalen-1-amine, **4c** ($CDCl_3$, 75.4 MHz)

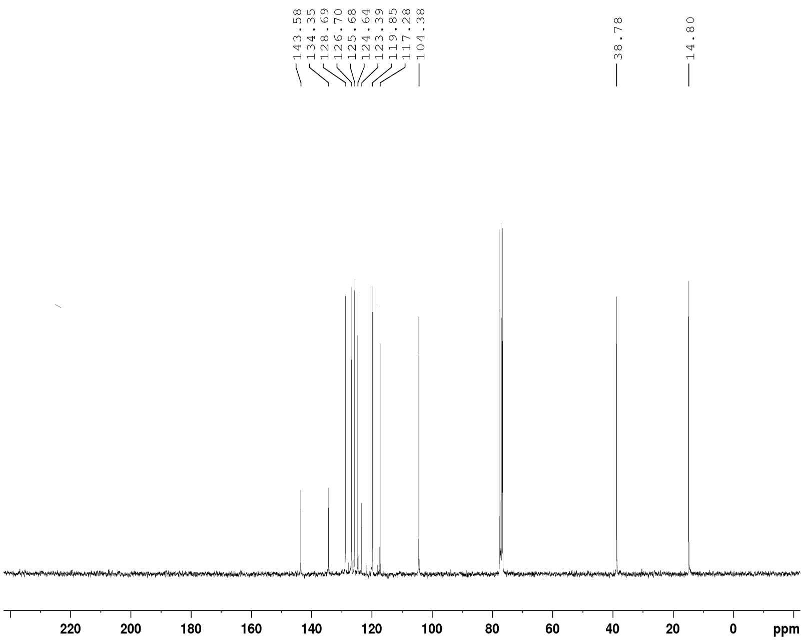

Supplementary Figure 77. ^{1}H NMR of 1-(*N*,*N*-dimethylamino)naphthalene, **4d** ($CDCl_3$, 300.1 MHz)

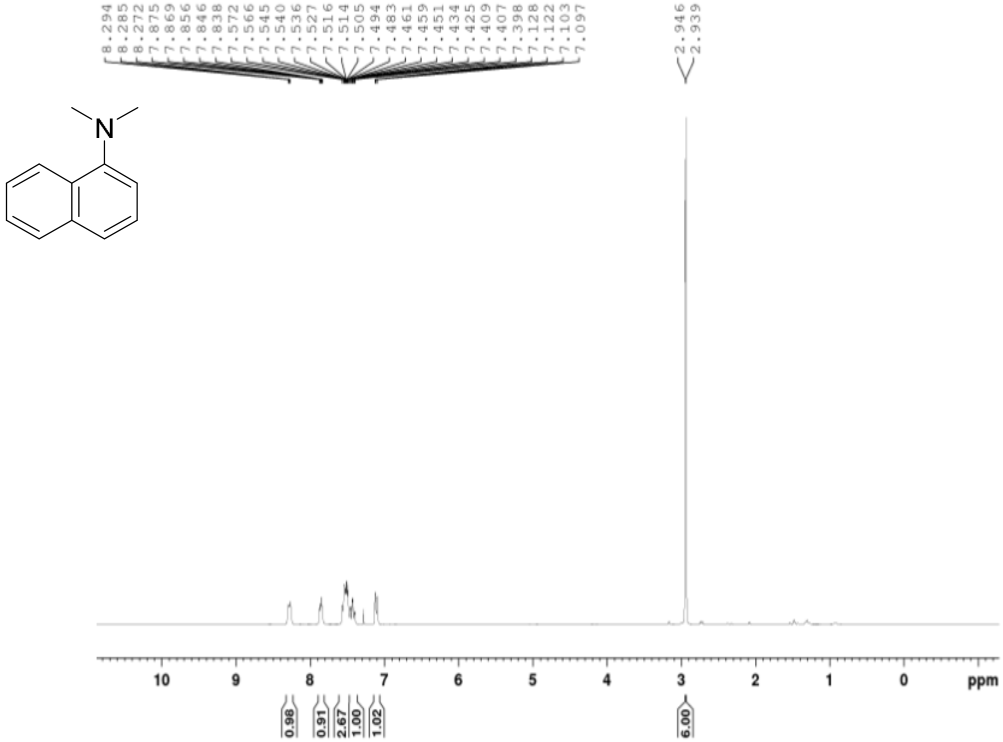

Supplementary Figure 78. $^{13}C\{^{1}H\}$ NMR of 1-(*N*,*N*-dimethylamino)naphthalene, **4d** ($CDCl_3$, 125.8 MHz)

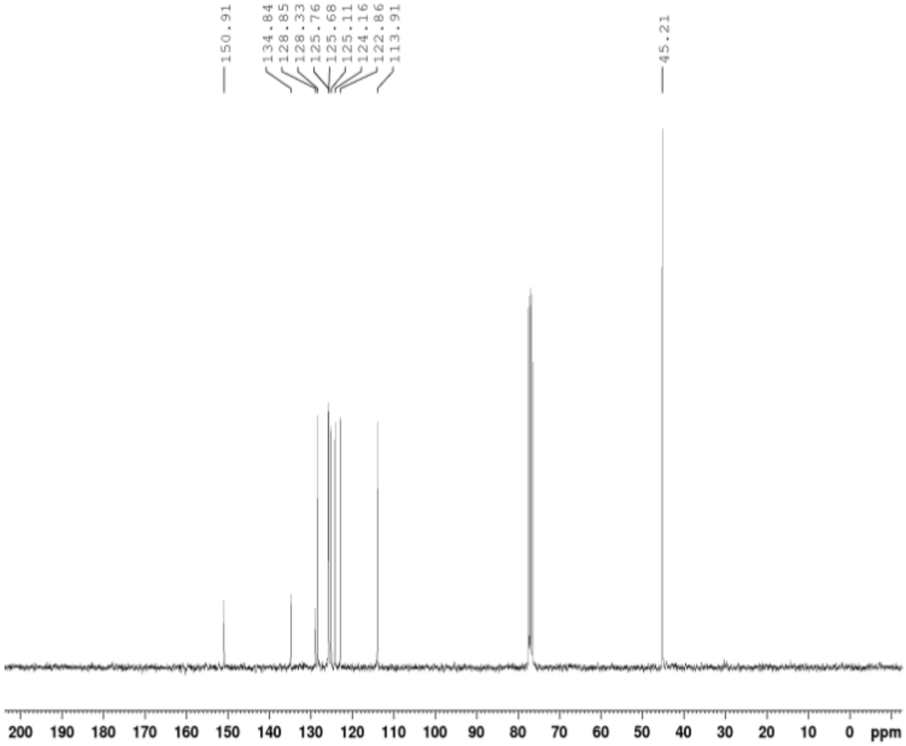

Supplementary Figure 79. ^{1}H NMR of 4-Methoxy-*N*-octylaniline, **4e** ($CDCl_3$, 300.1 MHz)

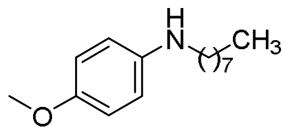

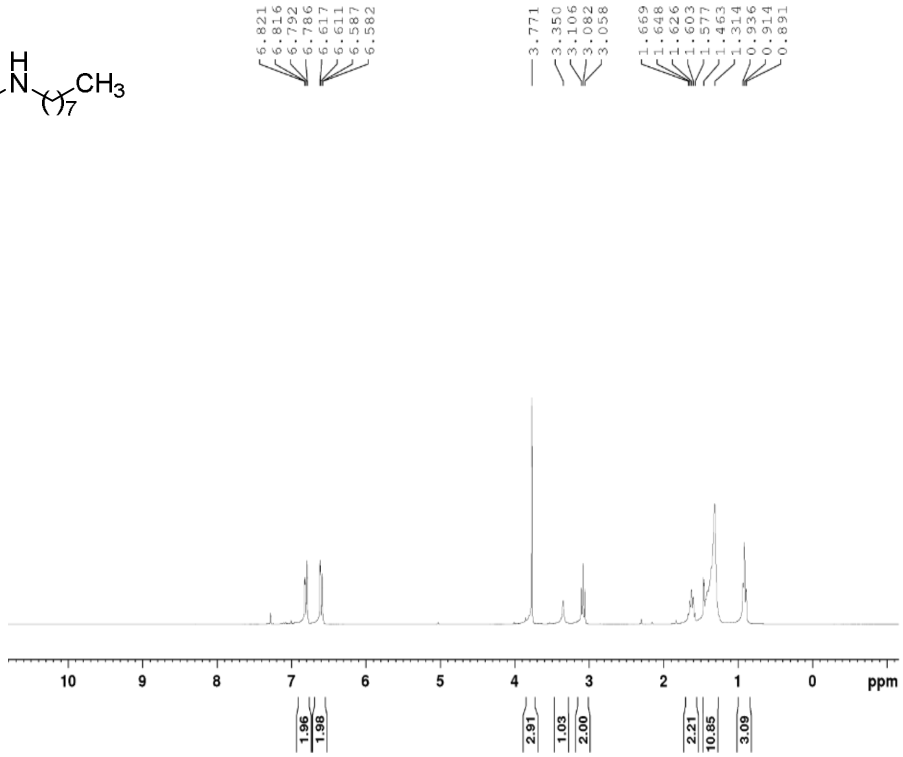

Supplementary Figure 80. $^{13}C\{^{1}H\}$ NMR of 4-Methoxy-*N*-octylaniline, **4e** ($CDCl_3$, 125.8 MHz)

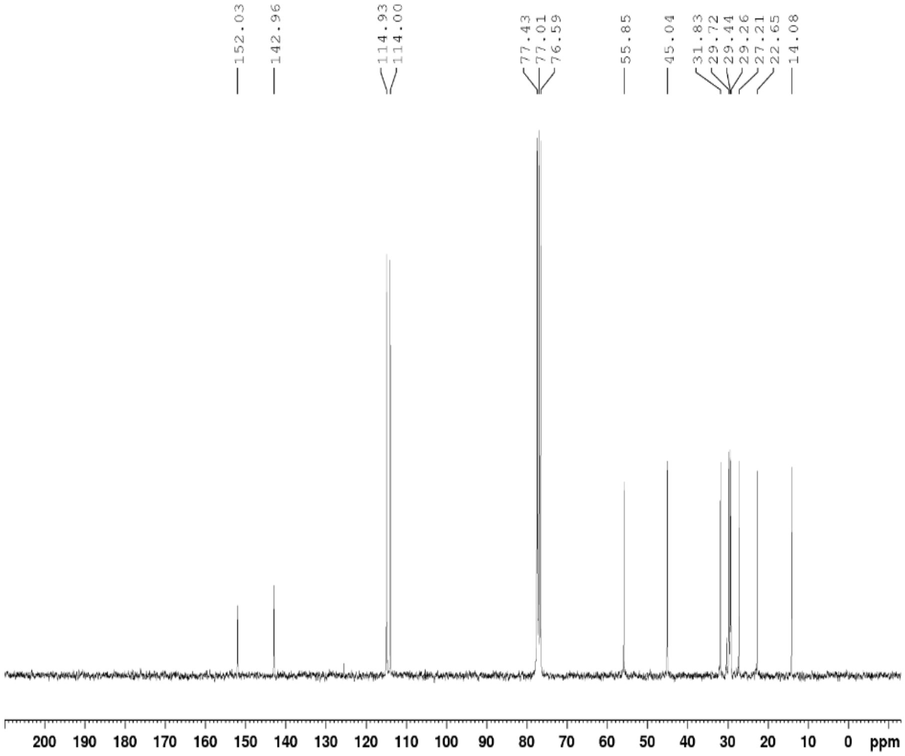

Supplementary Figure 81. ^{1}H NMR of *N*-Octyl-4-(trifluoromethyl)anilinie, **4f** ($CDCl_3$, 500.1 MHz)

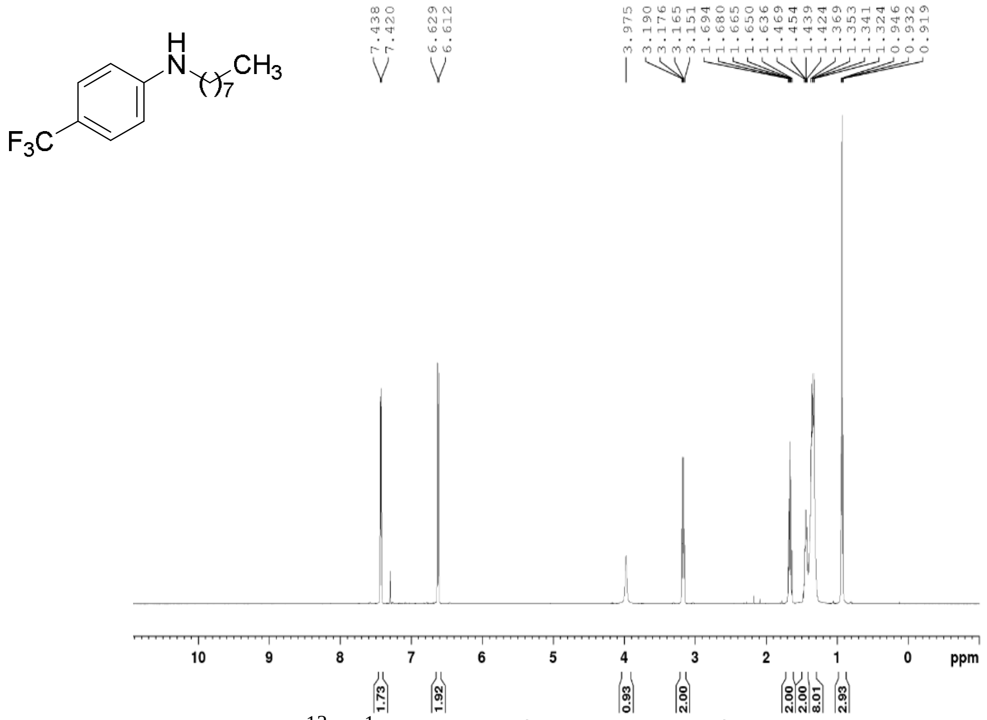

Supplementary Figure 82. $^{13}C\{^{1}H\}$ NMR of *N*-Octyl-4-(trifluoromethyl)anilinie, **4f** ($CDCl_3$, 125.8 MHz)

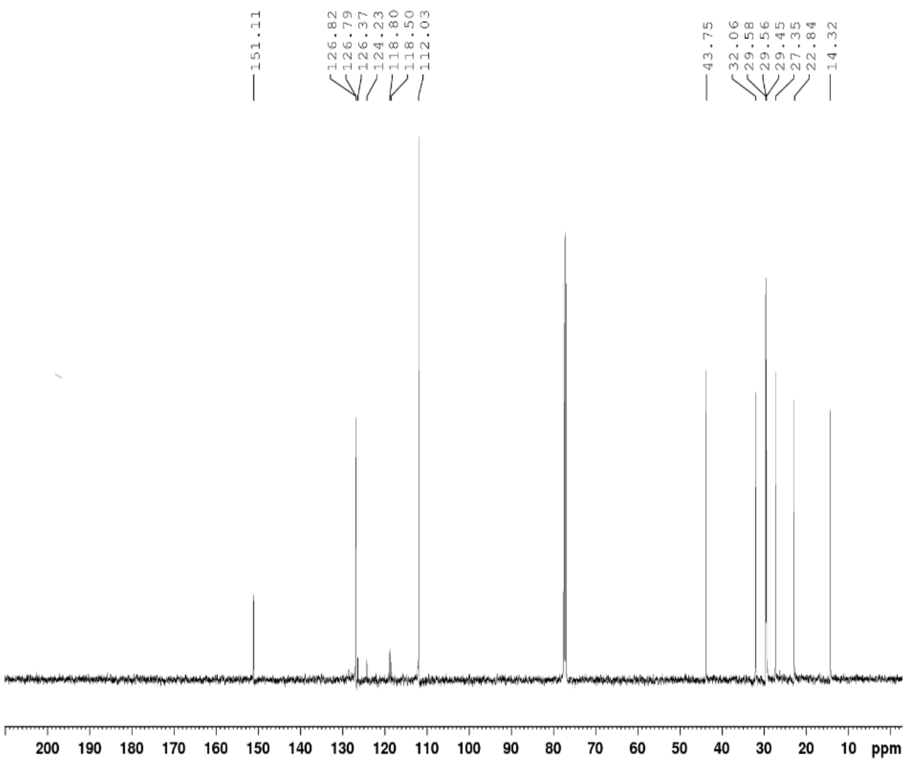

Supplementary Figure 83. ^{1}H NMR of 4-(Octylamino)benzonitrile, **4g** ($CDCl_3$, 500.1 MHz)

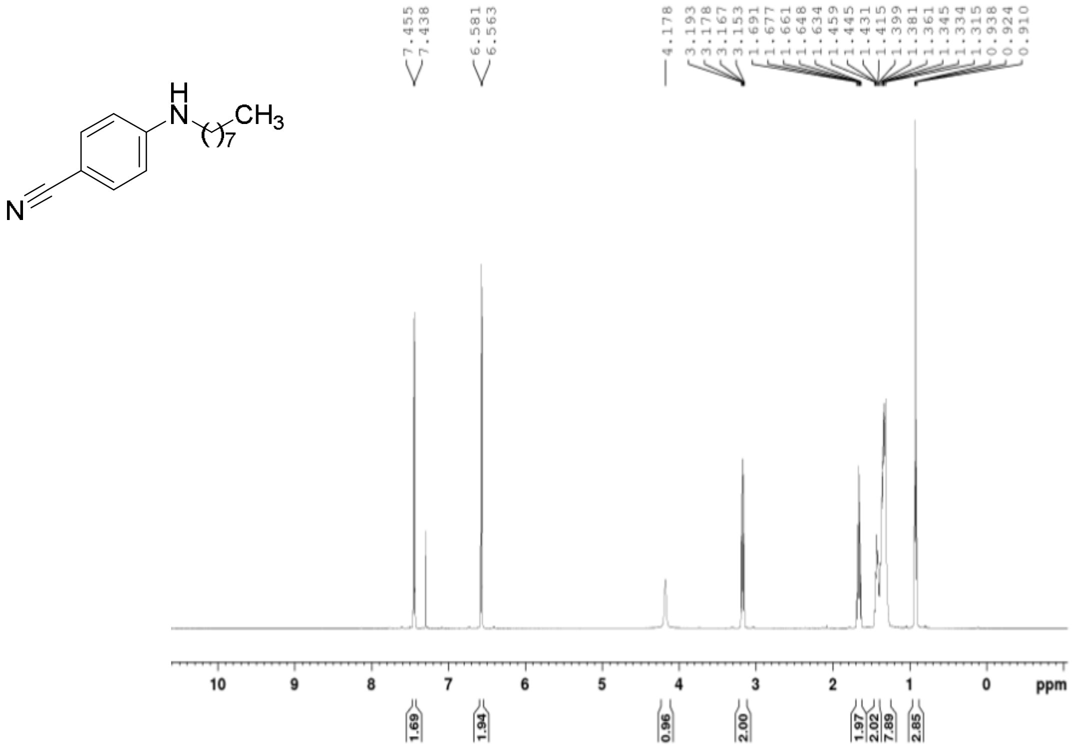

Supplementary Figure 84. $^{13}C\{^{1}H\}$ NMR of 4-(Octylamino)benzonitrile, **4g** ($CDCl_3$, 125.8 MHz)

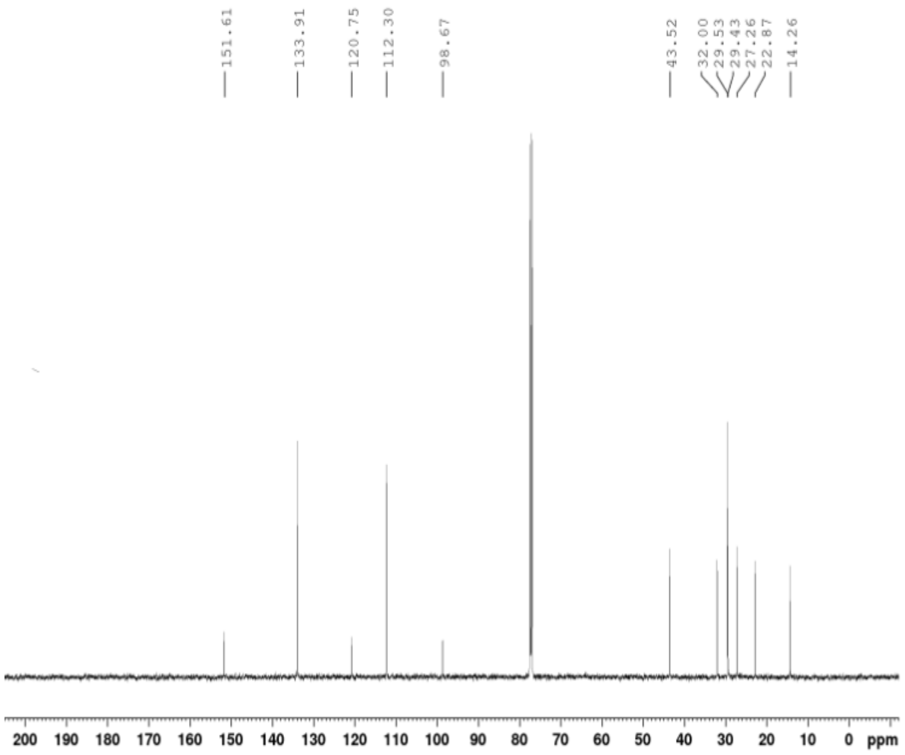

Supplementary Figure 85. ^{1}H NMR of 2,5-Dimethyl-*N*-octylaniline, **4h** ($CDCl_3$, 500.1 MHz)

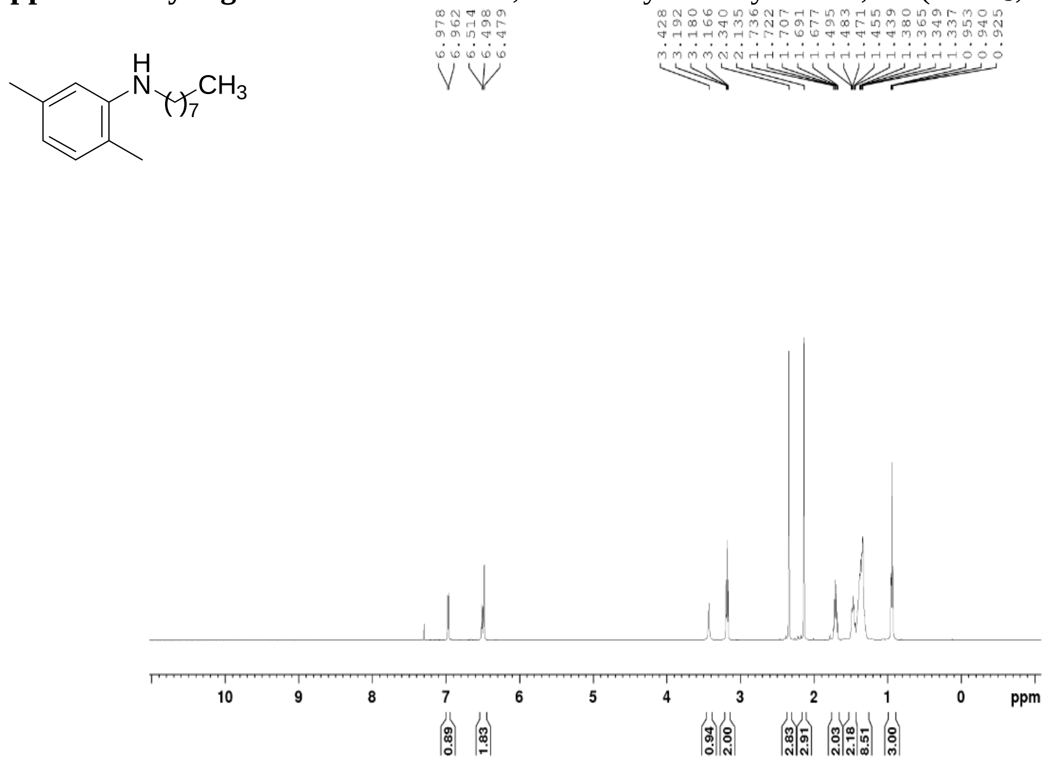

Supplementary Figure 86. $^{13}C\{^{1}H\}$ NMR of 2,5-Dimethyl-*N*-octylaniline, **4h** ($CDCl_3$, 125.8 MHz)

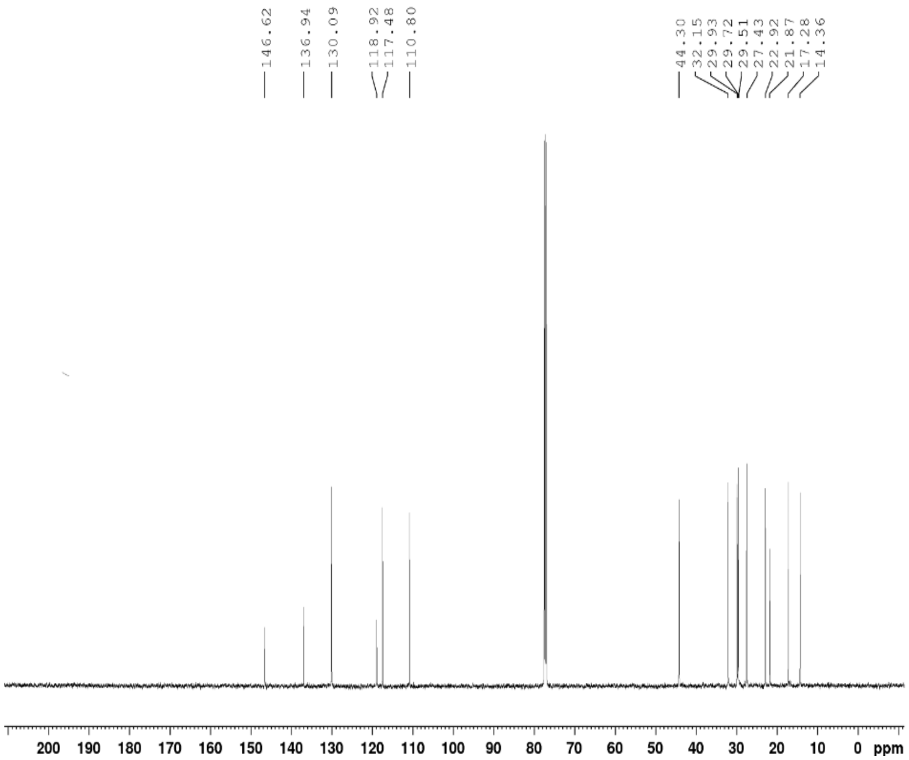

Supplementary Figure 87. ^{1}H NMR of 2’,6’-dimethoxy-*N*-octylamine, **4i** ($CDCl_3$, 300.1 MHz)

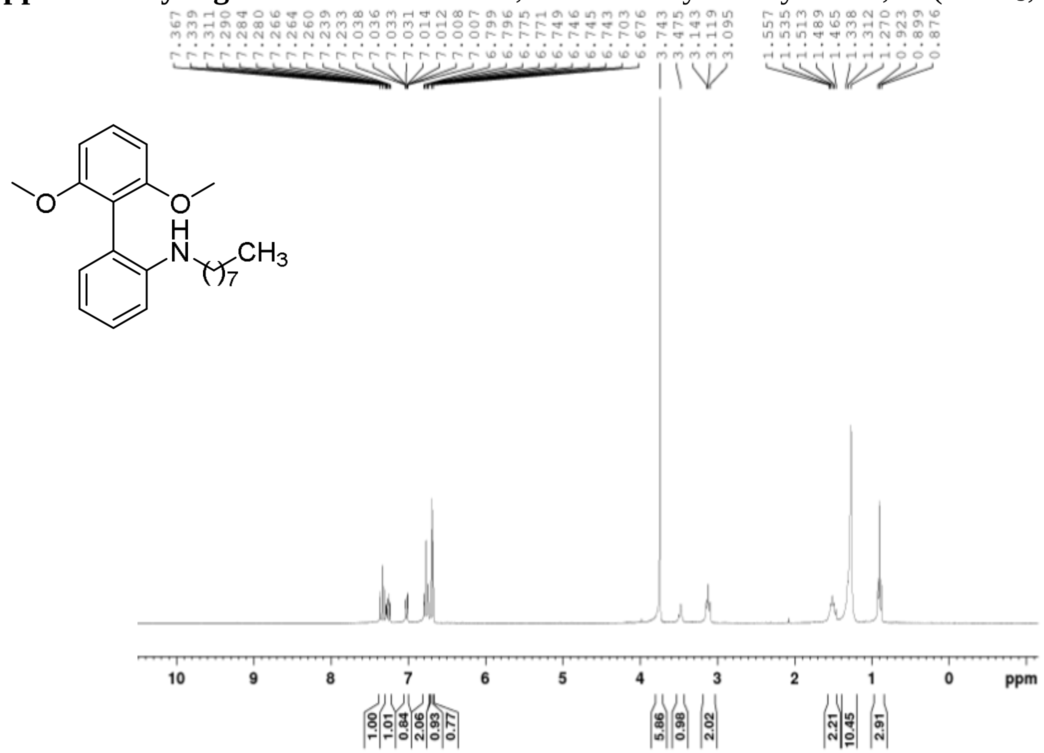

Supplementary Figure 88. ^{13}C{^{1}H} NMR of 2’,6’-dimethoxy-*N*-octylamine, **4i** ($CDCl_3$, 75.5 MHz)

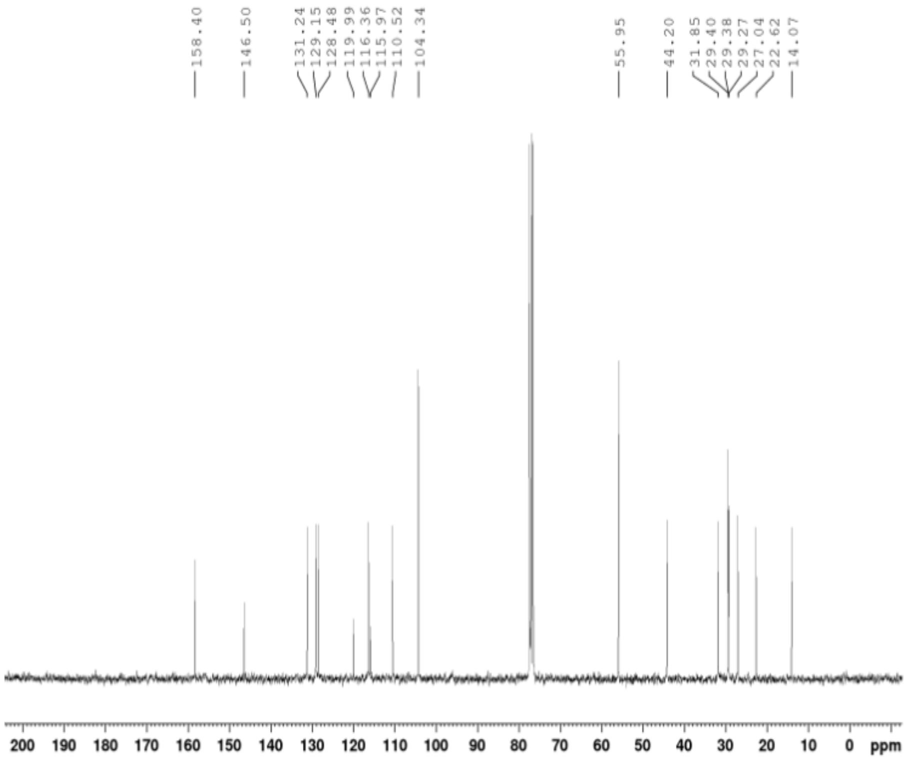

Supplementary Figure 89. ^{1}H NMR of Methyl-(2-methyl-quinolin-4-yl)amine, **4j** ($CDCl_3$, 500.1 MHz)

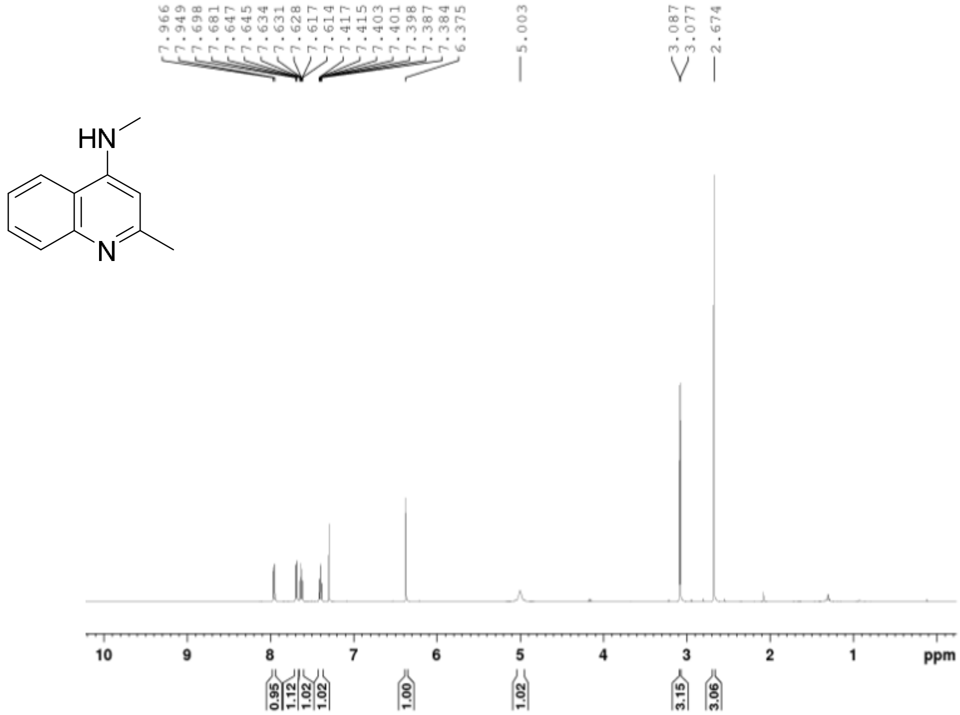

Supplementary Figure 90. $^{13}C\{^{1}H\}$ NMR of Methyl-(2-methyl-quinolin-4-yl)amine, **4j** ($CDCl_3$, 125.8 MHz)

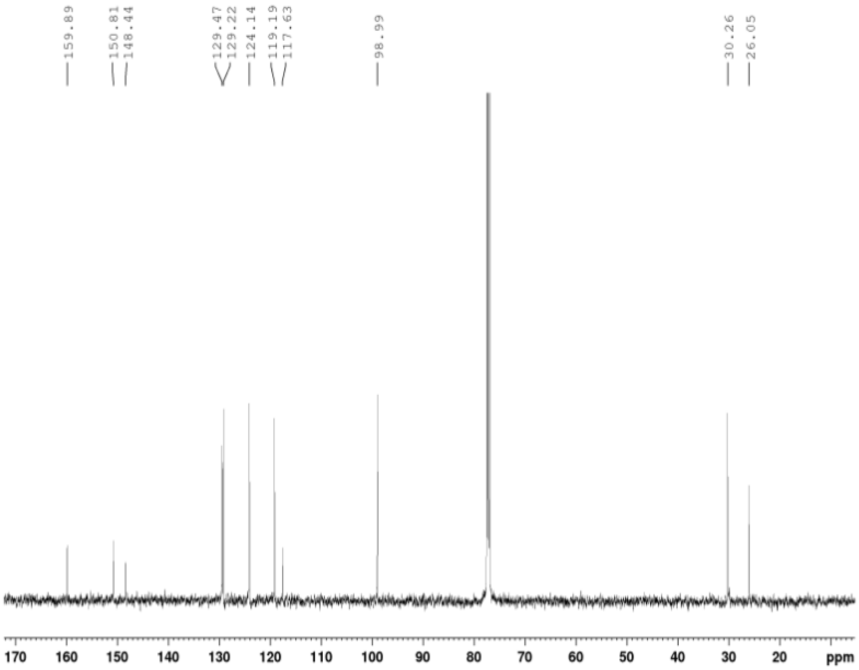

Supplementary Figure 91. ^{1}H NMR of *N*-ethyl-2-methylquinolin-4-amine, **4k** (DMSO, 300 MHz)

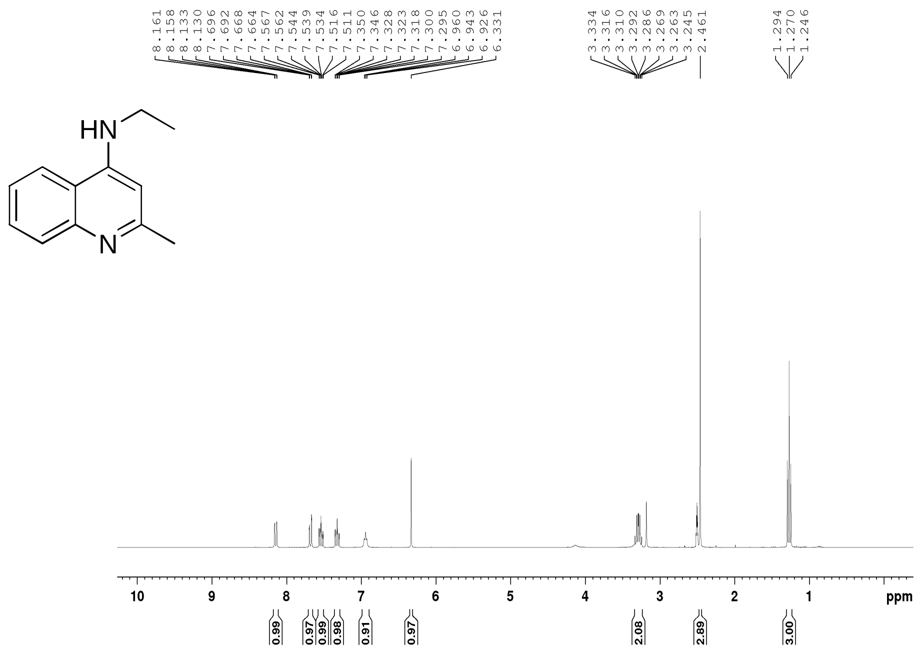

Supplementary Figure 92. ^{13}C{^{1}H} NMR of N-ethyl-2-methylquinolin-4-amine, **4k** (DMSO, 75.5 MHz)

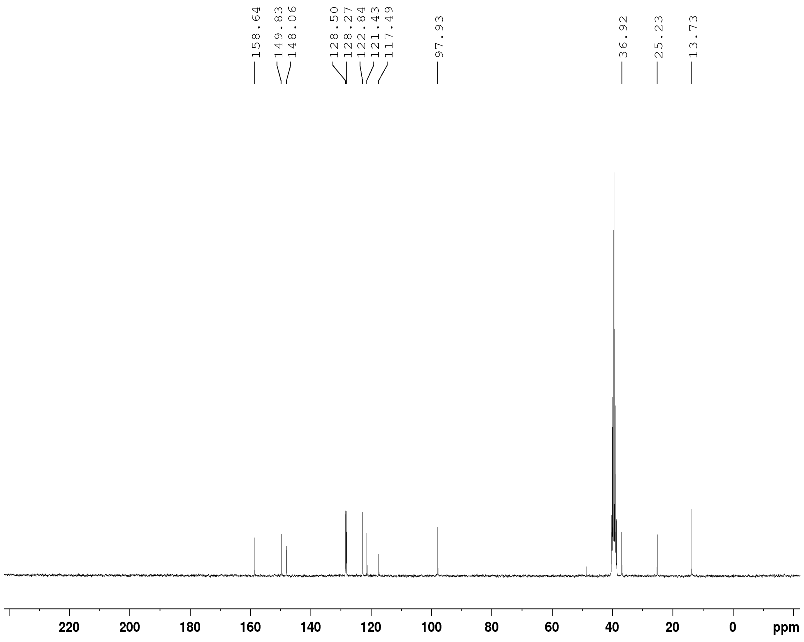

Supplementary Figure 93. ^{1}H NMR of *Sec*- Butyl-(2-methyl-quinolin-4-yl)amine, **4l** ($CDCl_3$, 500.1 MHz)

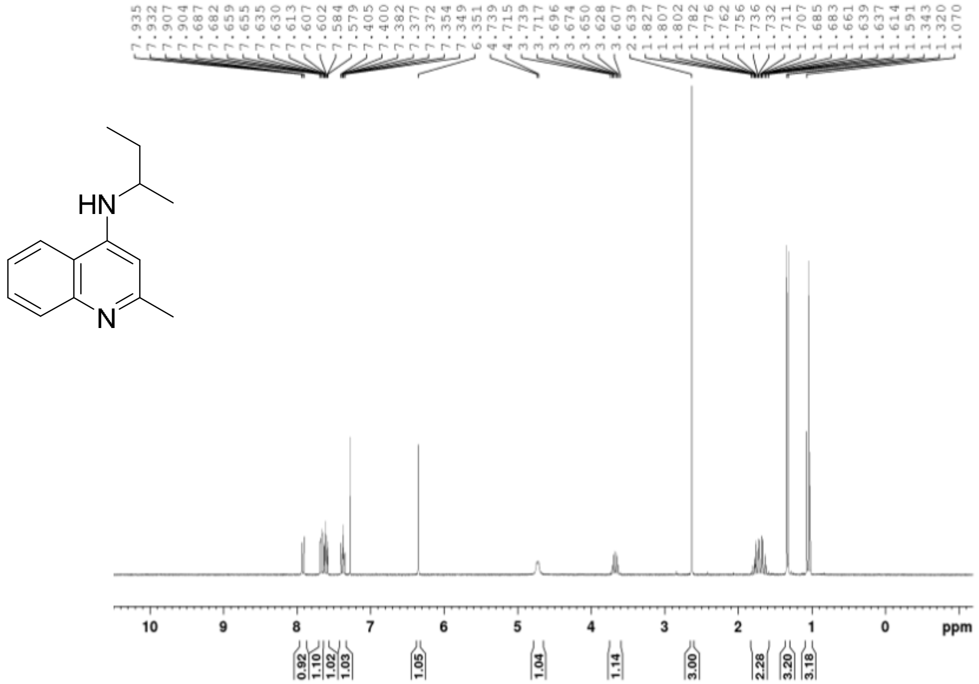

Supplementary Figure 94. $^{13}C\{^{1}H\}$ NMR of *Sec*- Butyl-(2-methyl-quinolin-4-yl)amine, **4l** ($CDCl_3$, 125.8 MHz)

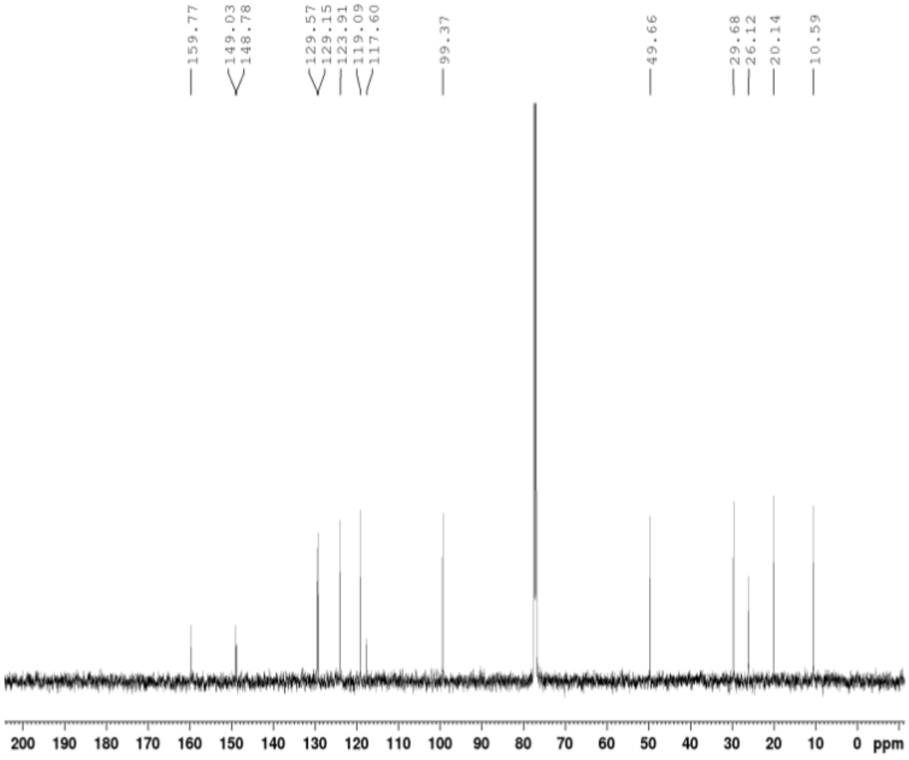

Supplementary Figure 95. ^{1}H NMR of Methyl-quinoxalin-6-yl-amine, **4m** ($CDCl_3$, 500.1 MHz)

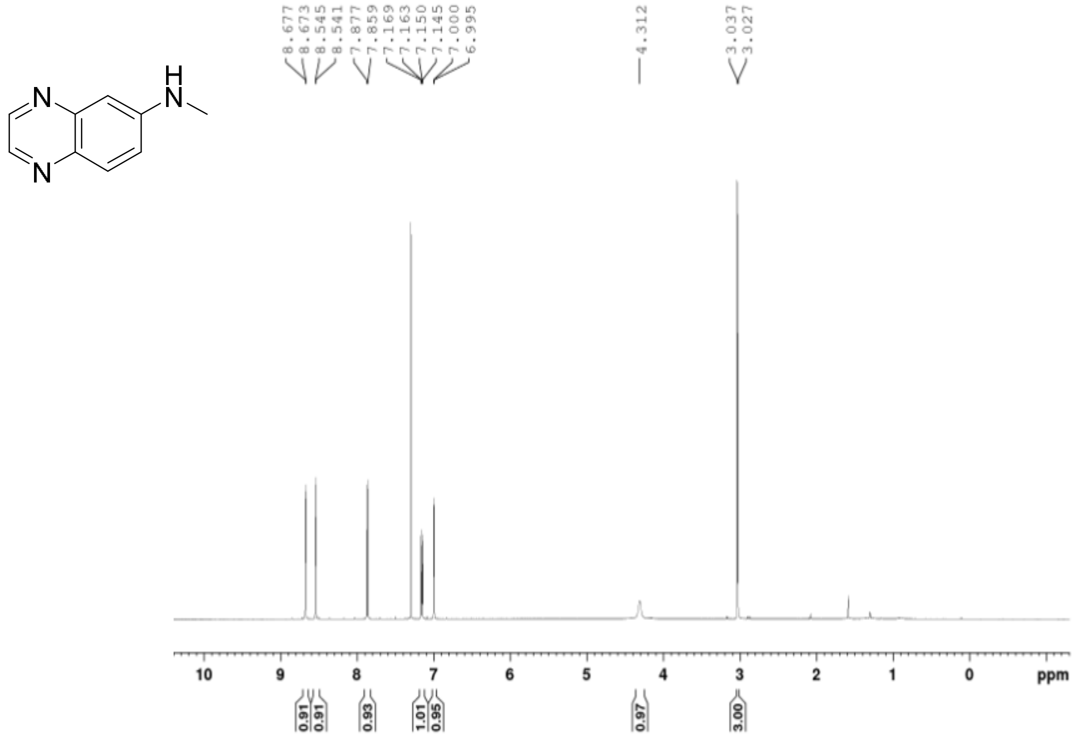

Supplementary Figure 96. $^{13}C\{^{1}H\}$ NMR of Methyl-quinoxalin-6-yl-amine, **4m** ($CDCl_3$, 125.8 MHz)

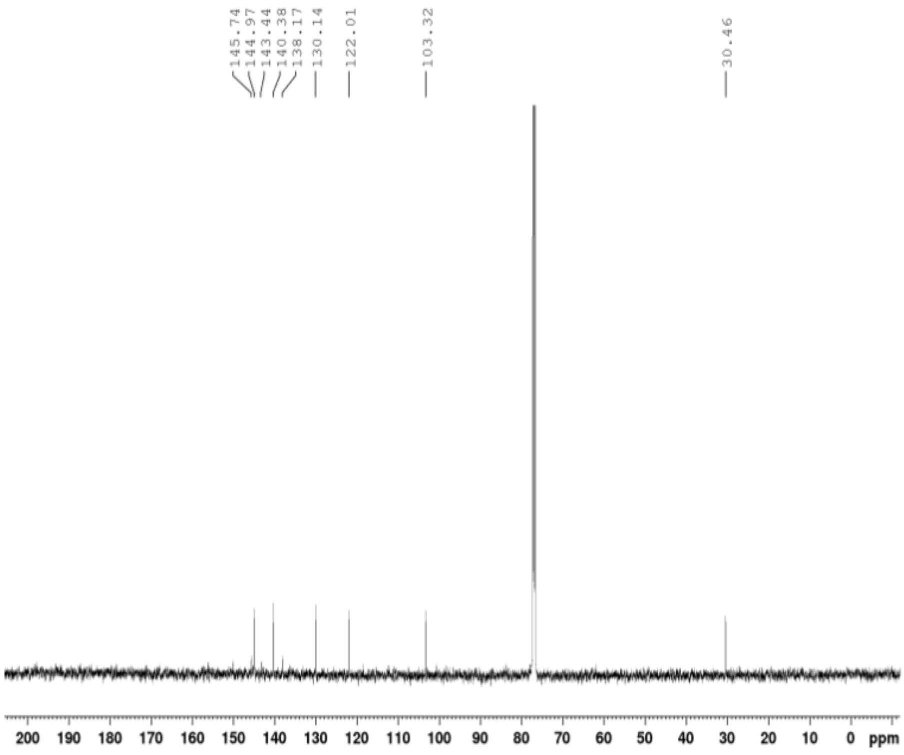

Supplementary Figure 97. ^{1}H NMR of Benzo[1,3]dioxol-5-yl-methyl-amine, **4n** ($CDCl_3$, 300.1 MHz)

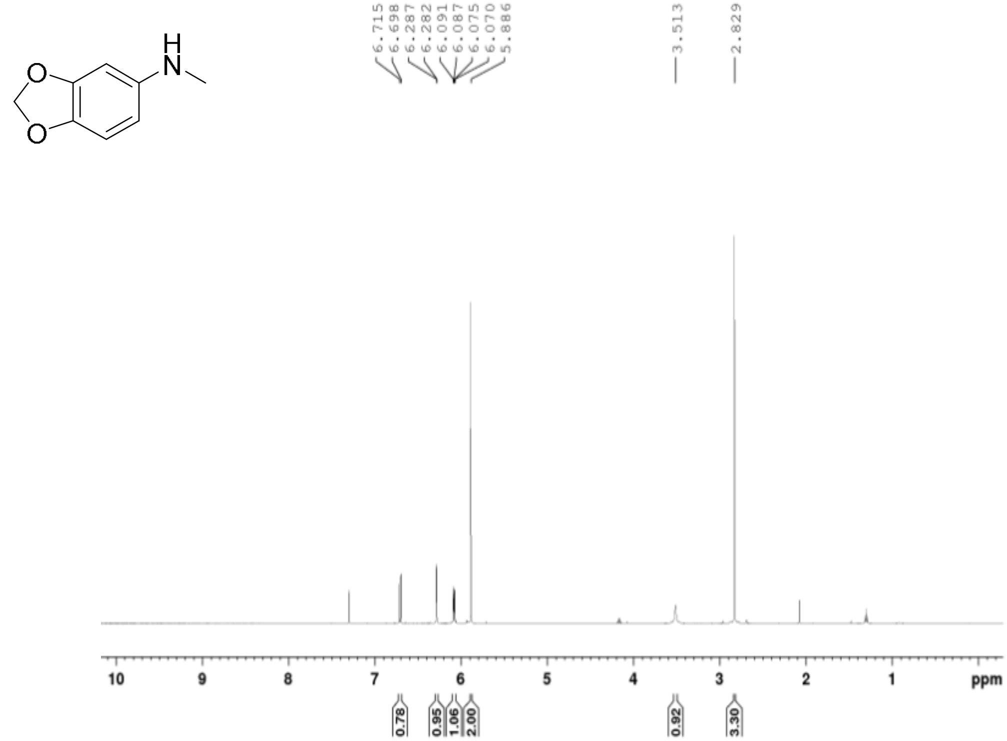

Supplementary Figure 98. $^{13}C\{^{1}H\}$ NMR of Benzo[1,3]dioxol-5-yl-methyl-amine, **4n** ($CDCl_3$, 125.8 MHz)

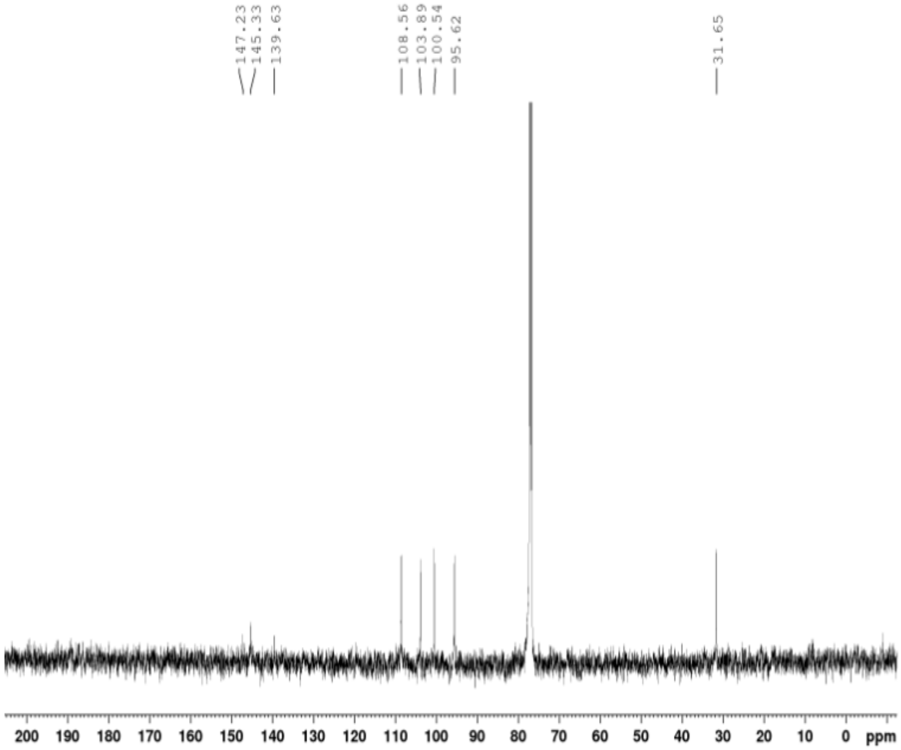

Supplementary Figure 99. ^{1}H NMR of 4-(*N*-Methylamino)phenylboronic acid pinacol ester, **4o** ($CDCl_3$, 500.1 MHz)

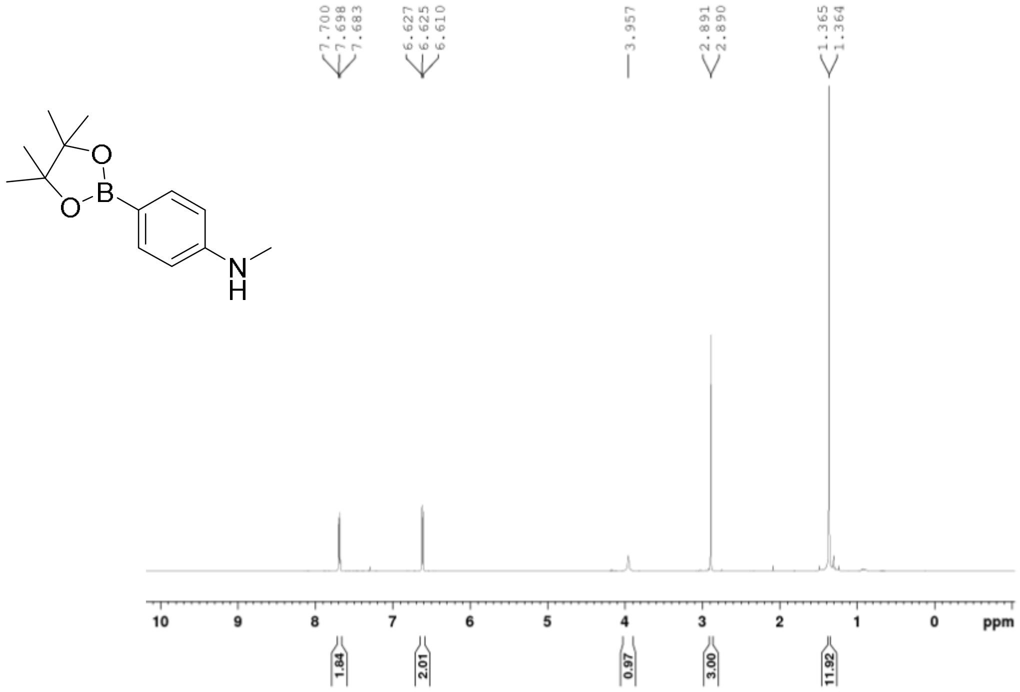

Supplementary Figure 100. ^{13}C{^{1}H} NMR of 4-(*N*-Methylamino)phenylboronic acid pinacol ester, **4o** ($CDCl_3$, 125.8 MHz)

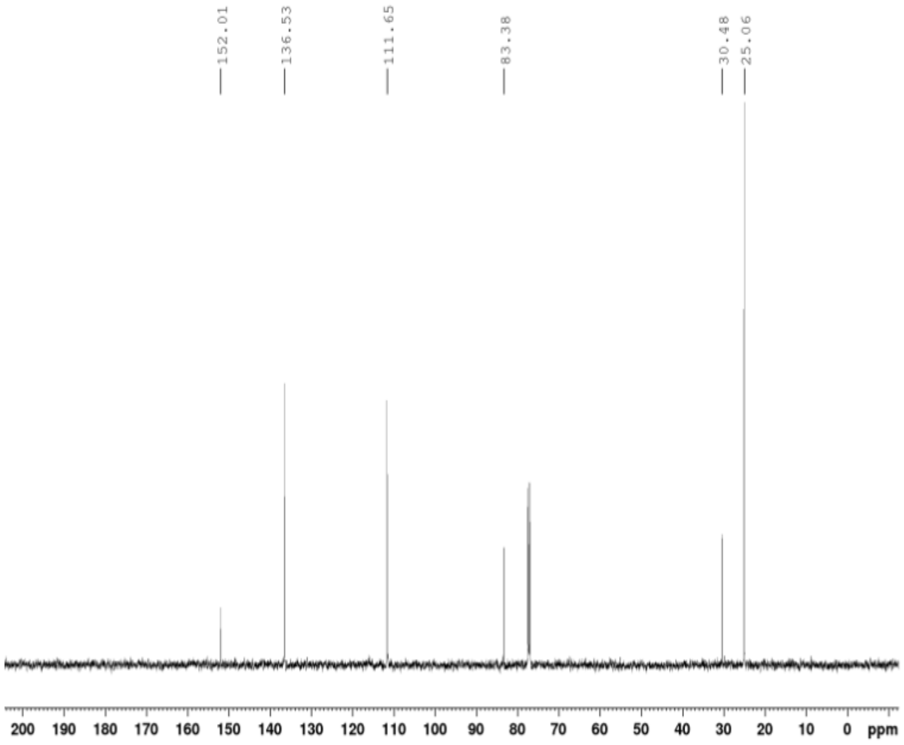

Supplementary Figure 101. ^{1}H NMR of *N*-ethyl-3-methylpyridin-2-amine, **4p** ($CDCl_3$, 300.1 MHz)

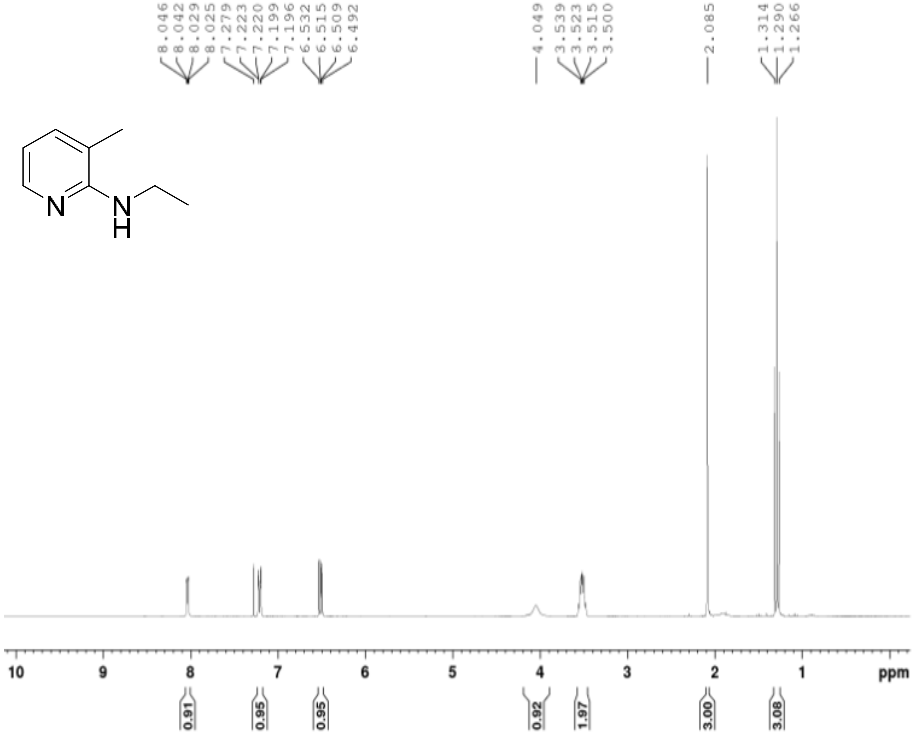

Supplementary Figure 102. $^{13}C\{^{1}H\}$ NMR of *N*-ethyl-3-methylpyridin-2-amine, **4p** ($CDCl_3$, 75.5 MHz)

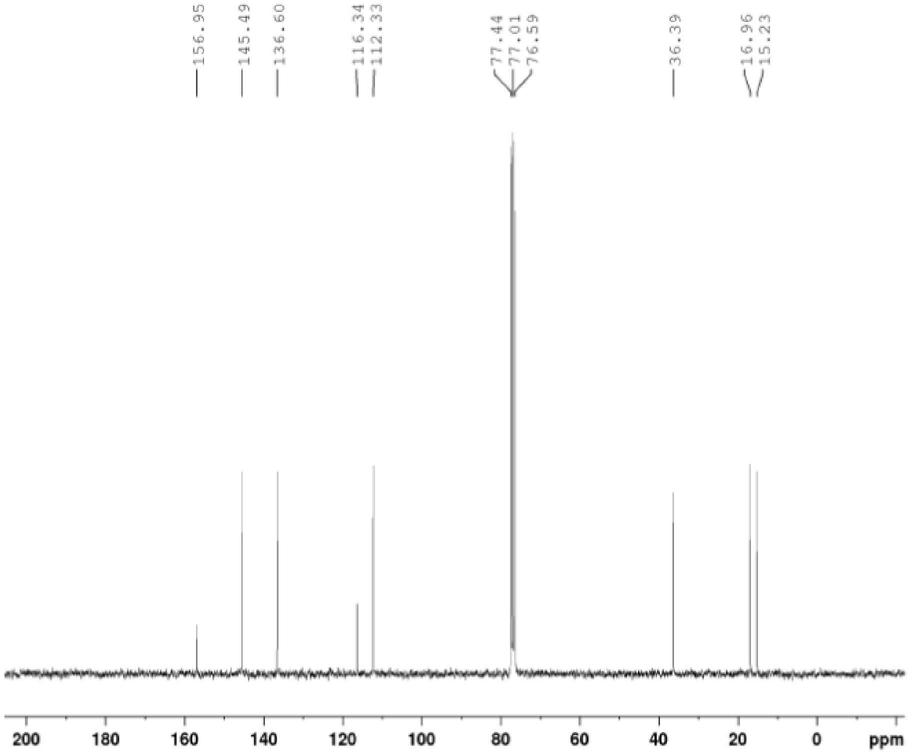

Supplementary Figure 103. ^{1}H NMR of Cyclohexyl-(2-methyl-benzothiazol-5-yl)-amine, **4q** ($CDCl_3$, 500.1 MHz)

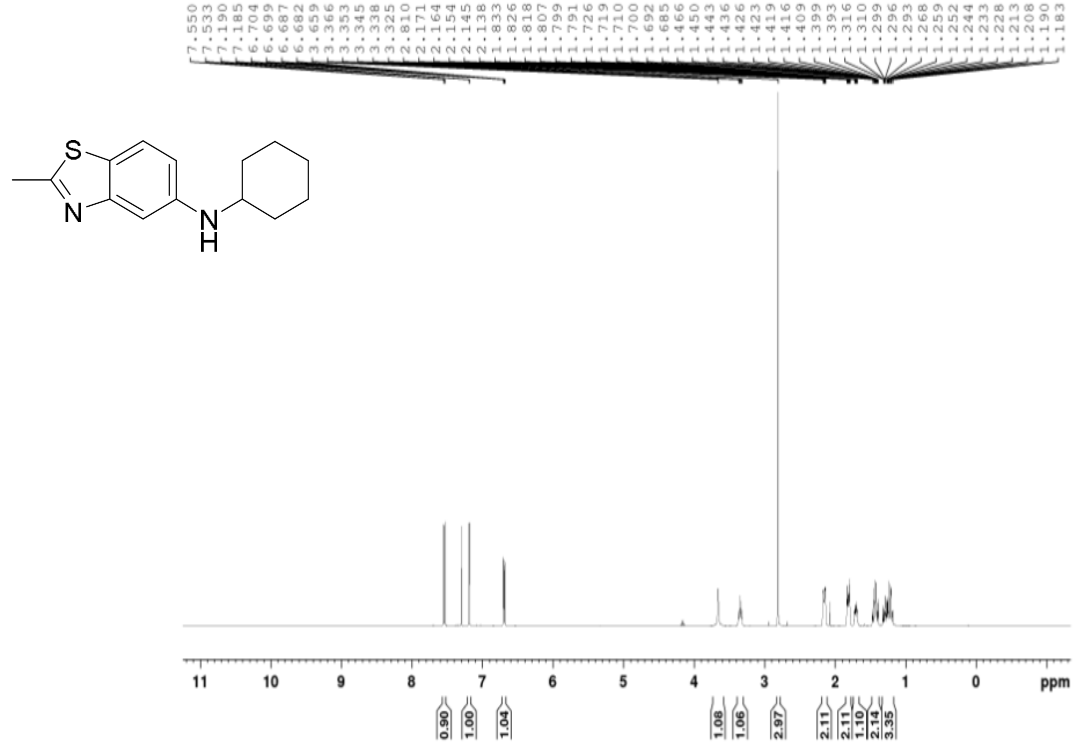

Supplementary Figure 104. ^{13}C{^{1}H} NMR of Cyclohexyl-(2-methyl-benzothiazol-5-yl)-amine, **4q** ($CDCl_3$, 125.8 MHz)

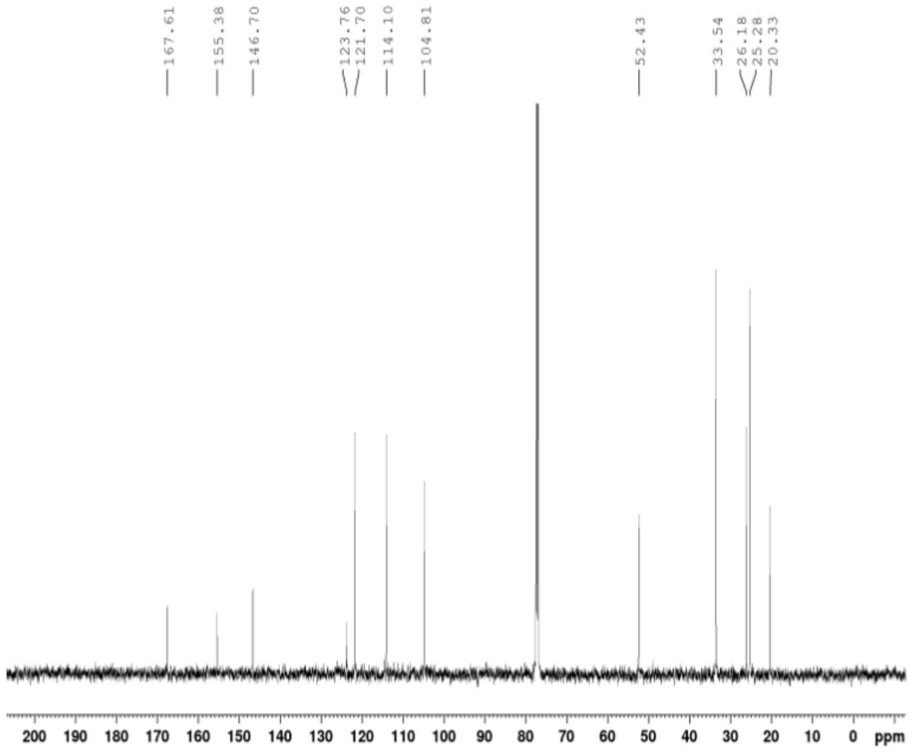

Supplementary Figure 105. ^{1}H NMR of *N*-ethyl-2-methylbenzo[*d*]thiazol-5-amine, **4r** ($CDCl_3$, 500.1 MHz)

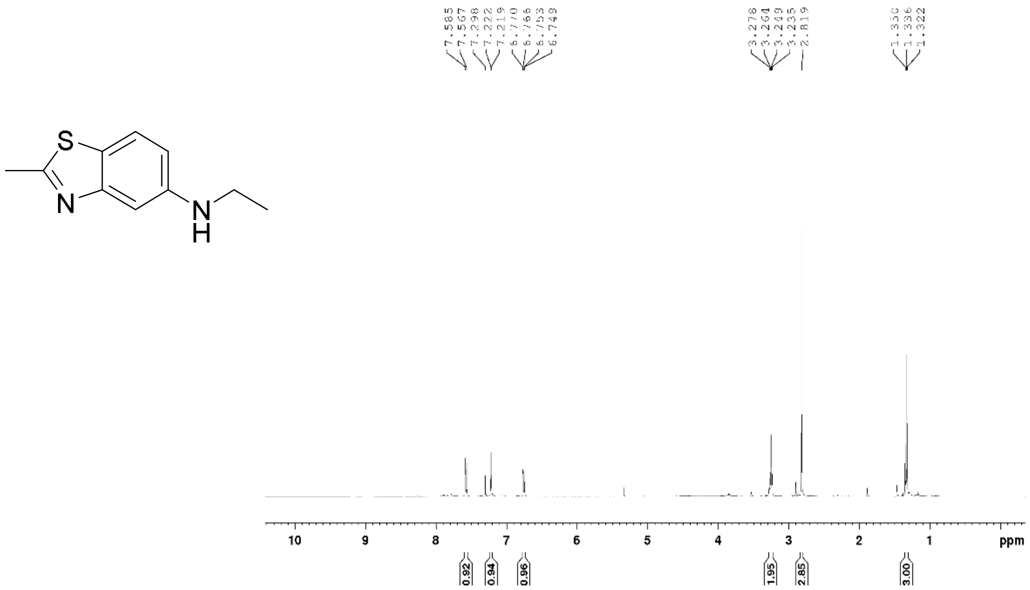

Supplementary Figure 106. ^{13}C{^{1}H} NMR of N-ethyl-2-methylbenzo[*d*]thiazol-5-amine, **4r** ($CDCl_3$, 125.8 MHz)

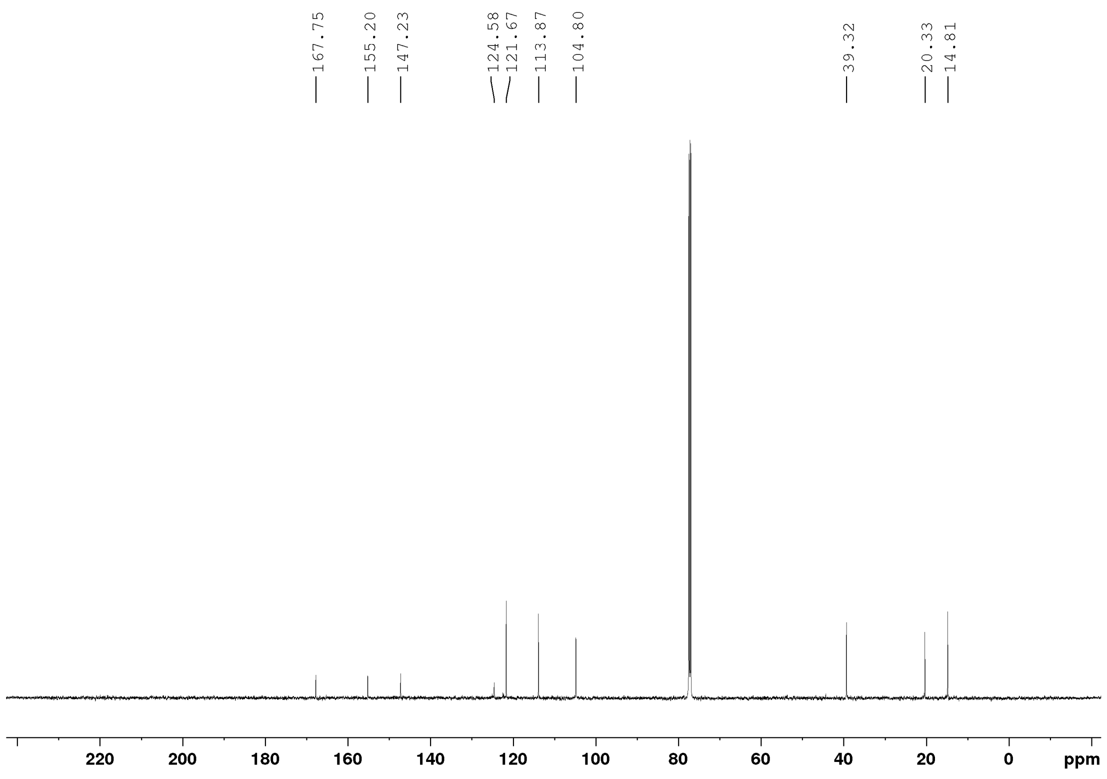

Supplementary Figure 107. ^{1}H NMR of (4-Phenoxy-phenyl)-(1-phenyl-ethyl)-amine, **4s** ($CDCl_3$, 300.1 MHz)

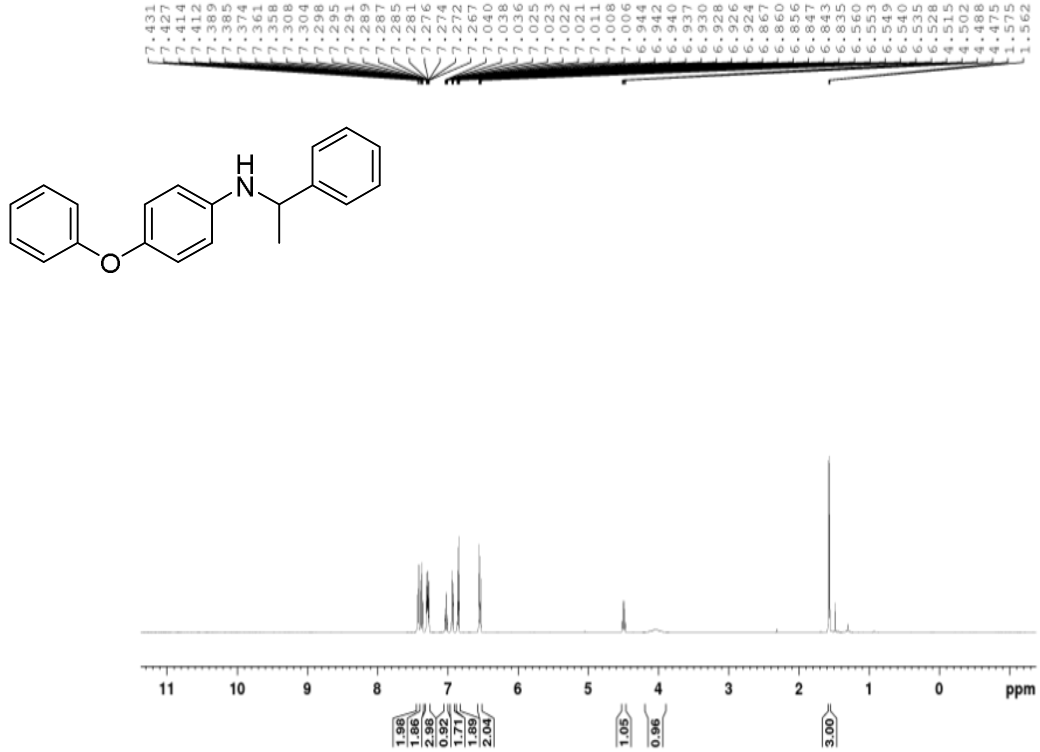

Supplementary Figure 108. $^{13}C\{^{1}H\}$ NMR of (4-Phenoxy-phenyl)-(1-phenyl-ethyl)-amine, **4s** ($CDCl_3$, 75.5 MHz)

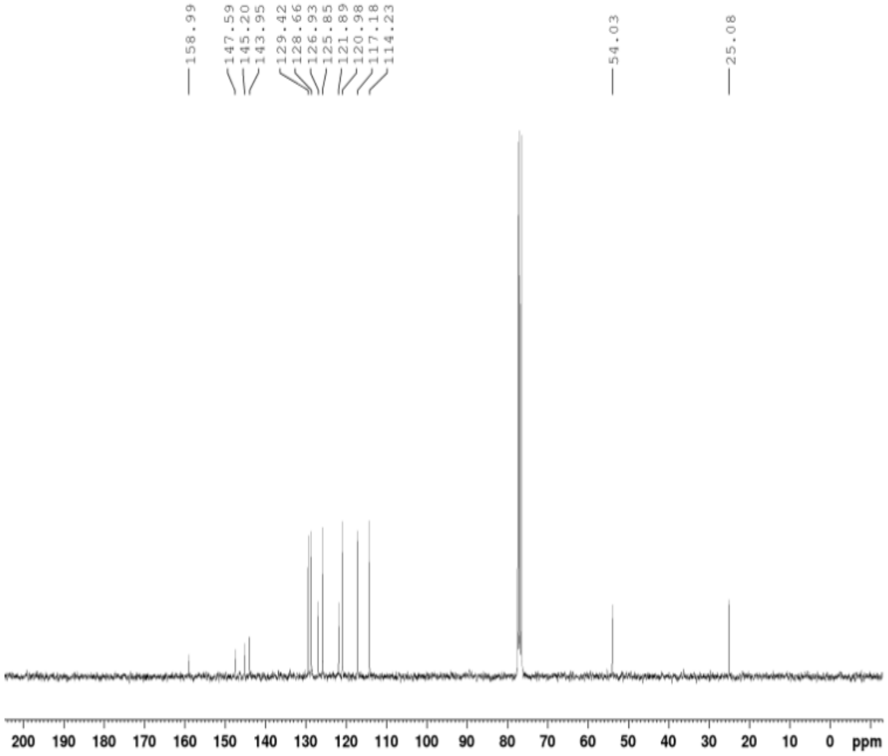

Supplementary Figure 109. ^{1}H NMR of 4-methyl-*N*-phenylpiperazin-1-amine, **4t** ($CDCl_3$, 500.0 MHz)

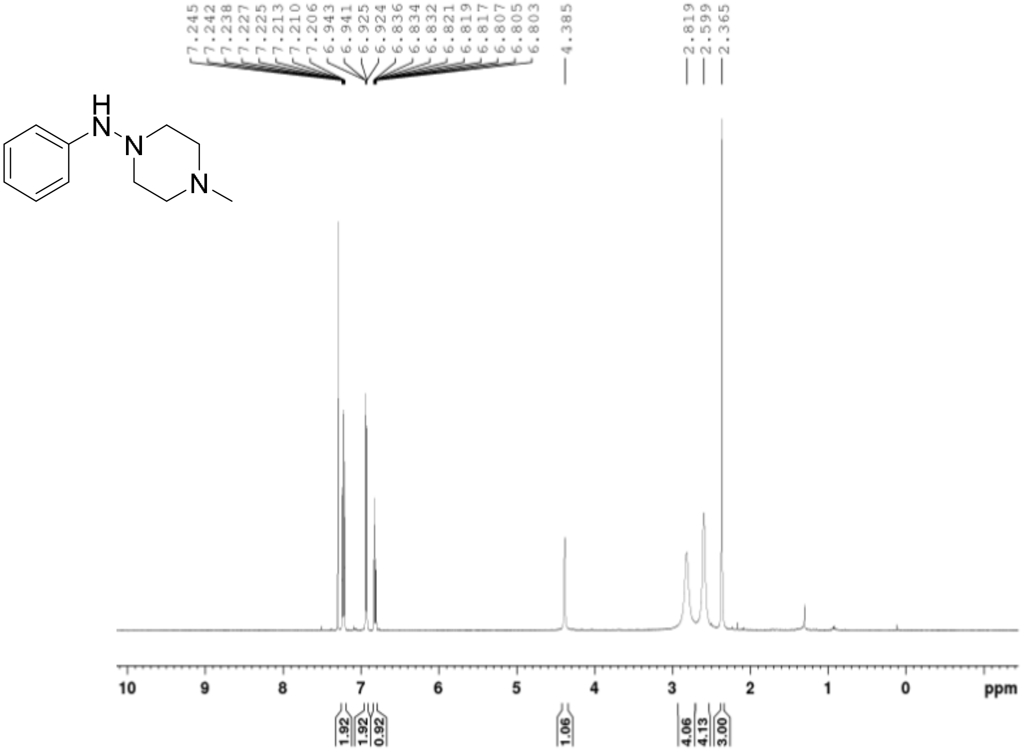

Supplementary Figure 110. $^{13}C\{^{1}H\}$ NMR of 4-methyl-*N*-phenylpiperazin-1-amine, **4t** ($CDCl_3$, 125.8 MHz)

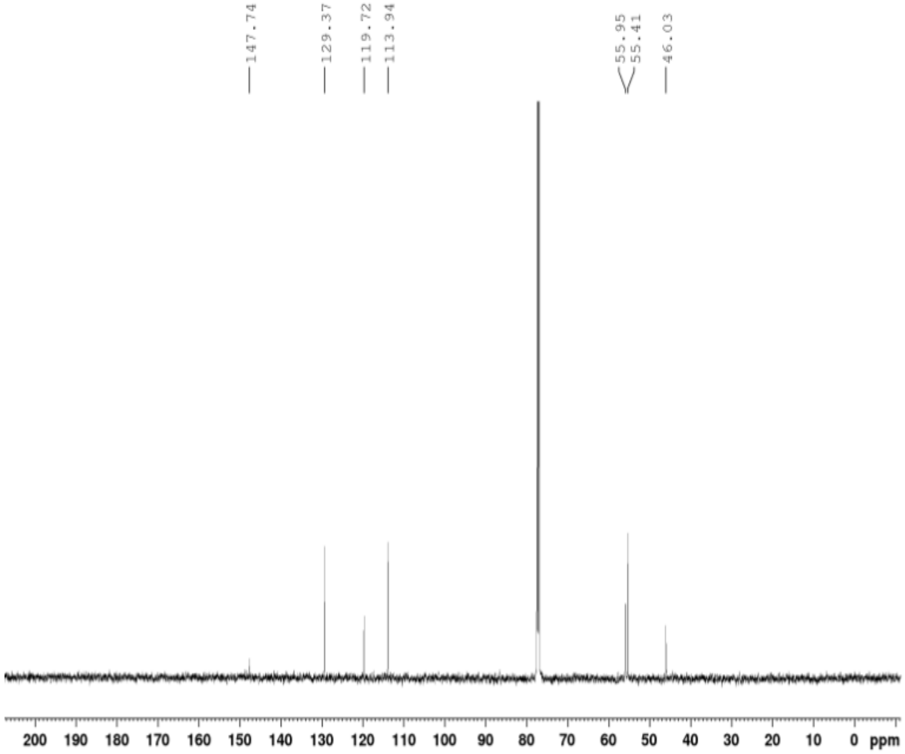

Supplementary Figure 111. ^{1}H NMR of Benzo[*b*]thiophen-5-yl-(3-trifluoromethyl-phenyl)-amine, **4u** ($CDCl_3$, 500.1 MHz)

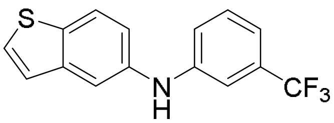

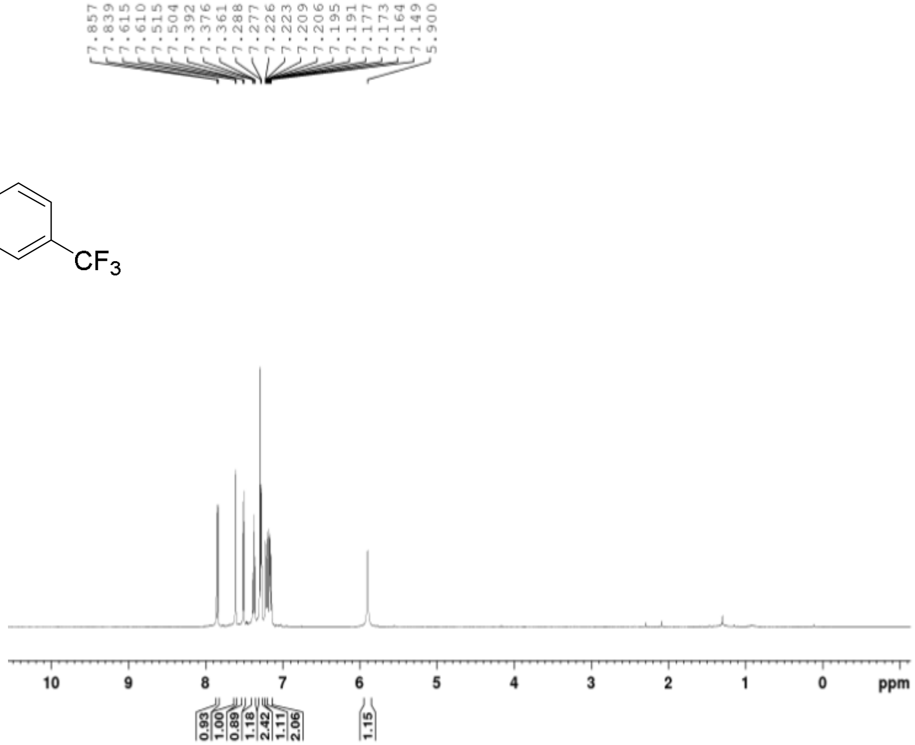

Supplementary Figure 112. $^{13}C\{^1H\}$ NMR of Benzo[*b*]thiophen-5-yl-(3-trifluoromethyl-phenyl)- amine, **4u** ($CDCl_3$, 125.8 MHz)

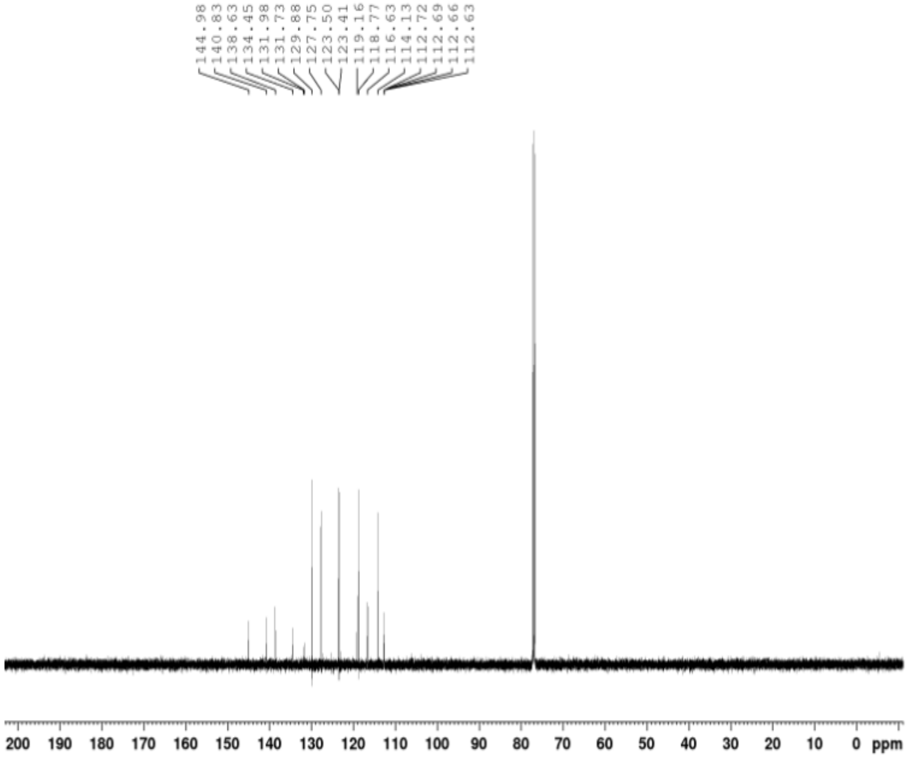

Supplementary Figure 113. ^{1}H NMR of (4-Methoxy-phenyl)-(3-trifluoromethyl-phenyl)- amine, **4v** ($CDCl_3$, 500.1 MHz)

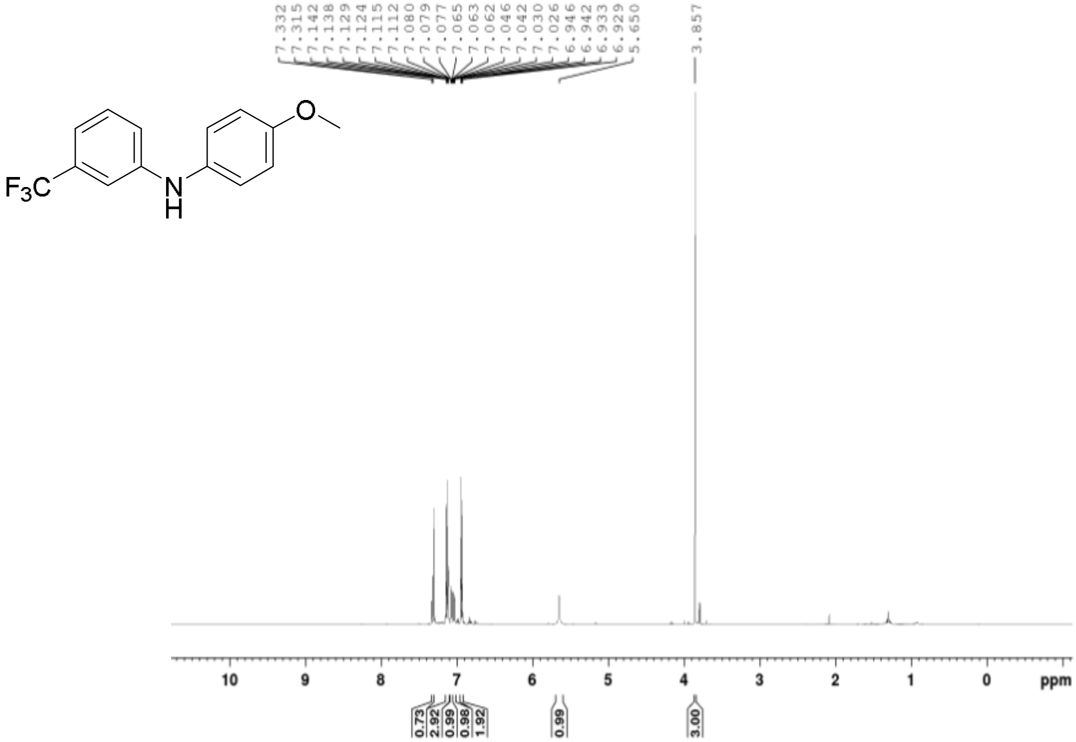

Supplementary Figure 114. $^{13}C\{^{1}H\}$ NMR of (4-Methoxy-phenyl)-(3-trifluoromethyl-phenyl)-amine, **4v** ($CDCl_3$, 125.8 MHz)

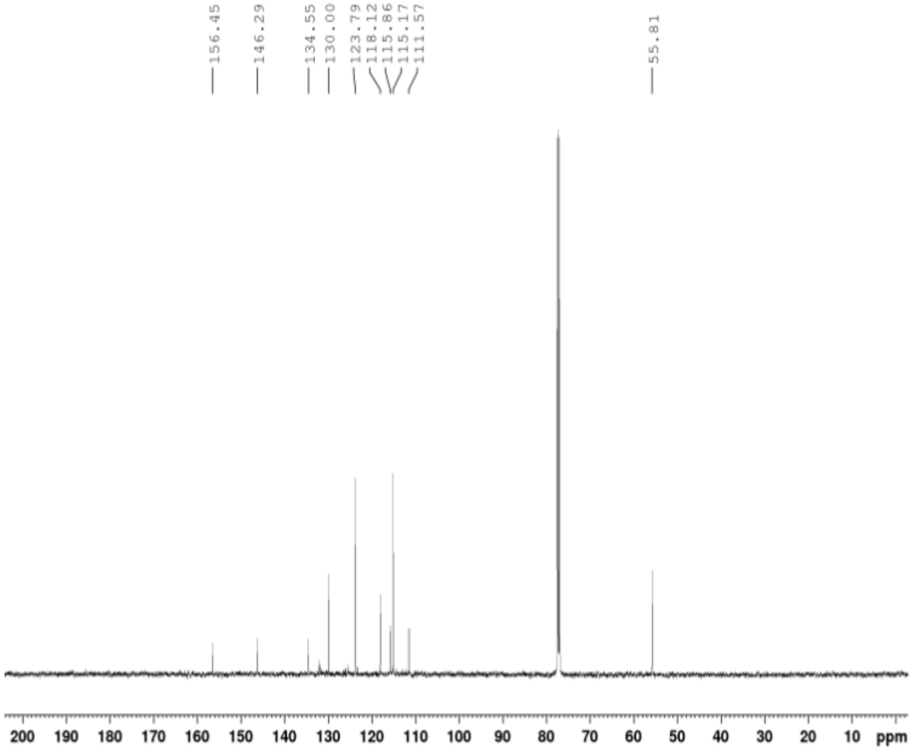

Supplementary Figure 115. ^{1}H NMR of (4,6-Dimethoxy-pyrimidin-2-yl)-phenethyl-amine, **4w** ($CDCl_3$, 500.1 MHz)

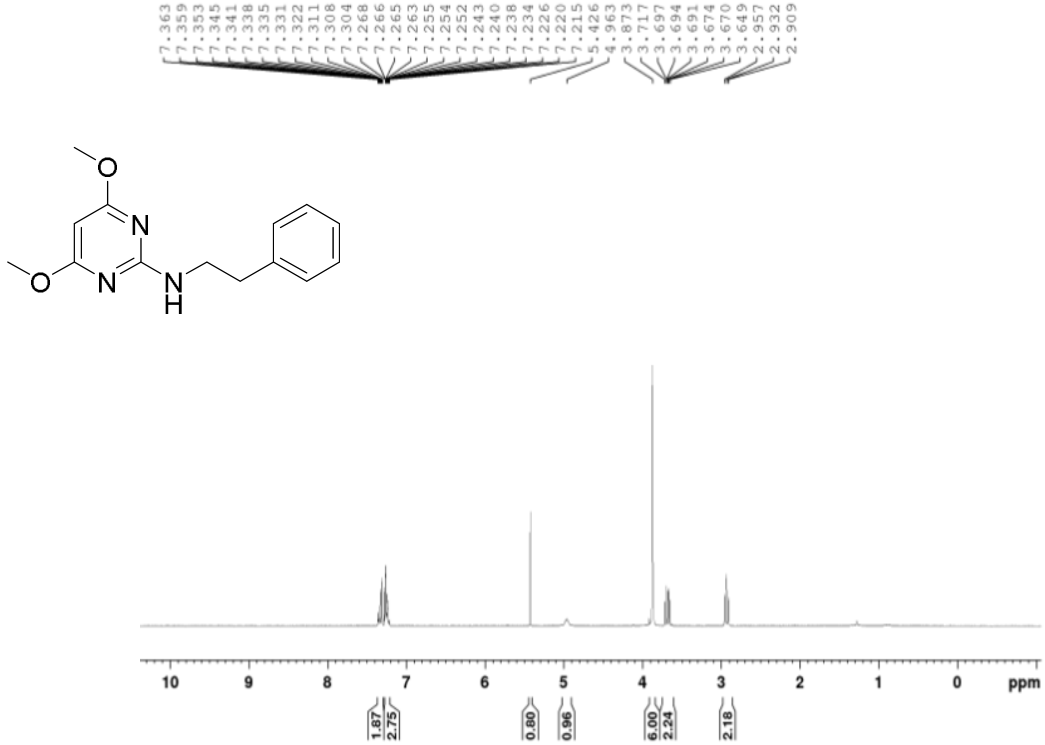

Supplementary Figure 116. ^{13}C{^{1}H} NMR of (4,6-Dimethoxy-pyrimidin-2-yl)-phenethyl-amine, **4w** ($CDCl_3$, 125.8 MHz)

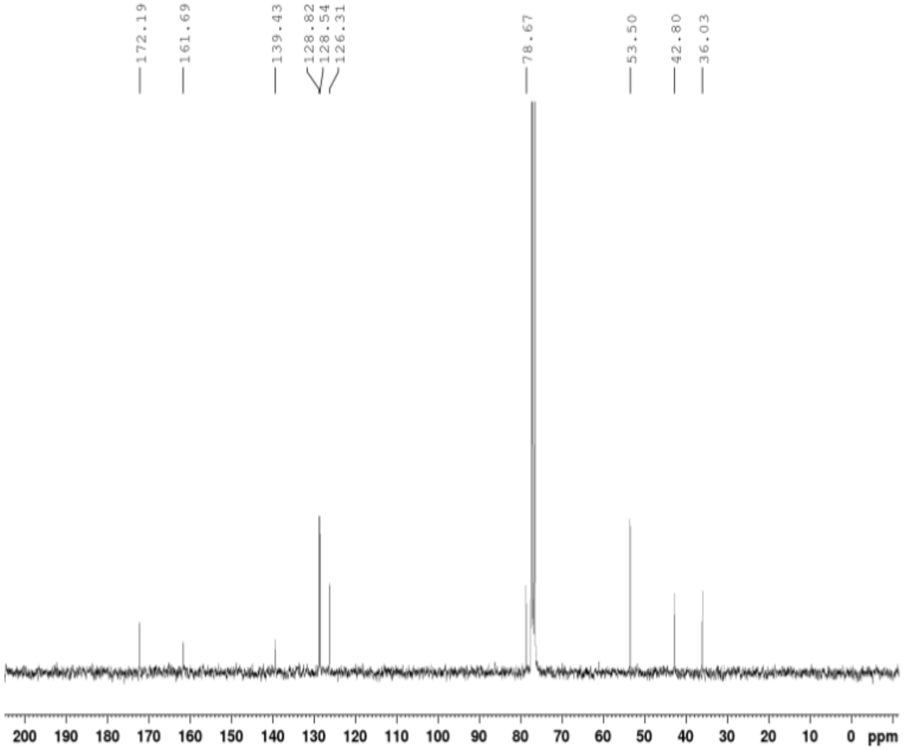

Supplementary Figure 117. ^{1}H NMR of Furan-2-ylmethyl-(4-pyrrol-1-yl-phenyl)-amine, **4x** ($CDCl_3$, 500.1 MHz)

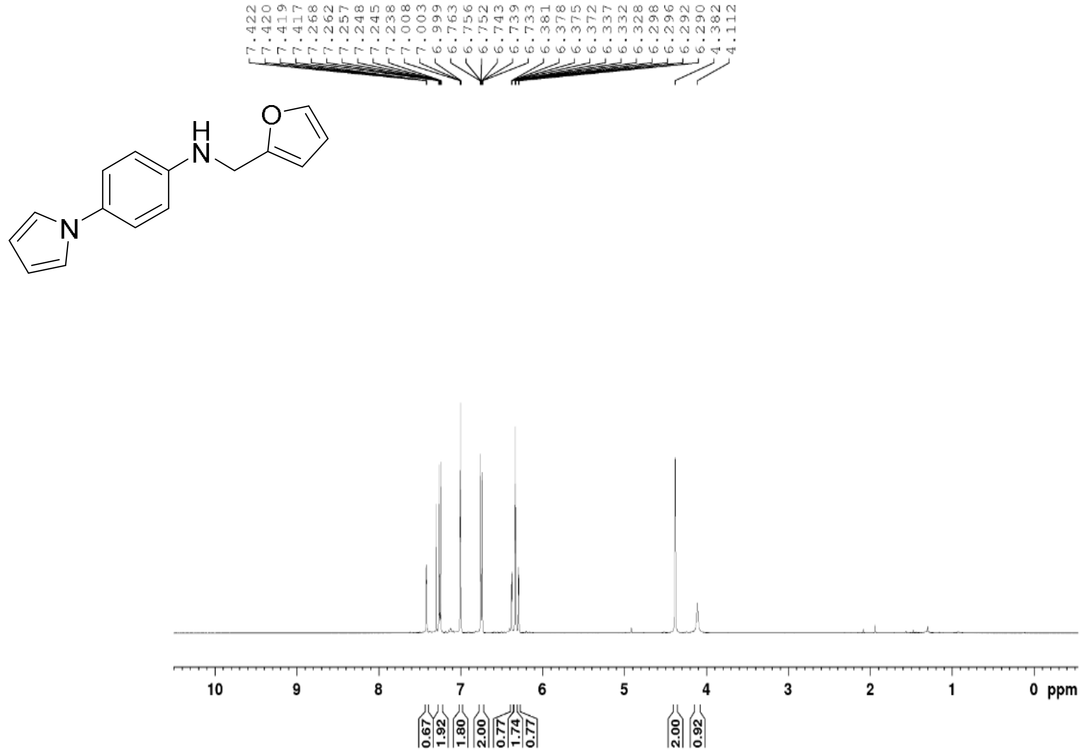

Supplementary Figure 118. $^{13}C\{^{1}H\}$ NMR of Furan-2-ylmethyl-(4-pyrrol-1-yl-phenyl)-amine, **4x** ($CDCl_3$, 125.8 MHz)

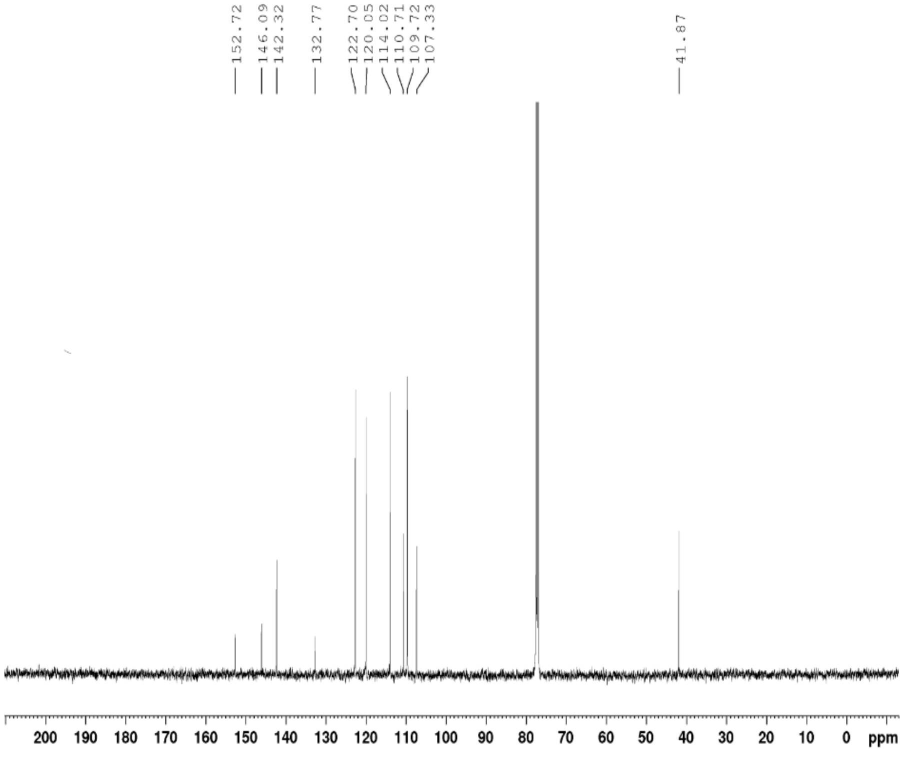

Supplementary Figure 119. ^{1}H NMR of Phenyl-{4-[(tetrahydro-furan-2-ylmethyl)- amino]-phenyl}-methanone, **4y** ($CDCl_3$, 500.1 MHz)

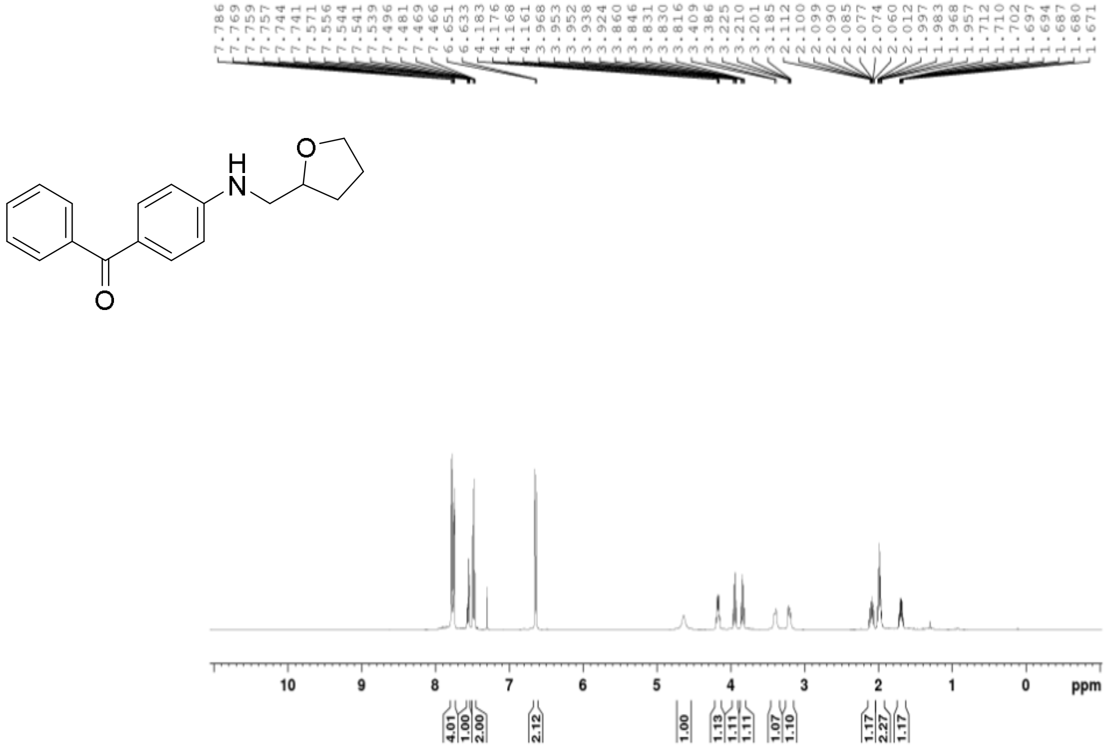

Supplementary Figure 120. ^{13}C{^{1}H} NMR of Phenyl-{4-[(tetrahydro-furan-2-ylmethyl)-amino]-phenyl}-methanone, **4y** ($CDCl_3$, 125.8 MHz)

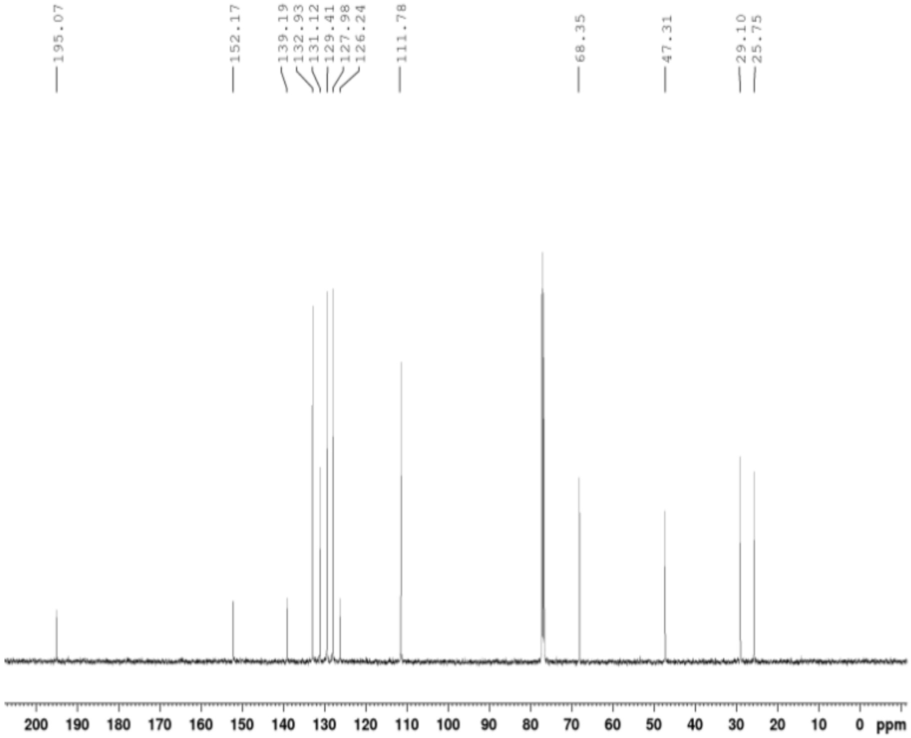

Supplementary Figure 121. ^{1}H NMR of 4-Methyl-3-[(thiophen-2-ylmethyl)-amino]-benzonitrile, **4z** ($CDCl_3$, 300.1 MHz)

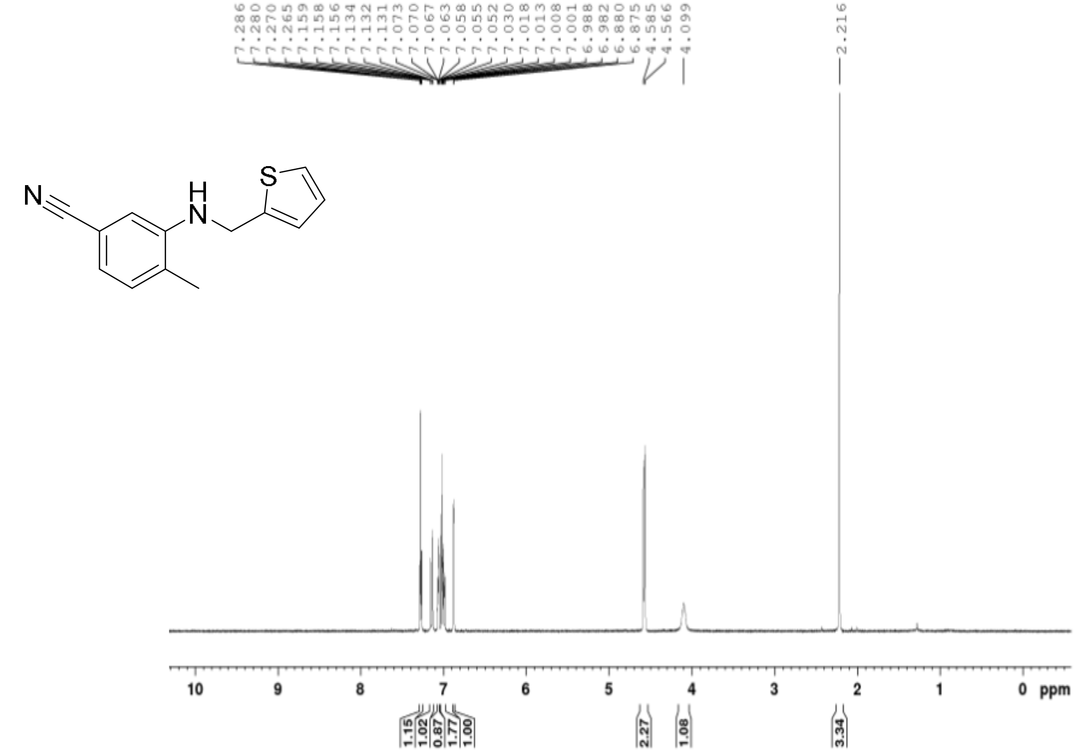

Supplementary Figure 122. $^{13}C\{^{1}H\}$ NMR of 4-Methyl-3-[(thiophen-2-ylmethyl)-amino]-benzonitrile, **4z** ($CDCl_3$, 125.8 MHz)

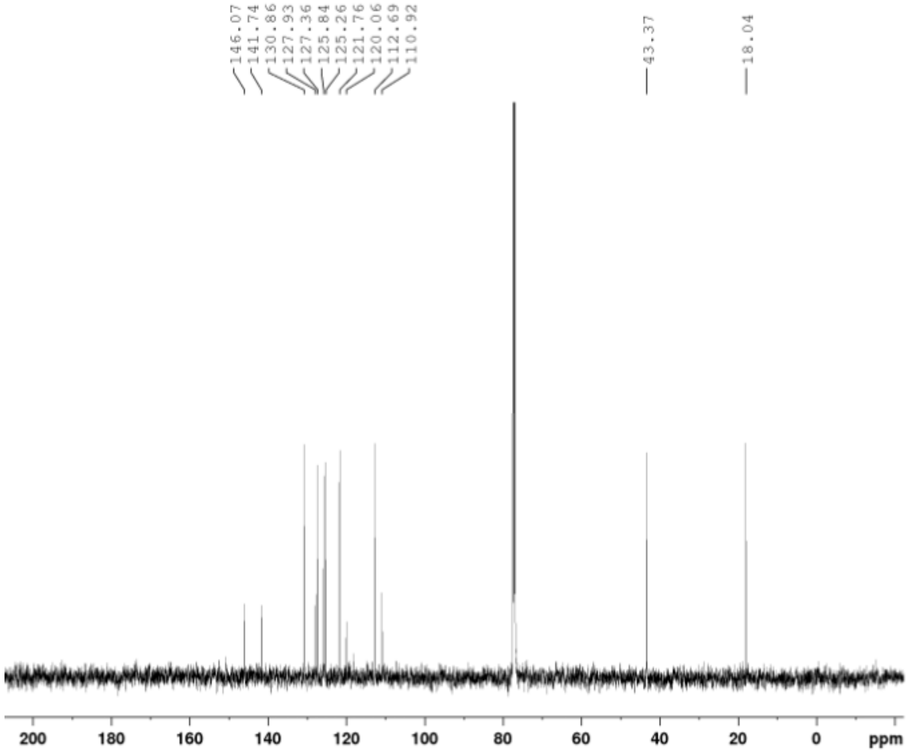

Supplementary Figure 123. ^{1}H NMR of (2,5-Dimethyl-phenyl)-thiophen-2-ylmethyl-amine, **4aa** ($CDCl_3$, 500.1 MHz)

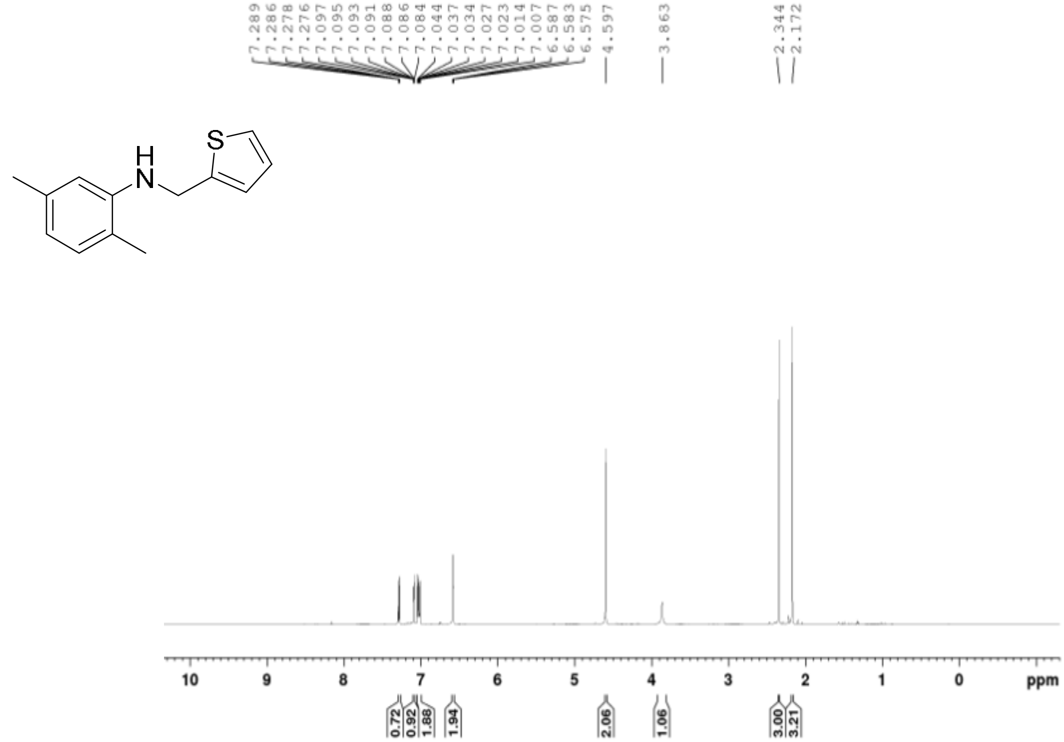

Supplementary Figure 124. $^{13}C\{^{1}H\}$ NMR of (2,5-Dimethyl-phenyl)-thiophen-2-ylmethyl-amine, **4aa** ($CDCl_3$, 125.8 MHz)

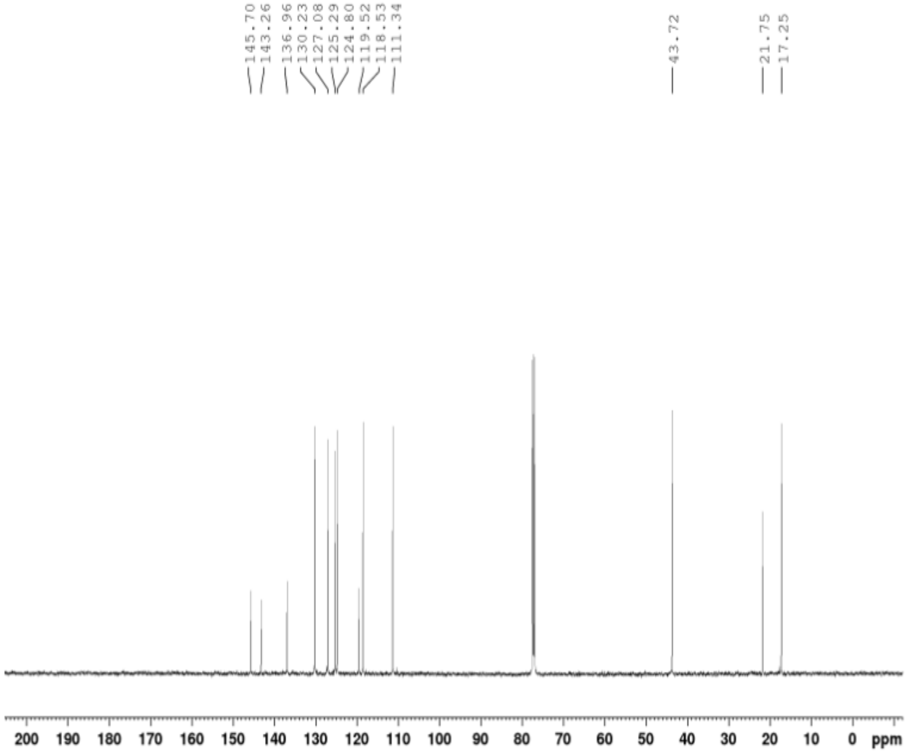

Supplementary Figure 125. ^{1}H NMR of Benzyl-quinolin-8-yl-amine, **4ab** ($CDCl_3$, 300.1 MHz)

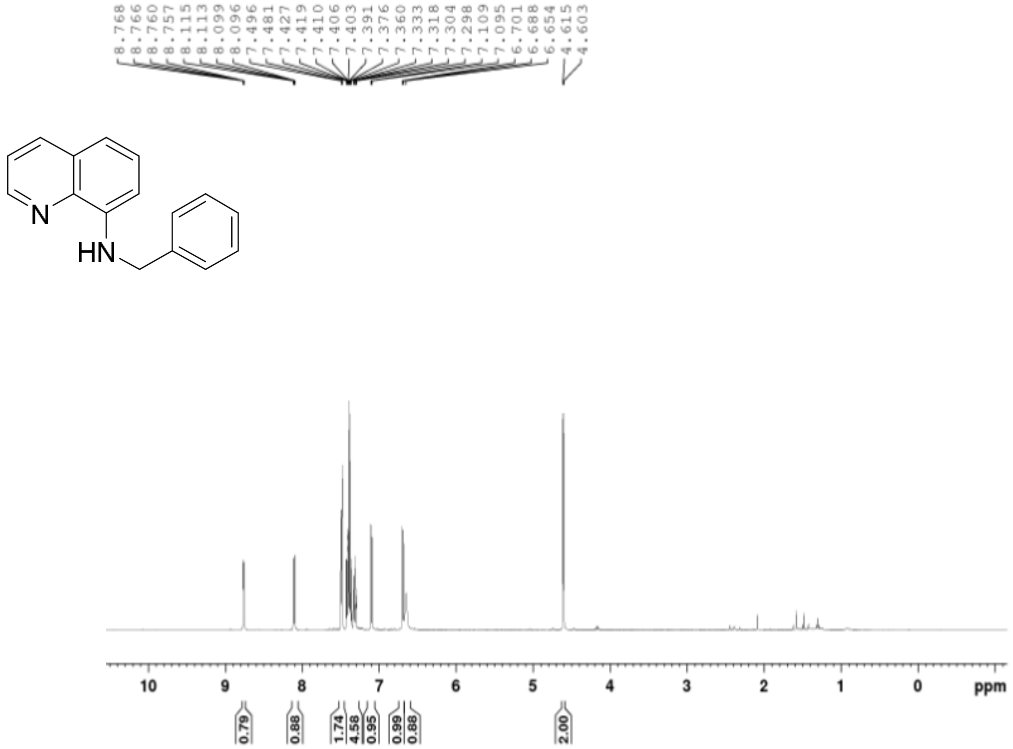

Supplementary Figure 126. $^{13}C\{^{1}H\}$ NMR of Benzyl-quinolin-8-yl-amine, **4ab** ($CDCl_3$, 75.5 MHz)

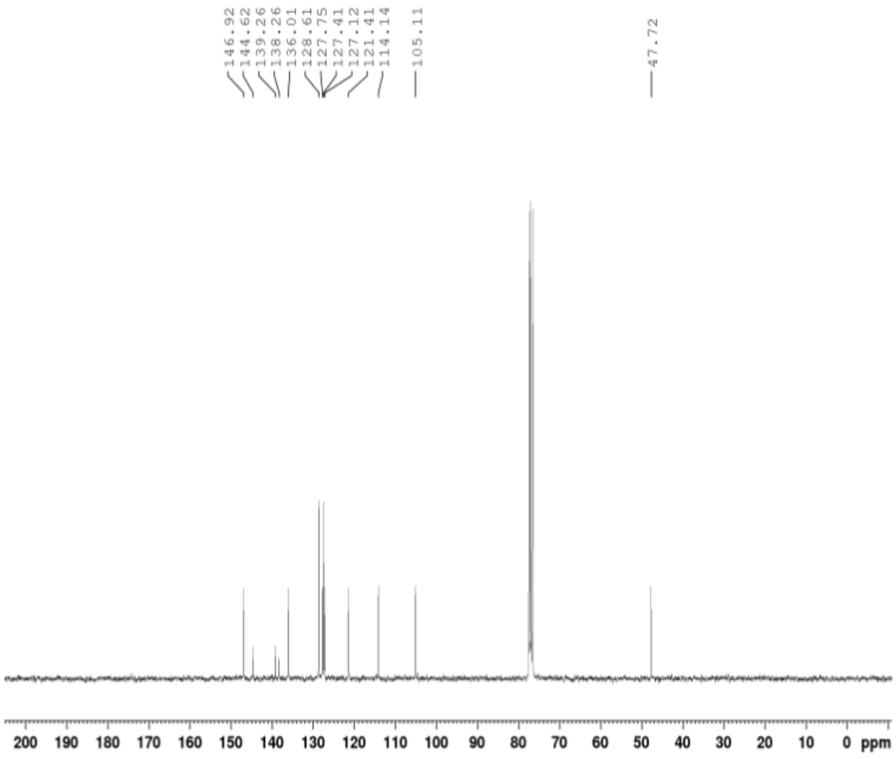

Supplementary Figure 127. ^{1}H NMR of 2-Allylamino-benzonitrile, **4ac** ($CDCl_3$, 500.1 MHz)

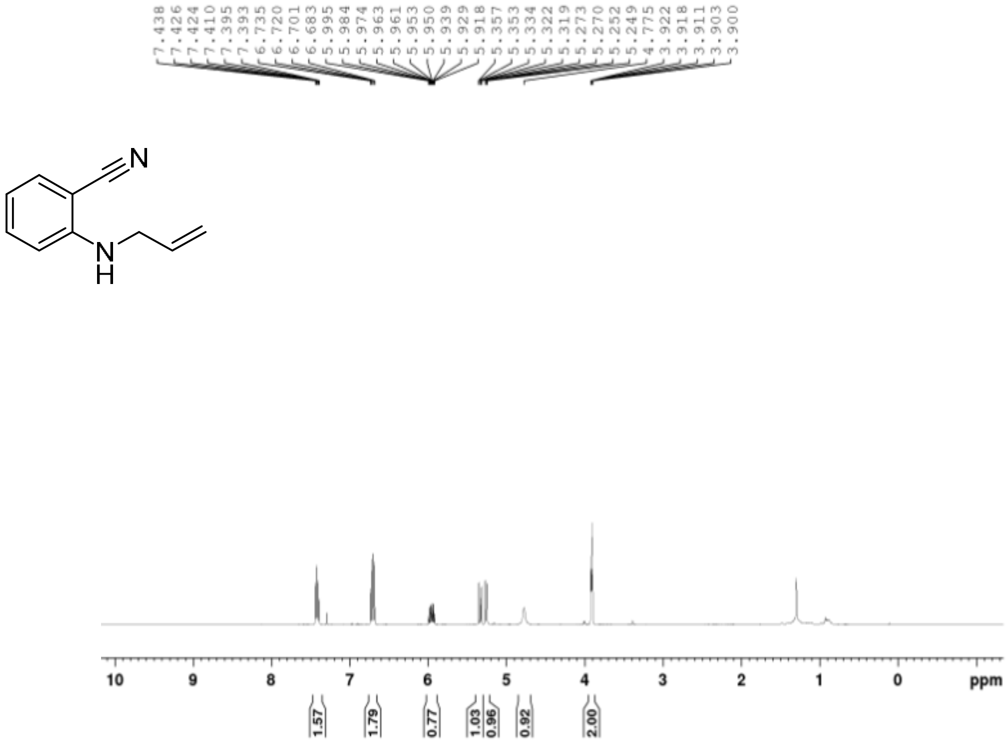

Supplementary Figure 128. $^{13}C\{^1H\}$ NMR of 2-Allylamino-benzonitrile, **4ab** ($CDCl_3$, 125.8 MHz)

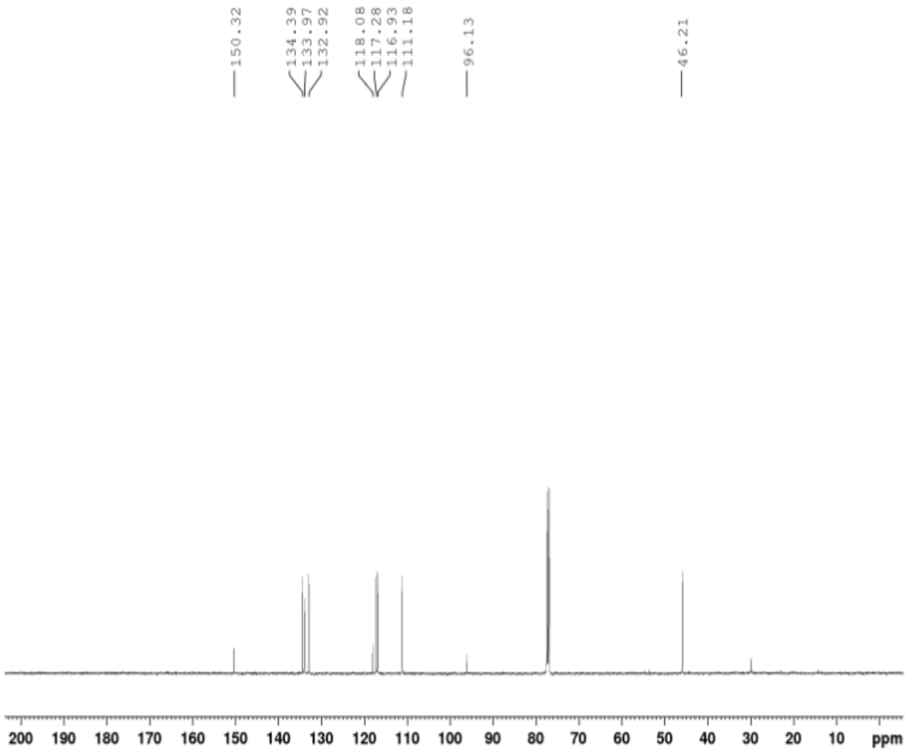

Supplementary Figure 129. ^{1}H NMR of (6-Methoxy-pyridin-2-yl)-piperidin-4-ylmethyl-amine, **4ad** ($CDCl_3$, 300.1 MHz)

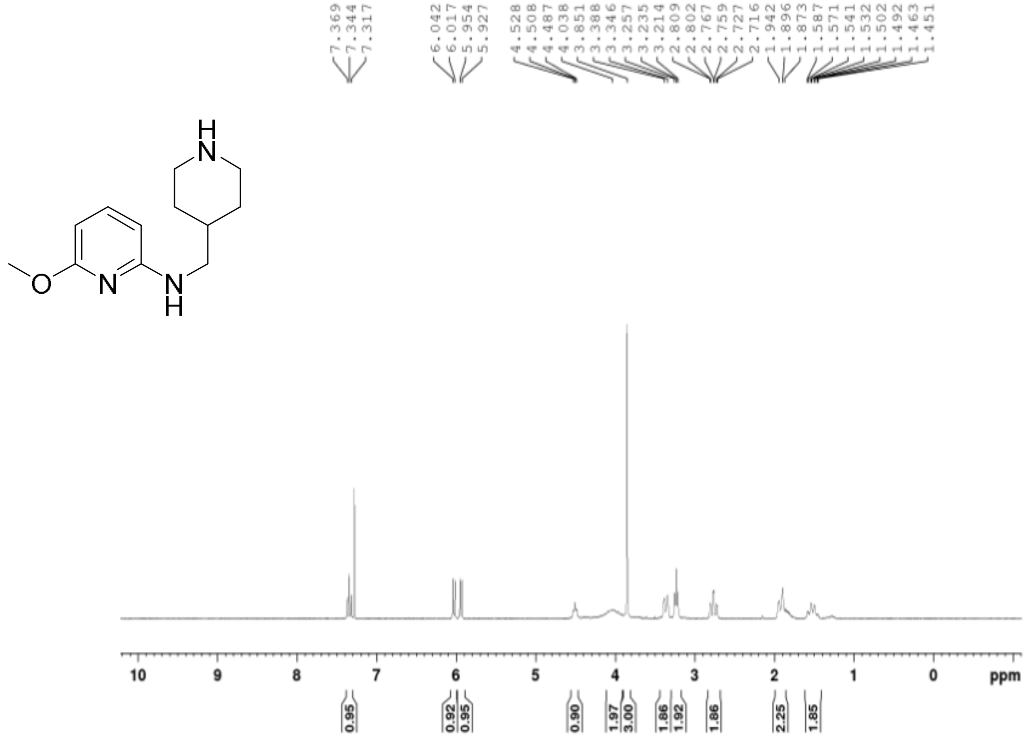

Supplementary Figure 130. $^{13}C\{^{1}H\}$ NMR of (6-Methoxy-pyridin-2-yl)-piperidin-4-ylmethyl-amine, **4ad** ($CDCl_3$, 125.8 MHz)

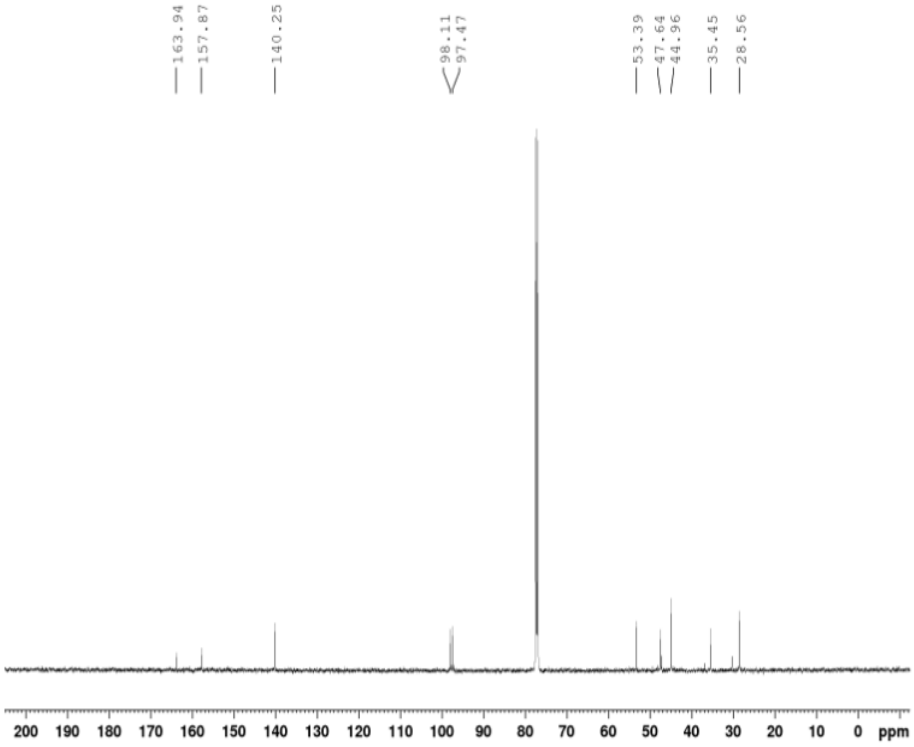

Supplementary Figure 131. ^{1}H NMR of *N*,*N*'-Diphenyl-ethane-1,2-diamine, **4ae** ($CDCl_3$, 300.1 MHz)

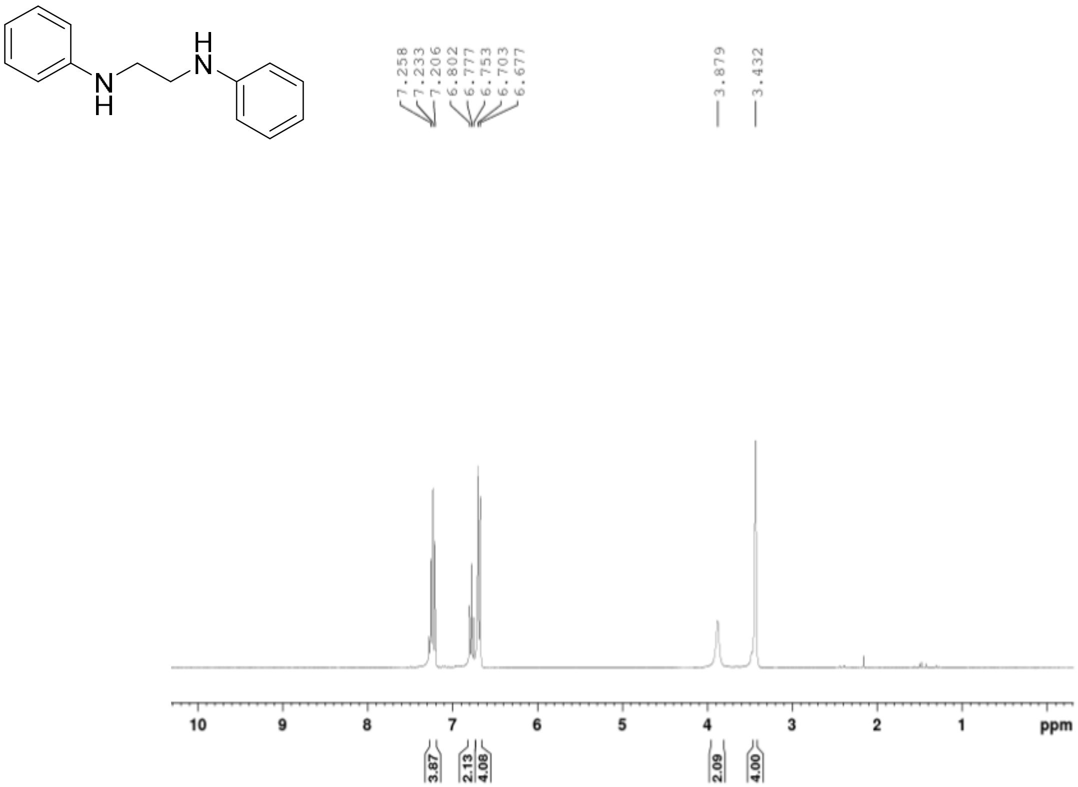

Supplementary Figure 132. ^{13}C{^{1}H} NMR of *N*,*N*'-Diphenyl-ethane-1,2-diamine, **4ae** ($CDCl_3$, 125.8 MHz)

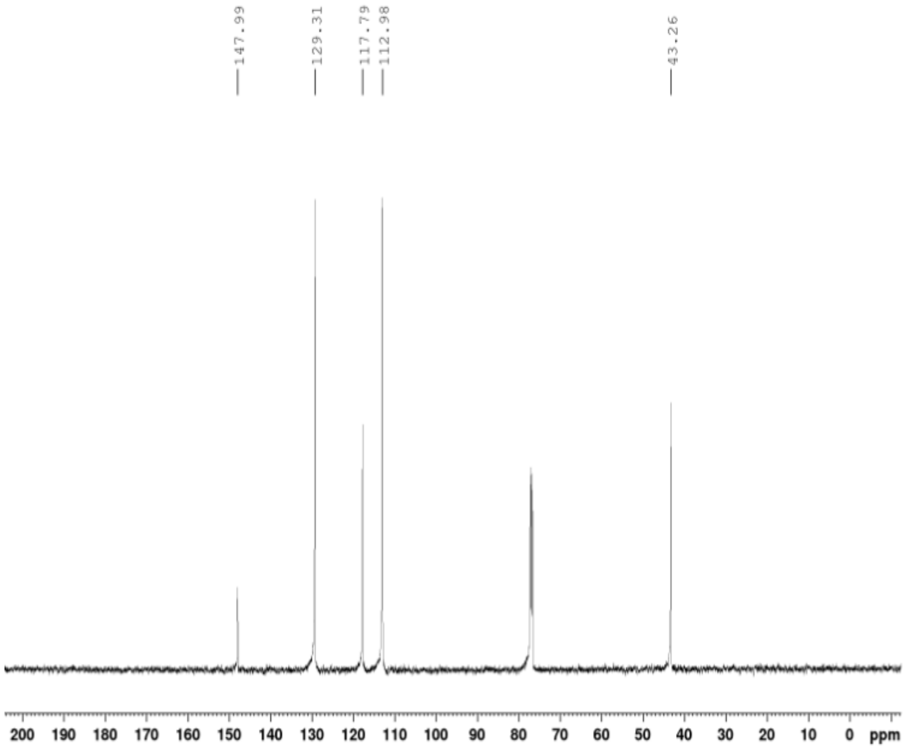

Supplementary Figure 133. ¹H NMR of *N*¹ -Phenyl-*N*² -(pyridin-3-yl)ethane-1,2-diamine, **4af** ($CDCl_3$, 300.1 MHz)

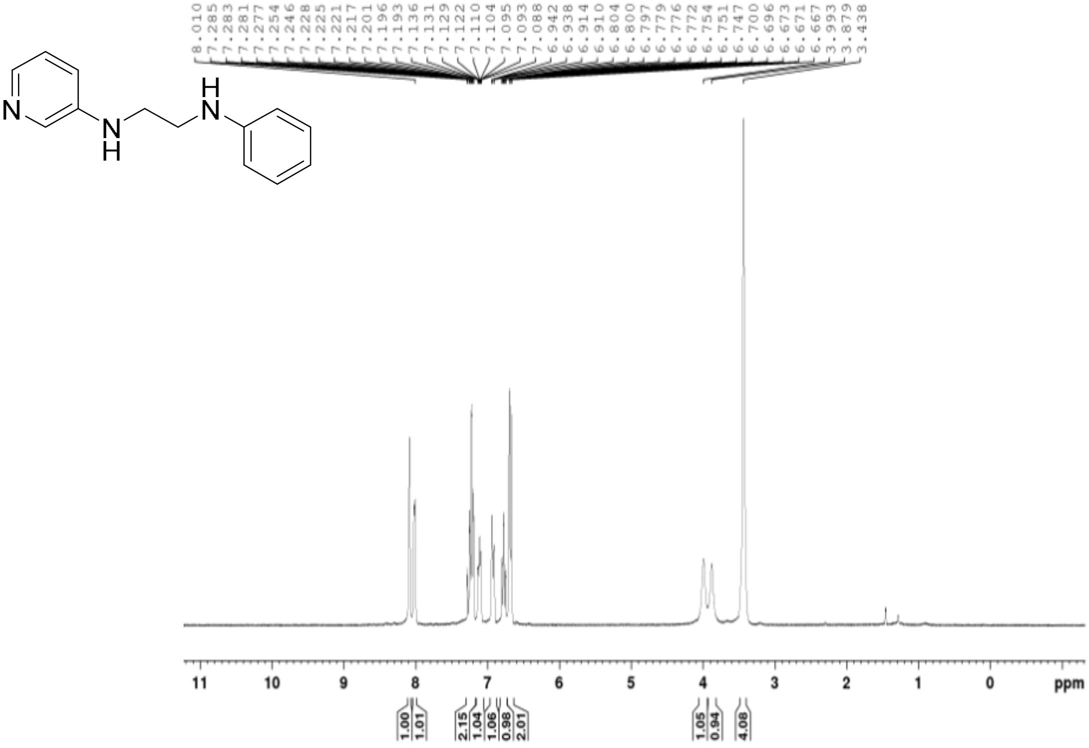

Supplementary Figure 134. ¹³C{¹H} NMR of *N*¹ -Phenyl-*N*² -(pyridin-3-yl)ethane-1,2-diamine, **4af** ($CDCl_3$, 125.8 MHz)

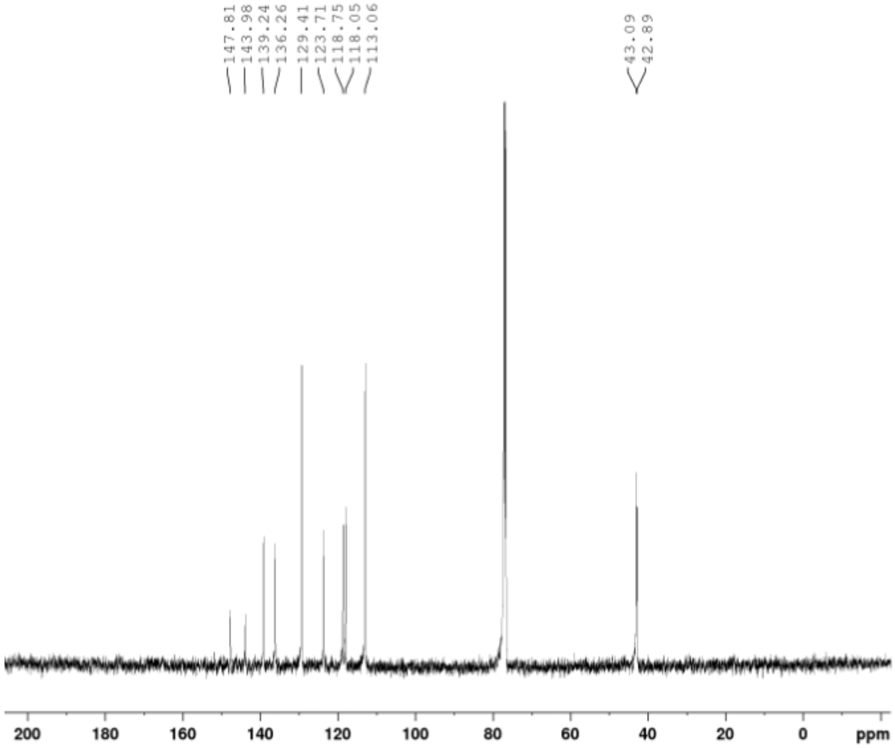

Supplementary Figure 135. ^{1}H NMR of 9-(4-methoxyphenyl)-9H-carbazole, **4ag** ($CDCl_3$, 300.1 MHz)

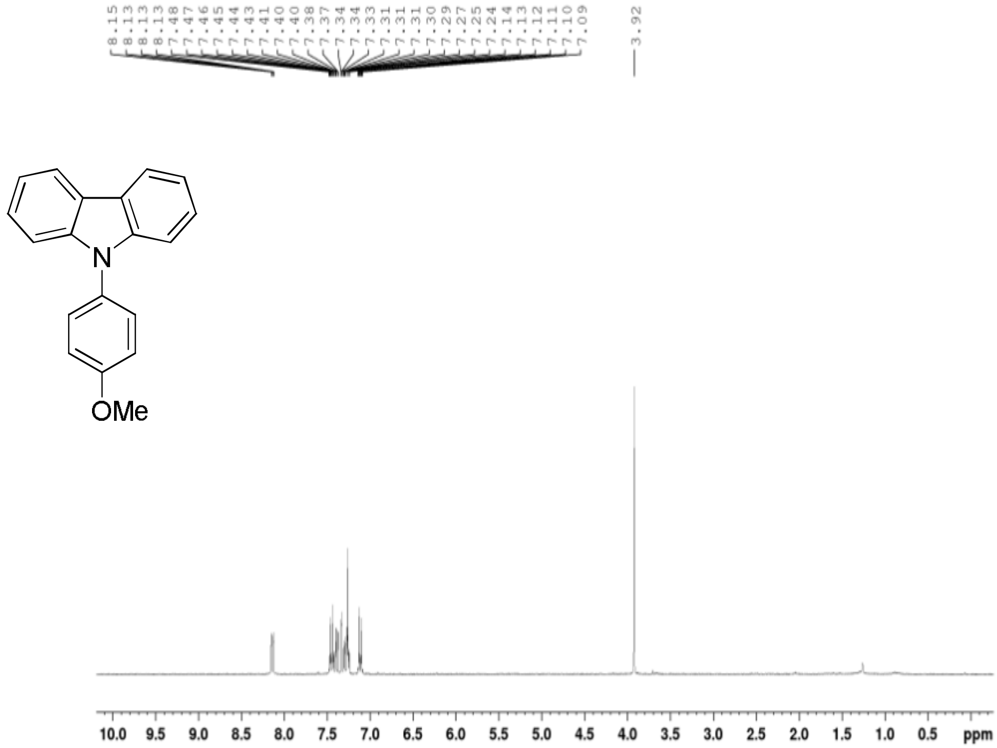

Supplementary Figure 136. $^{13}C\{^{1}H\}$ NMR of 9-(4-methoxyphenyl)-9H-carbazole, **4ag** ($CDCl_3$, 125.8 MHz)

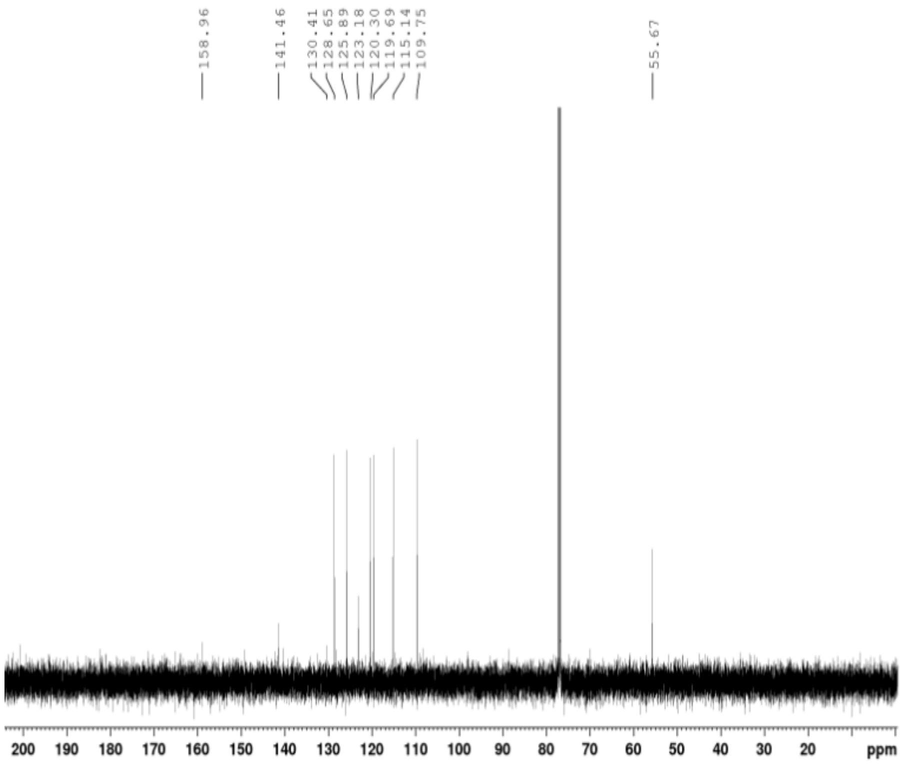

Supplementary Figure 137. ^{1}H NMR of 4-(9H-carbazol-9-yl)benzonitrile, **4ah** ($CDCl_3$, 300.1 MHz)

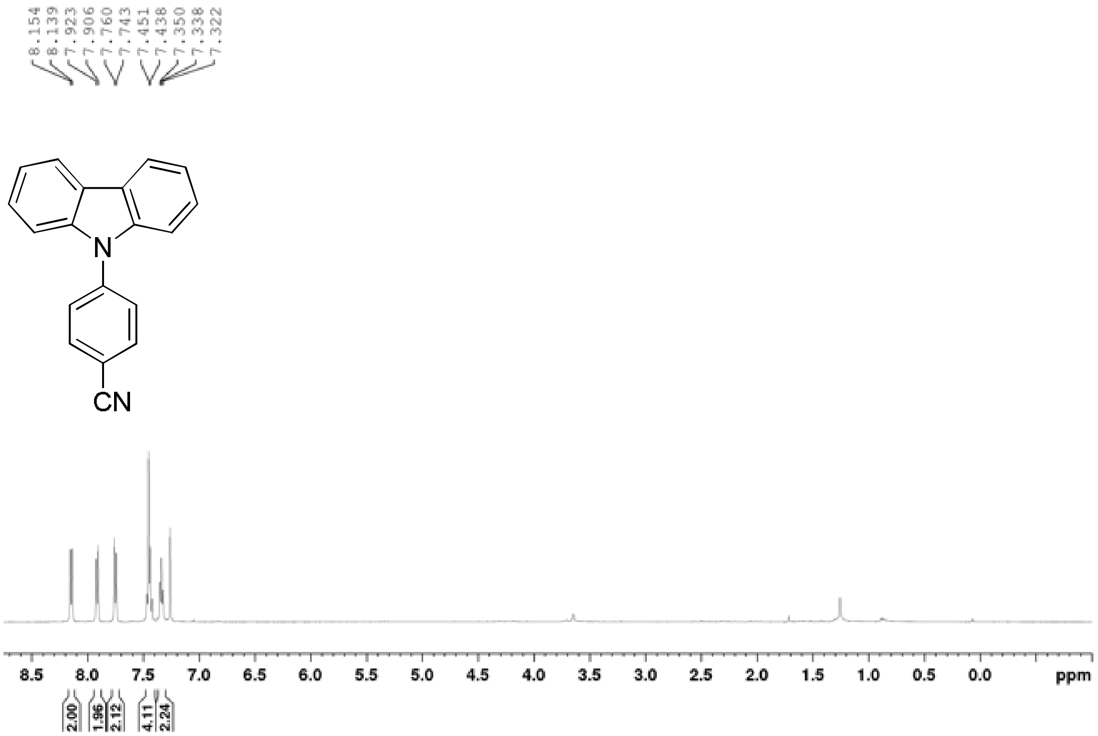

Supplementary Figure 138. ^{13}C{^{1}H} NMR of 4-(9H-carbazol-9-yl)benzonitrile, **4ah** ($CDCl_3$, 125.8 MHz)

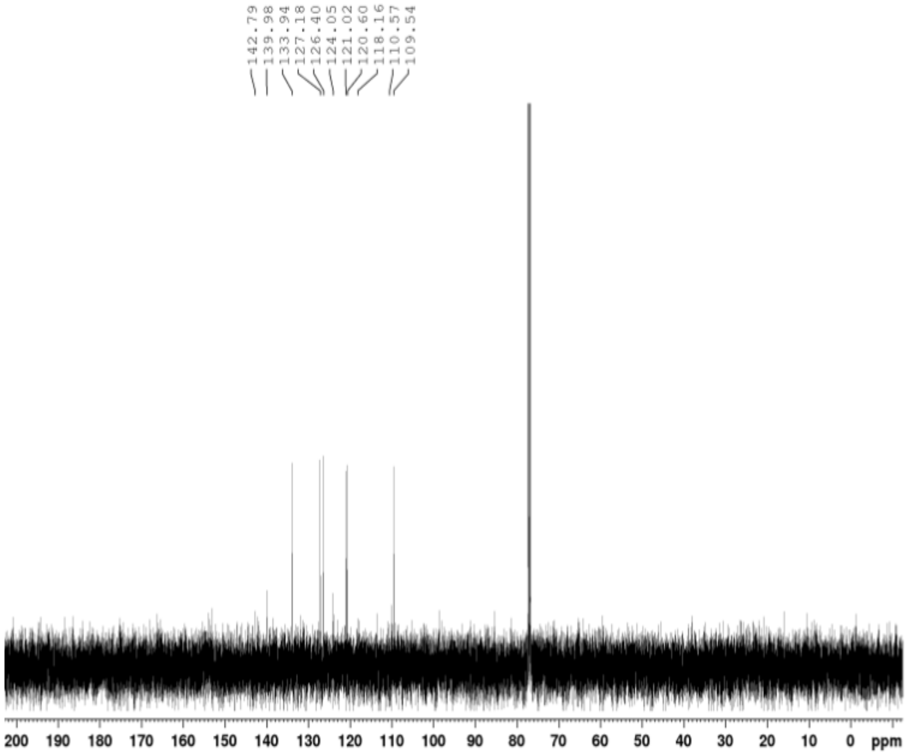

Supplementary Figure 139. ^{1}H NMR of 1-(4-Methoxy-phenyl)-1H-indole, **4ai** ($CDCl_3$, 300.1 MHz)

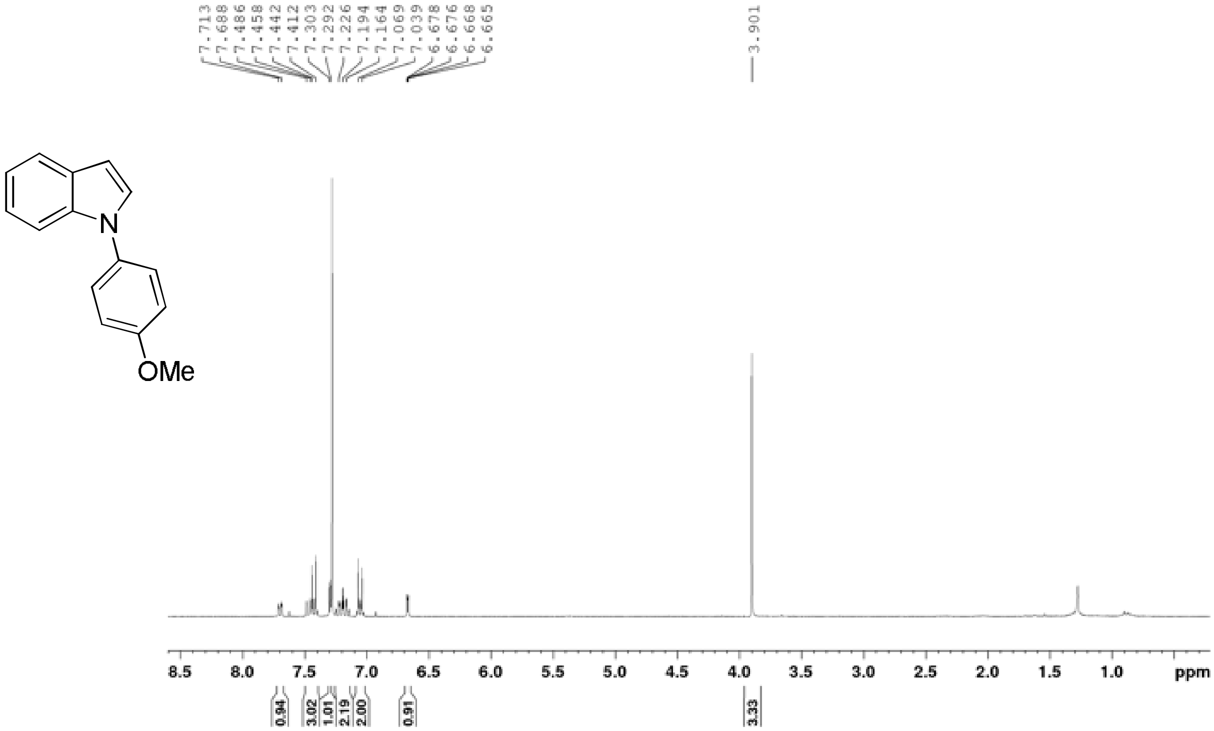

Supplementary Figure 140. ^{13}C{^{1}H} NMR of 1-(4-Methoxy-phenyl)-1H-indole, **4ai** ($CDCl_3$, 125.8 MHz)

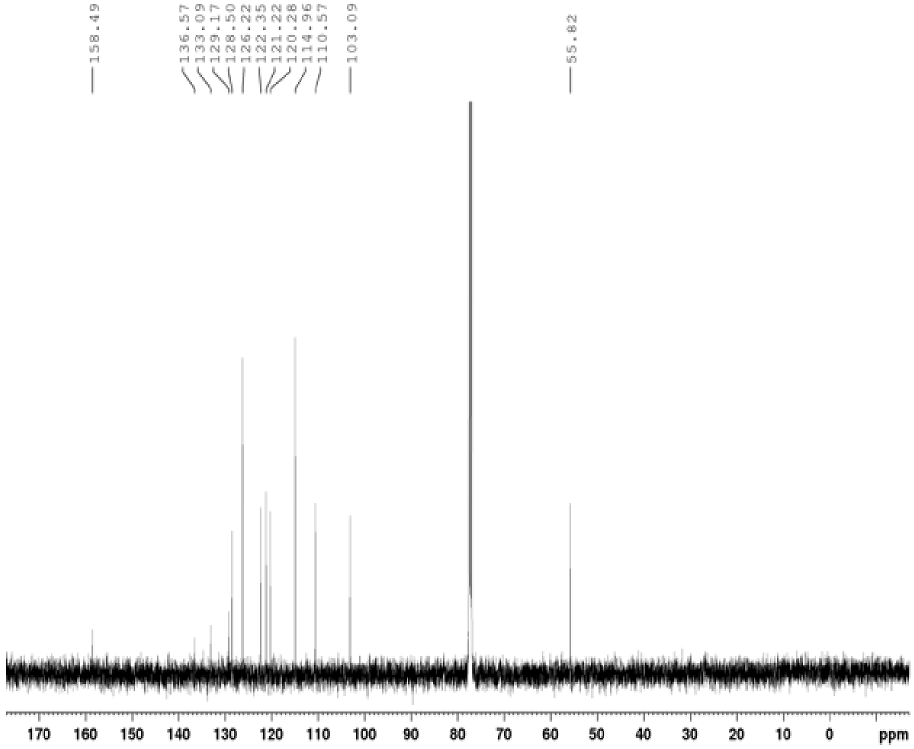

Supplementary Figure 141. ^{1}H NMR of 4-(1H-pyrrolo[2,3-b]pyridin-1-yl)benzonitrile, **4aj** ($CDCl_3$, 300.1 MHz)

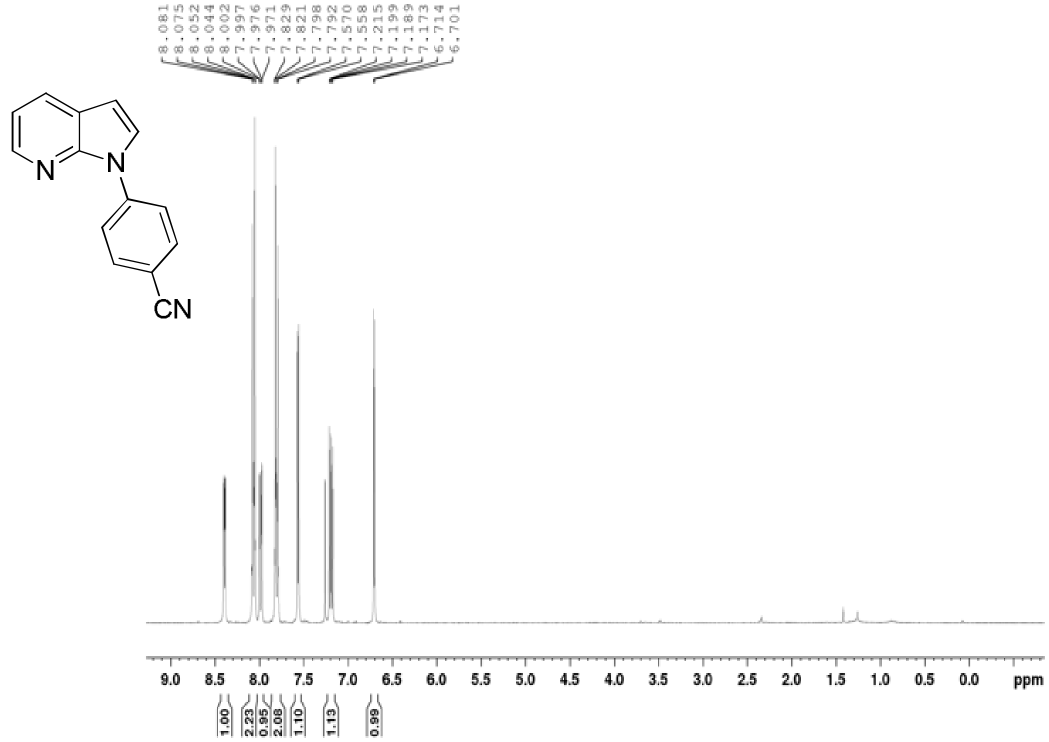

Supplementary Figure 142. $^{13}C\{^{1}H\}$ NMR of 4-(1H-pyrrolo[2,3-b]pyridin-1-yl)benzonitrile, **4aj** ($CDCl_3$, 125.8 MHz)

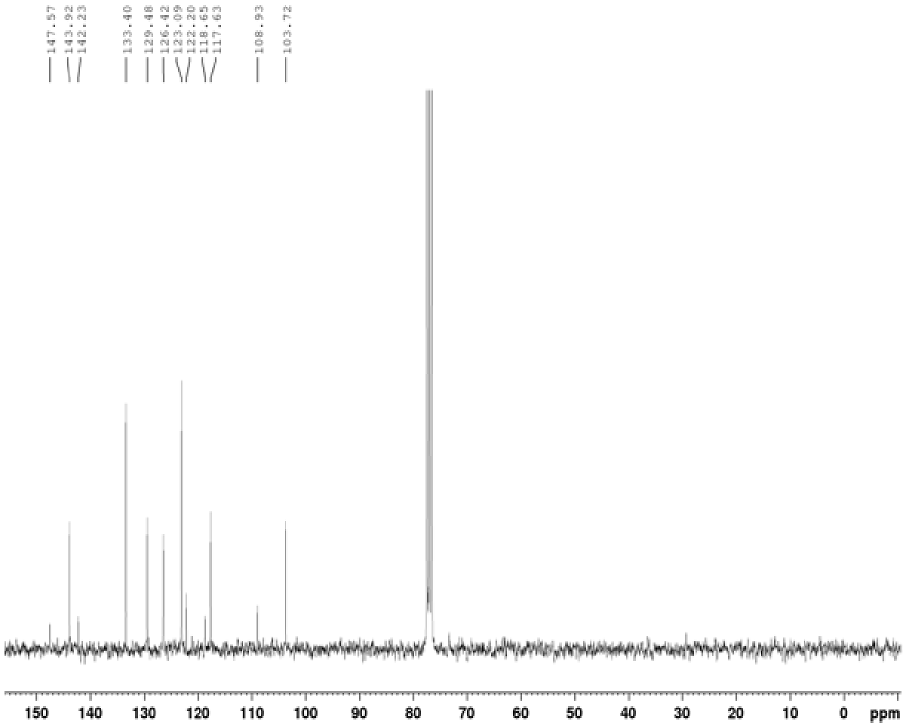

Supplementary Table 1. Crystallographic Experimental Details for [(**L1**)$NiCl_2$]•0.5CH_2Cl_2.

A. Crystal Data	
formula	$C_{30.5}H_{35}Cl_3NiO_3P_2$
formula weight	676.58
crystal dimensions (mm)	0.25 × 0.22 × 0.08
crystal system	triclinic
space group	$P\bar{1}$ (No. 2)
unit cell parameters	
a (Å)	8.8871 (7)
b (Å)	9.1067 (7)
c (Å)	20.1958 (16)
α (deg)	82.8202 (12)
β (deg)	83.4850 (11)
γ (deg)	72.4192 (10)
V (Å^3)	1541.0 (2)
Z	2
ρ_{calcd} (g cm^{-3})	1.458
μ (mm^{-1})	1.025
B. Data Collection and Refinement Conditions	
diffractometer	Bruker PLATFORM/APEX II CCD
radiation (λ [Å])	graphite-monochromated Mo Kα (0.71073)
temperature (°C)	–100
scan type	ω scans (0.3°) 15 s exposures)
data collection 2θ limit (deg)	55.04
total data collected	18661 ($-11 \le h \le 11$, $-11 \le k \le 11$, $-25 \le l \le 26$)
independent reflections	6841 (R_{int} = 0.0494)
number of observed reflections (*NO*)	5456 [$F_o^2 \ge 2\sigma(F_o^2)$]
structure solution method	Patterson/structure expansion (*DIRDIF–2008*)
refinement method	full-matrix least-squares on F^2 (*SHELXL–2014*)
absorption correction method	multi-scan (*TWINABS*)
range of transmission factors	0.9420–0.8508
data/restraints/parameters	6841 / 0 / 346
goodness-of-fit (S) [all data]	1.052
final R indices	
R_1 [$F_o^2 \ge 2\sigma(F_o^2)$]	0.0464
wR_2 [all data]	0.1152
largest difference peak and hole	1.122 and –0.469 e Å^{-3}

Supplementary Table 2. Crystallographic Experimental Details for **C1**•$0.5C_5H_{12}$•$0.5C_4H_8O$.

A. Crystal Data

formula	$C_{41.50}H_{51}ClNiO_{3.50}P_2$
formula weight	761.92
crystal dimensions (mm)	0.21 × 0.13 × 0.13
crystal system	monoclinic
space group	*C*2/*c* (No. 15)
unit cell parameters	
a (Å)	20.629 (4)
b (Å)	19.421 (4)
c (Å)	19.876 (4)
β (deg)	104.776 (3)
V ($Å^3$)	7700 (3)
Z	8
ρ_{calcd} (g cm^{-3})	1.315
μ (mm^{-1})	0.695

B. Data Collection and Refinement Conditions

diffractometer	Bruker D8/APEX II CCD
radiation (λ [Å])	graphite-monochromated Mo Kα (0.71073)
temperature (°C)	–100
scan type	ω scans (0.3°) (20 s exposures)
data collection 2θ limit (deg)	54.21
total data collected	33021 ($-26 \le h \le 26$, $-24 \le k \le 24$, $-25 \le l \le 25$)
independent reflections	8473 (R_{int} = 0.0381)
number of observed reflections (*NO*)	6451 [$F_o^2 \ge 2\sigma(F_o^2)$]
structure solution method	intrinsic phasing (*SHELXT-2014*)
refinement method	full-matrix least-squares on F^2 (*SHELXL–2014*)
absorption correction method	Gaussian integration (face-indexed)
range of transmission factors	0.9573–0.8867
data/restraints/parameters	8473 / 45 / 427
goodness-of-fit (*S*) [all data]	1.028
final *R* indices	
R_1 [$F_o^2 \ge 2\sigma(F_o^2)$]	0.0459
wR_2 [all data]	0.1344
largest difference peak and hole	1.029 and –0.751 e $Å^{-3}$

Supplementary Methods

I. Characterization of Isolated Amination Products

4-Phenylaniline (**2a**)

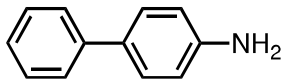

Following **GPA**: (1.8 mmol ammonia, 3 mol% **C1**, 2 eq. LiO*t*Bu) Purified by column chromatography (10:1, hexanes/EtOAc) to yield **2a** as a beige yellow solid in 63% yield from the corresponding bromide. ^{1}H NMR (300 MHz, $CDCl_3$): δ 7.58-7.56 (m, 2H), 7.46-7.40 (m, 4H), 7.32-7.28 (m, 1H), 6.80-6.77 (m, 2H), 3.72 (br s, 2H); ^{13}C{^{1}H} NMR (75.5 MHz, $CDCl_3$): δ 145.8, 141.2, 131.6, 128.7, 128.0, 126.4, 126.3, 115.4. Agrees with data previously reported in the literature.[1] Following **GPD**: (0.02 mmol ammonium acetate, 2 mol % **C1**, 140 °C, 5 minutes) the title compound was isolated in 76% yield from the corresponding chloride.

4'-Methylbiphenyl-4-amine (**2b**)

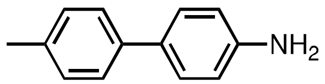

Following **GPA**: (1.8 mmol ammonia, 4 mol% **C1**) Purified by column chromatography (5:1, hexanes/EtOAc) to yield **2b** as a yellow solid in 78% yield from the corresponding chloride. ^{1}H NMR (500 MHz, $CDCl_3$): δ δ 7.48-7.43 (m, 4H), 7.25-7.23 (m, 2H), 6.79-6.78 (m, 2H), 3.89 (br s, 2H), 2.43 (s, 3H); ^{13}C{^{1}H} NMR (125.8 MHz, $CDCl_3$): δ 145.8, 138.5, 136.1, 131.9, 129.7, 128.0, 126.5, 115.6, 21.2. Agrees with data previously reported in the literature.[1]

4'-Methoxybiphenyl-4-amine (**2c**)

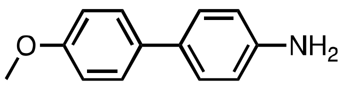

Following **GPA**: (1.8 mmol ammonia, 4 mol% **C1**) Purified by column chromatography (5:1, hexanes/EtOAc) to yield as a yellow solid in 86% yield from the corresponding chloride. ^{1}H NMR (500 MHz, $CDCl_3$): δ 7.50-7.48 (m, 2H), 7.40-7.39 (m, 2H), 6.99-6.97 (m, 2H), 6.79-6.78 (m, 2H), 3.87 (s, 3H), 3.73 (br s, 2H); ^{13}C{^{1}H} NMR (125.8 MHz, $CDCl_3$): δ 158.7, 145.5, 134.1, 131.6, 127.8, 127.6, 115.7, 114.3, 55.6. Agrees with data previously reported in the literature.[1] Following **GPE** the title compound was generated in 60% yield from the corresponding chloride on the basis of NMR integration using ferrocene as an internal standard.

Naphthalen-1-amine (**2d**)

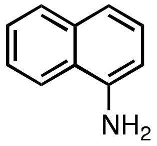

Following **GPA**: (1.8 mmol ammonia, 2 mol% **C1**, 25 °C) Purified by column chromatography (10:1, hexanes/EtOAc) to yield as a purple solid in 89% yield from the corresponding chloride. ^{1}H NMR (500 MHz, $CDCl_3$): δ 7.88-7.84 (m, 2H), 7.51-7.49 (m, 2H), 7.36-7.29 (m, 2H), 6.82 (m, 1H), 4,17 (br s, 2H); ^{13}C{ ^{1}H} NMR (125.8 MHz, $CDCl_3$): δ 142.3, 134.6, 128.8, 126.5, 126.0, 125.1, 123.9, 121.0, 109.1. Agrees with data previously reported in the literature.[1] Following **GPD**: (5 mmol ammonium acetate, 1 mol % **C1**, 110 ˚C, 5 minutes) the title compound was isolated in 99% yield from the corresponding chloride. Following **GPE**: the title compound was generated in 85% yield from the corresponding chloride on the basis of NMR integration using ferrocene as an internal standard. Following **GPA**: (3.0 mmol ammonia, 3 mol % **C1**, 25 ˚C) the title compound was isolated in 73% yield from the corresponding bromide. Following **GPA**: (3.0 mmol ammonia, 4 mol% **C1**) the title compound was isolated in 68% yield from the corresponding iodide. Following **GPA**: (4.2 mmol ammonia, 4 mol % **C1**) the title compound was isolated in 43% yield from the corresponding triflate. Following **GPA**: (4.2 mmol ammonia, 5 mol% **C1**, 2 equiv LiO*t*Bu) the title compound was isolated in 59% yield from the corresponding imidazolyl sulfonate. Following **GPC**: (5 mol% **C1**, 8.3 equiv ammonia) the title compound was isolated in 71% yield from the corresponding mesylate. Following **GPC**: (5 mol% **C1**, 4.2 equiv ammonia, [aryl mesylate] = 0.12 M) the title compound was generated in 60% yield at 25 ˚C from the corresponding mesylate on the basis of NMR integration using ferrocene as an internal standard.

Naphthalen-2-amine (**2e**)

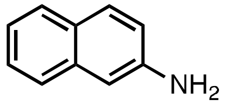

Following **GPA**: (3.0 mmol ammonia, 2 mol% **C1**, 25 °C) Purified by column chromatography (5:1, hexanes/EtOAc) to yield **2e** as a purple solid in 76% yield from the corresponding tosylate. ^{1}H NMR (500 MHz, $CDCl_3$): δ 7.77-7.69 (m, 2H), 7.64-7.62 (d, 1H), 7.42-7.39 (m, 1H), 7.28-7.25 (m, 1H), 7.03-7.02 (m, 1H), 6.99-6.98 (m, 1H), 3.88 (br s, 2H); ^{13}C{ ^{1}H} NMR (125.8 MHz, $CDCl_3$): δ 144.2, 135.1, 129.4, 128.2, 127.9, 126.7, 126.0, 122.7, 118.4, 108.9. Agrees with data previously reported in the literature.[2]

[1,1'-Biphenyl]-2-amine (**2f**)

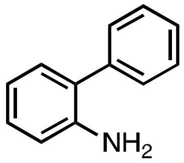

Following **GPA**: (3.0 mmol ammonia, 3 mol % **C1**, 25 °C) Purified by column chromatography (10:1, hexanes/EtOAc) to yield **2f** as a solid in 83% yield from the corresponding bromide. ^{1}H NMR (300 MHz, $CDCl_3$): δ 7.48-7.47 (m, 4H), 7.39-7.33 (m, 1H), 7.19-7.14 (m, 2H), 6.88-6.78 (m, 2H), 3.78 (br s, 2H); ^{13}C{^{1}H} NMR (125.8 MHz, $CDCl_3$): δ 143.7, 139.8, 130.7, 129.3, 129.0, 128.7, 127.9, 127.4, 118.9, 115.8. Agrees with data previously reported in the literature.[3] Following **GPC**: (5 mol% **C1**, 8.3 equiv ammonia) the title compound was generated in 58% yield from the corresponding mesylate on the basis of NMR integration using ferrocene as an internal standard.

4-(1H-Pyrrol-1yl)aniline, (**2g**)

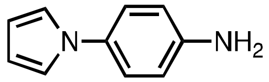

Following **GPA**: (3.0 mmol ammonia, 4 mol% **C1**, 2 equiv LiO*t*Bu) Purified by column chromatography (10:1, hexanes/EtOAc) to yield **2g** as a light brown solid in 76% yield from the corresponding chloride. ^{1}H NMR (500 MHz, $CDCl_3$): δ 7.23-7.22 (m, 2H), 7.02-7.01 (m, 2H), 6.77-6.75 (m, 2H), 6.35-6.34 (m, 2H), 3.76 (br s, 2H); ^{13}C{ ^{1}H} NMR (125.8 MHz, $CDCl_3$): δ 144.7, 133.1, 122.6, 119.9, 115.9, 109.7. Agrees with data previously reported in the literature.[1] Following **GPD**: (2 mol % **C1**, 140 °C, 20 minutes) the title compound was isolated in 56% yield from the corresponding chloride.

2,5-Dimethylaniline (**2h**)

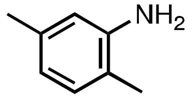

Following **GPA**: (3.0 mmol ammonia, 4 mol% **C1**, 25 °C) Purified by column chromatography (10:1, hexanes/EtOAc) to yield **2h** as a colorless oil in 89% yield from the corresponding chloride. ^{1}H NMR (500 MHz, $CDCl_3$): δ 7.01 (d, J = 7.5 Hz, 1H), 6.62 (d, J = 8.0 Hz, 1H), 6.57 (s, 1H), 3.61 (br s, 2H), 2.33 (s, 3H), 2.20 (s, 3H); ^{13}C{ ^{1}H} NMR (125.8 MHz, $CDCl_3$): δ 144.6, 136.8, 130.5, 119.5, 115.9, 21.2, 17.0. Agrees with data previously reported in the literature.[2] Following **GPA**: (3.0 mmol ammonia, 4 mol% **C1**, 25 °C) the title compound was isolated as a colourless oil in 78% yield from the corresponding bromide. Following **GPC**: (5 mol% **C1**, 8.3 equiv ammonia) the title compound was generated in 58% yield from the corresponding mesylate on the basis of NMR integration using ferrocene as an internal standard.

3-Amino-4-methylbenzonitrile (**2i**)

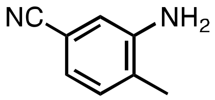

Following **GPA**: (3.0 mmol ammonia, 3 mol% **C1**, 25 °C) Purified by extraction with 1.0 M aqueous HCl to yield **2i** as a solid in 70% yield from the corresponding bromide. ^{1}H NMR (300 MHz, $CDCl_3$): δ 7.14-7.12 (m, 1H), 7.01-7.00 (m, 1H), 6.93 (s, 1H), 3.82 (br s, 2H), 2.22 (s, 3H); ^{13}C{ ^{1}H} NMR (125.8 MHz, $CDCl_3$): δ 145.2, 131.3, 127.9, 122.5, 119.6, 117.5, 110.6, 17.9. Data agrees with commercial source material (CAS: 60710-80-7).

N-(5-Fluoro-2-methylphenyl)-4-methylbenzenesulfonamide (**2j**)

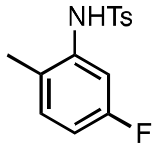

Following **GPD**: (5 mmol ammonium acetate, 5 mol% **C1**, 140 ˚C, 20 minutes). The crude product was tosylated using a literature procedure[1] to yield **2j** as a white solid in 63% yield. ^{1}H NMR (300 MHz, $CDCl_3$): δ 7.70-7.65 (m, 2H), 7.28-7.25 (m, 2H), 7.19-7.15 (m, 1H), 7.06-7.01 (m, 1H), 6.80-6.73 (m, 1H), 6.57 (br s, 1H), 2.41 (s, 3H), 1.99 (s. 3H); ^{13}C{^{1}H} NMR (75.5 MHz, $CDCl_3$): δ 161.3 (J_{CF} = 244.0 Hz), 144.1, 136.4, 135.6 (J_{CF} = 10.4 Hz), 129.7, 127.1, 125.2 (J_{CF} = 3.1 Hz), 112.3 (J_{CF} = 21.1 Hz), 110.1 (J_{CF} = 25.3 Hz), 21.5, 16.8; HRMS *m/z* ESI$^+$ found 302.0621 [M + Na]$^+$ calculated for $C_{14}H_{14}FNO_2SNa$ 302.0627.

2-Anisidine (**2k**)

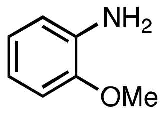

Following **GPA**: (3.0 mmol ammonia, 3 mol% **C1**) Purified by extraction with 1.0 M aqueous HCl to yield **2k** as a brown oil in 80% yield (ca. 90% purity) from the corresponding chloride. ^{1}H NMR (300 MHz, $CDCl_3$): δ 6.85-6.76 (m, 4H), 3.96-3.89 (m, 5H); ^{13}C{^{1}H} NMR (125.8 MHz, $CDCl_3$): δ 147.6, 136.2, 121.3, 118.8, 115.3, 110.7, 55.8. Agrees with data previously reported in the literature.[2] Following **GPD**: (5 mmol ammonium acetate, 5 mol% **C1**, 160 °C, 30 minutes) The crude product was tosylated using a literature procedure[1] to yield **2k** as a brown oil in 57% yield. ^{1}H NMR (300 MHz, $CDCl_3$): δ 7.68-7.65 (m, 2H), 7.55-7.52 (m, 1H), 7.22-7.19 (m, 2H), 7.07-7.01 (m, 2H), 6.94-6.88 (m, 1H), 6.76-6.73 (m, 1H), 3.66 (s, 3H), 2.37 (s. 3H); ^{13}C{^{1}H} NMR (75.5 MHz, $CDCl_3$): δ 149.4, 143.6, 136.4, 129.3, 127.3, 126.1, 125.2, 121.1, 121.0, 110.6, 55.6, 21.5.

3-Anisidine (**2l**)

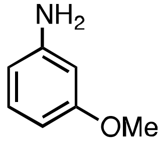

Following **GPA**: (1.8 mmol ammonia, 3 mol% **C1**) Purified by extraction with 1.0 M aqueous HCl to yield **2l** as a brown oil in 60% yield (ca. 90% purity) from the corresponding chloride. ^{1}H NMR (300 MHz, $CDCl_3$): δ 7.12-7.09 (m, 1H), 6.39-6.33 (m, 2H), 6.29 (s, 1H), 3.80 (s, 3H), 3.74 (br s, 2H); ^{13}C{^{1}H} NMR (125.8 MHz, $CDCl_3$): δ 160.9, 147.7, 130.3, 108.3, 104.4, 101.4, 55.3. Agrees with data previously reported in the literature.[4] Following **GPA** (3.0 mmol ammonia, 3 mol% **C1**): Purified by extraction with 1.0 M aqueous HCl to yield the title compound in 70% yield (ca. 90% purity) from the corresponding bromide.

2-(2-Methyl-1,3-dioxolan-2-yl)aniline (**2m**)

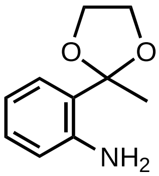

Following **GPA**: (5 mmol ammonia, 5 mol% **C1**) Purified by preparatory TLC (10:1 EtOAc/NEt_3) to yield **2m** as a yellow oil in 83% yield from the corresponding chloride. ^{1}H NMR (500 MHz, $CDCl_3$): 7.39-7.37 (m, 1H), 7.14-7.11 (m, 1H), 6.77-6.74 (m, 1H), 6.68-6.66 (m, 1H), 4.32 (br s, 2H), 4.12-4.05 (m, 2H), 3.90-3.83 (m, 2H), 1.75 (s, 3H); ^{13}C{^{1}H} NMR (125.8 MHz, $CDCl_3$): 144.7, 129.5, 127.0, 126.3, 118.2, 117.2, 109.9, 64.5, 24.9; HRMS *m/z* ESI^{+} found: 180.1024 [M+H]$^{+}$ calculated for $C_{10}H_{14}NO_2$ 180.1019.

4-Aminobenzophenone (**2n**)

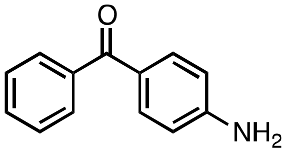

Following **GPD**: (5 mmol ammonium acetate, 5 mol% **C1**, 140 °C, 5 minutes). Purified by column chromatography (0% EtOAc/100% hexanes to 50/50% EtOAc/hexanes gradient) to yield **2n** as a white solid in 60% yield from the corresponding chloride. ^{1}H NMR (300 MHz, DMSO): δ 7.57-7.62 (m, 3H), 7.54-7.56 (m, 1H), 7.51-7.52 (m, 2H), 7.48-7.50 (m, 1H), 6.57-6.64 (m, 2H), 6.14 (br s, 2H); ^{13}C{^{1}H} NMR (75.5 MHz, DMSO); δ 193.3, 153.7, 139.0, 132.5, 130.9, 128.7, 128.1, 123.6, 112.5. Agrees with data previously reported in the literature.[3]

4-Amino-α-methylstyrene (**2o**)

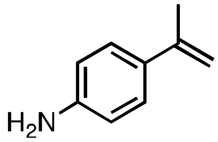

Following **GPD**: (5 mmol ammonium acetate, 3 mol% **C1**, 140 °C, 20 minutes). Purified by column chromatography (0% EtOAc/100% hexanes to 30/70% EtOAc/hexanes gradient) to yield **2o** as a brown oil in 65% yield from the corresponding chloride. ^{1}H NMR (300 MHz, $CDCl_3$): δ 7.30-7.36 (m, 2H), 6.63-6.70 (m, 2H), 5.25-5.29 (m, 1H), 4.92-4.97 (m, 1H), 3.68 (br s, 2H), 2.13 (s, 3H); ^{13}C{^{1}H} NMR (75.5 MHz, $CDCl_3$): δ 145.8, 142.7, 131.6, 126.4, 114.7, 109.3, 21.8. Agrees with data previously reported in the literature.[3]

4-Aminoisoquinoline (**2p**)

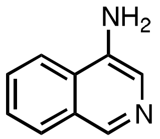

Following **GPB**: Purified by column chromatography (10:1:0.1, hexanes/EtOAc/NH*i*Pr_2) to yield **2p** as a light brown solid in 81% yield from the corresponding bromide. ^{1}H NMR (500 MHz, $CDCl_3$): δ 8.69-8.68 (m, 1H), 7.93 (t, *J* = 9 Hz, 2H), 7.31-7.28 (m, 1H), 7.20-7.18 (m, 1H), 6.93-6.92 (m, 1H) 4.00 (br s, 2H) ; ^{13}C{^{1}H} NMR (125.8 MHz, $CDCl_3$): δ 147.1, 144.8, 143.7, 134.0, 130.8, 130.0, 121.8, 121.6, 107.6. Agrees with data previously reported in the literature.[1]

6-Aminoquinoline (**2q**)

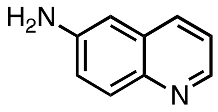

Following **GPB**: Purified by preparatory TLC (7:2:1, hexanes/EtOAc/NH*i*Pr_2) to yield **2q** as a white solid in 82% yield from the corresponding chloride. ^{1}H NMR (500 MHz, $CDCl_3$): δ 8.79 (s, 1H), 8.08 (s, 1H) 7.96 (d, *J* = 8.2 Hz, 1H), 7.85 (d, 8.4 Hz, 1H), 7.73-7.72 (m, 1H), 4.12 (br s, 2H); ^{13}C{^{1}H} NMR (125.8 MHz, $CDCl_3$): δ 143.7, 136.9, 129.3, 128.9, 128.6, 128.1, 127.3, 126.4, 120.2. Agrees with data previously reported in the literature.[1]

8-Aminoquinoline (**2r**)

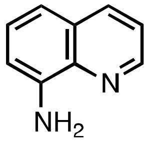

Following **GPC**: Purified by column chromatography (50% DCM/hexanes to 10% NEt_3/hexanes) followed by an acidic work up with ethyl acetate, 1 M aqueous HCl and distilled water. The organic fractions were combined, dried over Na_2SO_4 and concentrated under reduced pressure to yield **2r** as an orange oil in 53% yield from the corresponding mesylate. ^{1}H NMR (500 MHz, $CDCl_3$): δ 8.81-8.79 (m, 1H), 8.11-8.10 (m, 1H), 7.41-7.36 (m, 2H), 7.20-7.18 (m, 1H), 6.98-6.96 (m, 1H), 5.02 (br s, 2H); ^{13}C{^{1}H} NMR (125.8 MHz, $CDCl_3$): δ 147.6, 144.2, 138.7, 136.2, 129.1, 127.6, 121.5, 116.3, 110.2. Agrees with data previously reported in the literature.[4]

4-Aminoquinaldine (**2s**)

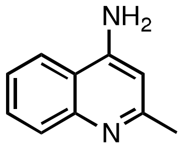

Following **GPE**: Purified by column chromatography (7:2:1, hexanes/EtOAc/NH*i*Pr_2) to yield **2s** as an light yellow solid in 87% yield from the corresponding chloride. ^{1}H NMR (500 MHz, $CDCl_3$): δ 7.92 (d, J = 8.5 Hz, 1H), 7.72-7.70 (m, 1H) , 7.63-7.59 (m, 1H), 7.40-7.36 (m, 1H), 6.50 (s, 1H), 4.68 (br s, 2H), 2.58 (s, 3H) ; ^{13}C{^{1}H} NMR (125.8 MHz, $CDCl_3$): δ 159.5, 149.8, 18.9, 129.6, 129.3, 124.3, 120.2, 117.6, 25.5. Agrees with data previously reported in the literature.[1] Following **GPD**: (0.5 mmol ammonium acetate, 1 mol% **C1**, 140 °C, 5 minutes) the title compound was isolated in 78% yield from the corresponding chloride.

2-Amino-4,6-dimethoxypyrimidine (**2t**)

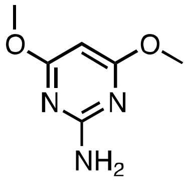

Following **GPB**: Purified by preparatory TLC (10% NH*i*Pr_2/hexanes) to yield **2t** as a white solid in 71% yield from the corresponding chloride. ^{1}H NMR (500 MHz, $CDCl_3$): δ 5.49 (s, 1H), 4.96 (br s, 2H), 3.87 (s, 6H); ^{13}C{^{1}H} NMR (125.8 MHz, $CDCl_3$): δ 172.7, 162.5, 79.9, 53.9. Agrees with data previously reported in the literature.[5]

2-Aminoquinazoline (**2u**)

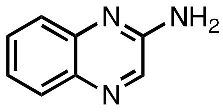

Following **GPE**: Purified by column chromatography (8:2:0.2, hexanes/EtOAc/NH*i*Pr_2) to yield **2u** as a yellow solid in 76% yield from the corresponding chloride. ^{1}H NMR (500 MHz, $CDCl_3$): δ 8.33 (s, 1H), 7.92 (d, J = 9.6 Hz, 1H), 7.67-7.66 (m, 1H), 7.62-7.59 (m, 1H), 7.46-7.43 (m, 1H), 7.26 (s, 1H), 4.93 (s, 2H); ^{13}C{^{1}H} NMR (125.8 MHz, $CDCl_3$): δ 152.1, 141.7, 138.1, 137.9, 130.7, 129.3, 126.5, 125.6. Agrees with data previously reported in the literature.[1]

5-Aminobenzothiophene (**2v**)

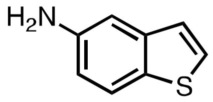

Following **GPE**: Purified by column chromatography (9:1:0.1, hexanes/EtOAc/NH*i*Pr_2) to yield **2v** as a white solid in 68% yield from the corresponding chloride. ^{1}H NMR (500 MHz, $CDCl_3$): δ 7.67 (d, J = 8.6 Hz, 1H), 7.42 (d, J = 5.4 Hz, 1H), 7.19 (d, J = 5.4 Hz, 1H), 7.15-7.14 (m, 1H), 6.83-6.81 (m, 1H), 3.73 (s, 2H); ^{13}C{^{1}H} NMR (125.8 MHz, $CDCl_3$): δ 143.8, 141.1, 130.7, 127.3, 123.3, 123.2, 115.1, 108.5. Agrees with data previously reported in the literature.[1]

5-Amino-2-methylbenzothiazole (**2w**)

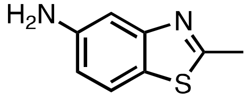

Following **GPB**: (5 mol% **C1**) Purified by column chromatography (5:5:0.1, hexanes/EtOAc/NH*i*Pr$_2$) to yield **2w** as a white solid in 77% yield from the corresponding chloride. ^{1}H NMR (500 MHz, CDCl$_3$): δ 7.58 (d, J = 8.5 Hz, 1H), 7.28-7.27 (m, 1H), 6.80-6.67 (m, 1H), 3.82 (br s, 2H), 2.82 (s, 3H); ^{13}C{^{1}H} NMR (125.8 MHz, CDCl$_3$): δ 168.0, 155.1, 145.5, 125.7, 121.9, 114.7, 107.7, 20.3. Agrees with data previously reported in the literature.[1]

4-Amino-6,7-dimethoxyquinazoline (**2x**)

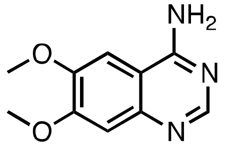

Following **GPE**: Purified by column chromatography (5:5:1, hexanes/EtOAc/NH*i*Pr$_2$) to yield **2x** as a beige-yellow solid in 90% yield from the corresponding chloride. ^{1}H NMR (500 MHz, DMSO): δ 8.25 (s, 1H), 7.67 (s, 1H), 7.46 (br s, 2H), 7.06 (s, 1H), 3.89 (s, 3H), 3.88 (s, 3H); ^{13}C{^{1}H} NMR (125.8 MHz, DMSO): δ 160.4, 154.0, 153.8, 148.1, 146.6, 108.0, 106.7, 102.9, 56.1, 55.6; Agrees with data previously reported in the literature.[5]

9-Amino-1,2,3,4-tetrahydroacridine (**2y**)

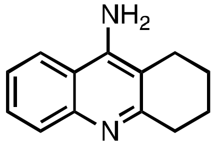

Following **GPB**: (5 mol% **C1**) Purified by column chromatography (6:4:1, EtOAc/hexanes/NH*i*Pr$_2$) to yield **2y** as a beige solid in 87% yield from the corresponding chloride. ^{1}H NMR (500 MHz, CDCl$_3$): δ 7.91-7.88 (m, 1H), 7.72-7.69 (m, 1H), 7.59-7.53 (m, 1H), 7.39-7.33 (m, 1H), 4.72 (br s, 2H), 3.05-3.01 (m, 2H), 2.63-2.59 (m, 2H), 1.99-1.88 (m, 4H); ^{13}C{^{1}H} NMR (125.8 MHz, CDCl$_3$): δ 158.6, 146.8, 146.5, 128.8, 128.7, 124.1, 119.9, 117.3, 110.6, 34.1, 23.9, 23.0, 22.9. Agrees with data previously reported in the literature.[6]

3-Aminoestrone (**2z**)

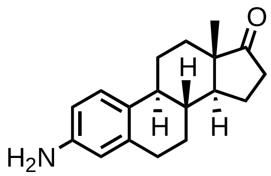

Following **GPB**: Purified by column chromatography (30% EtOAc/hexanes) to yield as a white solid in 58% yield **2z** from the corresponding triflate. ^{1}H NMR (500 MHz, $CDCl_3$): δ 7.12 (d, *J* = 8.3 Hz, 1H), 6.57-6.55 (m, 1H), 6.49-6.48 (m, 1H), 3.56 (br s, 2H), 2.89-2.86 (m, 2H), 2.56-2.51 (m, 1H), 2.43-2.39 (m, 1H), 2.28-2.23 (m, 1H), 2.21-2.13 (m, 1H), 2.11-2.06 (m, 1H), 2.04-1.96 (m, 2H), 1.70-1.41 (m, 7H), 0.94 (s, 3H); ^{13}C{^{1}H} NMR (125.8 MHz, $CDCl_3$): δ 221.2, 144.4, 137.6, 130.3, 126.4, 115.6, 113.3, 50.7, 48.3, 44.2, 38.7, 36.1, 31.8, 29.7, 26.8, 26.2, 21.8, 14.1. Agrees with data previously reported in the literature.[7]

N-(1-Naphthyl)-octylamine (**4a**)

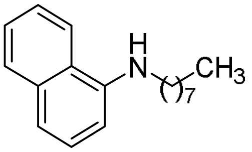

Following **GPF**: (0.50 mmol 1-chloro-naphthylene, 0.55 mmol octylamine, 1 mol% C1, 25 °C) the title product was isolated as a yellow oil in 96% yield. A 1% ethyl acetate/hexanes eluent system was used for column chromatography on silica gel. ^{1}H NMR (500 MHz, $CDCl_3$): δ 7.85-7.82 (m, 2H), 7.50-7.45 (m, 2H), 7.41-7.38 (m, 1H), 7.30-7.26 (m, 1H), 6.66 (d, *J* = 8.5 Hz, 1H), 4.35 (br s, 1H), 3.32-3.30 (m, 2H), 1.85-1.80 (m, 2H), 1.57-1.51 (m, 2H), 1.45-1.35 (m, 8H) 0.96 (t, *J* = 6.8 Hz, 3H); ^{13}C{^{1}H} NMR (125.8 MHz, $CDCl_3$): δ 143.9, 134.6, 128.9, 126.9, 125.9, 124.8, 123.6, 120.0, 117.4, 104.5, 44.6, 32.3, 29.7, 29.6, 27.6, 22.9, 14.4. Spectral data are consistent with the literature.[8] Following **GPF** (25 °C) the title product was isolated as a yellow oil in 94% yield from the corresponding bromide. Following **GPH** the title product was isolated as a yellow oil in 91% yield from the corresponding mesylate (5 mol% **C1**).

N-Methylnaphthalen-1-amine (**4b**)

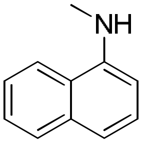

Following **GPD**: (1.0 mmol 1-chloronaphthalene, 5.0 mmol methylammonium chloride, 1 mol% **C1**, 110 °C, 5 minutes). The title product was isolated as a brown oil in 99% yield. ^{1}H NMR (300 MHz, $CDCl_3$): δ 7.79-7.88 (m, 2H), 7.40-7.54 (m, 3H), 7.28-7.33 (m, 1H), 6.65 (d, *J* = 7.5 Hz, 1H), 4.48 (br s, 1H), 3.05 (s, 3H); ^{13}C{^{1}H} (75.5 MHz, $CDCl_3$): δ 144.5, 134.2, 128.6, 126.7, 125.7, 124.7, 123.4, 119.8, 117.3, 103.8, 31.0. Spectral data are in agreement with the literature.[3]

N-Ethylnaphthalen-1-amine (**4c**)

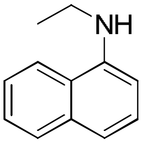

Following **GPD**: (1.0 mmol 1-chloronaphthalene, 5.0 mmol ethylammonium chloride, 1 mol% **C1**, 110 °C, 5 minutes): The title product was isolated as a dark brown oil in 99% yield. ^{1}H NMR (300 MHz, $CDCl_3$): δ 7.81-7.88 (m, 2H), 7.37-7.53 (m, 3H), 7.29 (d, *J* = 7.9 Hz, 1H) 6.67 (d, *J* = 6.9 Hz, 1H), 4.33 (br s, 1H), 3.37 (q, *J* = 7.1 Hz, 2H), 1.45 (t, *J* = 7.1, 3H); ^{13}C{^{1}H} (75.5 MHz, $CDCl_3$): δ 143.5, 134.3, 128.6, 126.6, 125.6, 124.6, 123.3, 119.8, 117.2, 104.3, 38.7, 14.8. Spectral data are in agreement with the literature.[3]

1-(*N*,*N*-Dimethylamino)naphthalene (**4d**)

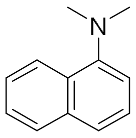

Following **GPG**: (0.60 mmol 1-chloronaphthylene, 1.8 mmol dimethylamine, 5 mol% **C1**, 110 °C) the title product was isolated as a brown oil in 57% yield. A 2% ethyl acetate/hexanes eluent system was used for column chromatography on silica gel. ^{1}H NMR (300 MHz, $CDCl_3$): δ 8.29-8.27 (m, 1H), 7.87-7.84 (m, 1H), 7.57-7.48 (m, 3H), 7.46-7.40 (m, 1H), 7.13-7.10 (m, 1H), 2.95-2.94 (m, 6H); ^{13}C{^{1}H} NMR (75.5 MHz, $CDCl_3$): δ 145.7, 145.0, 143.4, 140.4, 138.2, 130.1, 122.0, 103.3, 30.5. Spectral data are in agreement with the literature.[9]

4-Methoxy-*N*-octylaniline (**4e**)

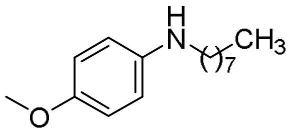

Following **GPF**: (0.50 mmol 4-chloroanisole, 0.55 mmol octylamine, 5 mol% **C1**, 60 °C) the title product was isolated as a dark yellow oil in 85% yield. A 5% ethyl acetate/hexanes eluent system was used for column chromatography on silica gel. ^{1}H NMR (300 MHz, $CDCl_3$): δ 6.82-6.79 (m, 2H), 6.62-6.58 (m, 2H), 3.77 (s, 3H), 3.35 (br s, 1H), 3.11-3.06 (m, 2H), 1.67-1.58 (m, 2H), 1.46-1.31 (m, 10H), 0.91 (t, *J* = 11.3 Hz, 3H); ^{13}C{^{1}H} NMR (75.5 MHz, $CDCl_3$): δ 152.0, 143.0, 114.9, 114.0, 55.9, 45.0, 31.8, 29.7, 29.4, 29.3, 27.2, 22.7, 14.1. Spectral data are in agreement with the literature.[10] Following **GPF** the title product was isolated as a dark yellow oil in 93% yield from the corresponding bromide (5 mol% **C1**, 60 °C).

N-Octyl-4-(trifluoromethyl)aniline (**4f**)

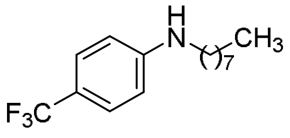

Following **GPF**: (0.50 mmol 1-chloro-4-(trifluoromethyl)benzene, 0.55 mmol octylamine, 1 mol% **C1**, 25 °C) the title product was isolated as a yellow oil in 70% yield. A 1% ethyl acetate/hexanes eluent system was used for column chromatography on silica gel. ^{1}H NMR (500 MHz, $CDCl_3$): δ 7.44-7.42 (m, 2H), 6.63-6.61 (m, 2H), 3.97 (br s, 1H), 3.19-3.15 (m, 2H), 1.69-1.64 (m, 2H), 1.47-1.42 (m, 2H), 1.37-1.32 (m, 8H), 0.93 (t, *J* = 6.9 Hz, 3H); ^{13}C{^{1}H} NMR (125.8 MHz, $CDCl_3$): δ 151.1, 126.8 (d, J_{CF} = 3.7

Hz), 126.4 (d, J_{CF} = 269 Hz), 118.8 (d, J_{CF} = 38.4 Hz), 112.0, 43.8, 32.1, 29.6, 29.5, 29.4, 27.4, 22.8, 14.3. Spectral data are in agreement with the literature.[10] Following **GPF** (25 °C) the title product was isolated as a yellow oil in 52% yield from the corresponding bromide (3 mol% **C1**).

4-(Octylamino)benzonitrile (**4g**)

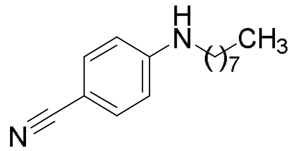

Following **GPF**: (0.50 mmol 4-chlorobenzonitrile, 0.55 mmol octylamine, 1 mol% **C1**, 25 °C) the title product was isolated as a yellow solid in 80% yield. A 5% ethyl acetate/hexanes eluent system was used for column chromatography on silica gel. ^{1}H NMR (500 MHz, $CDCl_3$): δ 7.46-7.44 (m, 2H), 6.58-6.56 (m, 2H), 4.18 (br s, 1H), 3.19-3.15 (m, 2H), 1.69-1.63 (m, 2H), 1.46-1.40 (m, 2H), 1.38-1.32 (m, 8H), 0.92 (t, J = 6.9 Hz, 3H); ^{13}C{^{1}H} NMR (125.8 MHz, $CDCl_3$): δ 151.6, 133.9, 130.1, 120.8, 112.3, 98.7, 43.5, 32.0, 29.5, 29.4, 27.3, 22.9, 14.3. Spectral data are in agreement with the literature.[10]

2,5-Dimethyl-*N*-octylaniline (**4h**)

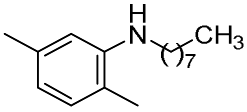

Following **GPF**: (0.50 mmol 2-chloro-1,4-dimethylbenzene, 0.55 mmol octylamine, 1 mol% **C1**, 25 °C) the title product was isolated as a yellow oil in 75% yield. A 1% ethyl acetate/hexanes eluent system was used for column chromatography on silica gel. ^{1}H NMR (500 MHz, $CDCl_3$): δ 6.98-6.96 (m, 1H), 6.51-6.48 (m, 2H), 3.43 (br s, 1H), 3.18 (t, J = 6.6 Hz, 2H), 2.34 (s, 3H), 2.13 (s, 3H), 1.74-1.68 (m, 2H), 1.50-1.44 (m, 2H), 1.38-1.34 (m, 8H), 0.94 (t, J = 7.1 Hz, 3H); ^{13}C{^{1}H} NMR (125.8 MHz, $CDCl_3$): δ 146.6, 136.9, 130.1, 118.9, 117.5, 110.8, 44.3, 32.6, 29.9, 29.7, 29.5, 27.4, 22.9, 21.9, 17.3, 14.4. Spectral data are in agreement with the literature.[10]

2’,6’-Dimethoxy-*N*-octylamine (**4i**)

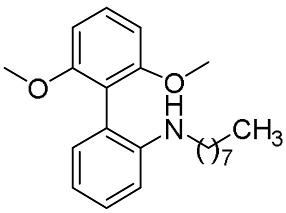

Following **GPF**: (0.50 mmol 2′-bromo-2,6-dimethoxybiphenyl, 0.55 mmol octylamine, 5 mol% **C1**, 25 °C) the title product was isolated as a light brown oil in 98% yield. A 0% to 5% ethyl acetate/hexanes

eluent system was used for column chromatography on silica gel. ^{1}H NMR (300 MHz, $CDCl_3$): δ 7.34 (t, *J* = 8.4 Hz, 1H), 7.29-7.23 (m, 1H), 7.04-7.01 (m, 1H), 6.80-6.74. (m, 2H), 6.70 (s, 1H), 6.68 (s, 1H), 3.74 (s, 6H), 3.47 (br s, 1H), 3.14-3.09 (m, 2H), 1.56-1.46 (m, 2H), 1.34-1.27 (m, 10H), 0.92-0.88 (m, 3H); ^{13}C{^{1}H} NMR (75.5 MHz, $CDCl_3$): δ 158.4, 146.5, 131.2, 129.1, 128.5, 120.0, 116.4, 116.0, 110.5, 104.3, 56.0, 44.2, 31.9, 29.4, 29.3, 29.2, 27.0, 22.6, 14.1; HRMS *m/z* ESI^{+} found 342.2428 $[M+H]^{+}$ calculated for $C_{22}H_{32}NO_2$ 342.2433.

Methyl-(2-methyl-quinolin-4-yl)amine (**4j**)

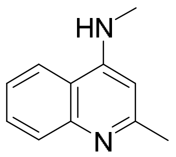

Following **GPG**: (0.50 mmol 4-chloroquinaldine, 3.5 mmol methylamine, 5 mol% **C1**, 25 °C) the title product was isolated as a white solid in 55% yield. A 2% trimethylamine, 38% hexane, 60% ethyl acetate eluent system was used for column chromatography on silica gel. ^{1}H NMR (500 MHz, $CDCl_3$): δ 7.97-7.95 (m, 1H), 7.70-7.68 (m, 1H), 7.65-7.61 (m, 1H), 7.42-7.38 (m, 1H), 6.38 (s, 1H), 5.00 (br s, 1H), 3.09 (d, *J* = 5.0 Hz 3H), 2.67 (s, 3H); ^{13}C{^{1}H} NMR (125.8 MHz, $CDCl_3$): δ 159.9, 150.8, 148.4, 129.5, 129.2, 124.1, 119.2, 117.6, 99.0, 30.3, 26.0. Spectral data are in agreement with the literature.[11] Following **GPD**: (1.0 mmol 4-chloroquinaldine, 5.0 mmol methylammonium chloride, 1 mol% **C1**, 140 °C, 5 minutes) the title product was isolated as a white solid in 99% yield.

N-Ethyl-2-methylquinolin-4-amine (**4k**)

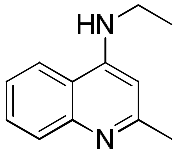

Following **GPD**: (1.0 mmol 4-chloroquinaldine, 5.0 mmol ethylammonium chloride, 1 mol% **C1**, 140 ˚C, 5 minutes) the title product was isolated as a white solid in 95% yield. ^{1}H NMR (300 MHz, DMSO): δ 8.16-8.13 (m, 1H), 7.69-7.66 (m, 1H), 7.50-7.57 (m, 1H), 7.28-7.36 (m, 1H), 6.96-6.93 (m, *J* = 5.1 Hz, 1H), 6.33 (s, 1H), 3.23-3.34 (m, 2H), 2.16 (s, 3H), 1.27 (t, *J* = 7.1 Hz, 3H); ^{13}C{^{1}H} (75.5 MHz, DMSO): δ 158.6, 149.8, 148.0, 128.5, 128.2, 122.8, 121.4, 117.4, 97.9, 36.9, 25.2, 13.7; HRMS *m/z* ESI^{+} found 187.1230 $[M+H]^{+}$ calculated for $C_{12}H_{15}N_2$ 187.1191.

sec-Butyl-(2-methyl-quinolin-4-yl)amine (**4l**)

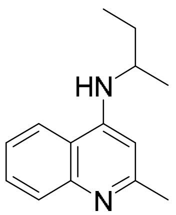

Following **GPF**: (0.50 mmol 4-chloroquinaldine, 0.55 mmol *sec*-butylamine, 5 mol% **C1**, 25 °C) the title product was isolated as a white solid in 65% yield. A 1% trimethylamine, 39% hexane, 60% ethyl acetate eluent system was used for column chromatography on silica gel. ^{1}H NMR (300 MHz, $CDCl_3$): δ 7.93-7.90 (m, 1H), 7.69-7.65 (m, 1H), 7.63-7.58 (m, 1H), 7.40-7.35 (m, 1H), 6.35 (br s, 1H), 4.74-4.72 (m, 1H), 3.74-3.60 (m, 1H), 2.64 (s, 3H), 1.83-1.59 (m, 2H), 1.34 (d, *J* = 6.8 Hz, 3H), 1.04 (t, *J* = 7.4 Hz, 3H); ^{13}C{^{1}H} NMR (125.8 MHz, $CDCl_3$): δ 159.8, 149.0, 148.8, 129.6, 129.2, 123.9, 119.1, 117.6, 99.4, 49.7, 29.7, 26.1, 20.1, 10.6; HRMS *m/z* ESI$^+$ found 215.1543 $[M+H]^+$ calculated for $C_{14}H_{19}N_2$ 215.1548.

Methyl-quinoxalin-6-yl-amine (**4m**)

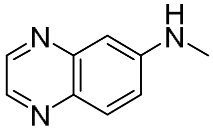

Following **GPG**: (0.60 mmol 6-chloroquinoxaline, 4.2 mmol methylamine, 5 mol% **C1**, 25 °C, 0.085M concentration of aryl halide) the title product was isolated as a bright yellow solid in 81% yield. A 60% ethyl acetate/hexanes eluent system was used for column chromatography on silica gel. ^{1}H NMR (500 MHz, $CDCl_3$): δ 8.68 (d, *J* = 2.2 Hz, 1H), 8.54 (d, *J* = 2.0 Hz, 1H), 7.88 (d, *J* = 9.1 Hz, 1H), 7.17-7.15 (m, 1H), 7.00-6.99 (m, 1H), 4.31 (br s, 1H), 3.04 (d, *J* = 5.2 Hz, 3H); ^{13}C{^{1}H} NMR (125.8 MHz, $CDCl_3$): δ 145.7, 145.0, 143.4, 140.4, 138.2, 130.1, 122.0, 103.3, 30.5. Spectral data are in agreement with the literature.[12] Following **GPD**: (1.0 mmol 6-chloroquinoxaline, 5.0 mmol methylammonium chloride 1 mol% **C1**, 140 ˚C, 15 minutes): The title product was isolated as a bright yellow solid in 99% yield.

Benzo[1,3]dioxol-5-yl-methyl-amine (**4n**)

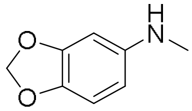

Following **GPG**: (0.60 mmol 5-Chloro-1,3-benzodioxole, 4.2 mmol methylamine, 5 mol% **C1**, 25 °C) the title product was isolated as a dark yellow oil in 80% yield. A 12% ethyl acetate/hexanes eluent system was used for column chromatography on silica gel. ^{1}H NMR (500 MHz, $CDCl_3$): δ 6.72 (d, *J* = 8.3 Hz, 1H), 6.29 (d, *J* = 2.4 Hz, 1H), 6.09-6.07 (m, 1H), 5.89 (s, 2H), 3.51 (br s, 1H), 2.83 (m, 3H); ^{13}C{^{1}H} NMR (125.8 MHz, $CDCl_3$): δ 147.2, 145.3, 139.6, 108.6, 103.8, 100.5, 95.6, 31.7. Spectral data are in agreement with the literature.[13] Following **GPF**: (5 mol% **C1**, 25 °C) the title product was isolated in 65% yield from the corresponding tosylate.

4-(*N*-Methylamino)phenylboronic acid pinacol ester (**4o**)

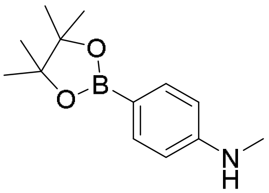

Following **GPG**: (0.24 mmol 4-chlorophenylboronic acid pinacol ester, 1.68 mmol methylamine, 3 mol% **C1**, 25 °C) the title product was isolated as a clear colourless oil in 69% yield. A 10% ethyl acetate/hexanes eluent system was used for column chromatography on silica gel. ^{1}H NMR (500 MHz, $CDCl_3$): δ 7.70-7.68 (m, 2H), 6.63-6.61 (m, 2H), 3.96 (br s, 1H), 2.89-2.88 (m, 3H), 1.37-1.36 (s, 12H); ^{13}C{^{1}H} NMR (125.8 MHz, $CDCl_3$): δ 152.0, 136.5, 111.7, 83.4, 30.5, 25.1; HRMS *m/z* ESI$^+$ found 234.1660 [M+H]$^+$ calculated for $C_{13}H_{21}BNO_2$ 234.1665.

N-Ethyl-3-methylpyridin-2-amine (**4p**)

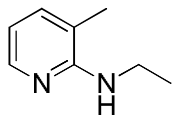

Following **GPD**: (1 mmol 2-chloro-3-methylpyridine, 5 mmol ethylammonium chloride, 5 mol% **C1**, 140 °C, 20 minutes) the title product was isolated as a light yellow oil in 73% yield. A 20% ethyl acetate/hexanes eluent system was used for column chromatography on silica gel. ^{1}H NMR (300 MHz, $CDCl_3$): δ 8.06-8.00 (m, 1H), 7.24-7.18 (m, 1H), 6.54-6.48 (m, 1H), 4.05 (br s, 1H) 3.58-3.46 (m, 2H), 2.08 (s, 3H), 1.29 (t, *J* = 7.17, 3H); ^{13}C{^{1}H} NMR (75.5 MHz, $CDCl_3$): δ 156.9, 145.5, 136.6, 116.3, 112.3, 36.4, 16.9, 15.2; HRMS *m/z* ESI$^+$ found 173.1073 [M+H]$^+$ calculated for $C_8H_{13}N_2$ 173.1034.

Cyclohexyl-(2-methyl-benzothiazol-5-yl)-amine (**4q**)

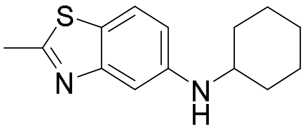

Following **GPF**: (0.50 mmol 5-Chloro-2-methylbenzothiazole, 0.55 cyclohexylamine, 5 mol% **C1**, 25 °C) the title product was isolated as a white solid in 98% yield. A 15% ethyl acetate/hexanes eluent system was used for column chromatography on silica gel. ^{1}H NMR (500 MHz, $CDCl_3$): δ 7.55 (d, *J* = 8.5 Hz, 1H), 7.12-7.18 (m, 1H), 6.70-6.68 (m, 1H), 3.66 (br s, 1H), 3.37-3.32 (m, 1H), 2.81 (s, 3H), 2.17-2.14 (m, 2H), 1.84-1.78 (m, 2H), 1.73-1.68 (m, 1H), 1.47-1.39 (m, 2H) 1.32-1.18 (m, 3H); ^{13}C{^{1}H} NMR (125.8 MHz, $CDCl_3$): δ 167.6, 155.4, 146.7, 123.8, 121.7, 114.1, 104.8, 52.4, 33.5, 26.2, 25.3, 20.3; HRMS *m/z* ESI$^+$ found 247.1263 [M+H]$^+$ calculated for $C_{14}H_{19}N_2S$ 247.1269.

N-Ethyl-2-methylbenzo[*d*]thiazol-5-amine (**4r**)

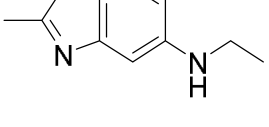

Following **GPD**: (1.0 mmol 5-chloro-2-methylbenzothiazole, 5.0 mmol ethylammonium chloride 2 mol% **C1**, 140 °C, 20 minutes) the title product was isolated as a brown solid in 52% yield. ^{1}H NMR (500 MHz, $CDCl_3$): δ 7.58-7.57 (m, 1H), 7.22-7.21 (m, 1H), 6.80-6.75 (m, 1H), 3.25 (q, *J* = 7.1 Hz, 2H), 2.21 (s, 3H),

1.33 (t, J = 7.1 Hz, 3H); $^{13}C\{^1H\}$ (125.8 MHz, $CDCl_3$): δ 167.7, 155.1, 147.2, 124.5, 121.6, 113.8, 104.8, 39.3, 20.3, 14.8; HRMS *m/z* ESI^+ found $[M+H]^+$ calculated for $C_{10}H_{13}N_2S$ 193.0755.

(4-Phenoxy-phenyl)-(1-phenyl-ethyl)-amine (**4s**)

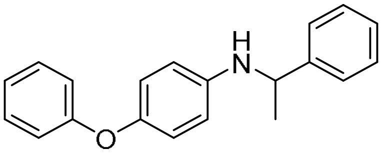

Following **GPF**: (0.50 mmol toluene-4-sulfonic acid 4-phenoxy-phenyl ester, 0.55 mmol racemic α-methylbenzylamine, 5 mol% **C1**, 25 °C) the title product was isolated as a yellow oil in 70% yield. A 5% ethyl acetate/hexanes eluent system was used for column chromatography on silica gel. ^1H NMR (500 MHz, $CDCl_3$): δ 7.43-7.41 (m, 2H), 7.39-7.36 (m, 2H), 7.31-7.26 (m, 3H), 7.04-7.01 (m, 2H), 6.94-6.92 (m, 2H), 6.86-6.84 (m, 2H), 6.56-6.53 (m, 2H), 4.51 (q, J = 13.4 Hz, 1H), 4.04 (br s, 1H), 1.58 (d, J = 6.7 Hz, 3H); $^{13}C\{^1H\}$ NMR (75.5 MHz, $CDCl_3$): δ 159.0, 147.9, 145.2, 144.0, 129.4, 128.7, 126.9, 125.9, 121.9, 121.0, 117.2, 114.2, 54.0, 25.1. Spectral data are consistent with the literature.[14] In an effort to evaluate whether such cross-couplings could be conducted with retention of stereochemistry within the α-methylbenzylamine substrate, the cross-coupling reaction was repeated using (S)-(−)-α-methylbenzylamine. In each case, the product **4s** formed from the racemic and separately the enantiopure α-methylbenzylamine starting material was dissolved in $CDCl_3$ (0.6 mL) and treated with europium tris[-(heptafluoropropylhydroxymethylene)-(+)-camphorate] (ca. 8-10 mg); the ^1H NMR spectrum of each mixture was then obtained. Notably, the ^1H NMR spectrum of **4s** synthesized from racemic α-methylbenzylamine displayed two equal-intensity methyl resonances (doublets), in keeping with the racemic nature of the α-methylbenzylamine starting material. In contrast, only a single doublet methyl resonance was observed in the case of **4s** prepared from (S)-(−)-α-methylbenzylamine under analogous conditions. These observations provide qualitative confirmation that racemization of the (S)-(−)-α-methylbenzylamine starting material under cross-coupling conditions leading to **4s** does not occur.

4-Methyl-*N*-phenylpiperazin-1-amine, (**4t**)

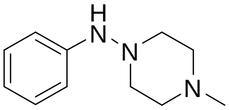

Following **GPF**: (0.50 mmol chlorobenzene, 5.5 mmol 4-methyl-1-piperazinamine, 3 mol% **C1**, 25 °C) the title product was isolated as a white solid in 76% yield. A 1% triethylamine, 2% methanol, 97% ethyl acetate eluent system was used for column chromatography on silica gel. ^1H NMR (500 MHz, $CDCl_3$): δ 7.24-7.21 (m, 2H), 6.94-6.92 (m, 2H), 6.84-6.80 (m, 1H), 4.38 (br s, 1H), 2.82 (s, 4H), 2.60 (s, 4H), 2.37 (s, 3H); $^{13}C\{^1H\}$ NMR (125.8 MHz, $CDCl_3$): δ 147.7, 129.4, 119.7, 113.9, 56.0, 55.4.3, 46.0. Spectral data are in agreement with the literature.[13] Following **GPF**: (3 mol% **C1**, 25 °C) the title product was isolated as a dark yellow oil in 76% yield from the corresponding tosylate.

Benzo[*b*]thiophen-5-yl-(3-trifluoromethyl-phenyl)- amine (**4u**)

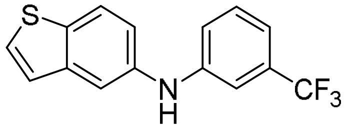

Following **GPF**: (0.50 mmol 5-chlorobenzothiophene, 0.55 mmol 3-(trifluoromethyl)aniline, 3 mol% **C1**, 25 °C) the title product was isolated as a green crystalline solid in 94% yield. A 100% hexane to 5% ethyl acetate/hexanes eluent system was used for column chromatography on silica gel. ^{1}H NMR (500 MHz, $CDCl_3$): δ 7.86 (d, *J* = 8.6 Hz, 1H), 7.62-7.61 (m, 1H), 7.52 (d, *J* = 5.4 Hz, 1H), 7.39-7.36 (m, 1H), 7.29-7.28 (m, 2H), 7.23-7.21 (m, 1H), 7.19-7.15 (m, 2H), 5.90 (br s, 1H); ^{13}C{^{1}H} NMR (125.8 MHz, $CDCl_3$): δ 145.0, 140.8, 138.6, 134.4, 132.0, 131.7, 129.9, 127.8, 123.5, 123.4, 119.2, 118.8, 116.6, 114.1, 112.7 (q, J_{CF} = 30.4 Hz); HRMS *m/z* ESI$^+$ found 294.0559 [M+H]$^+$ calculated for $C_{15}H_{11}F_3NS$ 294.0564.

(4-Methoxy-phenyl)-(3-trifluoromethyl-phenyl)- amine (**4v**)

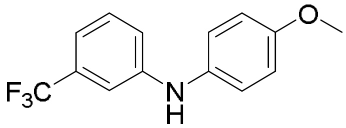

Following **GPF**: (0.50 mmol 3-chlorobenzotrifluoride, 0.55 mmol 4-methoxyaniline, 3 mol% **C1**, 25 °C) the title product was isolated as a dark oil in 97% yield. A 10% ethyl acetate/hexanes eluent system was used for column chromatography on silica gel. ^{1}H NMR (500 MHz, $CDCl_3$): δ 7.33-7.32 (m, 1H), 7.14-7.11 (m, 3H), 7.08-7.06 (m, 1H), 7.05-7.03 (m, 1H), 6.95-6.93 (m, 2H), 5.65 (br s, 1H), 3.86 (s, 3H); ^{13}C{^{1}H} NMR (125.8 MHz, $CDCl_3$): δ 156.5, 146.3, 134.6, 130.0, 123.8, 118.1, 115.9, 115.2, 111.6, 55.8. Spectral data are in agreement with the literature.[15]

(4,6-Dimethoxy-pyrimidin-2-yl)-phenethyl-amine (**4w**)

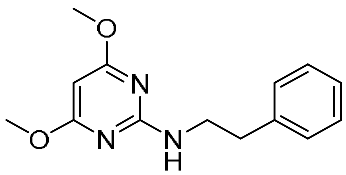

Following **GPF**: (0.50 mmol 2-chloro-4,6-dimethoxypyrimidine, 0.55 mmol phenethylamine, 3 mol% **C1**, 25 °C) the title product was isolated as a white solid in 67% yield. A 5% ethyl acetate/hexanes eluent system was used for column chromatography on silica gel. ^{1}H NMR (500 MHz, $CDCl_3$): δ 7.36-7.30 (m, 2H), 7.27-7.21 (m, 2H), 5.43 (s, 1H), 4.96 (br s, 1H), 3.87 (s, 6H), 3.72-3.65 (m, 2H), 2.93 (t, *J* = 12.0 Hz, 2H); ^{13}C{^{1}H} NMR (125.8 MHz, $CDCl_3$): δ 172.2, 161.7, 139.4, 128.8, 128.5, 78.7, 53.5, 42.8, 36.0; HRMS *m/z* ESI$^+$ found 260.1394 [M+H]$^+$ calculated for $C_{14}H_{18}N_3O_2$ 260.1399.

Furan-2-ylmethyl-(4-pyrrol-1-yl-phenyl)-amine (**4x**)

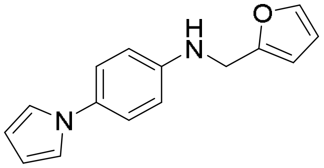

Following **GPF**: (0.50 mmol 1-(4-chlorophenyl)-1H-pyrrole, 0.55 mmol furfurylamine, 1 mol% **C1**, 25 °C) the title product was isolated as white solid in 85% yield. A 100% hexane to 5% ethyl acetate/hexanes eluent system was used for column chromatography on silica gel. ^{1}H NMR (500 MHz, $CDCl_3$): δ 7.42-7.41 (m, 1H), 7.27-7.24 (m, 2H), 7.01-6.99 (m, 2H), 6.76-6.73 (m, 2H), 6.38-6.37 (m, 1H), 6.34-6.33 (m, 2H), 6.30-6.29 (m, 1H), 4.38 (s, 2H), 4.11 (br s, 1H); $^{13}C\{^1H\}$ NMR (125.8 MHz, $CDCl_3$): δ 152.7, 146.1, 142.3, 132.8, 122.7, 120.1, 114.0, 110.7, 109.7, 107.3, 41.9; HRMS *m/z* ESI$^+$ found 239.1179 $[M+H]^+$ calculated for $C_{15}H_{15}N_2O$ 239.1184. Following **GPF**: (3 mol% **C1**) the title product was isolated as a white solid in 66% yield from the corresponding tosylate. Following **GPH**: (5 mol% **C1**) the title product was isolated as a white solid in 74% yield from the corresponding mesylate.

Phenyl-{4-[(tetrahydro-furan-2-ylmethyl)- amino]-phenyl}-methanone (**4y**)

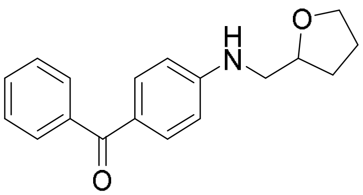

Following **GPF**: (0.50 mmol 4-benzophenone, 0.55 mmol tetrahydrofurfurylamine, 3 mol% **C1**, 25 °C) the title product was isolated as a yellow oil in 97% yield. A 10% ethyl acetate/hexanes eluent system was used for column chromatography on silica gel. ^{1}H NMR (500 MHz, $CDCl_3$): δ 7.78-7.74 (m, 4H), 7.57-7.54 (m, 1H), 7.50-7.47 (m, 2H), 6.65-6.63 (m, 2H), 4.64 (br s, 1H), 4.20-4.17 (m, 1H), 3.97-3.92 (m, 1H), 3.86-3.82 (m, 1H), 3.41-3.39 (m, 1H), 3.23-3.18 (m, 1H), 2.13-2.06 (m, 1H), 2.01-1.95 (m, 2H), 1.73-1.66 (m, 1H); $^{13}C\{^1H\}$ NMR (125.8 MHz, $CDCl_3$): δ 152.2, 139.2, 132.9, 131.1, 129.4, 128.0, 126.2, 111.8, 68.4, 47.3, 29.1, 25.8; HRMS *m/z* ESI$^+$ found 304.1308 $[M+Na]^+$ calculated for $C_{18}H_{19}NNaO_2$ 304.1313

4-Methyl-3-[(thiophen-2-ylmethyl)-amino]-benzonitrile (**4z**)

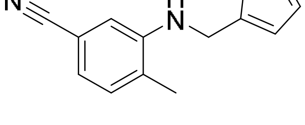

Following **GPF**: (0.50 mmol 3-bromo-4-methylbenzonitrile, 0.55 mmol 2-thiophenemethylamine, 1 mol% **C1**, 25 °C) the title product was isolated as a yellow solid in 94% yield. An 8% ethyl acetate/hexanes eluent system was used for column chromatography on silica gel. ^{1}H NMR (300 MHz, $CDCl_3$): δ 7.29-7.27 (m, 1H), 7.16-7.13 (m, 1H), 7.07-7.05 (m, 1H), 7.03-6.98 (m, 2H), 6.88-6.87 (m, 1H), 4.58 (d, *J* =

5.6 Hz, 2H), 2.22 (s, 3H); $^{13}C\{^1H\}$ NMR (125.8 MHz, $CDCl_3$): δ 146.1, 141.7, 130.9, 127.9, 127.4, 125.8, 125.3, 121.8, 120.1, 112.7, 110.9, 43.4, 18.0; HRMS *m/z* ESI^+ found 251.0613 $[M+Na]^+$ calculated for $C_{13}H_{12}N_2NaS$ 251.0619.

(2,5-Dimethyl-phenyl)-thiophen-2-ylmethyl-amine (**4aa**)

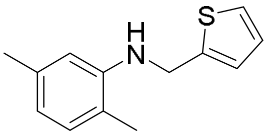

Following **GPH**: (0.50 mmol methanesulfonic acid 2,5-dimethyl-phenyl ester, 0.55 mmol 2-thiophenemethylamine, 5 mol% **C1**) the title product was isolated as a yellow oil in 87% yield. A 5% ethyl acetate/hexanes eluent system was used for column chromatography on silica gel. ^1H NMR (500 MHz, $CDCl_3$): δ 7.29-7.28 (m, 1H), 7.10-7.08 (m, 1H), 7.04-7.01 (m, 2H), 6.59-6.58 (m, 2H), 4.60 (s, 2H), 3.86 (br s, 1H), 2.34 (s, 3H), 2.17 (s, 3H); $^{13}C\{^1H\}$ NMR (125.8 MHz, $CDCl_3$): δ 145.7, 143.3, 137.0, 130.2, 127.1, 125.3, 124.8, 119.5, 118.5, 111.3, 43.7, 21.8, 17.3; HRMS *m/z* ESI^+ found 218.0998 $[M+H]^+$ calculated for $C_{13}H_{16}NS$ 218.1003.

Benzyl-quinolin-8-yl-amine (**4ab**)

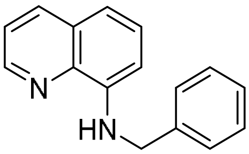

Following **GPF**: (0.50 mmol toluene-4-sulfonic acid quinolin-8-yl ester, 0.55 mmol benzylamine, 3 mol% **C1**, 25 °C) the title product was isolated as a yellow oil in 60% yield. A 5% ethyl acetate/hexanes eluent system was used for column chromatography on silica gel. ^1H NMR (500 MHz, $CDCl_3$): δ 8.77-8.76 (m, 1H), 8.12-8.10 (m, 1H), 7.50-7.48 (m, 2H), 7.43-7.30 (m, 5H), 7.11-7.10 (m, 1H), 6.70-6.69 (m, 1H), 6.55 (br s, 1H), 4.61-4.60 (m, 2H); $^{13}C\{^1H\}$ NMR (75.5 MHz, $CDCl_3$): δ 159.8, 149.0, 148.8, 129.6, 129.2, 123.9, 119.1, 117.6, 99.4, 49.7, 29.7, 26.1, 20.1, 10.6. Spectral data are consistent with the literature.[16] Following **GPH**: (3 mol% **C1**) the title product was isolated as a yellow oil in 55% yield from the corresponding mesylate.

2-Allylamino-benzonitrile (**4ac**)

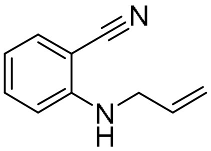

Following **GPF**: (0.12 mmol 2-chloro-benzonitrile, 0.132 mmol allylamine, 5 mol% **C1**, 60 °C) the title product was isolated as a yellow oil in 70% yield. A 30% CH_2Cl_2/hexanes eluent system was used for column chromatography on silica gel. ^1H NMR (500 MHz, $CDCl_3$): δ 7.44-7.39 (m, 2H), 6.74-6.68 (m, 2H), 5.99-5.92 (m, 1H), 5.36-5.32 (m, 1H), 5.27-5.25 (m,1H), 4.77 (br s, 1H), 3.92-3.90 (m, 2H); $^{13}C\{^1H\}$

NMR (125.8 MHz, $CDCl_3$): δ 150.3, 134.4, 134.0, 132.9, 118.1, 117.3, 116.9, 111.2, 96.1, 46.2. Spectral data are consistent with the literature.[17]

(6-Methoxy-pyridin-2-yl)-piperidin-4-ylmethyl-amine (**4ad**)

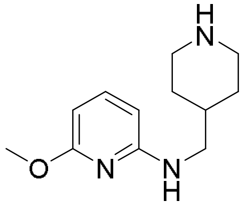

Following **GPF**: (0.50 mmol 2-chloro-6-methoxypyridine, 0.55 mmol 4-(aminomethyl)piperidine, 3 mol% **C1**, 25 °C) the title product was isolated as a clear solid in 78% yield. The reaction mixture was cooled and filtered through a short plug of alumina and washed with ethyl acetate (50 mL) and the product was collected with methanol (40 mL). After concentrating the methanol solution under reduced pressure, the crude product was purified by washing with cold hexanes (3 x 5 mL). ^{1}H NMR (300 MHz, $CDCl_3$): δ 7.34 (t, *J* = 8.0 Hz 1H), 6.04 (d, *J* = 7.8 Hz 1H), 5.95 (d, *J* = 8.0 Hz 1H), 4.53-4.49 (m, 1H), 4.04 (s, 2H), 3.85 (s, 3H), 3.39-3.35 (m, 2H), 3.26-3.21 (m, 2H), 2.81-2.72 (m, 2H), 1.94-1.87 (m, 2H), 1.59-1.45 (m, 2H); ^{13}C{^{1}H} NMR (125.8 MHz, $CDCl_3$): δ 163.9, 157.9, 140.3, 98.1, 97.5, 53.4, 47.6, 45.0, 35.6, 28.6; HRMS *m/z* ESI$^+$ found 222.1601 $[M+H]^+$ calculated for $C_{12}H_{20}N_3O$ 222.1606.

N,*N'*-Diphenyl-ethane-1,2-diamine (**4ae**)

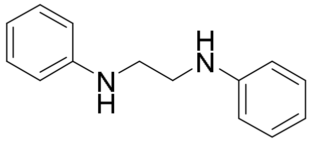

Following **GPF**: (0.50 mmol chlorobenzene, 0.55 mmol *N*-phenylethylenediamine, 3 mol% **C1**, 25 °C) the title product was isolated as a white solid in 96% yield. The reaction mixture was cooled and filtered through a short plug of silica on Celite and washed with dichloromethane (40 mL). After concentrating the so-formed mixture under reduced pressure, the crude product was purified by washing with cold hexanes (3 x 5 mL). ^{1}H NMR (300 MHz, $CDCl_3$): δ 7.26-7.21 (m, 4H), 6.80-6.75 (m, 2H), 6.70-6.68 (m, 4H), 3.88 (s, 2H), 3.43 (s, 4H); ^{13}C{^{1}H} NMR (125.8 MHz, $CDCl_3$): δ 148.0, 129.3, 117.8, 113.0, 43.3. Spectral data are consistent with the literature.[18] Following **GPF**: (3 mol% **C1**) the title product was isolated as a white solid in 98% yield from the corresponding tosylate.

N^1 -Phenyl-N^2-(pyridin-3-yl)ethane-1,2-diamine (**4af**)

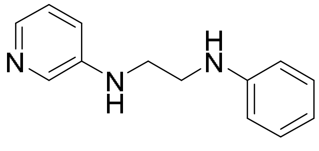

Following **GPF**: (0.50 mmol 3-chloropyridine, 0.55 mmol *N*-phenylethylenediamine, 3 mol% **C1**, 25 °C) the title product was isolated as a pale yellow oil in 65% yield. The reaction mixture was cooled and

filtered through a short plug of silica on Celite and washed with dichloromethane (40 mL). After concentrating the so-formed mixture under reduced pressure, the crude product was purified by washing with cold hexanes (3 x 5 mL). ^{1}H NMR (300 MHz, $CDCl_3$): δ 8.10-8.08 (m, 1H), 8.03-8.01 (m, 1H), 7.28-7.19 (m, 2H), 7.14-7.09 (m, 1H), 6.94-6.91 (m, 1H), 6.80-6.75 (m, 1H) 6.70-6.68 (m, 2H), 3.99 (br s, 1H), 3.88 (br s, 1H), 3.44 (s, 4H); ^{13}C{^{1}H} NMR (125.8 MHz, $CDCl_3$): δ 147.8, 144.0, 139.2, 136.3, 129.4, 123.7, 118.7, 118.0, 113.1, 43.1, 42.9. Spectral data are consistent with the literature.[19]

9-(4-Methoxyphenyl)-9H-carbazole (**4ag**)

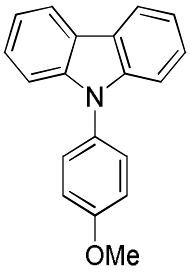

Following **GPI**: (0.2 mmol 4-chloroanisole, 0.2 mmol carbazole) the title compound was isolated as a yellow oil in 58 % yield. Purified by preparatory TLC using 10:1 hexanes:ethyl acetate. ^{1}H NMR (300 MHz, $CDCl_3$): δ 8.15-8.13 (m, 2H), 7.48-7.46 (m, 1H), 7.45-7.43(m, 1H) 7.41-7.40 (m, 1H), 7.38-7.37 (m, 1H), 7.34-7.33 (m, 1H), 7.31-7.29 (m, 1H), 7.27-7.24 (m, 2H), 7.14-7.09 (m, 2), 3.92 (s, 3H); ^{13}C{^{1}H} NMR (125.8 MHz, $CDCl_3$): δ 159.0, 141.5, 130.4, 128.7, 125.9, 123.2, 120.3, 119.7, 115.1, 109.8, 55.7. Spectral data are in agreement with the literature.[20]

4-(9H-Carbazol-9-yl)benzonitrile (**4ah**)

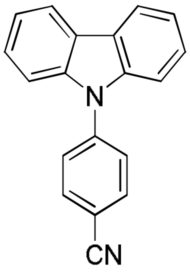

Following **GPI**: (0.2 mmol 4-chlorobenzonitrile, 0.2 mmol carbazole) the title compound was isolated as a yellow oil in 63 % yield. Purified by preparatory TLC using 10:1 hexanes:ethyl acetate. ^{1}H NMR (500 MHz, $CDCl_3$): δ 8.15 (d, J = 7.8 Hz, 2H), 7.91 (d, J = 8.4 Hz, 2H), 7.75 (d, J = 8.4 Hz, 2H), 7.45-7.43 (m, 4H), 7.35-7.32 (m, 2H); ^{13}C{^{1}H} NMR (125.8 MHz, $CDCl_3$): δ 142.8, 140.0, 133.9, 127.2, 126.4, 124.1, 121.0, 120.6, 118.2, 110.6, 109.5. Spectral data are in agreement with the literature.[21]

1-(4-Methoxy-phenyl)-1H-indole (**4ai**)

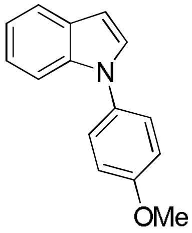

Following **GPI**: (0.2 mmol 4-chloroanisole, 0.2 mmol indole) the title compound was isolated as a yellow oil in 68 % yield. Purified by preparatory TLC using 10:1 hexanes:ethyl acetate. ^{1}H NMR (300 MHz, $CDCl_3$): δ 7.71 (d, *J* = 8.7 Hz, 1H), 7.50-7.40 (m, 3H), 7.30-7.29 (m, 1H), 7.21-7.17 (m, 2H), 7.09-7.03 (m, 2H), 6.68-67 (m, 1H), 3.90 (s, 3H); ^{13}C{^{1}H} NMR (125.8 MHz, $CDCl_3$): δ 158.5, 136.6, 133.1, 129.2, 128.5, 126.2, 122.4, 121.2, 120.3, 115.0, 110.6, 103.1, 55.8. Spectral data are in agreement with the literature.[18]

4-(1H-Pyrrolo[2,3-b]pyridin-1-yl)benzonitrile (**4aj**)

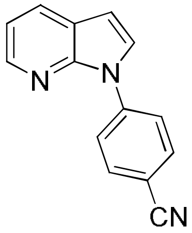

Following **GPI**: (0.2 mmol 4-chlorobenzonitrile, 0.2 mmol indole) the title compound was isolated as a yellow oil in 56 % yield. Purified by preparatory TLC using 10:1 hexanes: diisopropylamine. ^{1}H NMR (300 MHz, $CDCl_3$): δ 8.41-8.38 (m, 1H), 8.08-8.04 (m, 2H), 8.00-7.97 (m, 1H), 7.83-7.79 (m, 2H), 7.57 (d, *J* = 3.8 Hz, 1H), 7.22-7.17 (m, 1H), 6.71 (d, *J* = 3.8 Hz, 1H); ^{13}C{^{1}H} NMR (125.8 MHz, $CDCl_3$): δ 147.6, 143.9, 142.2, 133.4, 129.5, 126.4, 123.1, 122.2, 118.7, 117.6, 108.9, 103.7. HRMS *m/z* ESI$^+$ found: 220.0865 $[M+H]^+$ calculated for $C_{14}H_{10}N_3$ 220.0869.

Supplementary References

1. Borzenko, A., *et al.* Nickel-Catalyzed Monoarylation of Ammonia. *Angew. Chem. Int. Ed.* **54**, 3773-3777 (2015).
2. Lundgren, R.J., Peters, B.D., Alsabeh, P.G. & Stradiotto, M. A P,N-Ligand for Palladium-Catalyzed Ammonia Arylation: Coupling of Deactivated Aryl Chlorides, Chemoselective Arylations, and Room Temperature Reactions. *Angew. Chem. Int. Ed.* **49**, 4071-4074 (2010).
3. Green, R.A. & Hartwig, J.F. Nickel-Catalyzed Amination of Aryl Chlorides with Ammonia or Ammonium Salts. *Angew. Chem. Int. Ed.* **54**, 3768-3772 (2015).
4. Voth, S., Hollett, J.W. & McCubbin, J.A. Transition-Metal-Free Access to Primary Anilines from Boronic Acids and a Common +NH2 Equivalent. *J. Org. Chem.* **80**, 2545-2553 (2015).
5. Cheung, C.W., Surry, D.S. & Buchwald, S.L. Mild and Highly Selective Palladium-Catalyzed Monoarylation of Ammonia Enabled by the Use of Bulky Biarylphosphine Ligands and Palladacycle Precatalysts. *Org. Lett.* **15**, 3734-3737 (2013).
6. Lou, Y.-H., *et al.* The Acute Hepatotoxicity of Tacrine Explained by 1H NMR Based Metabolomic Profiling. *Toxicol. Res.* **4**, 1465-1478 (2015).
7. Schön, U., *et al.* An Improved Synthesis of 3-Aminoestrone. *Tetrahedron Lett.* **46**, 7111-7115 (2005).
8. Shen, Q., Ogata, T. & Hartwig, J.F. Highly Reactive, General and Long-Lived Catalysts for Palladium-Catalyzed Amination of Heteroaryl and Aryl Chlorides, Bromides, and Iodides: Scope and Structure–Activity Relationships. *J. Am. Chem. Soc.* **130**, 6586-6596 (2008).
9. Lewis, R.S., Wisthoff, M.F., Grissmerson, J. & Chain, W.J. Metal-Free Functionalization of N,N-Dialkylanilines via Temporary Oxidation to N,N-Dialkylaniline N-Oxides and Group Transfer. *Org. Lett.* **16**, 3832-3835 (2014).
10. Ge, S., Green, R.A. & Hartwig, J.F. Controlling First-Row Catalysts: Amination of Aryl and Heteroaryl Chlorides and Bromides with Primary Aliphatic Amines Catalyzed by a BINAP-Ligated Single-Component Ni(0) Complex. *J. Am. Chem. Soc.* **136**, 1617-1627 (2014).
11. Teguh, S.C., *et al.* Novel Conjugated Quinoline–Indoles Compromise Plasmodium falciparum Mitochondrial Function and Show Promising Antimalarial Activity. *J. Med. Chem.* **56**, 6200-6215 (2013).
12. Green, R.A. & Hartwig, J.F. Palladium-Catalyzed Amination of Aryl Chlorides and Bromides with Ammonium Salts. *Org. Lett.* **16**, 4388-4391 (2014).
13. Alsabeh, P.G. & Stradiotto, M. Addressing Challenges in Palladium-Catalyzed Cross-Couplings of Aryl Mesylates: Monoarylation of Ketones and Primary Alkyl Amines. *Angew. Chem. Int. Ed.* **52**, 7242-7246 (2013).
14. Liu, X.-Y. & Che, C.-M. Highly Enantioselective Synthesis of Chiral Secondary Amines by Gold(I)/Chiral Brønsted Acid Catalyzed Tandem Intermolecular Hydroamination and Transfer Hydrogenation Reactions. *Org. Lett.* **11**, 4204-4207 (2009).
15. Zhang, Y., César, V., Storch, G., Lugan, N. & Lavigne, G. Skeleton Decoration of NHCs by Amino Groups and its Sequential Booster Effect on the Palladium-Catalyzed Buchwald–Hartwig Amination. *Angew. Chem. Int. Ed.* **53**, 6482-6486 (2014).
16. Du, Y., Oishi, S. & Saito, S. Selective N-Alkylation of Amines with Alcohols by Using Non-Metal-Based Acid–Base Cooperative Catalysis. *Chem. Eur. J.* **17**, 12262-12267 (2011).
17. Kitov, P.I., Vinals, D.F., Ng, S., Tjhung, K.F. & Derda, R. Rapid, Hydrolytically Stable Modification of Aldehyde-Terminated Proteins and Phage Libraries. *J. Am. Chem. Soc.* **136**, 8149-8152 (2014).

18. Crawford, S.M., Lavery, C.B. & Stradiotto, M. BippyPhos: A Single Ligand With Unprecedented Scope in the Buchwald–Hartwig Amination of (Hetero)aryl Chlorides. *Chem. Eur. J.* **19**, 16760-16771 (2013).
19. Tardiff, B.J., McDonald, R., Ferguson, M.J. & Stradiotto, M. Rational and Predictable Chemoselective Synthesis of Oligoamines via Buchwald–Hartwig Amination of (Hetero)Aryl Chlorides Employing Mor-DalPhos. *J. Org. Chem.* **77**, 1056-1071 (2012).
20. Zhou, Y. & Verkade, J.G. Highly Efficient Ligands for the Palladium-Assisted Double N-Arylation of Primary Amines for One-Sep Construction of Carbazoles. *Adv. Synth. Catal.* **352**, 616-620 (2010).
21. Creutz, S.E., Lotito, K.J., Fu, G.C. & Peters, J.C. Photoinduced Ullmann C–N Coupling: Demonstrating the Viability of a Radical Pathway. *Science* **338**, 647-651 (2012).
